# Supplementary material for: The Triassic turtle of Thailand – revision of ‘Proganochelys’ ruchae
Source: PLoS One. 2025 Mar 19;20(3):e0316338. doi: 10.1371/journal.pone.0316338 (PMC12279380; doi:10.1371/journal.pone.0316338)
Supplement: S1 Text — (DOCX) [file pone.0316338.s001.docx]

**Contents**

[Institutional abbreviations 2](#_Toc183478998)

[Sources for scoring 3](#_Toc183478999)

[Changed scores 8](#_Toc183479000)

[Character list 14](#_Toc183479001)

[Matrix 23](#_Toc183479002)

[Trees and synapomorphies 41](#_Toc183479003)

[Strict consensus 41](#_Toc183479004)

[Majority rule (50%) 78](#_Toc183479005)

[References 107](#_Toc183479006)

Institutional abbreviations

**CSMM,** Carl-Schweizer-Museum Murrhardt, Murrhardt, Germany.

**ISEZ**, Institute of Systematics and Evolution of Animals, Polish Academy of Sciences, Cracow, Poland.

**IVPP**, Institute of Vertebrate Paleontology and Paleoanthropology, Chinese Academy of Sciences, Beijing, China.

**JM**, Jura-Museum Eichstätt, Eichstätt, Germany.

**MAGNT**, Museum and Art Gallery of the Northern Territory, Darwin, Australia.

**MB**, Museum für Naturkunde, Berlin, Germany.

**MNHN**, Muséum National d'Histoire Naturelle, Paris, France.

**MVZ**, Herpetology Collection (Arctos), Museum of Vertebrate Zoology, University of California, Berkeley, USA.

**NHMD**, Natural History Museum of Denmark, Copenhagen, Denmark.

**NHMUK**, The Natural History Museum, London, UK.

**NMMNH**, New Mexico Museum of Natural History and Science, Albuquerque, USA.

**NMS**, Naturmuseum Solothurn, Solothurn, Switzerland.

**OUMNH**, Oxford University Museum of Natural History, Oxford, UK.

**PRC,** Palaeontological Research and Education Centre, Mahasarakham University, Mahasarakham, Thailand (former CY).

**PULR**, Universidad Nacional de La Rioja, La Rioja, Argentina.

**PVSJ** Paleontología de Vertebrados, Museo de Ciencias Naturales de San Juan, San Juan, Argentina.

**SAAF**, St. Augustine Alligator Farm and Zoological Park, St. Augustine, USA.

**SM**, Sirindhorn Museum, Department of Mineral Resources, Kalasin, Thailand (former TF, Department of Mineral Resources, Bangkok, Thailand).

**SMF**, Sauriermuseum Frick, Frick, Switzerland.

**SMNS**, Staatliches Museum für Naturkunde Stuttgart, Stuttgart, Germany.

**UF**, Florida Museum of Natural History, University of Florida, Gainesville, USA.

**UMMZ**, Museum of Zoology, University of Michigan, Ann Arbor, USA.

**USNM**, National Museum of Natural History, Vertebrate Zoology, Smithsonian Institution, Washington DC, USA.

**YPM**, Yale Peabody Museum of Natural History (Vertebrate Zoology), New Haven, USA.

**ZPAL**, Institute of Paleobiology, Polish Academy of Sciences, Warsaw, Poland.

Sources for scoring

3D models and/or CT data with ark identifiers were obtained from MorphoSource ([www.morphosource.org](http://www.morphosource.org)). Louisiana State University & Agricultural and Mechanical College provided access to the LSUMZ data, the collection of which was funded by oVert TCN; NSF DBI-1701402; NSF DBI-1701714. University of California-Berkeley provided access to the MVC data, the collection of which was funded by oVert TCN; NSF DBI-1701713; NSF DBI-1701714; NSF DBI-1701797. Photographs of *Myuchelys latisternum* MCZ:Herp:R-35011 were examined from the MCZBASE: The Database of the Zoological Collections of the Museum of Comparative Zoology (<https://mczbase.mcz.harvard.edu>).

*Adocus beatus*: [1,2].

*Anosteira ornata*: [1].

*Apalone spinifera*: LSUMZ:Herps:93180 (ark:/87602/m4/M77347), USNM:Herp:USNM 313653 (ark:/87602/m4/521201).

*Araripemys barretoi*: [3,4].

*Baena arenosa*: [1,5].

*Baptemys wyomingensis*: [1].

*Basilemys variolosa*: [1].

*Boremys pulchra*: [1,5–7].

*Caretta caretta*: [10], USNM:HERP:214140 (ark:/87602/m4/520088, ark:/87602/m4/520092, ark:/87602/m4/520515), YPM:VZ:YPM Herr 002957 (ark:/87602/m4/M72695).

*Carettochelys insculpta*: [11].

*Chelodina* (*Macrochelodina*) *oblonga* (subjective senior synonym of *Chelodina colliei* – see Boulenger [12], Shea *et al.* [13], Rhodin *et al.* [14], and references therein): [12,13].

*Chelodina longicollis*: MVZ:Herp:106891 (ark:/87602/m4/450718, ark:/87602/m4/450724, ark:/87602/m4/450733).

*Chelonia mydas*: [10], USNM:Herp:USNM 220785 (ark:/87602/m4/520444), YPM:VZ:YPM Herr 018243 (ark:/87602/m4/450762, ark:/87602/m4/450766, ark:/87602/m4/450772, ark:/87602/m4/M167773).

*Chelonioidis chilensis*: UF:Herp:52530 (ark:/87602/m4/423473).

*Chelonioidis gringorum*: [15,16].

*Chelus fimbriatus*: [17], UF:Herp:85199 (ark:/87602/m4/M56545).

*Chelydra serpentina*: [18], ISEZ uncat.

*Chisternon undatum*: [1,5].

*Chrysemys picta*: YPM:VZ:YPM Herr 019741 (ark:/87602/m4/451169, ark:/87602/m4/451175, ark:/87602/m4/451184, ark:/87602/m4/M72850).

*Chubutemys coppeloi*: [19,20].

*Condorchelys antiqua*: [21,22].

*Dermatemys mawii*: [1], UMMZ:Herps:75286 (ark:/87602/m4/451675, ark:/87602/m4/451681, ark:/87602/m4/451685, ark:/87602/m4/M82213).

*Dermochelys coriacea*: [23].

*Dinochelys whitei*: [24].

*Dracochelys bicuspis*: [25].

*Echmatemys wyomingensis*: [1].

*Eileanchelys waldmani*: [26,27].

*Elseya dentata*: MAGNT:REPTILE:R24818 (ark:/87602/m4/538445), UMMZ:Herps:203554 (ark:/87602/m4/451690, ark:/87602/m4/451696, ark:/87602/m4/M82207).

*Emarginachelys cretacea*: [28].

*Emys orbicularis*: ISEZ R/444.

*Erymnochelys madagascariensis*: [29], MVZ:Herp:238759 (ark:/87602/m4/451757, ark:/87602/m4/451759, ark:/87602/m4/451766), YPM:VZ:YPM Herr 018118 (ark:/87602/m4/M72700).

*Eurotestudo hermanni*: MVZ:Herp:238087 (ark:/87602/m4/385515).

*Geoclemys hamiltonii*: [30], UF:Herp:85201 (ark:/87602/m4/423481, ark:/87602/m4/451769, ark:/87602/m4/451775, ark:/87602/m4/451784).

*Glyptops plicatulus*: [1,24].

*Gopherus polyphemus*: [31], MVZ:Herp:53943 (ark:/87602/m4/423824), OUMNH:ZC:21297 (ark:/87602/m4/451909, ark:/87602/m4/451914, ark:/87602/m4/451923), USNM:Herp:USNM 540796 (ark:/87602/m4/520145).

*Hangaiemys hoburensis*: [32].

*Heckerochelys romani*: [33].

*Hoplochelys crassa*: [1].

*Indochelys spatulata*: [34,35].

*Judithemys sukhanovi*: [36].

*Jurassichelon oleronensis*: [37].

*Kallokibotion bajazidi*: [38–40].

*Kayentachelys aprix*: [41,42].

*Keuperotesta limendorsa*: [43–46], SMNS 17757.

*Kinosternon flavescens*: MVZ:Herp:244070 (ark:/87602/m4/385683).

*Lissemys punctata*: [47,48], MVZ:Herp:248398 (ark:/87602/m4/452453, ark:/87602/m4/452446, ark:/87602/m4/452449, ark:/87602/m4/M66482).

*Macrochelys temminckii*: [18,49,50].

*Meiolania platyceps*: [51].

*Mesodermochelys undulatus*: [52].

*Mongolemys elegans*: [53,54], ZPAL MgCh/21, ZPAL MgCh/74a, ZPAL MgCh/75, ZPAL MgCh/77, ZPAL MgCh/79.

*Mongolochelys efremovi*: [55,56].

*Myuchelys latisternum*: MCZ:Herp:R-35011.

*Naomichelys speciosa*: [57].

*Neurankylus eximus*: [5,58].

*Niolamia argentina*: [59].

*Notoemys laticentralis*: [60–62].

*Notoemys oxfordiensis*: [61,63].

*Notoemys zapatocaensis*: [61,64,65].

*Odontochelys semitestacea*: [45,66–74], IVPP V 13240, IVPP V 15639, IVPP V 15653.

*Ordosemys leios*: [75].

*Otwayemys cunicularius*: [76].

*Palaeochersis talampayensis*: [77–80], PULR 068.

*Patagoniaemys gasparinae*: [81].

*Pelodiscus sinensis*: [47], OUMNH:ZC:08801 (ark:/87602/m4/355703, ark:/87602/m4/452660, ark:/87602/m4/452667, ark:/87602/m4/452670).

*Pelomedusa subrufa*: UMMZ:Herps:197253 (ark:/87602/m4/452676, ark:/87602/m4/452682, ark:/87602/m4/452685, ark:/87602/m4/M70051).

*Phrynops geoffroanus*: [82,83].

*Plastomenus thomasii*: [1,48].

*Platychelys oberndorferi*: [84,85].

*Platysternon megacephalum*: UF:Herp:76497 (ark:/87602/m4/M39799, ark:/87602/m4/M39884).

*Plesiobaena antiqua*: [5,86].

*Plesiochelys etalloni*: [84,87,88], NMS 8475, NMS 8696, NMS 9153.

*Pleurosternon bullockii*: [89,90].

*Podocnemis expansa*: USNM:Herp:USNM 222470 (ark:/87602/m4/520242, ark:/87602/m4/520945).

*Prochelidella cerrobarcinae*: [91].

*Proganochelys quenstedtii*: [9,50,92–96], MB.1910.45.2, MB.1910.45.3, SMF 09-F2, SMNS 10012, SMNS 15759, SMNS 16980, SMNS 17203, SMNS 17204.

*Proterochersis porebensis*: [44–46,97–102] and complete hypodigm, including ZPAL V. 39/1, ZPAL V. 39/2, ZPAL V. 39/4, ZPAL V. 39/22, ZPAL V. 39/48, ZPAL V. 39/49, ZPAL V. 39/50, ZPAL V. 39/72, ZPAL V. 39/.164, ZPAL V. 39/170, ZPAL V. 39/370, ZPAL V. 39/402, ZPAL V. 39/377, ZPAL V. 39/378, ZPAL V. 39/385, ZPAL V. 39/404, ZPAL V. 39/416, ZPAL V. 39/420, ZPAL V. 39/501, ZPAL V. 39/503.

*Proterochersis robusta*: [9,44–46,98–100,103,104], CSMM uncat., NHMUK 38650, NHMUK 38651, NHMUK 38652, NHMUK 38653, SMNS 11396, SMNS 12777, SMNS 16442, SMNS 16603, SMNS 17561, SMNS 17755, SMNS 17755a, SMNS 17930, SMNS 18440, SMNS 50917, SMNS 56606.

*Santanachelys gaffneyi*: [105].

*Siamochelys peninsularis*: [106].

*Sichuanchelys chowi*: [107,108].

*Sichuanchelys palatodentata*: [109].

*Sinemys lens*: [110].

*Solnhofia parsonsi*: [111], JM SCHA 70.

*Staurotypus triporcatus*: [2], UF:Herp:33255 (ark:/87602/m4/453759, ark:/87602/m4/453765, ark:/87602/m4/453793), MVZ:Herp:270006 (ark:/87602/m4/423808).

*Sternotherus odoratus*: YPM:VZ:YPM Herr 019736 (ark:/87602/m4/453961, ark:/87602/m4/453964, ark:/87602/m4/453972).

*Stylemys nebrascensis*: [1,112], NHMD 163497, NHMD 163498.

*Thaichelys ruchae*: [8,9], MNHN.F.THA10, MNHN.F.THA11, MNHN.F.THA12, MNHN.F.THA13, MNHN.F.THA14, MNHN.F.THA15, MNHN.F.THA16, MNHN.F.THA17, MNHN.F.THA18, MNHN.F.THA19, MNHN.F.THA20, MNHN.F.THA21, MNHN.F.THA22.1 (cast of TF 1440-5a), MNHN.F.THA22.2 (cast of TF 1440-5b), MNHN.F.THA22.3 (cast of TF 1440-5c), PRC 195 (former CY3-158), PRC196 (former CY3-160), PRC 197 (former CY3-211), PRC 198 (former CY3-212), PRC 199 (former CY3-213), PRC 200 (former CY3-214), and PRC 201 (former CY3-215), PRC 202 (former CY3-373), SM2015-1-001, SM2017-1-124, SM2017-1-125, SM2017-1-126, SM2017-1-127, SM2017-1-128, SM2017-1-129, SM2017-1-130, SM2017-1-131, SM2017-1-133, SM2017-1-134, SM2017-1-135, SM2017-1-136.

*Toxochelys latiremis*: [1,113].

*Trachemys scripta*: [114], SAAF unnumbered (ark:/87602/m4/505110, ark:/87602/m4/505116: [115]), USNM:Herp:USNM 521293 (ark:/87602/m4/520318, ark:/87602/m4/521134).

*Trinitichelys hiatti*: [5].

*Waluchelys cavitesta*: [79,80], PVSJ 903, PVSJ 904.

*Xenochelys formosa*: [1,116,117].

*Xinjiangchelys wusu*: [118].

*Yaminuechelys maior*: [119].

*Yehguia tatsuensis*: [120].

Changed scores

***Keuperotesta limendorsa*:**

- Dorsal vertebra B: ? to 0 (cylindrical, longer than wide, keeled ventrally). The vertebral column of SMNS 17757 is only partially preserved, but the ventral keel is evident in the visible part [43–45].
- Nuchal B, elongate costiform process of nuchal: ? to 0 (absent). There is no evidence of a costiform process in SMNS 17757 [43–45].
- Pelvis B, thyroid fenestrae: 0 (two small)/1 (two big or partially separated) to 0 (two small). The thyroid fenestrae are small in SMNS 17757, their partial enlargement is asymmetric and clearly an effect of damage [44,46].
- Supramarginal A: ? to 1 (partial row present). The surface of the carapace in SMNS 17757 is very incomplete, but a small part of the first left supramarginal, as well as the contact between the marginal, pleural, and the first vertebral scute areas are preserved anteriorly and posteriorly, allowing scoring of this character [44].

***Mongolemys elegans*:**

- Caudal B, centra: ? to 1 (formed). Based on a CT scan of ZPAL MgCh/77.
- Dorsal vertebra B: ? to 0 (cylindrical, longer than wide, keeled ventrally). Based on ZPAL MgCh/77 (CT scan).
- Epiplastron B, thick anterior border: ? to 1 (absent). Based on ZPAL MgCh/21 and ZPAL MgCh/77 (CT scan).
- Humeral B, humeropectoral sulcus: ? to 0 (only in the hyoplastra). Following Khosatzky and Młynarski [53] and confirmed on ZPAL MgCh/21, ZPAL MgCh/77, and ZPAL MgCh/79.
- Neural B, shape: ? to 1 (regular, often hexagonal, longer than wide). Following Khosatzky and Młynarski [53] and confirmed on ZPAL MgCh/21, ZPAL MgCh/75, and ZPAL MgCh/77.
- Nuchal C: ? to 1 (longer than wide or as long as wide). Following Khosatzky and Młynarski [53] and confirmed on ZPAL MgCh/77.
- Peripheral bones: ? to 0 (posterior peripheral bones without internal cavity Based on ZPAL MgCh/77 (CT scan).
- Pes C: ? to 0 (5 digits). Based on ZPAL MgCh/77.
- Suprapygal A: ? to 2 (two elements). Following Khosatzky and Młynarski [53] and confirmed on ZPAL MgCh/21, ZPAL MgCh/75, and ZPAL MgCh/77.
- Xiphiplastron A, distinct anal notch: 1 (present) to 0 (absent). Following Khosatzky and Młynarski [53] and Cadena *et al.* [54], and confirmed on ZPAL MgCh/21, ZPAL MgCh/74a, and ZPAL MgCh/75.

***Odontochelys semitestacea*:**

- Anal A: ? to 0 (only covers parts of the xiphiplastra). In agreement with Lyson *et al.* [67] – although we consider the sulcus to be more inverted V-shaped in IVPP V 13240 than drawn by those authors, we accept its position within the xiphiplastron.
- Carapace D, sculpturing of the shell: - to 0 (absent). There is no distinct unique sculpture on either costals or plastron of any *Odontochelys semitestacea* specimen [66,67,70].
- Dorsal vertebra B: ? to 0 (cylindrical, longer than wide, keeled ventrally). Based on IVPP V 15653 [72,74].
- Entoplastron C, distinct posterolateral entoplastral process: ? to 0 (present). In agreement with Nagashima *et al.* [68], based on IVPP V 15653.
- Epiplastron C, dorsal process: - to 1 (present, osseous contact with carapace absent). Following Li *et al*. [66] and Nagashima *et al.* [68], confirmed on IVPP V 15639 and IVPP V 15653.
- Extragular A: ? to 0 (present). The extragular scute borders in *Odontochelys semitestacea* IVPP V 13240 are somewhat obscured by the disarticulation of the extragular processes, but the sulcus is identifiable on the left side, just beside the edge of the process [66,67,70]. Faint probable sulci are also visible in that area in IVPP V 15639.
- Extragular B, medial contact of extragulars: ? to 0 (absent). Based on IVPP V 13240 and IVPP V 15639.
- Extragular D: ? to 0 (not reaching the entoplastron). Based on IVPP V 13240.
- Gular A: ? to 0 (one pair). The gulars in *Odontochelys semitestacea* are clearly paired, as noted by Lyson *et al.* [67]. Confirmed on IVPP V 13240.
- Humeral A: ? to 0 (one pair). The humeral outline is clearly visible in IVPP V 13240, first outlined by Lyson *et al.* [67]. There is also no sign of a plastral hinge in that region (state 1) [66,67,70].
- Humeral B, humeropectoral sulcus: ? to 0 (only in the hyoplastra). We agree with the interpretation of Lyson *et al.* [67] that the humeropectoral sulcus in *Odontochelys semitestacea* lies outside of the ventral exposition of the entoplastron.
- Humerus A: ? to 0 (ectepicondylar foramen in a channel). The channel is present in IVPP V 13240 [66,67,70]. note, however, that this character is ontogeny-dependent [46].
- Humerus B, shoulder: ? to 1 (absent). The shoulder is not present in any known specimen of *Odontochelys semitestacea*. note, however, that this character is ontogeny-dependent [46].
- Ilium A, elongated iliac neck: ? to 0 (absent). The morphology is visible in the holotype [66].
- Intergular A: ? to 0 (absent). Following Lyson *et al.* [67], confirmed on IVPP V 13240 and IVPP V 15639.
- Pectoral A: ? to 0 (present). First noticed and outlined by Lyson *et al.* [67], confirmed on IVPP V 13240 [66,70].
- Pectoral B: ? to 0 (anteroposteriorly developed). The proportions of the pectoral scutes in *Odontochelys semitestacea* are similar as in *Proterochersis* spp. [67,98].
- Pectoral girdle B: 1 (inside rib cage) to 0 (outside rib cage). *Odontochelys semitestacea* is the only taxon in the matrix for which that state is applicable [66,69].
- Pelvic girdle, ischium: ? to 1 (not covered ventrally by the plastron, seen in ventral view). Based on IVPP V 13240 and IVPP V 15639 [66,67].
- Plastral scutes A: ? to 0 (present). The scutes were clearly present on the plastron of *Odontochelys semitestacea* (particularly visible in IVPP V 13240), first noticed and drawn by Lyson *et al.* [67], confirmed on IVPP V 13240 and IVPP V 15639.
- Plastral scutes B, pronounced midline plastral sulcus sinuous: ? to 0 (absent). Mesial plastral sulcus is slightly sinuous in all Triassic stem turtles, including *Odontochelys semitestacea*, but it never deviates away from the midline so strongly as in the taxa scored in this matrix as 1 (present). Based on IVPP V 13240 and IVPP V 15639.

***Palaeochersis talampayensis*:**

- Humerus B, shoulder: ? to 1 (absent). The humeri of *Palaeochersis talampayensis* PULR 068 are damaged but no shoulder seems to be present [78]. note, however, that this character is ontogeny-dependent [46].
- Intergular A: ? to 1 (present). The scalation pattern in the Australochelyidae is very indistinct. However, both *Palaeochersis talampayensis* and *Waluchelys cavitesta* have at the anterior edges of their plastra a rounded, slightly protruding anteriorly and ventrally, mesial tubercle [77–80]. We propose that this tubercle corresponds to an epidermal scute. Given that the more anterolaterally located projections are morphologically similar to the extragular projections of *Proterochersis porebensis* and *Thaichelys ruchae*, the tubercle in question may represent either fused gulars or an intergular. Because there is a precedent when it comes to the presence of the intergular in Triassic turtles established by *Proganochelys quenstedtii* [9,50], but there is no evidence of gular fusion, the tubercle in question is here proposed to be an intergular.
- Supramarginal A: ? to 0 (complete row present)/1 (partial row present). The presence of supramarginals in *Palaeochersis talampayensis* is generally accepted [77–80], but their extent is uncertain due to poor preservation. Therefore, this character is coded here accounting for the ambiguity.

***Plesiochelys etalloni*:**

- Epiplastron C, dorsal process: ? to 2 (absent). Following Bräm [84], confirmed on NMS 8475, NMS 8696, and NMS 9153.

***Proganochelys quenstedtii*:**

- Cervical A: - to 1 (one cervical present). Following Fraas [93], Gaffney [50,92], and Scheyer *et al.* [95], confirmed on MB.1910.45.2, SMF 09-F2, SMNS 10012, and SMNS 16980.
- Intergular A: 0 (absent) to 1 (present). There is a small intergular scute in *Proganochelys quenstedtii*, termed the median scute by Gaffney [50] and the intergular scute by Broin [9]. Confirmed on SMNS 16980 and SMNS 17204.
- Multiple osteoderms forming peripherals: 0 (absent) to ?. The sutures are mostly not traceable in *Proganochelys quenstedtii* [50,92,121,122].
- Neural B: 0 (irregular in shape, wider than long) to 1 (regular, often hexagonal, longer than wide). This is clearly demonstrated by SMNS 17203, documented already by Gaffney [50].
- Pelvic girdle, ischium: ? to 1 (not covered ventrally by the plastron, seen in ventral view). Following Gaffney [50], confirmed on SMNS 16980 and SMNS 17204.

***Proterochersis porebensis*:**

- Anal A: ? to 0 (only covers parts of the xiphiplastra). The restriction of the anal scutes to the xiphiplastra is documented in ZPAL V. 39/170 [99].
- Caudal B, centra: ? to 0 (all amphicoelous). Based on caudal vertebrae found in isolation and in association with ZPAL V. 39/48 and ZPAL V. 39/72 [44,46].
- Cervical articulation A: ? to 0 (not formed). Based on isolated vertebrae found in the type locality.
- Cervical vertebra B, ventral keels: ? to 0 (absent or slightly developed in all vertebrae). The extent of keeling in cervical vertebrae of *Proterochersis porebensis*, based on isolated vertebrae found in the type locality, is similar as in *Proganochelys quenstedtii*.
- Cervical vertebra D, triangular diapophyses: ? to 0 (absent). Based on isolated vertebrae found in the type locality.
- Cervical vertebra J, postzygapophyses: ? to 0 (not united in midline). Based on isolated vertebrae found in the type locality.
- Chevron A: ? to 0 (present on nearly all caudals). The chevrons are not preserved themselves in the material of *Proterochersis porebensis*, but clear facets are present on caudal vertebral centra from the type locality.
- Costal A, medial contact of costal I: ? to 0 (absent). Based on ZPAL V. 39/22, ZPAL V. 39/48, ZPAL V. 39/49, ZPAL V. 39/72 [44–46,99].
- Costal B, medial contact of posterior costals: ? to 0 (absent). Based on ZPAL V. 39/48, ZPAL V. 39/49, ZPAL V. 39/72, ZPAL V. 39/370, ZPAL V. 39/402 [44,46,98,99].
- Dorsal rib B, contact of the last two dorsal rib pairs with costals: ? to 0 (present). Based on ZPAL V. 39/48, ZPAL V. 39/49, ZPAL V. 39/72 [44,46,99].
- Dorsal rib D, articulation tubercule on the anterior face of the first thoracic rib: ? to 0 (absent, smooth anterior face). The ribs of the first thoracic (dorsal) vertebra are absent or severely damaged in all specimens of *Proterochersis porebensis*, although they seem to be at least partially preserved in ZPAL V. 39/48 and ZPAL V. 39/72 [45]. As preserved, there is no evidence for an articulation tubercle and the morphology seems consistent with that observed in *Keuperotesta limendorsa* and suggested by steinkerns (natural internal molds) of *Proterochersis robusta* [43–46], so this character is scored accordingly. However, this assessment should be reevaluated when new, better preserved specimens become available.
- Dorsal vertebra A, anterior articulation of first dorsal centrum: ? to 0 (at most slightly anteroventrally). Based on ZPAL V. 39/48, ZPAL V. 39/72 [44–46].
- Dorsal vertebra B: ? to 0 (cylindrical, longer than wide, keeled ventrally). Based on ZPAL V. 39/48, ZPAL V. 39/49, ZPAL V. 39/72, ZPAL V. 39/377, ZPAL V. 39/378 [44–46,98].
- Dorsal vertebra C, costo-vertebral tunnel: ? to 1 (wide all along the entire length of the thoracic vertebrae). The morphology in *Proterochersis porebensis* is generally the same as in *Proterochersis robusta*. Based on ZPAL V. 39/1, ZPAL V. 39/2, ZPAL V. 39/48, ZPAL V. 39/49, ZPAL V. 39/72 [98].
- Entoplastron A, anterior entoplastral process: ? to 0 (present). Inferred from ZPAL V. 39/404, ZPAL V. 39/501, ZPAL V. 39/503 [46,99].
- Extragular D: ? to 0 (not reaching the entoplastron). Based on ZPAL V. 39/404, ZPAL V. 39/501, ZPAL V. 39/503 [46,99].
- Humerus A: ? to 0 (ectepicondylar foramen in a channel). Based on ZPAL V. 39/50 [46]. note, however, that this character is ontogeny-dependent [46].
- Humerus B, shoulder: ? to 0 (present). Based on ZPAL V. 39/50, ZPAL V. 39/.164 [46]. note, however, that this character is ontogeny-dependent [46].
- Humerus C, lateral process: ? to 0 (in the proximal end of the humerus). Based on numerous specimens, including ZPAL V. 39/50, ZPAL V. 39/.164 [46].
- Humerus D, lateral process: ? to 0 (seen in dorsal view). Based on numerous specimens, including ZPAL V. 39/50, ZPAL V. 39/.164 [46].
- Humerus E, length of the humerus: ? to 1 (more than two times the width of the proximal end). Based on numerous specimens, including ZPAL V. 39/50, ZPAL V. 39/.164 [46]. note that this character may potentially be ontogeny dependent – in large specimens the proximal end width is nearly half the humerus length, while in smaller specimens it is less [46].
- Hyo-hypoplastron A: ? to 0 (not fused). In *Proterochersis* spp. the hyo- and hypoplastra are fully separated [44,103]. We treat this character as independent from the frequent ankylosis which affects the whole shell [44,98,99,101].
- Hyoplastron A, axillary buttresses contact: ? to 0 (peripherals only). Based on ZPAL V. 39/48, ZPAL V. 39/49, ZPAL V. 39/72 [44,46,99]. Accounting for the complex structure of the carapace in *Proterochersis* spp., which involves numerous supernumerary dermal bones [99,101] that may potentially contribute to the contact with the axillary buttress, and the frequent shell ankylosis obscuring individual bone contacts [44,98,99,101], in this case the character is understood as the lack of expansion of the axillary buttress onto the costals.
- Hypoplastron A, inguinal buttresses: ? to 0 (peripherals only). Based on numerous specimens, including ZPAL V. 39/48, ZPAL V. 39/49, ZPAL V. 39/72 [44,46,99]. See Hyoplastron A above.
- Musk ducts A: ? to 0 (absent). Based on numerous specimens, including ZPAL V. 39/48, ZPAL V. 39/49, ZPAL V. 39/72 [44,46,99].
- Neural B: ? to 1 (regular, often hexagonal, longer than wide). Based on ZPAL V. 39/4, ZPAL V. 39/416 [99].
- Nuchal A, cervical articulates with the nuchal: ? to 1 (articulation absent). Based on ZPAL V. 39/22 [46,99].
- Nuchal B, elongate costiform process of nuchal: ? to 0 (absent). Based on ZPAL V. 39/22 [46,99].
- Nuchal C: ? to 0 (wider than long). Based on ZPAL V. 39/22 [46,99].
- Pelvic girdle, ischium: 1 (not covered ventrally by the plastron, seen in ventral view) to 0 (covered ventrally by the plastron)/1 (not covered ventrally by the plastron, seen in ventral view). The posterior extent of the ischium in *Proterochersis porebensis* is variable [98].

***Proterochersis robusta*:**

- Anal A: ? to 0 (only covers parts of the xiphiplastra). The restriction of the anal scutes to the xiphiplastra is documented in SMNS 16442 [99].
- Costal A, medial contact of costal I: ? to 0 (absent). Based on SMNS 16442 [99].
- Costal B, medial contact of posterior costals: ? to 0 (absent). Based on SMNS 17755a [99].
- Dorsal rib B, contact of the last two dorsal rib pairs with costals: ? to 0 (present). Based on NHMUK 38653, SMNS 11396, SMNS 12777, SMNS 17930 [44,46,100].
- Dorsal rib D, articulation tubercule on the anterior face of the first thoracic rib: ? to 0 (absent, smooth anterior face). At least partial imprints of the first thoracic (dorsal) rib seem to be present in well-preserved steinkerns (SMNS 12777, SMNS 16442, SMNS 17930) and provide no evidence of an articulation tubercle [46].
- Dorsal vertebra B: ? to 0 (cylindrical, longer than wide, keeled ventrally). Based on NHMUK 38652, SMNS 11396, SMNS 56606 [46,100].
- Dorsal vertebra C, costo-vertebral tunnel: ? to 1 (wide all along the entire length of the thoracic vertebrae). First noticed by Fraas [103], confirmed on SMNS 11396, SMNS 12777, SMNS 16442, SMNS 16603, SMNS 17930 [44,46,100].
- Entoplastron A, anterior entoplastral process: ? to 0 (present). Based on SMNS 16442 [46,99].
- Epiplastron C, dorsal process: ? to 1 (present, osseous contact with carapace absent). Based on SMNS 12777, SMNS 16603, SMNS 16442, SMNS 17930 [44,46,100].
- Extragular D: ? to 0 (not reaching the entoplastron). Based on SMNS 16442 [46,99].
- Humeral B, humeropectoral sulcus: ? to 0 (only in the hyoplastra). Based on SMNS 16442, SMNS 17755 [46,99].
- Hyoplastron A, axillary buttresses contact: ? to 0 (peripherals only). Based on SMNS 16442, SMNS 17930 [99]. See *Proterochersis porebensis*.
- Hypoplastron A, inguinal buttresses: ? to 0 (peripherals only). Based on SMNS 17930, SMNS 18440 [99]. See *Proterochersis porebensis*.
- Neural B: ? to 1 (regular, often hexagonal, longer than wide). Based on SMNS 16442 [99].
- Nuchal A, cervical articulates with the nuchal: ? to 1 (articulation absent). Based on SMNS 16442, SMNS 17930 [46,99].
- Nuchal B, elongate costiform process of nuchal: ? to 0 (absent). There is no evidence of a costiform process in body fossils nor steinkerns of *Proterochersis robusta*.
- Nuchal C: ? to 0 (wider than long). Inferred from SMNS 16442 [99].
- Pelvic girdle, ischium: ? to 0 (covered ventrally by the plastron). Based on SMNS 12777, SMNS 17561, SMNS 56606 [44,46,98,99,103,104].
- Pelvis B, thyroid fenestrae: 0 (two small)/1 (two big or partially separated) to 0 (two small). The bone around the thyroid fenestrae is very thin in *Proterochersis* spp., making it prone to damage and, as a result, an artificial enlargement of the fenestrae. However, in the undamaged *Proterochersis robusta* SMNS 16603 the fenestrae are small, consistent with the morphology observed in *Proterochersis porebensis* [44,46].

***Stylemys nebrascensis*:**

- Pelvis A: 1 (sutured) to 0 (pelvis-shell attachment by ligaments). Based on NHMD 163497 and NHMD 163498.

***Waluchelys cavitesta*:**

- Intergular A: ? to 1 (present). Based on PVSJ 903 [79]. See *Palaeochersis talampayensis*.

Character list

Ordered characters indicated with an asterisk.

1. Abdominal A*. 0: present, with medial contact; 1: present, medial contact absent; 2: absent.
2. Abdominal B*. 0: two pairs; 1: one pair; 2: absent.
3. Anal A. 0: only cover parts of the xiphiplastra; 1: anteromedially overlap onto hypoplastra.
4. Antrum postoticum A*. 0: antrum postoticum absent; 1: incipient; 2: fully developed.
5. Antrum postoticum B. 0: enlarged and laterally enclosed; 1: enlarged but not closed laterally.
6. Basiocccipital B. 0: deep C-shaped concavity between basioccipital tubera absent; 1: deep C-shaped concavity present.
7. Basioccipital A. 0: with two or one ventral basioccipital tubercle; 1: tubercle absent.
8. Basisphenoid A. 0: rostrum basisphenoidale flat; 1: rod-like, thick, and rounded.
9. Basisphenoid B. 0: paired pits on ventral surface absent; 1: present, restricted to the basisphenoid; 2: present in the basisphenoid (basisphenoid highly rugose) and developing posteriorly reaching the basioccipital; 3: present in in the basisphenoid and pterygoid or in the pterygoid.
10. Basisphenoid C. 0: reduced to a v-shaped basisphenoid trapped between the pterygoids and the basioccipital absent; 1: reduced to a v-shaped basisphenoid trapped between the pterygoids and the basioccipital present.
11. Basisphenoid D. 0: Basisphenoid shape not triangular (pentagonal/quadrangular); 1: triangular.
12. Canalis caroticum C. 0: pattern A; 1: pattern B; 2: pattern C.
13. Canalis caroticum D. 0: junction of palatine artery and internal carotid artery not enclosed in bone; 1: enclosed in bone.
14. Canalis caroticum E. 0: canalis carotici interni posterior to bifurcation in ac and ap not covered ventrally by bone; 1: covered ventrally by bone.
15. Canalis caroticum F* . 0: arteria palatina enters the skull through the interpterygoid vacuity or intrapterygoid slit; 1: enters the skull through the foramen caroticum laterale between bs and pt; 2: all ots path inside the skull.
16. Canalis caroticum G. 0: fpcci (entrance of internal carotid artery into the skull) absent; 1: formed by pterygoid; 2: formed by pterygoid and basisphenoid; 3: formed by pro, pro and bs, or pro and pt; 4: formed by bs.
17. Carapace A*. 0: carapacial scutes present; 1: partially present; 2: absent.
18. Carapace B*. 0: tricarinate carapace absent; 1: present, but only slightly; 2: present and pronounced.
19. Carapace C. 0: absent; 1: present.
20. Carapace D. 0: sculpturing of the shell absent; 1: present.
21. Carapace E. 0: sculpturing of the shell like in *Hydromedusa*; 1: like in *Pleurosternon*; 2: like in trionychians; 3: like in *Naomichelys*.
22. Carapace F. 0: the width of the posterior half of carapace is the same or is slightly wider than the anterior half; 1: pentagonal in shape, with the anterior border more or less straight and the posterior half tapering posteriorly.
23. Caudal A. 0: tail club present; 1: absent.
24. Caudal B. 0: all centra amphicoelous; 1: formed centra.
25. Caudal C. 0: anterior caudal vertebrae procoelous or platycoelous; 1: anterior caudal vertebrae opisthocoelous.
26. Caudal D. 0: posterior caudal vertebrae procoelous or platycoelous; 1: posterior caudal vertebrae opisthocoelous.
27. Cervical A*. 0: cervicals absent, carapacial scutes otherwise present; 1: one cervical present; 2: more than one cervical present.
28. Cervical articulation A. 0: not formed; 1: formed.
29. Cervical articulation H. 0: 8(dorsal; 1: 8)dorsal; 2: none, vertebrae only meet at zygapophyses.
30. Cervical articulation I. 0: double articulation between 5th and 6th absent; 1: present.
31. Cervical articulation J. 0: double articulation between 6th and 7th absent; 1: present.
32. Cervical articulation K. 0: central articulation cervical 6-7 concave-convex; 1: platycoelous.
33. Cervical articulation L. 0: double articulation between 7th and 8th absent; 1: present.
34. Cervical rib A. 0: present; 1: absent.
35. Cervical vertebra B. 0: ventral keels absent or slightly developed in all vertebrae; 1: ventral keels more developed on posterior vertebrae.
36. Cervical vertebra C. 0: cervical centrum 8<7 absent; 1: present.
37. Cervical vertebra D. 0: triangular diapophyses absent; 1: triangular diapophyses present.
38. Cervical vertebra E. 0: biconvex cervical vertebra in the middle of the neck absent; 1: present.
39. Cervical vertebra F. 0: biconvex cervical vertebra in the middle of the neck 2nd; 1: 3rd; 2: 4th; 3: 5th.
40. Cervical vertebra G. 0: biconcave cervical vertebra absent; 1: present.
41. Cervical vertebra H. 0: total height of centra and neural arch longer than the anteroposterior length of the cervical centra; 1: total height of centra and neural arch much shorter than the anteroposterior length of the cervical centra.
42. Cervical vertebra I. 0: neural arch on 8th cervical not modified; 1: neural arch on 8th cervical modified with the postzygapophyses pointing anteroventrally.
43. Cervical vertebra J. 0: postzygapophyses not united in midline; 1: postzygapophyses united in midline.
44. Cervical vertebra K. 0: ventral process on cervical 8 absent; 1: present well developed (as tall or taller than the high of the centrum).
45. Cervical vertebra L*. 0: eighth presacral vertebra co-ossified with the carapace and succeeding vertebrae; 1: eighth presacral vertebra has an intermediate (transitional) cervico-dorsal morphology, can be sutured to the carapace but not to the succeeding vertebrae; 1: eighth presacral vertebra free from the carapace and succeeding vertebrae, movable.
46. Chevron A. 0: present on nearly all caudals; 1: absent or poorly developed along posterior caudals.
47. Coracoid. 0: flat, sub-ovoid or with rounded posteromedial edge (bee-wing shaped); 1: flat, rectangular or with a distinctly angular posteromedial Edge; 2: columnar, at least at its base.
48. Coracoid foramen A. 0: length of the coracoid foramen less than half of the length of the glenoid fossa; 1: length of the coracoid foramen more than half of the length of the glenoid fossa.
49. Costal A. 0: medial contact of costal I absent; 1: present.
50. Costal B*. 0: medial contact of posterior costals absent; 1: medial contact of up to three posterior costals present; 2: medial contact of all costals present.
51. Costal C. 0: absent, costals fully or almost fully ossified, fontanelles abs or red; 1: present.
52. Costal D. 0: absence of alternative short and long ends in the lateral part of the costals; 1: presence.
53. Costal E. 0: 10 pairs; 1: 9 pairs; 2: 8 pairs or less.
54. Cranial scale P. 0: scale J formed by several scales; 1: scale J formed by only one scale.
55. Cranial scute B. 0: scute D meeting in midline no; 1: yes.
56. Cranial scute C. 0: scute X much smaller than D scute no; 1: yes.
57. Cranial scute D. 0: X scute partially separates G scales no; 1: yes.
58. Cranial scute E. 0: scutes A, B, and C forming a continuous posterolateral shelf yes; 1: no.
59. Cranial scute F. 0: D scute high; 1: low.
60. Cranial scute G. 0: B scute a recurved horn no; 1: yes.
61. Cranial scute H. 0: B scute in cross section triangular; 1: round.
62. Cranial scute I. 0: scute B and D in contact yes; 1: no.
63. Cranial scute J. 0: A scute small and not forming a large shelf no; 1: yes.
64. Cranial scute K. 0: A scute small A scute very large; 1: A scute comparable in size to B scute.
65. Cranial scute L. 0: Y and Z scutes relatively larges mal; 1: large.
66. Cranial scute M. 0: Y scute pentagonal pointing posteriorly and separating the medial contact of G scutes; 1: rectangular not separating the medial contact of G scutes.
67. Cranial scute N. 0: H scute present; 1: absent.
68. Cranial scute O. 0: scale F formed by several scales; 1: scale F formed by only one scale.
69. Cranial scutes A. 0: present; 1: absent.
70. Dentary A. 0: medial contact of dentaries fused; 1: sutured only.
71. Diploid number A. 0: 50; 1: 52; 2: 54; 3: 56; 4: 66 o 68; 5: 28-34; 6: 58.
72. Dorsal epiplastral process A. 0: lateroventroposterior excavation present, resulting in a distinct depression lateral to the anterior part of the medial ridge on the visceral surface of the entoplastron, partially roofed dorsally by the base of the dorsal epiplastral process; 1: lateroventroposterior excavation absent, no dorsally roofed depression in that area.
73. Dorsal epiplastral process B. 0: posterior relative to the body wall, separated from the rugose, scute-covered areas of the gular and extragular projections by a band of smooth bone and a flexure; 1: abutting the body wall, the anterior surface of the dorsal epiplastral process lies in the same plane and constitutes a continuation of the scute-covered surface of the extragular projection.
74. Dorsal epiplastral process C. 0: base distinctly broadened ventrally in anterior view, triangular, forming an extension of the ascending dorsolateral edge of the extragular projection; 1: base narrow, clearly separated from the dorsolateral edge of the extragular projection by a distinct difference in the angle between these structures.
75. Dorsal epiplastral process D. 0: located closer to the midline than to the lateral edge of the anterior plastral lobe, not extending significantly beyond the lateral extent of the gular scutes; 1: located more laterally, about halfway or more between the midline and the lateral edge of the anterior plastral lobe.
76. Dorsal rib A*. 0: length first thoracic rib long, extends a half or more than a half of the length of costal 1; 1: extends less than a half of costal 1.
77. Dorsal rib B. 0: contact of the two last dorsal rib pairs with costals present; 1: absent.
78. Dorsal rib C. 0: last dorsal rib long, contacting peripherals; 1: last dorsal rib short.
79. Dorsal rib D. 0: articulation tubercule on the anterior face of the first thoracic rib absent, smooth anterior face; 1: present.
80. Dorsal rib E*. 0: more than 10 pairs; 1: 10 pairs; 2: 9 pairs or less.
81. Dorsal vertebra A. 0: anterior articulation of first dorsal centrum faces at most slightly anteroventrally; 1: faces strongly anteroventrally.
82. Dorsal vertebra B. 0: cylindrical, longer than wide, keeled ventrally; 1: smooth and flat ventrally, hexagonal in shape.
83. Dorsal vertebra C. 0: costo-vertebral tunnel wide anteriorly and posteriorly; 1: wide all along the entire length of the thoracic vertebrae small the entire length.
84. Entoplastral scute. 0: absent; 1: present.
85. Entoplastron A. 0: anterior entoplastral process present; 1: absent.
86. Entoplastron B. 0: size of posterior entoplastral process long; 1: short.
87. Entoplastron C. 0: distinct posterolateral entoplastral process present; 1: absent.
88. Entoplastron D. 0: entoplastron V-shaped absent; 1: present.
89. Entoplastron E. 0: present; 1: absent.
90. Entoplastron F. 0: entoplastron tightly sutured with hyoplastron yes; 1: no.
91. Epiplastron A. 0: epiplastra and entoplastron narrow and elongate absent; 1: present.
92. Epiplastron B. 0: thick anterior border; 1: thick anterior border absent.
93. Epiplastron C*. 0: dorsal process present and in contact with the carapace; 1: present, osseous contact with carapace absent; 2: absent.
94. Epipterygoid A. 0: present, rod-like; 1: present, laminar; 2: absent.
95. Eustachian tube. 0: not enclosed in bone; 1: enclosed in bone.
96. Exoccipital A. 0: medial contact of exoccipitals dorsal to foramen magnum absent; 1: present.
97. Extragular A. 0: present; 1: absent.
98. Extragular B. 0: medial contact of extragulars absent; 1: present, contacting one another anterior to gulars; 2: present, contacting one another posterior to gulars.
99. Extragular D. 0: not reaching the entoplastron; 1: reach the entoplastron.
100. Extragular edge A. 0: anterior edge convex, straight, or nearly straight; 1: anterior edge distinctly concave in a horn-like fashion.
101. Extragular edge B. 0: lateral or anterolateral tip sharp; 1: lateral or anterolateral tip rounded.
102. Extragular edge C. 0: ventral deflection of the anterior edge present, the process concave ventrally and comma shaped in cross-section; 1: ventral deflection of the anterior edge absent, the process flat or convex ventrally and V- or U-shaped in cross-section.
103. Extragular process B. 0: dorsal, anterolaterally or laterally directed ridge extending from the tip of the extragular process to the base of the dorsal epiplastral process present and distinct; 1: absent or weak.
104. Extragular process B*. 0: large, projected about third or more of the extragular scute area; 1: minor, the anterolateral edge of the anterior plastral lobe gently scalloped; 2: none, the anterolateral edge of the anterior plastral lobe even.
105. Extragular process C. 0: dorsal surface of the projection convex; 1: dorsal surface distinctly concave in the posterolateral part of the projection.
106. Femur A. 0: articular surface of femoral head triangular in dorsal view; 1: rectangular to oval.
107. Fenestra perilymphatica A. 0: large; 1: relatively small.
108. Foramen jugulare posterius A. 0: separated from fenestra postotica; 1: coalescent with fenestra postotica.
109. Foramen jugulare posterius B. 0: separated from fenestra postotica by pterygoid; 1: separated by opisthotic and or exoccipital.
110. Foramen nervi hypoglossi (XII). 0: not covered ventrally by an extension of the pterygoid and the basioccipital; 1: covered ventrally by an extension of the pterygoid and the basioccipital; 2: covered ventrally by an extension of the bo.
111. Frontal A. 0: frontal contribution to orbit absent; 1: present.
112. Frontal B. 0: not fused; 1: fused.
113. Gular A. 0: one pair; 1: only one scute.
114. Gular edge. 0: spiky, distinctly conical rounded at the end to straight; 1: spiky, minor part of the scute width contributing to the spike.
115. Gular process*. 0: large, projected about third or more of the gular scute area; 1: minor, the anterior edge of the anterior plastral lobe gently scalloped; 2: none, the anterolateral edge of the anterior plastral lobe even.
116. Humeral A. 0: 1 pair; 1: 2 pair subdivided by a plastral hinge.
117. Humeral B. 0: humero-pectoral sulcus only in the hyoplastra; 1: humero-pectoral sulcus crossing the entoplastron.
118. Humerus A. 0: ectepicondylar foramen in a channel; 1: only a groove.
119. Humerus B. 0: shoulder present; 1: shoulder absent: pleurodires.
120. Humerus C. 0: lateral process in the proximal end of the humerus; 1: displaced from the proximal end, located in the shaft of the humerus.
121. Humerus D. 0: lateral process seen in dorsal view; 1: lateral process not seen in dorsal view.
122. Humerus E. 0: length of the humerus two times or less than the width of the proximal end; 1: length of the humerus more than two times the width of the proximal end.
123. Hyo-hypoplastron A. 0: not fused; 1: fused.
124. Hyomandibular nerve A. 0: path of hyomandibular branch facial nerve through cranio-quadrate space parallel to vena capitis lateralis; 1: independent to vena capitis lateralis.
125. Hyoplastron A. 0: axillary buttresses contact peripherals only; 1: peripherals and first costal.
126. Hyoplastron B*. 0: axillary buttress terminates on peripheral 2 or 1; 1: terminates on peripheral 3; 2: terminates on peripheral 4.
127. Hypoischium A. 0: present; 1: absent.
128. Hypoplastron A. 0: inguinal buttresses contact peripherals only; 1: peripheral and costal V; 2: peripherals, costal V, and costal VI.
129. Hypoplastron B. 0: Inguinal buttress terminates on peripheral 8; 1: 7; 2: 6.
130. Ilium A. 0: elongated iliac neck absent; 1: present.
131. Ilium B. 0: iliac scar extends from costals onto the peripherals and pygal; 1: in costals only or reaching pygal, but it does not extend onto peripherals.
132. Ilium D. 0: posterior notch in acetabulum absent; 1: present.
133. Ilium E. 0: thelial process absent; 1: present.
134. Inframarginal A. 0: present; 1: absent.
135. Inframarginal B. 0: 3 or more; 1: 2.
136. Inframarginal C. 0: axillar and inguinal not in contact; 1: axillar and inguinal in contact.
137. Intercaudal and caudal scutes. 0: absent; 1: present.
138. Intergular A. 0: absent; 1: present.
139. Ischium A. 0: with lateral processes absent; 1: with lateral processes present.
140. Jugal A. 0: jugal-squamosal contact present; 1: absent.
141. Jugal B. 0: jugal participation to upper temporal rim absent; 1: present.
142. Jugal-quadrate contact. 0: jugal clearly not in contact with quadrate, quadratojugal broad; 1: jugal nearly or clearly in contact with quadrate, quadratojugal reduced.
143. Lacrimal A. 0: present; 1: absent.
144. Manus A. 0: most digits with two shortened phalanges; 1: most digits with three elongate phalanges.
145. Manus and Pes A. 0: carpal and tarsal elements not flattened; 1: flattened.
146. Manus and Pes B. 0: hyperphalangy manus digits 4 and 5, pes digit 4 no; 1: yes.
147. Manus B*. 0: paddles absent; 1: short paddles present; 2: elongate paddles present.
148. Manus C*. 0: flippers absent; 1: short flippers present; 2: elongate flippers present.
149. Marginal A. 0: marginal scales overlap onto costals absent; 1: present.
150. Marginal B. 0: serration of posterior marginal scutes in adults pronounced, rounded tips of underlying peripherals; 1: serration of posterior marginal scutes in adults pronounced, spiky tips of underlying peripherals; 2: weak or no serration of posterior marginal scutes in adults.
151. Maxilla A. 0: do not contact each other in ventral view; 1: contacts each other in ventral view.
152. Maxilla B. 0: upper triturating surface not involving palatine or its contribution is minor; 1: involving palatine.
153. Maxilla C. 0: secondary palate formed by premaxilla, maxilla, and vomer, palatines not contacting in midline absent; 1: formed by premaxilla, maxilla, and vomer, palatines not contacting in midline present.
154. Maxilla D* . 0: triturating surface with only labial ridge present; 1: labial and lingual ridge present; 2: labial, lingual and accessory ridges present.
155. Maxilla E. 0: accessory ridge on maxilla present all along the triturating surface; 1: accessory ridge only in some sectors of the triturating surface.
156. Mesoplastron A*. 0: 2 pairs of meso with medial contact; 1: 1 pair of meso with medial contact; 2: 1 reduced pair; 3: absent.
157. Multiple osteoderms forming peripherals. 0: absent; 1: present.
158. Musk ducts A. 0: absent; 1: present.
159. Nasal A. 0: present; 1: absent.
160. Nasal B. 0: nasals contact another medially along their entire length; 1: medial contact of nasals partially or fully hindered by long anterior fl.
161. Nasal C. 0: dorsal exposure of nasal large; 1: greately reduced relative to that of all other elements.
162. Neural A. 0: neural formula 6>4<6<6<6<6 absent; 1: present.
163. Neural B. 0: irregular in shape, wider than long; 1: regular, often hexagonal, longer than wide.
164. Nuchal A. 0: cervical articulates with nuchal along a blunt facet; 1: articulation absent; 2: cervical articulates with nuchal along a raised pedestal.
165. Nuchal B*. 0: elongate costiform process of nuchal absent; 1: present, process crosses peripheral I to contact pe II and even III; 2: present, costiform process contacts peripheral 3.
166. Nuchal C. 0: wider than long; 1: longer than wide or as long as wide.
167. Nuchal emargination. 0: absent or indistinct; 1: present, excludes peripheral 1; 2: present, includes peripheral 1; 3: present, includes peripheral 2.
168. Opisthotic A. 0: loosely articulated; 1: tightly sutured.
169. Opisthotic B. 0: depressions for musculature absent; 1: present.
170. Opisthotic C*. 0: ventral ridge on opisthotic absent; 1: present, with an incipient enclosed middle ear region; 2: present, but modified with a enclosed middle ear region.
171. Opisthotic D2*. 0: processus interfenestralis, present, robust, not reaching the floor of cavum a-j; 1: present, robust, reaching the floor of cavum a-j; 2: present, small, reaching the floor of cavum a-j.
172. Palatine A. 0: palatine contribution to anterior extension of lat braincase absent; 1: present, well-developed.
173. Parietal A. 0: parietal-squamosal contact present; 1: absent.
174. Parietal B. 0: parietal contact with pt, epipt, and/or palatine absent; 1: present.
175. Parietal C. 0: length of anterior extension of the lateral braincase wall inter; 1: elongated; 2: short, enclosing the foramen nervi trigemini.
176. Parietal D. 0: overhanging process of the skull roof absent; 1: present.
177. Parietal E. 0: processus inferior parietalis forming posterior margin for nerv trigemini absent; 1: ... present.
178. Parietal F. 0: not contribute to the processus trochlearis oticum; 1: contributes to the processus trochlearis oticum.
179. Parietal G. 0: forming part of the foramen stapedio-temporalis; 1: not forming.
180. Parietal H*. 0: absent or weak, foramen stapedio-temporale concealed in dorsal view; 1: moderate, f.s.t. but not entire processes trochlearis exposed in dorsal view; 2: strong, entire processus trochlearis exposed in dorsal view.
181. Pectoral A. 0: present; 1: absent.
182. Pectoral B. 0: antero-posteriorly developed; 1: very short antero-posteriorly.
183. Pectoral girdle A*. 0: horizontal plate with a dorsal process, not triradiate, bridge closing coracoid foramen as wide or wider than the width of the coracoid foramen; 1: horizontal plate with a dorsal process, not triradiate, bridge closing coracoid foramen narrower than the width of the coracoid foramen; 1: trirradiate, bridge inexistent.
184. Pectoral girdle B. 0: pectoral girdle outside rib cage; 1: pectoral girdle inside rib cage.
185. Pelvic girdle. 0: ischium covered ventrally by the plastron; 1: ischium not covered ventrally by the plastron, ischium seen in ventral view.
186. Pelvis A. 0: pelvis-shell attachment by ligaments; 1: sutured.
187. Pelvis B*. 0: two, small separated thyroid fenestrae; 1: two, big separated fenestra or partially separated; 2: coalescent.
188. Peripheral A*. 0: more than 11 pairs; 1: 11 pairs; 2: 10 pairs less than 10 pairs.
189. Peripheral bones. 0: Posterior peripheral bones without internal cavity; 1: posterior peripheral bones with internal cavity.
190. Pes A. 0: claw on 5th digit present; 1: absent.
191. Pes B. 0: metatarsal V functions as true metatarsal; 1: metatarsal V functions as a tarsal.
192. Pes C. 0: 5 digits; 1: 4 digits.
193. Plastral kinesis A. 0: anterior; 1: anterior and posterior.
194. Plastral kinesis B. 0: between hyo and hypoplastron; 1: between hyo and epi-entoplastron.
195. Plastral scutes A. 0: present; 1: absent.
196. Plastral scutes B. 0: pronounced midline plastral sulcus sinuous absent; 1: present.
197. Plastron A. 0: connection between carapace and plastron osseous; 1: ligamentous.
198. Plastron B. 0: central plastral fontanella absent; 1: present.
199. Plastron C. 0: plastral kinesis absent; 1: present.
200. Postobital-maxilla contact. 0: absent, jugal forms part of the orbit; 1: present, jugal excluded from the orbit.
201. Postorbital A. 0: postorbital-palatine contact absent; 1: present, foramen palatinum posterius situated posterior to the orbit.
202. Prefrontal A. 0: medial contact on dorsal skull roof absent; 1: medial contact on dorsal skull roof present.
203. Prefrontal B. 0: prefrontal-vomer contact present; 1: prefrontal-vomer contact absent.
204. Prefrontal C. 0: prefrontal-palatine contact present; 1: prefrontal-palatine contact absent.
205. Prefrontal D*. 0: prefrontal exposure large; 1: reduced; 2: absent or near absent.
206. Prefrontal E. 0: prefrontal heavily sculptured present; 1: absent.
207. Premaxilla A. 0: external nares divided; 1: united.
208. Premaxilla B. 0: fusion of premaxilla absent; 1: present.
209. Premaxilla C. 0: foramen praepalatinum present; 1: absent, premaxillae well-ossified; 2: absent, foramen intermaxillaris present.
210. Premaxilla D. 0: exclusion of premaxilla from the apertura narium externa absent; 1: present.
211. Premaxilla E. 0: distinct, medial premaxillary hook along the labial margin absent; 1: present.
212. Prootic A. 0: dorsal exposure large; 1: dorsal exposure reduced or absent.
213. Pterygoid A. 0: pterygoid teeth present; 1: absent.
214. Pterygoid B*. 0: basipt process present and movable articulation; 1: basipt process present and sutured articulation; 2: basipt process absent and sutured articulation.
215. Pterygoid C. 0: triangular in shape; 1: reduced to an interpterygoid slit; 2: reduced to a paired foramen caroticum laterale.
216. Pterygoid C2. 0: Intrapterygoid slit extensive, completely covering fcb no; 1: yes.
217. Pterygoid D. 0: pterygoid-basioccipital contact absent; 1: present.
218. Pterygoid E. 0: processus trochlearis pterygoidei absent; 1: present.
219. Pterygoid F. 0: foramen palatinum posterius present; 1: present, but open laterally; 2: absent.
220. Pterygoid G. 0: medial contact of pterygoids present; 1: absent.
221. Pterygoid H. 0: pterygoid contribution to foramen palatinum posterius present; 1: absent.
222. Pterygoid I*. 0: vertical flange on lateral process absent; 1: present, almost all along the lateral process; 2: reduced.
223. Pterygoid J. 0: not reaching the exoccipitals; 1: reaching the exoccipitals.
224. Pterygoid K. 0: fossa podocnemidoidea absent; 1: present.
225. Pterygoid L. 0: processus pterygoideus externus like in *Proganochelys*; 1: like in testudinoids; 2: like in *Kayentachelys*.
226. Pterygoid M. 0: basisphenoid and pterygoid in the same level; 1: basisphenoid and pterygoid in different levels, step between both bones.
227. Pubis A. 0: lateral process small, poorly developed, columnar; 1: lateral process well developed and flat.
228. Pubis B. 0: epipubis process osseous or calcified; 1: cartilaginous or absent.
229. Pygal notch. 0: absent; 1: present.
230. Quadrate A. 0: flooring of the cranioquadrate space absent; 1: by pt, but pt does not cover the prootic; 2: by pt; 3: by qu and pro.
231. Quadrate B + C*. 0: development of the c.t. shallow, but not developed antpost; 1: shallow, but anteroposteriorly developed; 2: deep and anteroposteriorly developed.
232. Quadrate D. 0: precolumellar fossa absent; 1: large and deep.
233. Quadrate F: incisura columella auris*. 0: widely open, open all along its length, quadrate not completely rolled-up; 1: quadrate completely rolled-up, quadrate-quadrate and/or quadrate-squamosal close to each other but not sutured; 2: partially closed, quadrate completely rolled-up, quadrate-quadrate and/or quadrate-squamosal sutured to each other; 3: completely closed.
234. Quadrate G. 0: processus trochlearis oticum absent; 1: present.
235. Quadrate H. 0: processus trochlearis oticum formed by a grate contribution of quadrate; 1: small contribution of the quadrate.
236. Quadrate I. 0: quadrate-basisphenoid contact absent; 1: present.
237. Quadratojugal A. 0: present; 1: absent, due to the presence of a deep lower temporal emargination.
238. Quadratojugal B. 0: quadratojugal-maxilla contact absent; 1: present.
239. Quadratojugal C. 0: quadratojugal-squamosal contact below cavum tympani absent; 1: present.
240. Recessus scalae tympani A. 0: almost inexistent, not surrounded by bone; 1: well developed.
241. Sacrum. 0: contact of the neural spines of sacral vertebrae with carapace ossified; 1: chondral, ligamentous or none.
242. Scapula A*. 0: lamina between the dorsal process of the scapula and the acromion well developed; 1: lamina between the dorsal process of the scapula and the acromion reduced: *Kallokibotion*; 2: lamina between the dorsal process of the scapula and the acromion absent.
243. Scapulocoracoid. 0: angle between coracoid and acromion 130 degrees or less; 1: more than 130 degrees.
244. Squamosal A. 0: squamosal-postorbital contact present; 1: absent.
245. Squamosal B. 0: squamosal-supraoccipital contact absent; 1: present.
246. Squamosal C*. 0: posterolateral protuberances developing horns absent; 1: small protuberances; 2: big protuberances developed as horns.
247. Squamosal D. 0: long posterior process protruding beyond condylus occipitalis absent; 1: present.
248. Squamosal E. 0: Qu-Sq contact tightly sutured; 1: wide open.
249. Stapedial artery A. 0: posterior to fenestra ovalis between paraoccipital process and qu; 1: anterior to....
250. Stapedial artery B*. 0: relatively large; 1: significantly reduced in size; 2: absent.
251. Stapedial artery C. 0: foramen stapedio-temporalis located in the dorsal part of the otic region and points dorsally; 1: located in the anterior wall of the otic region and points anteriorly.
252. Supracaudal. 0: supracaudal scute absent; 1: supracaudal scute present as defined by Gaffney 1990 for *Proganochelys*.
253. Supramarginal A*. 0: complete row present; 1: partial row present; 2: absent.
254. Supraoccipital A. 0: crista occipitalis poorly developed; 1: protruding significantly posterior to the foramen magnum.
255. Supraoccipital B. 0: large supraoccipital exposure to dorsal skull roof absent; 1: present.
256. Supraoccipital C. 0: horizontal ventral crest in the supraoccipital absent or poorly developed anteriorly; 1: horizontal ventral crest present along all the crista supraoccipitalis.
257. Suprapygal A*. 0: none; 1: one element; 2: two elements; 3: more than 2 elements.
258. Supratemporal A. 0: present; 1: absent.
259. Tail club A. 0: with three spikes; 1: with two pairs of spikes .
260. Tail ring A. 0: absent; 1: present.
261. Tail ring B. 0: closed ventrally; 1: open ventrally.
262. Teeth A. 0: teeth present in premaxilla, maxilla, and dentary; 1: teeth absent in premaxilla, maxilla, and dentary.
263. Upper temporal fossa A. 0: present; 1: absent.
264. Vertebral A. 0: 4; 1: 5.
265. Vertebral B. 0: vertebral II-IV broader than pleurals; 1: vertebrals II-IV narrower or as narrow as pleurals.
266. Vertebral C. 0: sulcus between V 3 and 4 on neural VI; 1: on neural V.
267. Vertebral D. 0: Position of sulcus between vertebral 4 and 5 on the neural series; 1: on the suprapygals on the costals.
268. Vertebral E. 0: first vertebral scute bell-shaped, wide anteriorly and tapering posteriorly into a narrower, rounded median process invading the area of the wide second vertebral scute; 1: first vertebral scute subrectangular, hexagonal, or trapezoid with posterior edge roughly transverse and not significantly narrower than the anterior edge of the second vertebral scute; 2: first vertebral scute bell-shaped but does not invade the area of the second vertebral scute.
269. Vomer A*. 0: paired; 1: single; 2: single, greatly reduced.
270. Vomer B. 0: vomer-pterygoid contact in palatal view present; 1: absent, medial contact of palatines present.
271. Vomer C. 0: vomerine and palatine teeth present; 1: absent.
272. Vomer D. 0: vomer-premaxilla contact present; 1: absent.
273. Vomer E. 0: narrow and tall ventral crest on vomer absent; 1: present all along the vomer.
274. Vomer F. 0: domed palate absent; 1: present.
275. Xiphiplastron A. 0: distinct anal notch absent; 1: present.
276. Xiphiplastron B. 0: xiphiplastra narrow absent; 1: present.

Matrix

***Odontochelys semitestacea***? 0 ? 0 ? 0 0 ? ? 0 0 - ? ? ? ? - - 0 0 - - 1 0 - - - 0 - - - - - 0 0 ? 0 - - - ? 0 0 ? 0 0 0 0 - - - - 2 ? ? ? ? ? ? ? ? ? ? ? ? ? ? ? ? 0 ? 1 0 1 1 - - - - 0 - 0 ? 0 0 ? 0 0 0 0 0 ? 1 ? ? ? 0 0 ? (0 1) 0 1 0 0 0 0 ? - ? ? 0 0 0 1 1 0 0 0 1 0 0 1 0 ? - - 0 - - 0 - ? ? ? ? ? 0 0 ? ? 0 ? ? 1 0 1 0 0 - ? ? - 0 - - 0 - - 0 0 0 0 0 - - - - ? ? ? ? ? 0 ? ? 0 ? - ? 0 0 0 0 0 1 0 0 - - ? ? ? - - 0 0 1 0 0 ? ? 0 ? ? 0 1 0 ? ? 0 0 ? 0 0 0 - 0 0 ? 0 ? ? 0 0 2 - 0 0 - 0 0 0 ? 0 - 0 0 ? ? 0 ? 0 ? 0 ? 0 0 0 ? ? ? - - 0 0 - - 0 - 0 - 0 1 - - - ? - ? ? 0 ? ? ? 0 0

***Adocus beatus***0 1 0 2 0 ? 1 0 0 0 1 ? 0 1 1 1 0 0 1 1 2 0 1 ? ? ? 1 1 0 0 1 0 1 1 1 1 ? 0 - 0 1 1 0 1 2 ? 2 - 0 (0 1) 0 0 2 ? ? ? ? ? ? ? ? ? ? ? ? ? ? ? 0 0 ? ? ? ? ? 1 ? 1 ? ? 1 ? ? 0 1 1 1 0 0 0 0 1 2 1 0 0 0 0 0 0 1 1 ? 2 ? ? 0 1 - 1 1 0 0 1 2 0 1 0 0 0 1 1 0 0 0 0 1 0 0 1 - 0 1 0 0 - 0 0 1 1 0 0 1 1 ? ? 0 0 1 2 0 1 0 2 1 3 0 ? 1 - - 1 1 1 0 1 0 1 0 2 2 1 1 1 1 0 0 1 1 2 0 1 2 1 0 0 1 1 ? 1 1 ? - - 0 1 0 0 0 0 0 1 0 0 0 1 1 0 0 0 0 0 1 2 2 - 1 0 0 0 1 2 1 0 2 ? 0 1 0 2 2 0 3 1 1 0 0 0 0 1 ? 2 0 1 0 0 1 0 1 0 0 ? 2 1 0 1 ? 1 ? ? ? 1 1 1 1 1 1 ? 1 0 1 0 0 0 0 0

***Anosteira ornata***- ? - 2 ? ? 1 ? 0 ? ? ? ? ? ? ? 1 0 1 1 2 0 ? ? ? ? 1 ? ? ? ? ? ? ? ? ? ? ? ? ? ? ? ? ? ? ? ? ? 0 1 0 0 2 - - - - - - - - - - - - - - - 1 ? ? ? ? ? ? ? ? ? ? ? ? ? ? 0 1 1 1 0 0 1 0 1 ? ? 0 0 - - - - - - - - - ? 0 ? ? ? 1 ? - - - - - ? ? ? ? ? 0 0 0 2 1 0 1 ? - ? ? - - - 0 - ? 1 0 ? 1 1 ? ? 0 2 - 2 ? ? ? ? ? 3 0 0 1 - - 0 1 1 0 0 0 1 0 2 2 ? 1 ? ? 0 0 1 ? ? - - ? 1 0 ? ? 2 0 1 1 ? - - 1 - 1 0 0 ? 0 1 ? ? 0 1 1 ? 2 0 0 ? 1 2 2 - 1 0 0 1 (0 1) 0 ? 0 ? 0 ? ? 0 2 2 0 3 1 ? ? 0 0 0 ? ? ? ? 1 0 0 ? ? 1 0 ? 0 2 1 0 ? 0 1 ? ? ? 1 1 1 1 ? 1 ? 1 1 1 1 ? ? 0 0

***Apalone spinifera***- - - 2 0 0 1 0 0 0 1 2 1 1 2 1 2 0 1 1 2 - 1 1 0 0 - 1 2 0 1 0 1 1 0 1 0 0 - 0 1 1 0 0 2 1 2 - 0 1 - 0 2 - - - - - - - - - - - - - - - 1 0 4 - - - - 1 0 1 0 1 - 1 2 - 1 1 1 1 0 1 - 1 2 1 0 0 - - - - - - - - - 0 0 1 - 1 1 0 - - - - - 1 1 0 1 1 0 0 0 - 1 0 - 1 - 0 0 - - - - - 0 1 1 - 1 1 0 1 0 1 - - 1 0 0 1 - 3 - 0 1 - - 0 1 1 0 0 - 1 0 2 2 1 1 1 1 0 0 1 1 2 - - 2 1 0 0 2 3 - 1 1 0 - - 1 - 1 1 0 0 0 1 0 1 0 1 1 1 2 1 0 0 1 2 2 - 1 0 0 1 1 2 1 0 1 0 1 1 - 2 2 0 3 1 1 0 0 0 0 1 1 2 0 1 0 0 1 0 1 0 0 - - 1 0 1 0 1 - 0 - 1 1 - - - - - 1 1 1 1 0 0 0 -

***Araripemys barretoi***1 1 0 2 0 0 1 ? ? 0 0 - 1 1 2 3 0 2 1 1 2 0 1 1 0 0 0 1 1 0 0 0 0 1 0 ? 1 1 0 0 1 0 1 0 2 1 2 - 0 1 1 0 2 ? ? ? ? ? ? ? ? ? ? ? ? ? ? ? ? 1 ? - - - - 1 ? ? ? 1 0 1 2 0 1 1 1 1 0 1 1 1 2 2 ? 0 1 - - - - - - - - ? ? 1 - 0 1 0 0 1 1 0 0 1 ? 0 0 1 0 ? 1 1 1 0 1 ? ? ? ? 1 - - 0 0 ? 1 1 - 1 1 0 0 0 0 0 2 ? 0 0 0 - 3 0 0 1 - - 0 1 1 0 1 1 1 0 0 2 ? 1 1 1 0 ? - 1 2 0 0 2 1 0 1 ? 1 ? 1 1 0 - - 0 0 1 1 0 0 1 1 ? ? 0 1 1 0 0 0 0 0 1 2 2 - 0 1 0 1 0 0 0 0 - 0 ? ? 0 3 2 1 ? 0 - 0 0 0 0 1 ? 2 ? 1 0 0 0 0 ? 0 1 0 2 ? ? ? 1 1 - 0 - 1 1 1 1 1 1 0 ? 1 1 ? ? 0 1 0

***Australochelys africanus***? ? ? 0 - ? ? ? 0 0 ? ? ? ? 0 ? 0 ? 1 0 - ? ? ? ? ? ? ? ? ? ? ? ? ? ? ? ? ? ? ? ? ? ? ? ? ? ? ? ? ? ? ? ? ? ? ? ? ? ? ? ? ? ? ? ? ? ? ? ? ? ? ? ? ? ? ? ? ? ? ? ? ? ? ? ? ? ? ? ? ? ? ? ? ? 0 ? ? ? ? ? ? ? ? ? ? ? ? - - 0 ? ? ? ? ? ? ? ? ? ? ? ? ? ? ? ? ? ? ? ? ? ? ? ? ? ? ? ? ? ? 0 ? 0 ? ? ? ? ? ? ? ? 0 0 1 - ? ? ? ? ? ? ? ? ? ? ? ? 1 1 1 0 ? 0 ? ? 1 - - ? 0 ? ? ? ? ? ? ? ? ? ? ? ? ? ? ? ? 0 ? ? ? ? ? ? ? ? 1 0 ? ? 0 0 ? ? 1 0 - 0 0 0 0 0 ? 0 0 ? - ? ? ? 1 1 0 0 0 - 0 0 ? 0 0 ? ? ? 0 ? 0 0 0 ? ? ? ? ? 0 ? - ? ? ? ? ? 1 1 ? ? ? ? ? ? 0 ? ? 0 1 ? ?

***Baena arenosa***0 1 1 2 0 1 1 0 0 0 1 ? 1 1 2 2 0 0 1 1 1 0 1 1 1 1 2 0 - - - - - ? 0 ? 1 - - - 0 0 0 ? ? 0 ? - 0 0 0 0 2 ? ? ? ? ? ? ? ? ? ? ? ? ? ? ? 0 0 ? - - - - 0 0 1 ? ? 0 ? ? 0 1 1 1 0 0 0 0 ? 2 2 0 0 0 2 1 0 1 1 ? (0 1) ? ? 1 1 - ? 1 0 0 1 1 0 0 0 0 0 0 1 0 0 1 1 1 1 ? 1 - 0 0 0 0 - 0 0 1 1 0 0 1 ? ? ? ? ? 0 0 0 0 0 1 - 2 0 ? ? ? ? 0 1 1 0 1 0 1 0 2 2 0 0 1 1 0 0 0 ? 0 0 0 2 1 0 0 1 1 ? ? ? ? - - 0 0 0 0 0 0 0 0 0 0 2 1 1 0 0 0 0 ? 1 2 2 - 1 0 0 0 0 2 0 0 2 0 0 0 0 2 2 0 1 1 0 0 0 0 0 1 ? 2 ? 0 0 0 0 0 1 0 ? 0 2 0 0 - 0 1 - 0 - 1 1 1 1 1 1 0 1 0 1 0 0 0 0 0

***Baptemys wyomingensis***0 1 0 2 ? ? 1 ? 0 0 1 ? 1 1 2 1 0 1 1 0 - 0 1 1 ? ? 1 1 1 ? ? ? ? 1 ? 1 ? 1 ? ? ? ? ? ? ? 1 ? - 0 0 0 0 2 - - - - - - - - - - - - - - - 1 0 ? - - - - 1 ? 1 ? ? 1 ? ? 0 1 1 1 0 0 0 0 1 2 1 0 0 1 - - - - - - - - ? 0 ? ? 0 1 0 0 1 1 0 1 ? ? ? ? ? 0 0 1 1 1 1 ? 1 - 1 ? 0 0 - 0 0 ? 1 0 ? 1 1 ? ? 0 0 0 2 0 0 0 2 ? 3 0 ? 1 - - 0 1 1 0 0 1 1 0 2 2 1 1 1 1 0 0 0 ? 2 1 0 2 1 0 0 1 1 0 1 1 ? - - 0 0 0 0 0 ? 0 1 0 0 0 1 1 0 0 0 0 0 1 2 2 - 1 0 0 0 (0 1) ? 0 0 2 ? ? ? 0 2 2 0 1 1 0 0 0 0 0 1 ? 2 ? 1 0 0 0 0 - 2 - 0 2 1 0 0 ? 1 ? ? ? 1 1 1 1 1 1 0 1 0 1 0 0 0 0 0

***Basilemys variolosa***0 1 0 ? ? ? ? ? ? ? ? ? ? ? ? ? 0 0 1 1 2 0 ? ? ? ? 1 1 ? ? ? ? ? 1 ? 1 ? 0 - 0 ? ? ? ? ? ? ? ? 0 (0 1) 0 0 ? ? ? ? ? ? ? ? ? ? ? ? ? ? ? ? ? 0 ? - - - - ? ? ? ? ? ? ? ? 0 1 1 1 0 0 0 0 1 2 ? ? ? 0 (0 2) 1 0 1 ? ? 1 ? ? ? ? ? ? 1 ? 0 1 2 0 1 ? ? ? ? ? 0 ? 0 ? 1 0 ? 1 - 0 0 0 1 0 0 0 ? 1 0 ? 1 0 0 0 0 0 1 ? ? ? ? ? ? 3 0 ? 1 - - 1 1 1 0 1 1 1 0 ? ? ? 1 ? ? 0 0 ? ? ? 0 0 ? 1 0 0 1 1 ? ? ? 0 - - 0 1 0 0 0 ? 0 1 ? ? 0 1 1 0 ? 0 0 ? 1 2 2 - ? 0 ? ? ? ? ? ? ? ? 0 1 0 ? 2 0 ? 1 ? ? 0 0 0 ? ? ? ? 1 0 0 ? 0 ? ? ? 0 2 ? ? ? 1 1 ? ? ? 1 1 1 1 1 ? 0 ? ? 1 ? ? ? 0 0

***Boremys pulchra***0 1 ? 2 0 ? 1 0 0 0 1 ? 1 1 2 2 0 0 1 1 1 0 1 1 1 0 2 0 - - - - - 0 0 0 1 - - - ? ? ? ? 2 0 ? ? 0 0 0 0 2 - - - - - - - - - - - - - - - 1 0 ? - - - - 0 0 1 ? 1 0 ? ? 0 1 1 1 0 0 0 0 ? 2 2 0 0 0 2 ? 0 1 1 1 1 0 ? 1 ? ? ? 1 0 0 1 1 0 0 ? ? ? ? ? 0 0 1 ? ? 1 ? ? - ? ? 0 0 - 0 0 ? 1 0 0 1 ? ? ? ? ? 0 1 ? 1 ? ? ? 1 0 ? 0 0 1 0 1 1 0 0 0 1 0 2 2 0 1 1 1 0 0 0 ? 1 0 0 ? 1 0 0 ? 1 ? ? ? ? - - 0 0 0 0 0 1 0 0 0 0 2 1 1 0 0 0 0 0 1 2 2 - 1 0 0 0 1 ? 0 0 ? 0 ? ? 0 2 2 0 1 1 0 0 0 0 0 1 ? ? ? 0 0 0 0 0 1 0 0 0 2 0 0 0 2 1 - 0 - 1 1 1 1 1 1 0 1 0 1 ? 0 ? ? 0

***Thaichelys ruchae***? 0 ? ? ? ? ? ? ? ? ? ? ? ? ? ? 0 0 1 0 - ? ? ? ? ? ? ? ? ? ? ? ? ? ? ? ? ? ? ? ? ? ? ? ? ? ? ? ? ? 0 ? ? ? ? ? ? ? ? ? ? ? ? ? ? ? ? ? ? ? ? 1 0 1 0 ? ? ? ? 0 ? ? ? 0 ? ? ? ? ? ? ? ? {0 1} ? ? ? 0 0 ? 0 0 (0 1) 1 0 (0 1) ? ? ? ? ? ? ? 0 1 0 ? ? ? ? ? ? ? ? ? ? ? ? ? ? 1 ? ? ? ? ? ? ? ? ? ? ? ? ? ? ? ? ? ? ? ? ? ? ? ? ? ? ? ? ? ? ? ? ? ? ? ? ? ? ? ? ? ? ? ? ? ? ? ? ? ? ? ? ? ? ? ? ? ? ? ? ? ? ? ? 0 ? ? ? ? ? ? ? ? ? ? ? ? ? ? ? ? ? ? ? ? ? ? ? ? ? ? ? ? ? ? ? ? ? ? ? ? ? ? ? ? ? ? ? ? ? ? ? ? ? ? ? ? ? ? ? ? ? {0 1} ? ? ? ? ? ? ? ? ? ? ? ? ? ? ? ? ? ? ? ? ? ? ?

***Caretta caretta***0 1 0 1 0 1 1 1 0 1 1 ? 1 1 ? 1 0 0 1 0 - 1 1 1 0 0 1 1 1 0 0 1 1 1 1 1 0 1 2 0 1 0 0 0 1 1 2 - 0 0 1 0 2 - - - - - - - - - - - - - - - 0 0 3 - - - - 1 0 1 0 1 1 0 2 0 1 1 1 0 0 1 1 1 2 1 0 0 1 - - - - - - - - 1 0 1 - 0 (0 1) 0 0 1 2 0 ? 1 1 1 - 1 0 0 0 2 1 0 ? 1 - 0 0 0 ? ? 0 1 0 1 0 0 1 1 1 0 2 0 0 1 - 1 1 0 - 3 0 0 1 - - 0 1 2 0 0 1 1 0 2 2 0 0 1 2 0 0 0 1 0 0 0 2 1 0 0 2 0 0 1 1 0 - - 0 0 1 1 0 0 0 1 0 0 0 1 1 0 1 0 0 0 1 2 2 - 1 0 2 0 - 0 1 0 1 0 1 1 0 2 2 0 1 1 1 0 0 0 0 1 1 2 0 0 0 0 0 1 1 0 0 0 2 1 0 0 2 1 - 0 - 1 1 1 1 1 1 ? 1 0 1 1 0 0 0 1

***Carettochelys insculpta***- ? - 2 0 0 1 0 0 0 1 ? 1 1 2 1 1 0 1 1 2 0 1 1 0 0 - 1 1 0 1 0 1 1 0 1 0 0 - 0 1 1 0 0 2 1 ? - 0 1 0 0 2 - - - - - - - - - - - - - - - 1 0 4 - - - - 1 0 1 0 1 1 0 2 0 1 1 1 0 0 1 0 1 2 1 0 0 - - - - - - - - - ? 0 1 - 1 1 0 - - - - - 0 0 1 - 1 0 0 0 2 1 0 1 1 - 0 1 - - - 0 - 0 1 0 0 1 1 0 0 0 2 - - 0 0 0 0 - 3 0 0 1 - - 0 1 1 0 0 0 1 0 2 2 1 1 1 1 0 0 1 1 2 - - 2 1 0 0 2 2 0 1 1 0 - - 1 - 1 0 0 0 0 1 0 1 0 1 1 1 2 0 0 0 1 2 2 - 1 0 0 1 1 0 1 0 1 0 1 1 0 2 2 0 3 1 1 0 0 1 0 1 ? 2 ? 1 0 0 1 0 1 0 0 - 2 1 0 1 1 1 - 0 - 1 1 - - - - - 1 1 1 1 0 0 0 0

***Chelodina* (*Macrochelodina*) *oblonga***0 1 0 2 0 0 1 0 0 0 0 ? 1 1 2 3 0 0 1 1 0 0 1 1 0 0 1 1 1 0 0 0 0 1 0 0 1 1 3 1 1 0 1 0 ? 1 ? - 1 2 0 0 2 - - - - - - - - - - - - - - - 1 1 ? - - - - 1 0 1 0 ? 0 0 2 1 1 1 1 0 0 0 0 1 2 2 1 1 0 1 0 0 1 1 ? 2 ? ? 0 1 - 2 1 1 1 - - 0 0 1 1 0 0 1 0 1 1 1 1 0 1 1 1 0 0 1 - - 0 0 0 1 0 - 1 1 0 ? 0 0 0 2 1 1 0 0 - 3 0 ? 0 1 1 0 0 1 0 1 0 1 0 0 2 0 1 1 1 0 0 - 1 2 0 0 2 1 0 1 2 1 0 1 1 0 - - 0 0 0 0 0 0 1 0 1 1 1 1 1 0 0 0 0 0 1 2 2 - 0 1 0 0 0 0 0 0 - 0 0 1 0 3 2 0 2 0 - 1 1 - - 1 ? 2 ? 1 0 0 0 0 1 0 1 0 2 0 0 0 1 1 - 0 - 1 1 1 1 - 1 0 1 0 1 ? 0 0 1 0

***Chelodina longicollis***0 1 0 2 0 0 1 0 0 0 0 ? 1 1 2 3 0 0 1 1 0 0 1 1 0 0 1 1 1 0 0 0 0 1 0 0 1 1 3 1 1 0 1 0 2 1 2 - 1 2 0 0 2 - - - - - - - - - - - - - - - 1 1 2 - - - - 1 0 1 0 1 0 0 2 1 1 1 1 0 0 0 0 1 2 2 1 1 0 1 0 0 1 1 1 1 0 1 0 1 - 2 1 1 1 - - 0 0 1 1 0 0 1 0 1 1 1 1 0 1 1 1 0 0 1 - - 0 0 0 1 0 - 1 1 0 0 0 0 0 2 1 1 0 0 - 3 0 1 0 1 1 0 - 1 0 1 0 1 0 0 2 0 1 1 1 0 0 - 1 2 0 0 2 1 0 1 2 1 0 1 1 0 - - 0 0 0 0 0 0 1 0 1 1 1 1 1 0 0 0 0 0 1 2 2 - 0 1 0 0 0 0 0 0 - 0 0 1 0 3 2 0 2 0 - 1 1 - - 1 1 2 0 1 0 0 0 0 1 0 1 0 2 0 0 0 1 1 - 0 - 1 1 1 1 - 1 0 1 0 1 1 0 0 1 0

***Chelonia mydas***0 1 0 1 0 1 1 1 0 1 1 ? 1 1 2 1 0 0 1 0 - 1 1 1 0 0 1 1 1 0 0 1 1 1 1 1 0 1 2 0 1 0 0 1 1 1 2 - 0 0 1 0 2 - - - - - - - - - - - - - - - 0 0 3 - - - - 1 0 1 0 1 1 0 2 0 1 1 1 0 0 1 1 1 2 1 0 0 1 - - - - - - - - 1 0 1 - 0 1 0 0 1 2 0 ? 1 1 1 - 1 0 0 0 2 1 0 ? 1 - 0 0 0 ? ? 0 1 0 1 0 0 1 1 1 ? 2 0 0 2 - 1 1 2 0 3 0 0 1 - - 0 1 2 0 0 0 1 0 2 2 0 0 1 2 0 0 0 1 0 0 ? 2 1 0 0 2 1 0 1 1 0 - - 0 0 1 1 0 0 0 1 ? 0 0 1 1 0 1 0 1 0 1 2 2 - 1 0 2 0 - 0 1 0 1 0 1 1 0 2 2 0 1 1 0 0 0 0 0 1 1 2 0 0 0 0 0 1 1 0 0 0 2 1 0 0 2 1 - 0 - 1 1 1 1 1 2 0 1 0 1 1 0 0 0 1

***Chelonoidis chilensis***0 1 0 2 0 0 1 0 0 0 1 0 1 1 2 1 0 0 1 0 - 0 1 1 0 0 0 1 1 0 1 0 1 1 0 1 0 1 2 1 1 1 0 1 2 1 2 - 0 0 0 1 2 - - - - - - - - - - - - - - - 1 0 1 - - - - 1 0 1 0 1 1 0 2 0 1 1 1 0 0 0 0 0 2 1 0 0 1 - - - - - - - - ? 0 0 1 0 1 0 0 2 1 0 0 (0 1) 0 0 1 1 0 0 0 0 1 1 ? 1 - 0 0 0 1 0 0 0 0 1 0 - 1 0 0 0 0 0 0 2 0 0 0 2 0 3 0 0 1 - - 0 0 1 0 1 0 1 0 2 2 0 1 1 1 0 0 1 1 2 0 1 2 1 0 0 1 1 0 1 1 1 - - 0 0 0 0 0 0 0 1 0 0 0 1 1 0 0 0 0 0 1 2 2 - 1 0 0 0 1 0 0 0 2 0 0 1 0 2 2 0 3 1 0 0 0 0 0 1 1 2 0 1 0 0 0 0 1 0 0 0 2 1 0 0 2 1 - 0 - 1 1 1 1 0 1 0 1 0 1 0 1 1 1 0

***Chelonoidis gringorum***0 1 0 ? ? ? ? ? ? ? ? ? ? ? ? ? 0 0 1 0 - 0 ? ? ? ? 0 ? ? ? ? ? ? ? ? ? ? ? ? ? ? ? ? ? ? ? ? ? 0 0 0 1 2 ? ? ? ? ? ? ? ? ? ? ? ? ? ? ? ? ? ? ? ? ? ? ? ? ? ? ? ? ? ? 0 1 1 1 0 0 0 0 0 ? ? ? ? 1 - - - - - - - - ? ? ? ? ? ? ? 0 1 (0 1) 0 0 1 ? 0 ? 1 0 ? ? ? ? ? ? ? ? ? ? 0 1 0 0 0 ? ? ? ? ? ? ? ? ? ? 0 2 ? ? ? ? ? 3 0 0 ? ? ? 0 0 ? ? 1 0 ? ? ? ? ? ? ? ? ? ? ? ? ? 0 1 ? ? 0 0 1 1 0 ? ? ? - - 0 0 0 0 0 ? ? ? ? ? ? ? ? ? ? ? ? ? ? ? ? ? ? ? ? ? ? ? ? ? ? ? ? ? 0 ? ? ? ? ? ? ? ? ? ? ? ? ? ? ? ? ? ? ? ? ? ? 0 2 ? ? ? 2 ? ? ? ? ? ? 1 1 1 1 0 ? ? ? ? ? ? 1 0

***Chelus fimbriatus***0 1 0 2 0 0 1 0 0 0 0 - 1 1 2 3 0 2 1 0 - 0 1 1 0 0 1 1 1 0 0 0 0 1 1 1 1 1 3 1 1 0 1 0 2 1 2 - 0 0 1 0 2 - - - - - - - - - - - - - - - 1 1 0 - - - - 1 0 1 0 1 0 1 1 0 1 1 1 0 0 0 0 1 2 2 1 1 0 2 1 0 1 1 1 1 0 0 0 1 - 2 1 0 1 1 1 0 1 1 0 0 0 1 0 1 1 1 1 1 1 1 1 0 0 1 - - 0 0 0 1 0 - 1 1 0 0 0 0 0 1 0 1 0 0 - 3 0 1 1 - - 0 0 1 0 1 0 1 0 0 2 0 0 1 1 0 0 - 1 2 0 0 2 1 0 1 2 1 0 1 1 0 - - 0 0 0 0 0 0 1 0 1 0 1 1 1 1 0 0 0 0 1 2 2 - 0 1 0 0 0 0 0 0 - 0 0 1 0 3 2 0 2 0 - 0 1 - - 1 1 2 0 1 0 0 0 0 1 0 1 0 2 0 0 0 1 1 - 0 - 1 1 1 1 1 1 0 1 0 1 0 0 0 1 1

***Chelydra serpentina***1 1 1 2 0 0 1 0 0 0 1 0 1 1 2 1 0 0 1 0 - 0 1 1 0 1 1 1 1 0 1 0 1 1 1 1 0 1 2 0 1 1 0 1 2 0 2 - 0 0 0 0 2 ? ? ? ? ? ? ? ? ? ? ? ? ? ? ? 0 0 1 - - - - 1 0 1 0 1 1 1 1 0 1 1 1 0 0 1 1 ? 2 1 0 0 1 - - - - - - - - 1 0 0 ? ? 0 0 0 1 2 0 1 1 0 0 0 1 0 0 0 2 1 0 1 1 - 0 0 0 0 - 0 0 ? 1 0 0 1 1 ? ? 0 0 0 1 0 1 0 0 - 3 0 0 1 - - 0 0 1 2 0 0 1 0 2 2 0 1 1 1 0 0 0 ? 2 0 ? 2 1 0 0 1 1 0 1 1 ? - - 0 0 1 1 0 0 0 1 0 0 0 1 1 0 0 0 1 0 1 2 2 - 1 0 0 0 1 2 1 0 2 0 0 1 0 2 2 0 3 1 0 0 0 0 0 1 1 2 0 0 0 0 0 0 1 0 0 0 2 1 0 0 3 1 - 0 - 1 1 1 1 1 1 0 1 0 1 0 0 0 0 0

***Chinlechelys tenertesta***? ? ? ? ? ? ? ? ? ? ? ? ? ? ? ? 0 0 1 0 - ? ? ? ? ? ? ? ? ? ? ? ? ? ? ? ? ? ? ? ? ? ? ? ? ? ? ? ? ? ? ? ? ? ? ? ? ? ? ? ? ? ? ? ? ? ? ? ? ? ? ? ? ? ? ? ? ? ? ? ? 0 ? ? ? ? ? ? ? ? ? ? ? ? ? ? ? ? ? ? ? ? ? ? ? 0 ? ? ? ? ? ? ? ? ? ? ? ? ? ? ? ? ? ? ? ? ? ? ? 1 ? 0 ? ? ? ? ? ? ? ? ? ? ? ? ? ? ? ? ? 1 ? ? ? ? ? ? 1 ? ? ? ? ? ? ? ? ? ? ? ? ? ? ? ? ? ? ? ? ? ? ? ? ? ? ? ? ? ? ? ? ? ? ? ? ? ? ? ? ? ? ? ? ? ? ? ? ? ? ? ? ? ? ? ? ? ? ? ? ? ? ? ? ? ? ? ? ? ? ? ? ? ? ? ? ? ? ? ? ? ? ? ? ? ? ? ? ? ? ? ? ? ? ? ? ? ? ? ? ? ? ? ? ? ? ? ? ? 0 ? ? ? ? ? ? ? ? ?

***Chisternon undatum***0 1 1 2 0 1 1 0 0 0 1 ? 1 1 2 2 0 0 1 ? ? 0 1 1 ? ? 2 1 1 0 0 0 0 ? 0 0 ? 1 2 ? ? ? 0 ? ? 0 ? ? 0 0 0 0 2 - - - - - - - - - - - - - - - 1 0 ? ? ? ? ? 0 0 1 ? ? 0 ? ? 0 1 1 1 0 0 0 0 ? ? 2 0 0 0 2 1 0 1 1 ? 1 ? ? 1 1 - 0 1 0 0 1 (0 1) 0 0 ? 0 0 0 1 0 0 1 ? 1 1 ? 1 - 0 0 0 ? ? 0 0 1 1 0 0 1 ? ? ? ? ? ? 0 0 1 0 1 - 1 0 ? 0 0 1 0 1 1 0 0 0 1 0 2 2 0 0 1 1 0 0 ? ? 1 0 0 ? 1 0 0 1 1 ? ? ? ? - - 0 0 0 0 0 0 0 0 0 0 2 1 1 0 0 0 0 ? 1 2 2 - 1 0 0 0 1 2 0 0 2 0 0 0 0 2 2 0 {1 2} 1 0 0 0 0 0 1 ? 2 ? 0 0 0 0 0 1 0 0 ? 2 0 0 0 ? 1 ? 0 - 1 1 1 1 - 1 ? 1 0 1 0 0 0 0 0

***Chrysemys picta***0 1 0 2 0 0 1 0 0 0 1 0 1 1 2 1 0 0 1 0 - 0 1 1 0 0 1 1 1 1 1 0 1 1 1 1 0 1 2 1 1 1 0 1 2 1 2 - 0 0 0 0 2 - - - - - - - - - - - - - - - 1 0 0 - - - - 1 0 1 0 1 1 0 2 0 1 1 1 0 0 0 0 1 2 1 0 0 1 - - - - - - - - 0 0 0 1 0 1 0 0 2 1 0 0 1 0 0 1 1 0 0 1 1 1 1 0 1 - 0 0 0 1 0 0 0 1 1 0 - 1 1 0 0 0 0 0 2 0 0 0 2 0 3 0 0 1 - - 0 1 1 0 1 0 1 0 2 2 0 1 1 1 0 0 1 1 2 0 1 2 1 0 0 1 1 0 1 1 0 - - 0 0 0 0 0 0 0 1 0 0 0 1 1 0 0 0 0 1 1 2 2 - 0 0 0 0 1 2 0 0 2 0 0 1 0 2 2 0 1 1 0 0 0 0 0 1 1 2 0 1 0 0 0 0 1 0 0 0 2 1 0 0 1 1 - 0 - 1 1 1 1 1 1 0 1 0 1 0 0 0 1 0

***Chubutemys copelloi***? ? ? 2 ? 0 0 0 0 0 1 ? 0 0 1 0 0 0 1 0 - 0 ? ? ? ? {0 1} 1 1 ? ? ? 0 ? ? ? ? ? ? ? ? ? 0 ? 2 ? ? - 0 ? 0 0 ? ? ? ? ? ? ? ? ? ? ? ? ? ? ? ? ? ? ? ? ? ? ? ? ? ? ? ? 0 0 ? ? ? 0 ? 0 0 ? 0 ? ? {0 1} 0 ? ? ? ? ? ? ? ? ? ? ? ? 1 - 0 0 0 ? ? ? ? ? 0 ? ? ? ? 0 ? ? 1 ? ? 1 ? ? ? ? ? ? ? ? ? ? 1 0 ? 1 ? ? ? ? ? ? 2 ? ? 0 ? - ? 0 1 ? ? ? ? 1 ? ? 0 0 1 0 1 2 ? 0 1 ? 0 0 ? ? 0 ? ? 2 1 0 ? ? 1 0 ? ? ? - - 0 ? 1 ? 0 0 ? ? 0 0 0 1 1 ? ? ? ? ? 1 1 2 - 0 0 0 0 ? 0 0 0 0 ? ? ? ? 1 2 0 1 1 ? 0 0 0 ? 1 ? ? ? 0 0 ? 0 0 ? ? ? ? ? 1 0 0 ? 1 ? ? ? 1 1 1 0 ? 1 ? 1 1 1 0 1 ? ? ?

***Condorchelys antiqua***? ? ? ? - 1 0 ? 1 0 0 ? 0 0 0 0 0 0 1 0 - 0 1 0 - - ? 0 ? - - - - 0 0 ? 0 - - - 0 ? 0 0 ? 0 2 - 0 0 0 0 1 ? ? ? ? ? ? ? ? ? ? ? ? ? ? ? ? ? ? ? ? ? ? ? 0 ? ? ? ? 0 2 0 0 0 1 0 0 0 ? ? ? ? 0 ? ? ? ? ? ? ? ? ? ? 1 ? - - 0 ? 0 ? ? ? 0 ? 0 0 0 0 1 ? ? ? ? 1 0 0 ? ? ? 0 0 0 - ? ? 1 ? ? ? ? ? 0 ? 0 0 0 2 ? ? ? ? ? 1 0 1 ? ? ? 0 0 ? ? ? ? 1 0 1 1 ? ? ? ? 0 ? - ? 0 0 0 2 1 0 0 1 ? 0 ? ? ? - - 0 ? 1 1 0 ? ? ? ? ? ? ? ? ? ? ? ? ? 1 1 0 - 0 0 ? 0 ? 0 0 0 (0 2) 0 0 0 0 1 2 0 0 0 - 0 ? ? ? 0 ? ? ? ? ? 0 0 0 ? ? ? ? 2 ? ? - 2 ? - 0 - 1 1 1 0 0 1 0 ? ? ? ? ? ? ? 0

***Dermatemys mawii***0 1 0 2 0 1 1 0 0 0 1 ? 1 1 2 1 0 0 1 0 - 0 1 1 ? ? 1 1 1 0 1 0 1 1 1 1 0 0 - 0 1 1 0 1 2 1 2 - 0 1 0 0 2 - - - - - - - - - - - - - - - 1 0 3 - - - - 1 0 1 0 1 1 0 2 0 1 1 1 0 0 0 0 ? 2 1 0 0 1 - - - - - - - - 1 0 0 1 0 (0 1) 0 0 1 2 0 0 1 0 0 1 1 0 0 1 1 1 0 0 1 - 0 0 0 0 0 0 1 ? 1 0 0 1 1 0 0 0 0 0 2 0 0 0 2 0 3 0 1 1 - - 0 1 1 (0 1) 0 0 1 0 2 2 1 1 1 1 0 0 0 ? 2 1 0 2 1 0 0 1 1 0 1 1 0 - - 0 0 0 0 0 0 0 1 0 0 0 1 1 0 0 0 0 0 1 2 2 - 1 0 0 0 1 2 0 0 2 0 1 0 0 2 2 0 1 1 0 0 0 0 0 1 1 2 0 1 0 0 0 0 - 2 - 0 2 1 0 0 1 1 - 0 - 1 1 1 1 1 1 0 1 0 1 0 0 0 1 0

***Dermochelys coriacea***- - - 1 - 0 1 1 0 0 1 ? 1 1 2 2 2 0 1 - ? - 1 1 0 0 - 1 1 0 0 1 1 1 1 1 0 1 2 0 0 0 0 0 2 1 2 - 0 - 1 - 1 - - - - - - - - - - - - - - - 1 0 ? - - - - 1 0 - - 1 0 - - - 1 1 1 0 0 1 1 ? 2 2 0 0 - - - - - - - - - ? 0 1 - 0 0 0 - - - - - 1 1 1 - 1 ? 0 0 ? 1 0 ? 1 - 0 0 - - - - - 0 0 0 1 1 1 1 ? 2 0 - - 0 0 0 1 - 3 - 0 1 - - 0 - 2 0 - - 1 0 2 2 0 0 0 - 0 0 - 1 0 - - 2 1 0 0 1 3 - 1 1 ? ? ? 1 - 1 1 - 0 0 1 0 1 0 1 1 0 0 0 0 0 1 2 2 - 1 0 2 0 - 0 0 0 1 0 1 1 - 2 2 0 1 0 - 0 0 0 0 1 1 2 0 0 0 0 0 1 1 0 0 - 2 1 0 0 - 1 - 0 - 1 1 - - - - - 1 0 1 0 0 0 0 1

***Dinochelys whitei***0 1 (0 1) ? ? ? ? 0 ? ? ? ? ? ? ? ? 0 0 1 1 1 0 1 ? ? ? 1 0 - - - - - 0 ? ? ? - - - ? ? ? ? ? ? ? ? 0 0 0 0 2 ? ? ? ? ? ? ? ? ? ? ? ? ? ? ? ? 0 ? ? ? ? ? 0 ? 1 ? ? ? ? ? 0 1 1 1 0 0 0 0 ? 1 ? 0 ? 0 0 0 0 1 1 ? 0 ? ? ? ? ? ? 1 0 0 1 0 0 0 ? ? ? ? ? 0 ? ? 0 ? ? 0 ? ? ? ? 1 - - 0 0 ? 1 0 ? 1 1 ? ? 0 0 1 2 ? ? ? ? ? 1 0 ? 0 ? 1 0 1 ? ? 0 0 ? 0 ? ? ? ? ? ? 0 0 ? ? 0 0 0 ? 1 0 0 ? 1 ? 1 1 ? - - 0 0 0 0 0 ? ? 0 ? ? 1 1 1 0 ? 0 1 ? ? ? 2 - ? ? ? ? ? ? ? 0 ? 0 ? ? 0 ? 2 0 ? 1 ? ? 0 0 0 ? ? ? ? 0 ? 0 0 ? 1 0 ? 0 2 ? ? ? 2 ? ? ? ? 1 1 1 0 1 1 1 ? ? ? ? ? ? 0 0

***Dorsetochelys delairi***? ? ? 2 0 0 0 ? 1 0 0 ? 1 1 2 0 ? ? ? ? ? ? ? ? ? ? ? ? ? ? ? ? ? ? ? ? ? ? ? ? ? ? ? ? ? ? ? ? ? ? ? ? ? - - - - - - - - - - - - - - - 1 ? ? ? ? ? ? ? ? ? ? ? ? ? ? ? ? ? ? ? ? ? ? ? ? 1 0 0 ? ? ? ? ? ? ? ? ? ? ? 1 - 0 1 0 ? ? ? ? ? ? ? ? ? ? ? ? ? ? ? ? ? ? ? ? ? ? ? ? ? ? ? 1 0 ? 1 ? ? ? ? ? ? ? 0 1 0 0 - ? ? ? 0 0 1 ? ? ? ? ? ? 1 0 1 2 ? 0 1 ? 0 ? ? ? 0 ? ? ? ? ? ? ? ? ? ? ? ? ? ? ? ? ? ? ? 0 ? 0 0 0 1 1 1 0 ? 0 0 ? 1 2 2 - 1 0 0 0 0 2 0 0 2 0 ? ? ? 2 2 0 {1 2} 1 ? 0 0 0 0 1 ? ? ? 0 0 0 0 0 1 0 ? ? ? 0 0 - ? 1 ? ? ? 1 1 ? ? ? ? ? 1 0 1 0 0 ? ? ?

***Dracochelys bicuspis***? ? ? ? ? 0 1 ? 1 0 1 ? 0 1 1 1 0 0 1 ? ? 0 ? ? ? ? 0 1 1 0 0 0 0 0 1 1 ? 0 - 0 1 0 ? 0 2 ? 2 - 0 0 0 0 2 - - - - - - - - - - - - - - - 1 0 ? ? ? ? ? 0 0 1 ? 1 0 0 2 ? ? ? ? 0 0 1 1 ? ? 1 0 0 ? ? ? ? ? ? ? ? ? 1 ? ? ? 0 1 0 ? ? ? ? ? 0 ? 0 0 1 0 0 0 0 ? 0 0 ? ? ? ? ? ? ? 0 ? ? ? 0 ? 1 ? ? ? 0 0 ? 2 0 0 0 1 - 3 0 ? ? ? ? 0 1 1 0 0 1 1 0 2 2 ? ? 1 1 0 0 ? 1 ? ? ? 2 1 0 0 ? 1 ? 0 ? ? - - 0 ? 1 1 0 0 ? ? 0 ? ? ? 1 0 0 0 0 ? 1 1 2 - 1 0 0 0 0 2 1 0 2 0 ? ? 0 2 2 0 1 1 0 0 0 0 0 1 1 2 0 ? 0 0 0 ? 1 0 0 0 ? ? 0 ? 2 1 - 0 - 1 1 1 1 1 1 0 1 0 1 0 0 0 0 0

***Echmatemys wyomingensis***0 1 0 ? ? ? ? ? ? ? ? ? ? ? ? ? 0 0 1 0 - 0 ? ? ? ? 1 ? ? ? ? ? ? ? ? ? ? ? ? ? ? ? ? ? ? ? ? - 0 0 0 0 2 ? ? ? ? ? ? ? ? ? ? ? ? ? ? ? ? ? ? - - - - 1 0 1 ? ? ? 0 2 0 1 1 1 0 0 0 0 1 2 ? ? ? 1 - - - - - - - - ? ? ? ? ? ? ? 0 (1 2) 1 0 1 ? ? ? ? ? 0 ? 1 0 ? 1 1 1 - ? 0 0 1 0 0 0 ? ? ? ? ? ? ? ? ? ? 0 2 ? ? ? ? ? 3 0 ? ? ? ? 0 1 1 0 1 0 ? ? ? ? ? ? ? ? ? ? ? ? ? 0 0 2 1 0 0 ? 1 0 ? ? ? - - 0 0 0 0 0 ? ? ? ? ? ? ? ? ? ? ? ? ? ? ? ? ? ? ? ? ? ? ? ? ? ? ? ? ? 0 ? ? ? ? ? ? ? ? ? ? ? ? ? ? ? ? ? ? ? ? ? ? 0 2 ? ? ? ? ? ? ? ? ? ? 1 1 1 1 (0 1) ? ? ? ? ? ? 1 0

***Eileanchelys waldmani***? ? ? 2 ? 0 0 ? 1 0 0 - ? ? ? ? 0 ? 1 0 - ? ? ? ? ? 1 ? ? ? ? ? ? ? ? ? ? ? ? ? ? ? ? ? ? ? ? ? 0 0 0 0 2 ? ? ? ? ? ? ? ? ? ? ? ? ? ? ? ? 0 ? ? 0 1 0 ? ? ? ? ? ? ? ? 0 1 ? ? 0 0 0 0 ? 1 ? ? ? 0 ? ? 0 1 1 ? 0 ? ? ? 1 - 0 1 0 0 1 0 0 ? ? ? ? ? ? 0 ? ? ? ? ? ? ? ? ? ? 0 ? ? ? 0 ? 1 0 0 1 ? ? ? ? ? ? 2 0 ? 0 ? ? 1 0 ? 0 0 0 0 0 ? ? 0 0 1 0 1 2 ? 0 ? ? 0 ? - 1 0 0 ? ? ? 0 ? ? 1 ? ? ? ? - - 0 ? ? ? 0 ? 0 0 0 ? 1 1 1 0 0 0 0 0 1 ? ? - 0 0 ? ? ? ? 0 0 ? 0 ? ? 0 1 2 0 ? 0 - 0 0 0 0 1 ? ? ? 0 ? 0 0 0 ? 0 0 0 2 0 ? - 2 ? ? ? ? 1 1 1 0 0 1 ? 1 ? 1 0 0 0 0 0

***Elseya dentata***0 1 0 2 0 0 1 0 0 0 0 ? 1 1 2 3 0 0 1 ? ? 0 1 1 ? ? 0 1 1 0 0 0 0 1 ? 0 1 1 3 1 1 0 ? 0 2 1 2 - 1 2 0 0 2 - - - - - - - - - - - - - - - 1 0 ? - - - - 1 0 1 0 1 0 0 2 0 1 1 1 0 0 0 0 ? 2 2 1 0 0 0 ? 0 1 1 1 2 0 0 0 1 - ? 1 0 1 1 2 0 ? ? ? 0 ? ? 0 1 1 0 1 1 0 1 1 0 0 1 - - 0 0 ? 1 0 - 1 1 ? ? 0 0 0 1 1 1 0 2 0 3 0 ? 0 1 1 0 - 1 0 ? 0 1 0 0 2 0 0 1 1 0 0 - ? 0 0 0 2 1 0 1 2 1 0 1 1 ? - - 0 0 0 0 0 0 1 0 1 1 1 1 1 0 0 0 0 0 1 2 2 - 0 1 0 0 0 0 0 0 - 0 ? ? 0 3 2 0 2 0 - 0 1 - - 1 1 2 0 1 0 0 0 0 1 0 1 0 2 1 0 - ? 1 - 0 - 1 1 1 1 - 1 0 1 0 1 1 0 0 1 0

***Emarginachelys cretacea***? ? 0 2 0 ? 1 0 0 ? ? ? ? ? ? ? 0 1 1 ? ? 0 ? ? ? ? ? 1 1 ? ? ? ? 1 ? ? ? 1 ? ? ? ? ? ? 2 ? 2 - 0 0 0 ? 2 - - - - - - - - - - - - - - - 1 ? ? ? ? ? ? ? ? 1 ? 1 ? ? ? 0 1 1 1 0 0 ? 0 ? ? 1 0 0 1 - - ? ? ? ? ? ? 1 ? ? ? ? 1 ? 0 1 2 0 ? ? ? ? ? ? ? 0 0 1 1 0 ? 1 - 0 1 0 ? ? 0 0 ? 1 0 0 1 1 ? ? 0 0 0 1 ? ? ? ? ? 3 0 ? 1 - - 0 ? 1 2 ? 0 1 0 2 2 1 1 1 1 0 0 0 ? ? ? ? 2 1 0 0 ? 1 ? 1 1 ? - - 0 0 1 0 0 0 0 1 0 0 0 1 1 0 0 0 0 ? 1 2 2 - 1 0 0 0 1 2 ? ? ? ? ? ? 0 2 2 0 1 1 ? ? 0 0 0 ? ? 2 0 1 0 0 0 ? 1 0 ? 0 2 1 0 ? ? 1 ? ? ? 1 1 1 1 1 ? 0 1 0 1 0 ? ? 0 0

***Emys orbicularis***0 1 0 2 0 0 1 0 0 0 1 0 1 1 2 1 0 0 1 0 - 0 1 1 0 0 1 1 1 0 1 0 1 1 1 1 0 1 2 1 1 1 0 1 2 1 2 - 0 0 0 0 2 - - - - - - - - - - - - - - - 1 0 0 - - - - 1 0 1 0 1 1 0 2 0 1 0 1 0 0 0 0 1 2 1 0 0 1 - - - - - - - - 0 0 0 1 0 0 0 0 1 2 0 1 1 0 0 1 1 0 0 0 1 1 0 1 1 - 0 0 ? ? ? 0 0 ? 1 0 - 1 1 0 0 0 0 0 2 0 ? 0 0 - 3 0 0 1 - - 0 1 1 0 1 0 1 0 2 2 0 1 1 1 0 0 1 1 2 0 0 2 1 0 0 1 1 0 1 1 0 0 0 0 0 1 0 1 0 0 1 0 0 0 1 1 0 0 0 0 1 1 2 2 - 0 0 0 0 1 2 0 0 2 0 ? 1 0 2 2 0 1 1 0 0 0 0 0 1 1 2 0 ? 0 0 0 0 1 0 0 0 2 1 0 0 1 1 ? ? ? 1 1 1 1 1 1 0 1 0 1 0 0 0 0 0

***Erymnochelys madagascariensis***0 1 0 2 0 0 1 0 0 0 0 - 1 1 2 - 0 0 1 0 - 0 1 1 ? ? 0 1 1 0 0 0 0 1 0 0 1 1 0 0 1 0 0 0 2 1 2 - 0 1 0 0 2 - - - - - - - - - - - - - - - 0 0 ? - - - - 1 0 1 0 1 0 0 2 0 1 1 1 0 0 0 0 1 2 2 1 0 0 0 1 0 1 1 1 2 0 1 0 1 - ? 1 0 1 1 2 0 1 ? ? 0 ? ? 0 1 1 0 1 1 0 1 1 0 0 1 - - 0 0 ? 1 0 - 1 1 ? ? 0 0 0 2 0 1 0 2 0 2 0 1 1 - - 0 1 1 0 1 0 1 0 0 2 0 1 1 1 0 0 - ? 0 0 0 2 1 0 1 2 1 0 1 1 ? - - 0 0 0 0 0 0 1 1 1 1 0 1 1 0 0 0 0 ? 1 2 2 - 0 1 0 0 0 0 0 1 - 0 0 1 0 3 2 1 2 0 - 1 0 0 0 1 1 ? 0 1 0 0 0 0 1 0 ? 0 2 1 0 0 1 1 - 0 - 1 1 1 1 1 1 0 2 1 1 1 - 0 1 0

***Eurotestudo hermanni***0 1 0 2 0 0 1 0 0 0 1 0 1 1 2 1 0 0 1 0 - 0 1 1 0 0 1 1 1 0 1 0 1 1 1 1 0 1 2 1 1 1 0 1 2 1 2 - 0 0 0 1 2 - - - - - - - - - - - - - - - 1 0 ? - - - - 1 0 1 0 1 1 0 2 0 1 1 1 0 0 0 0 0 2 1 0 0 1 - - - - - - - - 1 0 0 1 0 1 0 0 1 1 0 0 1 0 0 1 1 0 0 1 0 1 0 0 1 - 0 0 0 1 0 0 0 1 1 0 - 1 0 0 0 0 0 0 0 0 0 0 2 0 3 0 0 1 - - 0 0 1 0 1 0 1 0 2 2 0 1 1 1 0 0 1 ? 2 0 1 2 1 0 0 1 1 0 1 1 1 - - 0 0 0 0 0 0 0 1 0 0 0 1 1 0 0 0 0 1 1 2 2 - 1 0 0 0 1 0 0 0 2 0 0 1 0 2 2 0 3 1 0 0 0 0 0 1 1 2 0 1 0 0 0 0 1 0 0 0 2 1 0 0 2 1 - 0 - 1 1 1 1 1 1 0 1 0 1 0 1 1 1 0

***Gaffneylania auricularis***0 1 ? 0 0 1 0 ? 0 0 0 - 0 1 0 1 0 ? 1 0 - ? ? 1 1 ? 1 1 ? ? ? ? ? 0 0 ? 0 ? ? ? 0 ? ? ? ? ? ? ? ? ? 0 ? ? ? ? ? ? ? ? 0 0 ? ? ? ? ? ? ? 0 0 ? ? ? ? ? ? ? ? ? ? ? ? ? ? ? ? ? ? ? ? ? ? ? {0 1} 1 0 ? ? ? ? ? ? ? ? ? ? ? 1 - 0 ? ? ? ? ? ? ? 0 0 0 0 0 ? ? ? ? ? ? ? ? ? ? ? ? ? ? ? ? ? ? ? ? ? ? ? ? ? ? ? ? ? ? ? ? ? ? 0 ? ? ? ? ? ? ? ? ? ? 1 0 1 2 ? ? ? ? ? ? ? ? ? ? ? ? ? ? 0 ? ? ? ? ? ? ? ? 0 ? 1 ? ? ? ? ? ? ? ? ? 1 0 ? 0 0 0 ? 2 1 0 1 0 ? ? ? ? 0 ? ? 1 ? ? ? 2 2 0 1 1 0 0 0 ? 1 1 ? ? ? ? ? 2 0 0 1 0 0 ? 2 ? ? ? ? ? ? ? ? 1 1 ? ? ? ? ? ? ? ? ? ? ? ? ?

***Geoclemys hamiltonii***0 1 0 2 0 0 1 0 0 0 1 0 1 1 2 1 0 1 1 0 - 0 1 1 0 0 1 1 1 0 1 0 1 1 1 1 0 1 2 1 1 1 0 1 2 1 2 - 0 0 0 0 2 - - - - - - - - - - - - - - - 1 0 ? - - - - 1 0 1 0 1 1 0 2 0 1 1 1 0 0 0 0 0 2 1 0 0 1 - - - - - - - - 1 0 0 1 0 0 0 0 2 1 0 1 1 0 0 1 1 0 0 1 1 1 1 1 1 - 0 0 0 1 0 0 0 1 1 1 - 1 1 0 0 0 0 0 2 0 0 0 0 - 3 0 1 1 - - 0 1 1 0 1 0 1 0 2 2 0 1 1 1 0 0 1 1 2 0 1 2 1 0 0 1 1 0 1 1 0 - - 0 0 0 0 0 ? 0 1 0 0 0 1 1 0 0 0 0 1 1 2 2 - 1 0 0 0 1 2 1 0 2 0 0 1 0 2 2 0 1 1 0 0 0 0 0 1 1 2 0 1 0 0 0 0 1 0 0 0 2 1 0 0 2 1 - 0 - 1 1 1 1 1 1 0 1 0 1 0 1 1 1 0

***Glyptops plicatulus***0 1 0 2 ? 1 0 0 1 0 0 ? 0 0 1 0 0 0 1 1 1 0 1 0 ? ? 1 0 - - - - - ? 0 0 1 - - - ? ? 0 ? 2 ? 2 - 0 0 0 0 2 - - - - - - - - - - - - - - - 1 0 ? ? ? ? ? 0 0 1 0 ? 0 0 2 0 1 1 1 0 0 0 0 1 1 1 0 ? 0 0 0 0 1 1 ? 2 ? 1 ? 1 - 2 1 0 0 1 (1 2) 0 0 0 0 0 0 1 0 0 1 1 1 1 0 1 - 0 0 0 0 - 0 0 ? 1 0 ? 1 ? ? ? ? ? 1 2 ? 0 0 1 - 1 0 ? 0 1 1 0 1 1 0 0 0 1 0 2 2 0 ? 1 1 ? 0 ? ? 0 0 0 2 1 0 0 ? 1 0 ? ? ? - - 0 0 0 0 0 1 ? 0 0 ? 1 1 1 0 ? 0 1 ? 1 1 2 - 1 0 0 1 0 2 0 0 2 0 ? ? 0 2 2 0 {1 2} 1 ? 0 0 ? 0 1 ? 2 0 ? ? 0 0 ? 1 0 0 0 2 0 ? - 2 1 - 0 - 1 1 1 0 1 1 0 1 ? 1 ? ? 0 0 0

***Gopherus polyphemus***0 1 0 2 0 0 1 0 0 0 1 0 1 1 2 1 0 0 1 0 - 0 1 1 0 0 1 1 1 0 1 0 1 1 1 1 0 1 2 1 1 1 0 1 2 1 2 - 0 0 0 1 2 - - - - - - - - - - - - - - - 1 0 (1 2) - - - - 1 0 1 0 1 1 0 2 0 1 1 1 0 0 0 0 0 2 1 0 0 1 - - - - - - - - 1 0 0 1 0 1 0 0 1 0 0 0 0 0 0 1 1 0 0 1 0 1 1 0 1 - 0 0 0 1 0 0 0 1 1 0 - 1 0 0 0 0 0 0 2 0 0 0 2 0 3 0 0 1 - - 0 0 1 0 1 0 1 0 2 2 0 1 1 1 0 0 1 1 2 0 1 2 1 0 0 1 1 0 1 1 1 - - 0 0 0 0 0 0 0 1 0 0 0 1 1 0 0 0 0 0 1 2 2 - 1 0 0 0 1 0 0 0 2 0 0 1 0 2 2 0 3 1 0 0 0 0 0 1 ? 2 ? 1 0 0 0 0 1 0 0 0 2 1 0 0 2 1 - 0 - 1 1 1 1 1 1 0 1 0 1 0 1 1 1 0

***Hangaiemys hoburensis***0 1 (0 1) 2 0 ? 1 ? 1 0 0 ? 0 1 1 1 0 0 1 0 - 0 ? ? ? ? 1 1 1 0 0 0 0 1 0 ? ? 1 2 0 ? ? ? ? ? ? ? ? 0 0 0 0 2 ? ? ? ? ? ? ? ? ? ? ? ? ? ? ? 0 ? ? ? ? ? ? 1 ? 1 ? ? 0 ? ? 0 1 1 1 0 0 0 1 1 ? ? 0 0 1 - - - - - - - - ? ? ? ? 0 1 0 0 1 1 0 0 ? ? ? ? ? 0 0 0 ? ? 0 ? ? ? ? ? 0 0 - 0 0 ? 1 0 ? 1 ? ? ? ? ? ? 2 0 1 0 ? ? 3 0 ? 0 0 1 0 ? 1 0 ? 0 1 0 2 2 0 0 1 1 0 0 0 ? 1 0 0 ? 1 ? 0 ? 1 ? ? ? ? - - 0 0 1 0 0 0 0 1 0 0 0 1 1 0 0 0 0 0 1 1 2 - 1 0 0 1 0 2 0 0 2 0 ? ? 0 2 2 ? 1 1 0 0 0 0 0 1 ? ? ? 0 0 0 0 0 1 0 0 0 2 1 0 0 ? 1 ? ? ? 1 1 1 1 1 1 0 1 0 1 0 0 0 0 0

***Heckerochelys romani***0 1 0 ? ? 1 ? 0 ? 0 0 ? 0 0 0 0 0 0 1 0 - 0 ? ? ? ? 1 0 - - - - - ? ? ? ? - - - ? ? ? ? 2 ? ? ? 0 ? 0 0 2 ? ? ? ? ? ? ? ? ? ? ? ? ? ? ? ? ? ? 1 0 1 0 ? ? ? ? ? 0 ? ? 0 1 1 0 0 0 0 1 ? 1 ? 0 ? 0 0 0 0 1 1 1 2 0 ? ? 1 - 0 1 0 0 1 2 0 0 ? ? ? ? ? 0 ? 0 0 1 0 0 ? - ? ? 0 0 - ? 0 ? 1 ? ? 1 ? ? ? ? ? 0 2 ? ? ? ? ? 1 0 1 0 ? ? 0 0 0 0 0 0 1 0 1 2 ? 0 ? ? 0 ? - ? 0 0 0 ? 1 ? 0 ? ? ? ? ? ? - - 0 0 1 1 0 ? ? 0 0 ? ? ? 1 ? ? ? ? ? 1 1 0 - 0 0 ? 0 ? 2 0 0 2 0 ? ? 0 1 2 ? 0 ? ? 0 ? ? ? 1 ? ? ? 0 ? ? 0 ? ? ? ? 0 ? 0 ? - 2 ? - 0 - ? ? 1 0 ? 1 ? 1 ? 1 ? ? ? 0 0

***Helochelydra nopcsai***? ? ? 2 0 1 0 ? 3 - - ? 1 1 2 1 0 ? 1 1 3 ? ? ? ? ? ? ? ? ? ? ? ? ? ? ? ? ? ? ? ? ? ? ? ? ? ? - ? ? ? ? ? - - - - - - - - - - - - - - - 1 0 ? ? ? ? ? ? ? ? ? ? ? ? ? ? ? ? ? ? ? ? ? ? ? 1 0 ? ? ? ? ? ? ? ? ? ? ? ? 1 - 0 1 0 ? ? ? ? ? ? ? 0 0 1 ? ? ? ? ? ? ? ? ? ? ? ? ? ? ? ? ? 1 0 0 1 ? ? ? ? ? ? ? - 0 - 0 - ? ? ? 0 ? 1 ? ? ? ? ? ? 1 0 1 ? ? 0 1 ? 0 ? ? ? ? ? ? 2 1 ? ? ? ? ? ? ? ? ? ? ? ? ? ? ? 1 0 0 0 ? 1 1 1 0 ? 0 0 ? 1 2 2 - 1 0 ? 0 ? 0 1 0 ? - ? ? ? 2 2 0 1 1 0 0 0 1 0 ? ? 1 ? 0 0 0 0 0 1 ? ? ? ? 1 0 0 ? 1 ? ? ? 1 1 ? ? ? ? ? ? ? 1 ? ? ? ? ?

***Hoplochelys crassa***1 1 0 ? ? ? ? ? ? ? ? ? ? ? ? ? 0 2 1 0 - ? ? ? ? ? 1 ? ? ? ? ? ? ? ? ? ? ? ? ? ? ? ? ? ? ? ? - 0 0 0 ? ? ? ? ? ? ? ? ? ? ? ? ? ? ? ? ? ? ? ? - - - - 1 0 1 ? ? ? ? ? ? 1 1 1 0 0 0 0 0 2 ? ? ? 1 - - - - - - - - ? ? ? ? ? ? ? 0 ? ? 0 - ? ? ? ? ? 0 ? 0 0 ? 0 ? ? - ? ? 0 0 - ? 0 ? ? ? ? ? ? ? ? ? ? 0 2 ? ? ? ? ? 3 ? ? ? ? ? 0 ? 1 1 ? ? ? ? ? ? ? ? ? ? ? ? ? ? ? 1 - 2 1 ? ? ? 1 ? ? ? ? - - 0 0 0 0 0 ? ? ? ? ? ? ? ? ? ? ? ? ? ? ? ? ? ? ? ? ? ? ? ? ? ? ? ? ? ? ? ? ? ? ? ? ? ? ? ? ? 1 2 ? ? ? ? ? ? ? ? ? ? 2 ? ? ? ? ? ? ? ? ? ? 1 1 1 ? ? ? ? ? ? ? ? 0 0

***Indochelys spatulata***? ? ? ? ? ? ? ? ? ? ? ? ? ? ? ? 0 0 1 0 - 0 ? ? ? ? ? ? ? ? ? ? ? ? ? ? ? ? ? ? ? ? ? ? 2 ? 2 ? 0 0 0 0 2 ? ? ? ? ? ? ? ? ? ? ? ? ? ? ? ? ? ? ? ? ? ? ? ? ? ? 1 ? ? ? ? 0 ? ? 0 0 0 0 ? ? ? ? ? ? ? ? 0 ? ? ? ? ? ? ? ? ? ? ? ? ? ? ? ? ? ? ? ? ? ? 0 ? ? ? ? ? ? ? ? ? ? ? ? ? 0 ? ? ? ? ? ? ? ? ? ? ? ? ? ? ? ? ? ? 1 0 ? ? ? ? 0 0 ? ? 0 ? ? ? ? ? ? ? ? ? ? ? ? ? ? ? ? ? 1 0 ? ? ? ? ? ? ? - - 0 ? ? ? 0 ? ? ? ? ? ? ? ? ? ? ? ? ? ? ? ? ? ? ? ? ? ? ? ? ? ? ? ? ? 0 ? ? ? ? ? ? ? ? ? ? ? ? ? ? ? ? ? ? ? ? ? ? ? 2 ? ? ? 2 ? ? ? ? ? ? 1 0 0 1 0 ? ? ? ? ? ? 0 0

***Judithemys sukhanovi***0 1 (0 1) 2 0 0 0 0 1 0 1 ? 0 1 1 1 0 0 1 0 ? 0 1 1 0 1 1 1 1 0 0 0 0 ? 1 1 0 1 2 0 1 (0 1) 0 1 2 1 ? - 0 0 0 0 2 - - - - - - - - - - - - - - - ? 0 ? - - - - 1 ? 1 0 ? 0 0 2 0 1 1 1 0 0 1 1 ? 2 ? 0 0 1 - - - - - - - - ? ? 1 ? 0 1 0 0 1 2 0 0 0 0 0 0 1 0 0 0 0 1 0 0 1 - 0 0 0 0 - 0 0 ? 1 0 - 1 1 0 ? 0 0 0 2 ? ? 0 ? ? 3 0 ? ? ? ? 0 1 1 0 0 0 1 0 2 2 ? ? 1 1 0 0 ? ? 1 0 0 2 1 0 0 ? 1 ? 1 1 0 - - 0 0 1 0 0 ? 0 1 ? ? 0 1 1 ? ? ? 0 ? 1 1 2 - 1 0 0 0 0 2 1 0 2 0 ? ? 0 2 2 0 1 1 ? 0 0 0 0 1 ? 2 ? ? 0 0 0 0 1 0 0 0 2 1 0 0 2 1 - 0 - 1 1 1 1 1 1 0 1 0 1 ? 0 0 0 0

***Jurassichelon oleronensis***0 1 ? 2 0 ? 1 0 0 0 1 ? 1 1 2 1 0 0 1 ? ? ? ? ? ? ? ? 0 - - - - - 1 ? ? 0 - - - 0 ? 0 ? ? ? ? ? 0 ? 0 ? ? ? ? ? ? ? ? ? ? ? ? ? ? ? ? ? 0 ? ? ? ? ? ? 0 ? ? 0 ? ? 0 ? ? ? ? ? ? ? ? ? ? ? 1 0 0 ? ? ? ? ? ? ? ? ? ? 0 1 - 0 1 0 ? ? ? 0 ? ? ? ? ? ? ? 0 1 0 ? ? ? ? ? ? ? ? ? ? ? ? ? 1 0 0 1 ? ? ? ? ? ? ? 0 ? 0 1 - 3 ? ? 0 0 1 0 1 1 0 0 0 1 0 2 2 0 0 1 1 0 1 0 ? 1 0 ? ? 1 ? ? ? ? ? ? ? ? - - 0 0 ? 1 0 0 0 0 0 0 1 1 1 0 0 0 0 1 1 2 2 - 1 0 1 0 - ? 1 0 2 0 ? ? ? 2 2 0 1 1 0 0 0 0 0 1 ? ? ? 0 0 0 0 0 1 0 0 ? 2 0 0 0 ? 1 ? ? ? 1 1 ? 0 ? 1 ? 1 1 1 0 0 0 ? ?

***Kallokibotion bajazidi***0 1 0 2 0 1 0 0 0 0 0 ? 0 0 1 0 0 0 1 0 - 0 ? 0 - - 0 0 - - - - - ? 0 1 0 - - - 0 ? 0 ? 2 0 2 - 0 0 0 0 2 ? ? ? ? ? ? ? ? ? ? ? ? ? ? ? 0 0 ? - - - - ? 0 1 ? ? ? 0 2 0 1 1 1 0 0 0 0 1 1 1 ? 0 0 0 0 0 1 1 1 2 0 0 ? 1 - 0 (0 1) 0 0 1 1 0 (0 1) 0 0 0 0 0 0 ? 1 0 ? 1 0 1 - 0 0 0 0 - 0 0 ? 1 0 0 1 ? ? ? ? ? 0 2 0 0 0 2 1 (1 2) 0 0 0 0 0 1 1 0 0 0 0 1 ? 1 2 0 0 1 ? 0 0 0 ? 0 0 0 2 1 0 0 {0 1} 1 ? ? ? ? - - 0 0 0 0 0 0 ? 0 ? ? 0 1 0 0 0 0 0 1 1 1 2 - 1 0 ? 0 ? 0 0 0 1 1 0 0 0 2 2 0 2 1 0 0 0 0 0 1 1 1 ? 0 0 0 0 0 1 0 0 0 2 ? 0 0 2 1 - 0 - 1 1 1 0 1 1 0 1 1 1 0 1 ? 1 0

***Kayentachelys aprix***0 1 0 1 - 1 0 0 1 0 0 ? 0 0 0 0 0 0 1 0 - 0 1 0 - - 1 0 - - - - - 0 0 ? 0 - - - ? ? 0 0 ? 0 ? - 0 0 0 0 1 ? ? ? ? ? ? ? ? ? ? ? ? ? ? ? 0 1 ? 1 0 1 0 0 0 0 ? ? 0 ? ? 0 0 0 1 0 0 0 0 ? 1 0 0 0 0 0 0 0 1 ? 1 1 0 ? 0 - - 0 1 0 0 1 1 0 0 0 ? 0 0 ? 0 ? 0 0 1 0 0 1 - 0 0 0 0 - 0 0 ? 1 0 0 1 ? ? ? 0 0 0 2 0 0 0 1 - 1 0 1 0 0 1 0 0 0 0 0 1 1 0 1 1 0 0 0 - 0 - - ? 0 0 0 2 1 0 0 1 (0 1) 0 ? ? ? - - 0 0 1 0 0 0 0 0 0 0 1 1 1 0 ? 0 0 0 0 1 0 - 0 0 0 0 0 0 0 0 2 0 0 0 0 1 2 0 0 0 - 0 0 0 0 0 ? ? ? 0 0 0 0 0 1 0 0 0 2 0 0 - 1 ? - 0 - 1 1 1 0 0 1 - 1 0 1 0 0 0 0 0

***Keuperotesta limendorsa***0 0 ? ? ? ? ? ? ? ? ? ? ? ? ? ? 0 0 1 0 - 0 ? 0 - ? 1 0 - - - - - 0 0 0 0 - - - 0 0 0 0 ? ? 0 0 ? ? 0 ? ? ? ? ? ? ? ? ? ? ? ? ? ? ? ? ? ? ? ? 0 ? ? 0 0 ? ? 0 ? 0 0 ? 0 ? 0 ? ? ? ? ? ? 1 ? ? ? ? ? ? ? ? ? ? ? ? ? ? ? ? ? ? ? ? ? ? 0 ? ? ? ? ? ? 0 ? ? ? ? 0 ? 1 ? 0 0 0 0 - ? ? ? ? ? ? ? ? ? ? ? ? ? ? ? ? ? ? ? 0 1 ? ? ? ? ? ? ? 0 ? 0 ? ? ? ? ? ? ? ? ? ? ? ? ? 0 0 0 1 ? 1 0 ? 0 ? ? ? - - 0 0 0 0 0 ? ? ? ? ? ? ? ? ? ? ? ? ? ? ? ? ? ? ? ? ? ? ? ? ? ? ? 0 0 1 ? ? ? ? ? ? ? ? ? ? ? ? 0 1 ? ? ? ? ? ? ? ? 0 1 ? ? ? ? ? ? ? ? ? ? 1 0 ? 0 ? ? ? ? ? ? ? ? 0

***Kinosternon flavescens***? 2 0 2 0 0 1 0 0 0 1 1 1 1 2 1 0 1 1 0 - 0 1 1 0 0 1 1 1 ? ? ? 1 1 1 1 0 1 1 0 1 1 0 1 2 1 2 - 1 1 0 0 2 - - - - - - - - - - - - - - - 1 0 3 - - - - 1 1 - 0 2 1 1 2 0 - - - ? 1 - - 1 2 1 0 0 1 - - - - - - - - 1 0 ? ? 0 0 0 0 1 1 1 - 1 0 0 1 1 0 0 0 2 1 0 1 1 - 1 1 0 1 1 0 1 0 1 0 ? 1 1 0 ? 0 0 0 2 0 0 0 0 - 3 0 0 1 - - 0 1 1 1 0 0 1 0 2 2 1 1 1 1 0 0 1 1 2 1 - 2 1 0 0 1 2 0 1 1 0 1 1 0 0 0 0 1 ? 0 1 0 0 0 1 1 0 0 0 0 0 1 2 2 - 1 0 0 0 1 0 0 0 2 0 1 1 0 2 2 0 3 1 1 0 0 1 0 1 1 2 0 1 0 0 0 0 1 1 0 0 2 1 0 0 1 1 - 0 - 1 1 1 1 1 2 0 1 0 1 0 0 0 0 0

***Lissemys punctata***- - - 2 0 0 1 0 0 0 1 2 1 1 2 1 2 0 1 1 2 - 1 1 - - - 1 2 0 1 0 1 1 0 1 0 0 - 0 1 1 0 0 2 1 2 - 0 1 - 0 2 - - - - - - - - - - - - - - - 1 0 4 - - - - 1 0 1 0 1 - 1 2 - 1 1 1 1 0 1 - 1 2 1 0 0 - - - - - - - - - 1 0 0 0 1 1 0 - - - - - 1 1 0 1 1 1 0 0 - 1 0 - 1 - 0 1 - - - - - 0 1 1 - 1 1 0 1 0 1 - - 1 0 0 0 - 3 ? 0 1 - - 0 1 1 0 0 - 1 0 2 2 1 1 1 1 0 0 1 1 2 - - 2 1 0 0 2 3 ? 1 1 0 - - 1 - 1 1 0 0 0 1 0 1 0 1 1 1 2 1 0 0 1 2 2 - 1 0 0 1 1 0 1 0 1 0 1 1 - 2 2 0 3 1 1 0 0 0 0 1 1 2 0 1 0 0 1 0 1 0 0 - - 1 0 1 0 1 - 0 - 1 1 - - - - - 1 1 1 1 0 0 0 -

***Macroclemys schmidti***? ? ? 2 0 0 1 ? 1 0 1 0 1 1 2 1 ? ? ? ? ? ? ? ? ? ? ? ? ? ? ? ? ? ? ? ? ? ? ? ? ? ? ? ? ? ? ? ? ? ? ? ? ? ? ? ? ? ? ? ? ? ? ? ? ? ? ? ? 0 ? ? ? ? ? ? ? ? ? ? ? ? ? ? ? ? ? ? ? ? ? ? ? ? 1 0 0 ? ? ? ? ? ? ? ? ? ? ? 0 1 0 0 0 ? ? ? ? ? ? ? ? ? ? ? ? ? ? ? ? ? ? ? ? ? ? ? ? ? ? ? 1 0 ? 1 ? ? ? ? ? ? ? 0 1 0 0 - ? ? ? 1 ? ? ? ? ? ? ? ? 1 0 2 ? ? 1 1 1 0 ? 0 1 2 ? ? ? ? ? ? ? ? ? ? ? ? ? ? ? ? ? ? ? 0 ? 1 ? ? 0 1 1 0 ? 0 1 0 1 2 2 - 1 0 0 0 ? 2 1 0 2 0 ? ? ? 2 2 0 3 1 0 0 0 0 0 1 ? ? ? 0 0 0 0 0 1 0 0 ? ? 1 0 0 ? 1 ? ? ? ? ? ? ? ? ? ? 1 0 1 0 0 0 ? ?

***Macroclemys temminckii***1 1 1 2 0 0 1 0 0 0 1 0 1 1 2 1 0 0 1 0 - 0 1 1 1 1 1 1 1 0 1 0 1 1 1 1 0 1 2 0 1 1 0 1 2 0 2 - 0 0 0 0 2 ? ? ? ? ? ? ? ? ? ? ? ? ? ? ? 0 0 1 - - - - 1 0 1 0 1 1 0 (0 1) 0 1 1 1 0 0 1 1 1 2 1 0 0 1 - - - - - - - - ? 0 0 1 0 0 0 0 1 2 0 ? 1 0 0 0 1 0 0 0 2 1 0 1 1 - 0 0 0 0 - 0 0 1 1 0 0 1 1 0 ? 0 0 0 1 0 1 0 0 - 3 0 0 1 - - 0 0 1 2 0 1 1 0 2 2 0 1 1 1 0 0 0 1 2 0 0 2 1 0 0 1 1 0 1 1 1 - - 0 0 1 1 0 0 0 1 0 0 0 1 1 0 0 0 1 0 1 2 2 - 1 0 0 ? 1 2 1 0 2 0 0 0 0 2 2 0 3 1 0 0 0 0 0 1 1 2 0 0 0 0 0 0 1 0 0 0 1 1 0 0 1 1 - 0 - 1 1 1 1 1 1 0 1 0 1 0 0 0 0 0

***Meiolania platyceps***? ? ? 0 0 1 0 0 0 0 0 - 0 1 0 1 0 0 1 0 - 0 0 1 1 1 1 1 1 0 0 0 0 0 0 0 0 1 2 0 0 0 0 0 2 0 2 - 0 0 0 ? 2 1 1 1 1 1 1 1 1 0 1 1 1 1 0 1 0 0 ? 1 0 1 0 0 0 ? 0 1 0 0 {0 1} 0 1 0 1 0 0 0 0 ? 1 0 1 0 0 0 0 0 1 1 1 0 0 1 0 ? ? 0 0 0 0 1 2 0 ? 0 0 0 0 0 ? 1 0 1 ? 0 0 1 - 0 0 ? ? ? 0 0 ? 1 0 0 1 0 0 0 0 0 1 (0 1) 0 0 0 2 1 ? 0 0 0 0 0 ? ? 0 0 0 0 1 0 1 2 0 0 1 - 0 0 0 1 0 ? ? 2 1 0 0 1 1 ? ? ? 0 - - 0 0 1 1 0 0 0 0 0 0 0 1 (0 1) 0 0 0 0 0 1 2 1 1 1 0 0 0 (0 1) 0 0 0 1 1 0 0 0 2 2 0 1 1 0 0 0 0 1 1 1 0 0 0 1 2 0 0 1 0 0 ? 2 1 1 0 ? 1 1 1 1 1 1 1 0 ? 1 ? 1 0 1 0 1 1 0 0

***Mesodermochelys undulatus***- ? - ? ? ? ? ? ? ? ? ? ? ? ? ? 1 0 1 0 - 0 1 1 0 0 ? 1 1 0 0 0 0 1 1 0 0 1 2 0 0 0 0 0 2 1 2 - 0 0 1 0 2 ? ? ? ? ? ? ? ? ? ? ? ? ? ? ? ? 0 ? - - - - 1 0 1 ? 1 0 ? ? ? 1 1 1 0 0 1 1 ? 2 ? ? ? - - - ? ? ? ? ? ? ? ? ? ? ? ? ? - ? ? - - 1 1 1 - 1 0 ? 0 ? 1 0 ? 1 - 0 0 - - - 0 - 0 ? ? ? ? ? 1 ? 2 0 - 2 ? ? ? ? ? 3 0 0 ? ? ? 0 1 2 0 0 0 ? ? ? ? ? ? ? ? ? ? ? ? ? - - 2 1 0 0 1 1 ? ? ? ? - - 1 - 1 1 0 ? ? ? ? ? ? ? ? ? ? ? ? ? ? ? ? ? ? ? ? ? ? ? ? ? ? ? 1 1 0 ? ? ? ? ? ? ? ? ? ? ? 1 2 0 ? ? ? ? ? ? ? ? - 2 1 0 ? 1 ? - 0 - ? ? ? ? ? ? - ? ? ? ? ? ? 0 1

***Mongolemys elegans***0 1 0 2 0 0 0 0 1 0 1 ? 0 1 1 1 0 0 1 0 - 0 1 1 0 0 1 1 1 0 1 0 1 ? ? ? ? 1 2 1 1 1 0 1 ? 0 ? - 0 0 0 ? 2 - - - - - - - - - - - - - - - 1 0 ? - - - - 1 ? 1 ? 1 1 0 ? 0 1 1 1 0 0 0 0 1 2 1 ? ? 1 - - - - - - - - 0 0 ? ? 0 1 0 0 1 1 0 0 ? ? ? ? ? 0 0 1 0 1 1 0 1 - 0 ? 0 0 - 0 0 ? 1 0 - 1 1 ? ? 0 0 0 2 0 0 0 2 0 3 0 0 1 - - 0 1 1 0 1 0 1 0 2 2 0 1 1 1 0 0 ? 1 2 0 0 2 1 0 0 ? 1 0 ? ? 0 - - 0 0 0 0 0 0 0 (0 1) 0 0 0 1 1 0 0 0 0 0 1 2 2 - 1 0 0 0 0 0 0 0 2 0 ? ? 0 2 2 0 ? 1 0 0 0 0 0 1 1 ? ? 0 0 0 0 0 1 0 0 0 2 1 0 ? 2 1 - 0 - 1 1 1 1 1 1 {0 1} 1 0 1 0 0 0 0 0

***Mongolochelys efremovi***0 1 0 2 1 1 0 ? 2 0 0 - 0 0 1 0 0 0 1 0 - 0 1 1 0 1 1 1 1 0 0 0 0 0 0 0 0 1 1 1 0 0 0 0 2 0 2 - 0 0 0 0 (1 2) 0 0 0 0 0 1 - - 0 1 1 - 0 0 0 0 0 ? ? ? ? ? 0 0 0 ? ? 0 0 2 0 1 0 1 0 0 0 1 ? 1 0 0 0 0 0 0 0 1 1 ? 1 ? 1 0 1 - 0 0 0 0 1 1 0 0 0 0 0 0 1 0 ? 0 0 1 0 0 1 - 0 0 0 0 - ? 0 ? 1 0 1 1 1 0 0 0 0 0 2 0 0 0 2 1 1 0 ? 0 0 1 0 0 0 0 0 2 1 0 1 2 0 0 1 0 0 0 0 1 0 0 0 2 1 0 0 1 1 ? ? 1 0 - - 0 0 1 1 0 0 0 0 0 0 0 1 1 0 0 0 0 1 1 1 2 - 0 0 0 0 0 (0 1) 0 0 0 1 0 ? ? 1 2 0 1 1 0 0 0 0 0 1 ? 1 0 0 1 1 0 0 1 0 0 0 2 1 0 0 (1 2) 1 - 0 - 1 1 1 0 1 1 0 1 0 1 0 1 1 0 0

***Myuchelys latisternum***0 1 0 2 0 0 1 0 1 0 0 ? 1 1 2 3 0 0 1 ? ? 0 1 1 0 0 0 1 1 0 0 0 0 1 0 0 1 1 3 1 1 0 0 0 2 1 2 - 1 2 0 0 2 - - - - - - - - - - - - - - - 1 0 ? - - - - 1 0 1 0 1 0 0 2 0 1 1 1 0 0 0 0 1 2 2 1 0 0 0 0 0 1 1 1 2 0 ? 0 1 - 0 1 0 1 1 2 0 0 ? 0 0 0 1 0 1 1 0 1 1 1 1 1 0 0 1 - - 0 0 0 1 0 - 1 1 ? ? 0 0 0 1 1 1 0 0 - 3 0 ? 0 1 1 0 - 1 0 1 0 1 0 0 2 0 0 1 1 0 0 - 1 0 0 0 2 1 0 1 2 1 0 1 1 ? - - 0 0 0 0 0 0 1 0 1 1 1 1 1 0 0 0 0 0 1 2 2 - 0 1 0 0 0 0 0 0 - 0 0 1 0 3 2 0 2 0 - 0 1 - - 1 1 2 ? 1 0 0 0 0 1 0 1 0 2 1 0 - 1 1 - 0 - 1 1 1 1 - 1 0 1 0 1 1 0 0 1 0

***Naomichelys speciosa***0 1 0 2 0 1 0 ? 3 0 0 ? 1 1 2 1 0 0 1 1 3 0 1 0 - - ? 1 1 0 0 1 0 0 0 1 0 1 2 0 0 1 0 ? ? 0 2 - 0 0 0 0 2 - - - - - - - - - - - - - - - 1 0 ? ? ? ? ? 0 0 1 ? 1 ? ? ? 1 ? 1 1 0 0 0 0 ? ? ? 0 ? ? ? ? ? 1 1 ? 2 ? 1 ? 1 - ? 1 0 0 ? ? 0 ? 0 0 0 0 1 0 ? 0 1 1 0 0 1 - 0 0 0 0 - 0 ? 1 1 0 0 1 1 0 0 0 0 ? 2 ? ? ? ? ? 1 0 0 0 1 1 0 1 ? 0 0 0 1 0 1 ? ? 0 ? ? 0 ? 0 1 0 0 0 2 1 0 0 {1 2} 1 ? 0 1 0 - - 0 0 1 1 0 1 ? 0 ? ? 1 1 1 0 ? 0 0 ? 1 2 2 - 1 0 ? ? ? ? 1 0 ? 0 0 ? 0 2 2 0 1 1 0 0 0 ? 0 ? 1 1 0 0 0 0 0 0 ? 0 0 0 2 1 0 0 3 1 - 0 - 1 1 1 0 1 1 0 ? ? ? ? ? ? 0 0

***Neurankylus eximius***0 1 0 ? ? 0 1 0 0 0 1 ? 1 1 2 2 0 0 1 1 1 0 ? ? ? ? 1 ? ? ? ? ? ? ? ? ? ? ? ? ? ? ? ? ? ? ? ? ? 0 0 0 0 2 ? ? ? ? ? ? ? ? ? ? ? ? ? ? ? ? ? ? ? ? ? ? 0 0 1 ? ? ? ? ? 0 1 1 1 0 0 0 0 ? ? 2 0 0 0 0 0 0 1 1 ? 2 ? ? 1 1 - ? 1 0 0 1 2 0 0 ? ? ? ? ? 0 0 1 ? ? 1 ? ? - ? ? 0 0 - 0 0 ? ? ? ? ? ? ? ? ? ? 1 0 ? ? ? ? ? 1 0 ? ? ? ? 0 1 1 0 0 0 1 ? 2 2 ? ? 1 1 ? 0 0 ? 1 0 0 ? 1 0 0 ? 1 ? ? ? ? - - 0 0 0 0 0 ? 0 0 ? ? 1 1 ? ? ? ? ? 0 1 2 2 - 1 0 ? 0 ? ? 0 0 ? 0 ? ? 0 2 2 0 1 1 0 0 ? ? ? 1 ? ? ? ? 0 0 0 ? 1 0 0 0 2 0 0 0 2 1 ? ? ? 1 1 1 1 1 1 0 ? ? ? ? ? ? ? 0

***Ninjemys oweni***? ? ? ? 0 ? ? ? ? ? ? ? ? ? ? ? ? ? ? ? ? ? 0 ? ? ? ? ? ? ? ? ? ? ? ? ? ? ? ? ? ? ? ? ? ? ? ? ? ? ? ? ? ? 1 1 1 0 0 0 0 0 0 0 1 1 0 0 1 0 ? ? ? ? ? ? ? ? ? ? ? ? ? ? ? ? ? ? ? ? ? ? ? ? ? ? ? ? ? ? ? ? ? ? ? ? ? ? ? ? ? ? ? ? ? ? ? ? ? ? ? ? ? ? ? ? ? ? ? ? ? ? ? ? ? ? ? ? ? ? ? 0 ? 1 ? ? ? ? ? ? ? ? 0 0 2 1 ? ? ? 0 ? ? ? ? ? ? ? ? ? ? ? ? ? ? ? ? 0 ? ? ? 0 ? ? ? ? ? ? ? ? ? ? ? ? ? ? ? ? ? ? ? ? ? ? ? ? ? ? 1 ? ? 0 0 ? ? ? ? ? ? ? 0 ? ? ? ? ? ? ? ? ? ? ? 2 ? ? ? ? ? ? ? ? ? ? ? ? ? ? 2 0 ? ? ? ? ? ? 1 ? 0 ? ? 1 1 0 1 1 ? ? ? ? ? 1 ? 1 0 1 1 ? ?

***Niolamia argentina***? ? ? ? 0 1 0 ? 0 0 0 - 0 1 0 1 0 ? 1 0 - ? 0 ? ? ? ? ? ? ? ? ? ? ? ? ? ? ? ? ? ? ? ? ? ? ? ? ? ? ? ? ? ? 0 0 0 0 0 (0 1) 0 0 (0 1) 0 0 0 (0 1) 1 1 0 0 ? ? ? ? ? ? ? ? ? ? ? ? ? ? ? ? ? ? ? ? ? ? ? {0 1} ? 0 ? ? ? ? ? ? ? ? ? ? ? ? ? 0 0 0 ? ? ? ? ? ? ? ? ? ? ? 1 ? ? ? ? ? ? ? ? ? ? ? ? ? ? ? 1 0 ? 1 ? ? ? ? ? ? (0 1) 0 0 0 1 - ? 0 ? 0 0 0 ? ? ? ? ? ? 1 0 1 ? 0 0 ? ? 0 ? 0 1 0 ? ? ? ? ? ? ? ? 0 ? ? ? ? ? ? ? ? ? ? ? 0 0 0 0 0 1 1 0 ? 0 0 0 1 2 1 0 1 0 0 0 ? 0 0 0 1 1 ? ? ? ? 2 0 ? 1 0 0 0 0 1 1 ? ? ? 0 1 2 0 ? ? ? 0 ? ? 1 1 0 ? 1 1 1 0 1 1 ? ? ? ? ? 1 0 1 0 1 1 ? ?

***Notoemys laticentralis***0 1 0 2 ? 0 1 ? 0 0 0 ? 1 1 2 3 0 0 1 0 - 1 ? ? ? ? 1 1 ? ? ? ? ? 1 0 ? 1 ? ? ? 1 ? 0 ? 2 ? ? - 0 0 0 0 2 ? ? ? ? ? ? ? ? ? ? ? ? ? ? ? ? ? ? ? ? ? ? 0 0 1 ? 1 ? 1 1 0 1 1 ? 0 0 0 0 ? ? ? ? 0 0 0 1 0 1 ? ? 1 ? ? ? 1 - 0 ? ? 1 1 2 0 0 0 1 0 0 1 0 ? 1 1 1 0 0 ? 1 ? 0 1 - - 0 0 ? ? ? ? ? ? 0 0 ? ? 0 2 ? ? ? ? ? 2 0 0 ? ? ? 0 0 1 0 0 0 1 ? 0 2 ? ? ? ? ? ? - ? ? 0 0 2 1 0 1 ? 1 ? ? 1 0 - - 0 0 0 1 0 ? ? ? ? ? ? ? ? ? ? ? ? 0 ? 2 ? ? 0 ? ? ? ? ? 0 0 ? 0 ? ? 0 3 2 0 ? 0 - 0 ? ? ? 1 ? 2 ? ? ? 0 0 0 1 0 0 0 2 ? ? ? 2 ? - 0 - ? ? 1 0 0 1 1 ? ? ? ? ? ? 1 0

***Notoemys oxfordiensis***0 1 ? ? ? ? ? ? ? ? ? ? ? ? ? ? 0 0 1 ? ? 1 ? ? ? ? ? 1 ? ? ? ? ? 1 ? ? ? ? ? ? ? ? 0 ? 2 ? ? ? 0 ? 0 0 ? ? ? ? ? ? ? ? ? ? ? ? ? ? ? ? ? ? ? ? ? ? ? ? ? ? ? ? ? ? ? 0 1 1 1 0 0 0 0 ? ? ? ? ? 0 0 1 0 1 1 ? 1 ? ? ? ? ? ? ? ? 1 1 2 0 0 ? ? 0 ? 1 0 ? 1 1 ? 0 1 ? 1 ? 0 1 - - ? 0 ? ? ? ? ? ? ? ? ? ? ? ? ? ? ? ? ? 2 0 0 ? ? ? 0 0 ? 0 0 ? ? ? ? ? ? ? ? ? ? ? ? ? ? 0 0 ? 1 0 1 1 ? ? ? ? ? - - 0 0 0 1 0 ? ? ? ? ? ? ? ? ? ? ? ? ? ? ? ? ? ? ? ? ? ? ? ? ? ? ? 0 0 ? ? ? ? ? ? ? ? ? ? ? ? ? ? ? ? ? ? ? ? ? ? ? ? 2 ? ? ? ? ? - 0 - ? ? ? ? ? ? ? ? ? ? ? ? ? ? 0

***Notoemys zapatocaensis***0 1 0 ? ? ? ? ? ? ? ? ? ? ? ? ? 0 0 1 0 - 1 ? ? ? ? 1 ? ? ? ? ? ? ? ? ? ? ? ? ? ? ? ? ? ? ? ? ? 0 0 0 0 2 ? ? ? ? ? ? ? ? ? ? ? ? ? ? ? ? ? ? ? ? ? ? 0 0 1 1 1 ? 1 1 0 1 1 1 0 0 0 0 ? ? ? ? ? 0 0 1 0 1 1 ? 0 ? ? ? ? ? ? ? ? 1 1 2 0 0 ? ? ? ? ? 0 ? 1 {0 1} 1 0 0 1 1 ? 0 1 - - 0 0 ? ? ? ? ? ? ? ? ? ? 0 2 ? ? ? ? ? 2 0 0 ? ? ? 0 0 ? 0 0 0 ? ? ? ? ? ? ? ? ? ? ? ? ? 0 0 ? 1 0 1 {1 2} 1 ? ? ? ? - - 0 0 0 1 0 ? ? ? ? ? ? ? ? ? ? ? ? ? ? ? ? ? ? ? ? ? ? ? ? ? ? ? ? ? 0 ? ? ? ? ? ? ? ? ? ? ? ? ? ? ? ? ? ? ? ? ? ? ? 2 ? ? ? 2 ? ? ? ? ? ? 1 0 0 1 1 ? ? ? ? ? ? 1 0

***Ordosemys leios***0 1 1 2 ? ? 0 0 1 0 ? ? 0 ? 1 ? 0 0 1 0 - 0 1 1 0 1 1 1 1 0 0 0 0 0 ? 0 0 1 2 1 ? 0 0 ? 2 1 2 - 0 0 0 0 2 ? ? ? ? ? ? ? ? ? ? ? ? ? ? ? ? ? ? ? ? ? ? 0 ? 1 0 ? 0 0 ? ? ? ? ? 0 0 ? - ? ? 1 0 0 ? ? 0 ? ? ? ? ? ? ? ? ? ? ? 1 0 ? ? ? 0 ? 0 ? 0 0 1 0 0 0 0 1 0 0 1 - 0 0 0 0 - 0 ? ? ? 0 ? 1 ? ? ? ? ? 0 2 ? ? ? ? ? 3 0 1 0 0 1 0 1 1 0 0 0 1 0 2 2 0 ? 1 1 0 0 ? ? 2 0 ? 2 1 0 0 1 1 ? ? ? ? ? ? 0 0 1 1 ? 0 0 0 0 0 0 1 1 0 0 0 0 ? 1 1 2 - 1 0 0 0 0 ? ? 0 ? 0 0 ? 0 2 2 0 ? 1 ? ? 0 0 0 ? ? 2 ? ? 0 0 0 ? 1 0 ? 0 2 1 0 ? 2 1 - 0 - 1 1 1 1 1 1 0 1 0 1 0 ? ? 0 0

***Otwayemys cunicularius***1 1 ? 2 ? ? ? ? ? ? ? ? ? ? ? ? 0 0 1 ? ? 0 ? 1 1 ? ? 1 1 0 ? ? ? 0 ? ? 0 ? ? 1 0 0 0 ? ? 0 ? ? 0 ? 0 0 ? ? ? ? ? ? ? ? ? ? ? ? ? ? ? ? ? 0 ? ? ? ? ? 0 ? ? 0 ? 0 0 2 0 1 ? ? ? 0 0 ? ? ? ? ? ? 0 0 0 0 1 1 ? 2 ? ? ? ? ? ? ? ? 0 1 2 0 0 ? ? ? ? ? 0 ? ? ? ? 0 2 ? ? ? ? 0 0 - 0 0 ? ? ? ? ? ? ? ? ? ? ? ? ? ? ? 0 - 3 0 0 ? ? ? ? ? ? 0 ? ? ? ? ? ? ? ? ? ? ? ? ? ? (0 1) 0 0 ? 1 ? ? ? ? ? ? ? ? - - 0 0 1 1 0 ? ? ? ? ? ? ? ? ? ? ? ? ? ? ? ? ? ? ? ? ? ? ? ? ? ? ? ? ? 0 ? ? ? ? 1 ? ? ? ? ? ? 1 ? ? ? ? ? ? ? ? ? 0 ? 2 ? ? - ? ? ? ? ? ? ? ? 0 ? 1 ? ? ? ? ? ? ? 0 0

***Palaeochersis talampayensis***? ? 0 0 - 0 0 ? 0 0 0 ? ? ? 0 ? 0 0 1 0 - 0 1 0 - - - 0 - - - - - 1 0 0 0 - - - 0 0 0 0 2 0 1 0 ? ? 0 ? ? ? ? ? ? ? ? ? ? ? ? ? ? ? ? ? ? 0 ? 1 1 0 1 0 0 ? ? ? 0 0 ? ? 0 0 1 0 0 0 0 ? 0 ? 0 0 ? ? ? 0 0 0 0 0 0 1 ? - - 0 0 0 ? ? 2 ? ? 0 1 0 0 1 0 ? 0 ? 0 0 ? 0 ? ? 0 ? ? ? 0 1 ? 1 0 ? 0 0 0 0 0 0 ? 2 ? ? 0 0 - 1 0 0 0 0 0 ? ? ? 0 ? 0 1 1 1 0 0 0 0 - 1 - - ? 0 ? ? 1 1 1 0 {0 1} ? 1 ? ? ? - - 0 0 0 0 0 ? 0 0 ? 0 0 1 0 0 ? 0 0 ? 1 1 0 - 0 0 0 0 0 1 0 0 0 - 0 0 1 1 1 0 0 0 - 0 0 0 0 0 ? 0 0 0 0 0 0 0 ? ? ? ? {0 1} 0 0 - ? 0 - 0 - 1 1 ? ? ? ? ? 0 ? 1 ? 0 ? 0 0

***Patagoniaemys gasparinae***0 1 ? ? ? ? ? ? ? ? ? ? ? ? ? ? 0 0 1 0 - 0 ? 1 1 0 1 1 ? 0 0 0 0 0 0 ? 0 1 2 1 0 ? 0 ? ? 0 ? ? 0 ? ? 0 ? ? ? ? ? ? ? ? ? ? ? ? ? ? ? ? ? ? ? ? ? ? ? 0 ? ? 0 ? 0 0 ? ? ? ? ? ? ? ? ? ? ? ? ? ? ? ? ? ? ? ? ? ? ? ? ? ? ? ? ? ? ? ? ? ? ? ? ? ? ? ? 0 ? 0 {0 1} ? ? {0 1} 1 - 0 0 0 ? ? ? ? 1 ? ? ? ? ? ? ? ? ? ? ? ? ? ? ? ? ? 0 ? ? ? ? ? ? 0 0 0 {1 2} ? ? ? ? ? ? ? ? ? ? ? ? ? 0 0 ? 1 ? 0 {1 2} ? 0 ? ? ? ? ? 0 ? 1 ? ? ? ? ? ? ? ? ? ? ? ? ? ? ? ? ? ? ? ? ? ? ? ? ? ? ? ? ? ? ? ? ? ? ? ? ? ? ? ? ? ? ? ? ? ? ? ? ? ? ? ? ? ? ? ? ? ? ? ? ? ? ? ? ? ? 1 0 ? 1 ? ? ? ? ? ? ? ? ?

***Peligrochelys walshae***? ? ? 2 ? 1 0 ? 2 0 0 - 0 ? 1 ? 0 ? 1 0 - ? ? 1 1 1 1 1 ? 0 0 ? 0 0 0 ? 0 1 2 ? 0 ? 0 ? ? 0 ? - ? ? ? ? ? 0 ? ? ? ? ? ? ? ? ? ? ? 0 0 0 0 0 ? ? ? ? ? ? ? ? ? ? ? ? ? ? ? ? ? ? ? ? ? ? ? (0 1) 0 ? ? ? ? ? ? ? ? ? ? 0 ? 1 - 0 0 0 ? ? ? ? ? 0 0 0 0 1 ? 1 ? ? ? ? ? ? ? ? ? ? ? ? ? ? ? ? 0 0 ? ? 0 ? 0 0 ? ? ? ? ? 1 - ? 0 ? ? ? ? 0 0 ? ? ? {1 2} 1 0 1 2 ? ? ? ? ? ? 0 1 ? ? ? 2 1 ? ? ? ? 0 ? ? ? ? ? ? ? 1 ? ? 0 ? 0 ? ? ? ? ? 0 ? 0 0 0 ? ? 2 - 0 ? ? ? ? ? 0 ? ? 1 ? ? ? ? 2 0 1 1 0 0 0 ? ? 1 ? 1 ? ? ? 1 ? 0 1 0 0 ? ? 1 ? ? ? ? ? ? ? 1 1 ? ? ? ? ? ? ? ? ? ? ? ? ?

***Pelodiscus sinensis***- - - 2 0 0 1 0 0 0 1 ? 1 1 2 1 2 0 1 1 2 - 1 1 ? ? - 1 2 0 ? 0 1 1 0 ? 0 0 - 0 1 1 0 0 2 1 2 ? 0 1 - 0 2 - - - - - - - - - - - - - - - 1 ? ? - - - - 1 0 - 0 1 - 1 2 - 1 1 1 1 0 1 0 1 ? 1 0 0 - - - - - - - - - 1 ? 1 - 1 1 0 - - - - - ? ? 0 ? ? 0 0 - - ? - - ? ? ? ? - - - - - ? 1 1 - 1 ? 0 1 ? ? - - 1 0 0 0 - 3 - 0 1 - - 0 1 1 0 0 - 1 0 2 2 1 1 1 1 0 0 1 1 2 - - ? 1 0 ? 2 3 - ? ? 0 - - 1 - 1 1 0 0 0 1 0 1 0 1 1 ? 2 ? ? 0 1 2 2 - 1 0 0 1 0 2 1 0 1 0 ? ? - 2 2 0 3 1 1 0 0 0 0 1 1 ? 0 1 0 0 1 0 1 0 0 - - 1 0 1 0 1 - 0 - 1 1 - - - - - 1 1 1 1 0 0 0 0

***Pelomedusa subrufa***0 1 0 2 0 0 1 0 0 0 0 - 1 1 2 3 0 0 1 0 - 0 1 1 0 0 0 1 1 0 0 0 0 1 0 0 1 1 0 0 1 0 0 0 2 1 2 - 0 1 0 0 2 - - - - - - - - - - - - - - - 0 0 5 - - - - 1 0 1 0 1 0 0 2 0 1 1 1 0 0 0 0 1 2 2 1 0 0 0 0 0 1 1 1 1 0 1 0 1 - 0 1 0 1 1 1 0 0 1 0 0 0 1 0 1 1 1 1 1 0 1 1 0 0 1 - - 0 0 0 1 0 - 1 1 0 0 0 0 0 0 0 1 0 2 0 2 0 1 1 - - 0 1 1 0 1 0 1 0 0 2 0 1 1 1 0 0 - 1 2 0 0 2 1 0 1 2 1 0 1 1 0 - - 0 0 0 0 0 0 1 1 1 1 0 1 1 0 0 0 0 0 1 2 2 - 0 1 0 0 0 0 0 0 - 0 0 1 0 3 2 1 2 0 - (0 1) 0 0 0 1 1 2 0 1 0 0 0 0 1 0 1 0 2 1 0 0 1 1 - 0 - 1 1 1 1 1 1 0 2 1 1 1 - 0 1 0

***Phrynops geoffroanus***0 1 0 2 0 0 1 0 1 0 0 ? 1 1 2 3 0 0 1 0 - 0 1 1 0 0 1 1 1 0 0 0 0 1 0 0 1 1 3 1 1 0 0 0 2 1 2 - 0 1 0 0 2 - - - - - - - - - - - - - - - 1 1 6 - - - - 1 0 1 0 1 0 0 2 0 1 1 1 0 0 0 0 1 2 2 1 1 0 0 0 0 1 1 1 1 0 ? 0 1 - 2 1 0 1 1 2 0 0 1 0 0 0 1 0 1 1 1 1 1 0 1 1 0 0 1 - - 0 0 0 1 0 - 1 1 0 0 0 0 0 2 1 1 0 0 - 3 0 1 0 1 1 0 1 1 0 1 0 1 0 0 2 0 0 1 1 0 0 - 1 2 0 0 2 1 0 1 2 1 0 1 1 0 - - 0 0 0 0 0 0 1 0 1 1 1 1 1 0 0 0 0 0 1 2 2 - 0 1 0 0 0 0 0 0 - 0 0 1 0 3 2 0 2 0 - 0 1 - - 1 1 2 0 1 0 0 0 0 1 0 1 0 2 0 0 0 1 1 - 0 - 1 1 1 1 1 1 0 1 0 1 1 0 0 1 0

***Plastomenus* aff. *thomasii***- - - 2 ? 0 1 ? 0 0 1 2 1 1 2 1 2 0 1 1 2 - 1 ? ? ? - ? ? ? ? ? ? ? ? ? ? ? ? ? ? ? ? ? ? ? ? ? 0 1 - 0 2 - - - - - - - - - - - - - - - 1 0 ? - - - - 1 0 ? ? ? ? ? ? - ? ? ? ? ? 1 0 1 ? 1 0 0 - - - - - - - - - ? ? 0 1 1 1 0 - - - - - ? ? ? ? ? 0 ? - - ? - - ? ? ? ? - - - - - ? 1 1 ? 1 ? ? ? ? ? - ? 1 0 0 0 - 3 - 0 1 - - 0 1 1 0 0 - 1 0 2 2 ? 1 1 1 0 ? 1 1 2 - - ? ? 0 ? ? 3 - ? ? ? - - 1 - 1 0 0 0 ? 1 0 ? 0 1 1 - - - - 0 1 2 2 - 1 0 0 1 1 2 1 0 1 0 ? ? - 2 2 0 3 1 1 0 0 0 0 1 ? ? ? 1 0 0 ? 0 ? 0 0 - - 1 0 1 0 1 ? ? ? 1 1 - - - - - 1 1 1 1 0 0 ? ?

***Platychelys oberndorferi***0 1 0 ? ? ? ? ? ? ? ? ? ? ? ? ? 0 0 1 0 - 1 ? 1 0 0 1 1 1 0 1 0 1 1 0 ? ? ? ? 1 1 0 0 0 2 1 ? ? 0 0 0 0 2 ? ? ? ? ? ? ? ? ? ? ? ? ? ? ? ? ? ? - - - - 0 0 1 1 1 0 1 1 0 1 1 1 0 0 0 0 1 ? ? ? ? 0 0 1 0 1 1 ? 2 ? ? ? ? ? ? ? ? 1 1 2 0 0 ? ? ? ? ? 0 ? 1 0 ? 1 0 1 0 0 0 1 - - 0 0 ? ? ? ? ? ? ? ? ? ? 0 1 ? ? ? ? ? 2 0 1 ? ? ? 0 0 1 0 0 0 ? ? ? ? ? ? ? ? ? ? ? ? ? 0 0 ? 1 0 1 1 1 ? ? ? ? - - 0 0 0 1 0 ? ? ? ? ? ? ? ? ? ? ? ? ? ? ? ? ? ? ? ? ? ? ? ? ? ? ? 0 1 0 ? ? ? ? ? ? ? ? ? ? ? 1 ? ? ? ? ? ? ? ? ? ? 0 1 ? ? ? {1 2} ? - 0 - ? ? 1 0 0 1 {0 1} ? ? ? ? ? ? 1 0

***Platysternon megacephalum***0 1 0 2 0 0 1 1 0 0 1 0 1 1 2 1 0 0 1 0 - 0 1 1 0 1 1 1 1 0 1 0 1 1 0 1 0 1 2 1 1 1 0 0 2 0 2 - 0 0 0 0 2 - - - - - - - - - - - - - - - 1 0 2 - - - - 1 0 1 0 1 1 1 0 0 1 1 1 0 0 0 0 1 2 1 0 0 1 - - - - - - - - 1 0 0 1 0 0 0 0 1 1 0 0 0 0 0 0 1 0 0 0 2 1 0 2 1 - 0 0 0 0 - 0 0 1 1 0 0 1 1 0 ? 0 0 0 2 0 1 0 0 - 3 0 1 1 - - 0 0 1 0 0 1 1 0 2 2 0 1 1 1 0 0 0 1 0 0 0 2 1 0 0 ? 1 0 1 1 0 - - 0 0 1 0 0 1 0 1 0 0 0 1 1 0 0 0 1 0 1 2 2 - 1 0 0 0 1 2 1 0 2 0 0 1 0 2 2 0 3 1 0 0 0 1 0 1 ? 2 0 0 0 0 0 0 1 0 0 0 2 1 0 0 2 1 - 0 - 1 1 1 1 1 1 1 1 0 1 0 0 0 1 0

***Plesiobaena antiqua***0 1 1 2 0 1 1 0 0 0 1 ? 1 1 2 2 0 0 1 1 1 0 1 0 ? ? 1 1 1 ? ? ? ? 0 0 0 ? ? ? ? ? ? ? ? 2 0 2 ? 0 0 0 0 (1 2) - - - - - - - - - - - - - - - 1 0 ? ? ? ? ? 0 0 1 ? 1 0 ? ? 0 1 1 1 0 0 0 0 ? ? 2 0 0 0 2 ? 0 1 ? ? 2 ? ? 1 1 - ? 1 0 0 1 2 0 0 ? ? ? ? ? 0 0 1 ? 1 1 ? 1 - 0 ? 0 0 - 0 0 ? 1 0 ? 1 1 ? ? 0 0 0 0 0 1 0 0 - 1 0 ? 0 0 1 0 1 1 0 0 0 1 0 2 2 0 1 1 1 0 0 0 ? 1 0 0 ? 1 0 0 ? 1 ? 1 1 ? - - 0 0 0 0 0 (0 1) 0 0 0 0 2 1 1 0 0 0 0 ? 1 2 2 - 1 0 0 0 0 2 0 0 2 0 ? ? 0 2 2 0 1 1 0 0 0 0 0 1 ? ? ? 0 0 0 0 0 1 0 0 0 2 0 0 0 ? 1 - 0 - 1 1 1 1 1 1 0 1 0 1 0 0 0 0 0

***Plesiochelys etalloni***0 1 (0 1) 2 0 ? 1 0 1 0 1 ? 1 1 2 1 0 0 1 0 ? 1 ? ? ? ? 2 ? ? ? ? ? ? ? ? ? ? ? ? ? ? ? ? ? 2 ? 2 - 0 0 0 0 2 ? ? ? ? ? ? ? ? ? ? ? ? ? ? ? 0 0 ? - - - - 0 0 1 ? ? 0 ? ? 0 1 1 1 0 0 0 0 ? 2 1 0 0 0 0 0 0 1 1 1 (0 1) 0 ? 0 ? ? ? 1 0 0 1 1 0 0 ? ? 0 0 1 0 0 1 ? 1 1 0 1 - 0 ? 0 0 - 0 0 ? 1 0 0 1 1 ? ? 0 0 0 2 0 1 0 1 - 3 0 ? 0 0 1 0 1 1 0 0 0 1 0 1 2 0 (0 1) 1 0 0 1 0 ? 1 0 0 2 1 0 0 ? 1 0 1 1 ? - - 0 (0 1) 0 (0 1) 0 0 0 1 0 0 0 1 1 0 0 0 0 0 1 2 2 - 1 0 1 0 0 2 0 0 2 0 ? ? 0 2 2 0 {1 2} 1 0 0 0 0 0 ? ? ? ? 0 0 0 0 ? 1 0 0 0 2 1 0 0 2 1 ? ? ? 1 1 1 0 1 1 0 1 0 1 0 0 0 0 0

***Pleurosternon bullocki***0 1 0 2 0 ? ? 0 1 0 0 ? 0 0 1 0 0 0 1 1 1 0 ? ? ? ? 0 0 - - - - - 0 ? ? ? - - - ? ? 0 ? 2 ? ? ? 0 0 0 0 2 - - - - - - - - - - - - - - - 1 ? ? ? ? ? ? 0 0 ? 0 ? ? 0 2 0 1 ? ? 0 0 0 0 ? ? 1 0 0 0 0 0 0 1 1 ? 2 ? ? 1 ? ? ? 1 0 1 1 2 0 0 ? ? ? ? ? 0 ? ? 1 ? 1 0 1 - 0 0 0 0 - 0 0 ? 1 0 ? 1 ? ? ? ? ? 1 ? ? ? ? 0 - 1 0 ? 0 1 1 0 1 ? 0 0 0 1 0 1 2 0 0 1 1 0 0 ? ? 0 0 0 ? 1 0 0 ? 1 0 ? ? ? - - 0 0 0 0 0 1 ? 0 ? ? 1 1 1 0 0 0 0 ? 1 1 2 - ? 0 0 1 0 2 ? 0 2 0 ? ? 0 2 2 0 1 1 ? 0 0 0 0 ? 1 ? ? 0 0 0 0 0 1 0 ? 0 2 ? 0 ? 1 1 ? ? ? 1 1 1 0 1 1 0 1 ? 1 0 ? ? 0 0

***Podocnemis expansa***0 1 0 2 0 0 1 0 1 0 0 ? 1 1 2 - 0 0 1 0 - 0 1 1 0 0 0 1 1 0 0 0 0 1 0 0 1 1 0 0 1 0 0 0 ? 1 ? - 0 1 0 0 2 - - - - - - - - - - - - - - - 0 0 5 - - - - 1 0 1 0 1 0 0 2 0 1 1 1 0 0 0 0 1 2 2 1 0 0 0 0 0 1 1 ? 2 ? 0 0 1 - 2 1 0 1 1 2 0 1 1 1 0 0 1 0 1 1 1 1 1 0 1 1 0 0 1 - - 0 0 0 1 0 - 1 1 0 0 0 0 0 2 1 1 0 2 1 2 0 1 1 - - 0 1 1 0 1 0 1 0 0 2 0 1 1 1 0 0 - 1 0 0 0 2 1 0 1 2 1 0 1 1 0 - - 0 0 0 0 0 0 1 1 1 1 0 1 1 0 0 0 0 0 1 2 2 - 0 1 0 0 1 0 0 1 - 0 0 1 0 3 2 1 2 0 - 1 0 0 0 1 ? 2 ? 1 0 0 0 0 1 0 1 0 2 1 0 0 1 1 - 0 - 1 1 1 1 1 1 0 2 1 1 1 0 0 1 0

***Portlandemys macdowelli***? ? ? 2 ? 0 1 0 1 0 1 ? 1 1 2 1 ? ? ? ? ? ? ? ? ? ? ? ? ? ? ? ? ? ? ? ? ? ? ? ? ? ? ? ? ? ? ? ? ? ? ? ? ? ? ? ? ? ? ? ? ? ? ? ? ? ? ? ? 0 0 ? ? ? ? ? ? ? ? ? ? ? ? ? ? ? ? ? ? ? ? ? ? ? 1 0 0 ? ? ? ? ? ? ? ? ? ? 0 1 - 0 1 0 ? ? ? ? ? ? ? ? ? ? ? 0 ? ? ? ? ? ? ? ? ? ? ? ? ? ? ? ? ? ? 1 ? ? ? ? ? ? ? 0 1 0 1 - ? ? ? 0 0 1 ? ? ? ? ? ? 1 0 1 2 0 ? 1 0 0 1 0 ? 1 ? ? ? ? ? ? ? ? ? ? ? ? ? ? ? ? ? ? ? ? 0 1 0 0 0 1 1 0 0 0 0 0 1 2 2 - 1 0 0 0 0 2 1 0 2 0 ? ? ? 2 2 0 1 1 0 0 ? ? ? 1 ? ? ? ? 0 0 0 0 1 0 0 ? ? ? ? ? ? 1 ? ? ? 1 1 ? ? ? ? ? 1 0 1 0 0 0 ? ?

***Prochelidella cerrobarcinae***0 1 0 ? ? ? ? ? ? ? ? ? ? ? ? ? 0 0 1 0 - 0 ? ? ? ? 1 ? ? ? ? ? ? ? ? ? ? ? ? ? ? ? ? ? ? ? ? ? 0 1 0 0 2 ? ? ? ? ? ? ? ? ? ? ? ? ? ? ? ? ? ? - - - - ? ? ? ? ? ? ? ? 0 1 1 1 0 0 0 0 1 ? ? ? ? 0 0 0 0 1 1 1 1 0 1 ? ? ? ? ? ? 1 1 2 0 0 ? ? ? ? ? 0 ? ? ? ? ? ? ? ? ? 0 1 - - 0 0 ? ? ? ? ? ? ? ? ? ? 0 2 ? ? ? ? ? 2 0 0 ? ? ? 0 1 ? ? 1 0 ? ? ? ? ? ? ? ? ? ? ? ? ? 0 0 ? ? 0 ? ? 1 0 ? ? ? - - 0 0 1 0 0 ? ? ? ? ? ? ? ? ? ? ? ? ? ? ? ? ? ? ? ? ? ? ? ? ? ? ? ? ? 0 ? ? ? ? ? ? ? ? ? ? ? 1 ? ? ? ? ? ? ? ? ? ? 0 2 ? ? ? 1 ? ? ? ? ? ? 1 1 1 1 0 ? ? ? ? ? ? 1 0

***Proganochelys quenstedtii***0 1 0 0 - 0 0 0 0 0 0 ? 0 0 0 0 0 0 1 0 - 0 0 0 - - 1 0 - - - - - 0 0 0 0 - - - 0 0 0 0 1 0 1 0 0 0 0 ? 1 0 0 1 1 1 1 0 - 0 1 1 1 0 0 0 0 0 ? 1 1 0 1 0 0 0 0 1 0 0 0 0 0 0 0 0 0 0 0 ? 0 ? 0 0 0 0 1 (0 1) 0 1 0 0 1 1 0 - - 0 0 0 0 0 0 0 0 0 0 0 0 0 0 0 0 ? 0 0 ? 0 - 0 0 ? ? ? 0 1 ? 0 0 ? 0 0 0 0 0 0 ? 1 0 0 0 1 - 1 ? 0 0 0 0 ? 1 0 0 ? {1 2} 0 0 0 0 0 0 0 - 0 - - ? 0 0 0 0 1 1 0 0 0 0 0 0 0 - - 0 0 0 0 0 0 0 0 ? 0 0 0 0 0 0 0 0 1 0 0 0 - 0 0 0 0 0 1 0 0 0 - 0 0 1 0 0 0 0 0 - 0 0 0 0 0 1 0 1 0 0 0 0 0 0 ? 0 1 0 0 0 - ? 0 0 0 - 1 1 0 0 ? 1 ? 0 0 0 0 0 1 0 0

***Proterochersis porebensis***0 0 0 ? ? ? ? ? ? ? ? ? ? ? ? ? 0 0 1 0 - 0 ? 0 ? ? 1 0 - ? ? ? ? ? 0 ? 0 ? ? ? ? ? 0 ? 0 0 0 0 0 0 0 0 0 ? ? ? ? ? ? ? ? ? ? ? ? ? ? ? ? ? ? 0 0 1 0 ? 0 ? 0 0 0 0 1 0 0 0 ? 0 0 0 0 ? 1 ? ? ? 0 0 ? 0 (0 1) (0 1) 1 0 0 0 ? ? ? ? ? ? 0 (0 1) 0 0 ? 0 0 0 0 1 0 ? 0 ? ? 0 ? 1 ? 0 0 0 0 - 1 0 ? ? ? ? ? ? ? ? ? ? ? 0 ? ? ? ? ? 0 1 0 ? ? ? ? 1 1 0 0 0 ? ? ? ? ? ? ? ? ? ? ? ? ? 0 0 0 1 {0 1} 1 0 0 0 ? ? ? - - 0 0 0 0 0 ? ? ? ? ? ? ? ? ? ? ? ? ? ? ? ? ? ? ? ? ? ? ? ? ? ? ? 0 0 1 ? ? ? ? ? ? ? ? ? ? ? 0 0 0 ? ? ? ? ? ? ? ? 0 1 ? ? ? ? ? ? ? ? ? ? 1 0 ? 0 ? ? ? ? ? ? ? 1 0

***Proterochersis robusta***0 0 0 ? ? ? ? ? ? ? ? ? ? ? ? ? 0 0 1 0 - 0 ? ? ? ? 1 ? ? ? ? ? ? ? ? ? ? ? ? ? ? ? ? ? 0 ? ? ? 0 0 0 ? 0 ? ? ? ? ? ? ? ? ? ? ? ? ? ? ? ? ? ? 0 0 1 0 ? 0 ? 0 0 0 0 1 0 0 0 1 0 0 0 0 ? 1 ? ? ? 0 0 ? 0 1 1 1 0 0 ? ? ? ? ? ? ? 0 1 0 0 0 ? ? ? ? ? 0 ? 0 ? ? 0 ? 1 ? 0 0 0 0 - 1 0 ? ? ? ? ? ? ? ? ? ? ? 0 ? ? ? ? ? 0 1 0 ? ? ? ? 1 1 0 0 0 ? ? ? ? ? ? ? ? ? ? ? ? ? 0 0 ? ? 0 1 0 0 0 ? ? ? - - 0 0 0 0 0 ? ? ? ? ? ? ? ? ? ? ? ? ? ? ? ? ? ? ? ? ? ? ? ? ? ? ? 0 0 1 ? ? ? ? ? ? ? ? ? ? ? 0 ? ? ? ? ? ? ? ? ? ? 0 1 ? ? ? ? ? ? ? ? ? ? 1 0 ? 0 ? ? ? ? ? ? ? 1 0

***Protochelydra zangerli***1 1 1 ? 0 ? ? ? 0 ? ? ? ? ? ? ? ? ? 1 ? ? ? ? ? ? ? ? ? ? ? ? ? ? ? ? ? ? ? ? ? ? ? ? ? ? ? ? ? ? ? 0 ? ? ? ? ? ? ? ? ? ? ? ? ? ? ? ? ? ? ? ? - - - - ? ? ? ? ? ? ? ? ? 1 1 1 0 0 ? 1 ? 2 ? 0 0 1 - - - - - - - - ? 0 ? ? ? 0 ? ? ? ? 0 ? ? ? ? ? ? ? 0 0 ? ? 0 ? 1 - 0 ? 0 ? ? ? 0 ? 1 0 0 1 ? ? ? ? ? 0 ? ? ? ? ? ? 3 ? ? 1 - - ? ? ? ? ? ? 1 0 ? ? ? 1 1 1 0 0 ? ? ? 0 ? ? 1 ? 0 ? ? ? ? ? ? - - 0 0 1 0 0 0 0 1 0 ? 0 1 1 0 0 0 0 ? 1 2 2 - 1 0 0 0 1 ? ? ? ? 0 ? ? ? 2 2 0 ? 1 ? ? 0 0 0 ? ? ? ? 0 0 0 0 ? 1 0 ? ? ? 1 0 ? ? 1 ? ? ? 1 1 ? ? ? ? ? 1 0 1 ? ? ? 0 0

***Santanachelys gaffneyi***? ? ? 2 0 ? 1 ? 0 0 1 ? ? ? ? ? 0 0 1 ? ? 0 1 ? ? ? ? ? ? ? ? ? 0 1 ? ? ? ? ? ? ? ? ? ? ? ? 2 - 0 0 1 0 2 ? ? ? ? ? ? ? ? ? ? ? ? ? ? ? 0 0 ? ? ? ? ? 0 ? 1 ? ? ? ? ? ? 1 1 1 0 0 1 0 ? ? ? 0 0 ? ? ? ? ? ? ? ? ? ? ? ? ? ? 1 0 ? ? ? ? ? ? ? ? ? ? 0 0 0 1 1 0 ? ? - 0 ? ? ? ? 0 ? ? 1 0 1 1 1 1 ? 1 0 ? 2 ? 1 0 1 - 3 0 ? 0 0 1 0 1 ? 0 0 0 1 0 2 ? 0 1 1 0 0 0 ? ? 0 ? ? 2 1 0 0 ? 1 ? 1 1 ? - - ? 0 1 1 0 0 0 0 0 0 0 1 1 0 ? 0 0 ? 1 2 2 - 1 0 1 0 - ? 0 0 2 0 ? ? 0 2 2 0 ? 1 0 0 0 0 0 1 ? 2 0 0 0 0 0 ? 1 0 ? ? 2 1 0 ? 1 1 ? ? ? 1 1 1 0 0 1 0 1 1 1 0 0 0 0 0

***Siamochelys peninsularis***0 1 (0 1) ? ? ? ? ? ? ? ? ? ? ? ? ? 0 0 1 1 1 0 ? ? ? ? 1 ? ? ? ? ? ? ? ? ? ? ? ? ? ? ? ? ? ? ? ? ? 0 0 0 0 2 ? ? ? ? ? ? ? ? ? ? ? ? ? ? ? ? ? ? ? ? ? ? ? ? ? ? ? ? ? ? ? 1 ? 1 0 0 0 0 ? ? ? ? ? 0 0 0 0 1 1 ? 2 ? ? ? ? ? ? ? ? 0 1 2 0 0 ? ? ? ? ? 0 ? ? 1 ? 0 1 ? ? ? ? 0 0 - ? 0 ? ? ? ? ? ? ? ? ? ? 0 2 ? ? ? ? ? 1 0 1 ? ? ? 0 1 ? 0 0 ? ? ? ? ? ? ? ? ? ? ? ? ? ? 0 0 ? ? ? ? ? 1 ? ? ? ? - - 0 1 1 0 0 ? ? ? ? ? ? ? ? ? ? ? ? ? ? ? ? ? ? ? ? ? ? ? ? ? ? ? ? ? ? ? ? ? ? ? ? ? ? ? ? ? ? ? ? ? ? ? ? ? ? ? ? 0 2 ? ? ? 2 ? ? ? ? ? ? 1 1 ? 1 0 ? ? ? ? ? ? 0 0

***Sichuanchelys chowi***0 1 0 ? ? ? ? ? ? ? ? ? ? ? ? ? 0 0 1 0 - 0 ? ? ? ? 1 ? ? ? ? ? ? ? ? ? ? ? ? ? ? ? ? ? ? ? ? ? 0 ? 0 0 2 ? ? ? ? ? ? ? ? ? ? ? ? ? ? ? ? ? ? ? ? ? ? ? ? ? ? ? ? ? ? 0 1 ? 1 0 0 0 0 ? ? ? ? ? 0 0 0 0 1 1 ? 2 ? ? ? ? ? ? ? ? 0 1 1 0 0 ? ? ? ? ? 0 ? ? ? ? ? ? ? ? ? ? 0 0 - 0 0 ? ? ? ? ? ? ? ? ? ? 0 0 ? ? ? ? ? 1 0 ? ? ? ? 0 1 ? ? 0 2 ? ? ? ? ? ? ? ? ? ? ? ? ? 0 0 ? ? ? ? ? 1 ? ? ? ? - - 0 0 0 0 0 ? ? ? ? ? ? ? ? ? ? ? ? ? ? ? ? ? ? ? ? ? ? ? ? ? ? ? ? ? 0 ? ? ? ? ? ? ? ? ? ? ? ? ? ? ? ? ? ? ? ? ? ? ? 2 ? ? ? 2 ? ? ? ? ? ? 1 0 0 0 0 ? ? ? ? ? ? 0 0

***Sichuanchelys palatodentata***0 1 ? 2 1 1 0 ? 2 0 0 ? 0 0 1 0 0 0 1 0 - ? 1 ? ? ? ? ? ? ? ? ? ? ? ? ? 0 ? ? ? 0 ? ? ? ? 0 2 - 0 ? 0 0 ? 0 1 ? 1 ? ? 0 - - ? ? - ? 0 0 0 0 ? ? ? ? ? ? ? ? ? ? ? ? ? 0 1 ? 1 0 0 0 0 ? ? ? 0 0 0 0 0 0 1 1 ? 1 ? ? 1 1 - 0 0 0 0 1 1 0 0 1 ? 0 ? 1 0 ? ? {0 1} ? ? 0 ? ? ? ? ? ? ? ? 0 ? 1 0 1 1 ? ? ? ? ? ? 2 0 0 0 0 - 1 0 ? 0 0 0 0 1 ? ? 0 2 1 0 1 2 ? 0 ? ? 0 ? ? ? 0 0 0 2 1 0 0 {1 2} {0 1 2} ? ? ? ? - - 0 ? 1 1 0 0 ? 0 0 0 (0 1) 1 1 0 0 0 0 ? 0 1 2 - 0 0 0 0 0 {1 2} 0 0 {0 2} 0 ? ? ? 1 2 0 1 ? ? 0 0 0 0 1 ? 1 ? 0 0 0 0 0 (0 1) ? ? ? 2 1 0 0 ? 1 - - - 1 1 1 0 1 0 0 1 0 1 0 ? ? 0 0

***Sinemys lens***0 1 ? 2 ? 0 0 ? 1 0 ? ? 0 1 1 1 0 0 1 ? ? 0 1 ? ? ? 0 ? ? ? ? ? ? ? ? ? ? ? ? ? ? ? ? ? ? ? ? ? 0 0 0 0 2 - - - - - - - - - - - - - - - 1 0 ? ? ? ? ? 0 ? ? ? ? ? ? ? 0 1 1 1 0 0 1 0 1 ? ? ? 0 ? ? ? ? ? ? ? ? ? ? ? ? ? 0 1 0 ? ? ? 0 ? ? ? ? ? ? 0 0 0 0 ? 0 0 ? ? ? ? 0 ? ? 0 ? ? 1 0 ? 1 1 0 0 0 0 0 2 ? ? ? 0 - 3 0 1 0 0 1 0 1 1 0 0 0 1 0 2 2 ? 1 1 1 0 0 0 ? 2 0 0 ? 1 0 0 ? 1 ? 1 1 0 - - 0 0 1 (0 1) 0 0 ? 0 ? ? 1 1 1 ? ? 0 0 0 1 2 2 - 0 0 0 0 0 ? 0 0 2 0 ? ? 0 2 2 ? ? 1 ? 0 ? 0 0 1 ? ? ? ? 0 0 0 ? 1 0 0 ? 2 1 0 ? 2 1 ? ? ? 1 1 1 1 1 1 0 ? ? 1 ? ? ? 0 0

***Solnhofia parsonsi***0 1 0 2 0 1 1 0 0 0 1 ? 1 1 2 1 0 0 1 0 - 1 1 0 ? ? 1 0 - - - - - 1 0 ? 0 - - - ? ? ? ? ? ? 2 - 0 0 0 0 2 ? ? ? ? ? ? ? ? ? ? ? ? ? ? ? 0 0 ? ? ? ? ? ? ? 1 ? ? ? ? ? ? ? ? ? 0 0 1 1 ? ? 1 0 0 ? ? ? ? ? ? ? ? ? 1 0 1 - 0 1 0 ? ? ? 0 0 ? ? 0 0 1 0 0 0 0 1 0 0 ? - ? ? ? ? ? 0 ? ? 1 0 ? 1 1 0 0 0 0 ? 2 - 1 1 0 - 3 0 ? 0 0 1 0 1 ? ? 0 0 1 0 2 2 0 (0 1) 1 0 0 0 0 ? 1 0 ? 2 1 0 0 ? 1 ? 1 1 0 - - 0 0 1 1 0 0 0 1 0 0 0 1 1 0 0 0 0 0 1 2 2 - 1 0 0 0 0 0 1 0 1 0 0 ? 0 2 2 0 1 1 0 0 0 0 0 1 ? 2 ? ? 0 0 0 0 1 0 0 ? 2 1 0 0 3 1 ? ? ? 1 1 1 0 1 1 ? 1 1 1 0 - - 0 0

***Staurotypus triporcatus***? 2 0 2 0 0 1 0 0 0 1 1 1 1 2 1 0 2 1 0 - 0 1 1 0 ? 1 1 1 0 1 0 1 1 1 1 0 1 1 0 1 1 0 1 2 1 2 - 0 (0 1) 0 0 2 - - - - - - - - - - - - - - - 1 0 3 - - - - 1 1 - 0 2 1 0 2 0 1 1 1 0 0 1 0 1 2 1 0 0 1 - - - - - - - - 1 0 0 1 0 0 0 0 1 2 0 - 1 0 0 1 1 0 0 0 2 1 0 1 1 - 1 1 0 1 1 0 0 0 1 1 0 1 1 ? ? 0 0 0 2 ? 0 0 0 - 3 0 1 1 - - 0 1 1 1 0 0 1 0 2 2 1 1 1 1 0 0 1 1 2 1 - 2 1 0 0 2 2 0 1 1 ? 0 1 0 0 0 0 1 0 0 1 0 0 0 1 1 0 0 0 1 0 1 2 2 - 1 0 0 0 1 0 0 0 2 0 1 0 0 2 2 0 3 1 1 0 0 1 0 1 1 2 0 1 0 0 0 0 1 1 0 0 2 1 0 0 1 1 - 0 - 1 1 1 1 1 1 0 1 0 1 0 0 0 0 0

***Sternotherus odoratus***? 2 0 2 0 0 1 0 0 0 1 1 1 1 2 1 0 1 1 0 - 0 1 1 0 0 1 1 1 0 1 0 1 1 1 1 0 1 1 0 1 1 0 1 2 1 2 - 1 1 0 0 2 - - - - - - - - - - - - - - - 1 0 3 - - - - 1 1 - 0 2 1 0 2 0 - - - ? 1 - - 1 2 1 0 0 1 - - - - - - - - 1 0 0 1 0 0 0 0 1 1 1 - 1 0 0 1 1 0 0 0 2 1 0 1 1 - 1 1 0 1 1 0 1 0 1 0 0 1 1 0 0 0 0 0 2 0 0 0 0 - 3 0 1 1 - - 0 1 1 1 0 0 1 0 2 2 1 1 1 1 0 0 1 1 2 1 - 2 1 0 0 1 2 0 1 1 0 0 1 0 0 0 0 1 0 0 1 0 0 0 1 1 0 0 0 0 0 1 2 2 - 1 0 0 0 1 0 0 0 2 0 1 0 0 2 2 0 3 1 1 0 0 1 0 1 1 2 0 1 0 0 0 0 1 1 0 0 2 1 0 0 1 1 - 0 - 1 1 1 1 1 (1 2) 0 1 0 1 0 0 0 0 0

***Stylemys nebrascensis***0 1 0 2 0 ? ? ? ? 0 ? 0 1 1 2 1 0 0 1 0 - 0 ? ? ? ? 1 ? ? ? ? ? ? ? ? ? ? ? ? ? ? ? ? ? ? ? 2 - 0 0 0 0 2 ? ? ? ? ? ? ? ? ? ? ? ? ? ? ? 1 ? ? - - - - ? ? ? ? ? ? ? ? 0 1 1 1 0 0 0 0 1 ? ? ? ? 1 - - - - - - - - (0 1) ? ? ? ? 1 0 0 1 1 0 0 1 0 0 1 1 0 ? 1 1 1 2 ? ? - ? ? 0 1 0 0 0 1 1 0 - 1 0 0 0 0 0 0 2 ? ? 0 ? ? 3 0 0 1 - - 0 ? ? 0 ? 0 1 0 ? ? ? 1 1 ? 0 ? ? ? 2 0 1 2 1 0 0 1 1 ? 1 1 0 - - 0 0 0 0 0 0 ? 1 ? ? 0 1 1 ? ? ? ? ? 1 2 2 - ? 0 ? ? ? ? ? 0 ? ? 0 0 0 ? 2 0 ? 1 ? 0 0 0 0 ? 1 2 ? 1 0 0 0 0 ? ? ? 0 2 ? ? ? 2 1 ? ? ? 1 1 1 1 1 1 0 ? ? 1 ? ? ? 1 0

***Toxochelys latiremis***0 1 1 2 0 0 1 0 (0 1) 1 1 ? 1 1 2 1 0 0 1 0 - ? 1 1 ? ? 1 1 1 0 ? ? 0 1 ? 1 0 1 2 0 ? ? ? ? ? 0 2 - 0 0 1 0 2 - - - - - - - - - - - - - - - ? 0 ? ? ? ? ? ? 0 1 ? ? 0 ? ? ? 1 1 1 0 0 1 1 1 ? ? 0 0 ? ? ? ? ? ? ? ? ? ? ? 1 - 0 1 0 ? ? ? 0 ? ? ? ? ? ? 0 0 0 2 1 0 1 ? - 0 ? 0 ? ? ? ? ? 1 0 1 1 1 1 ? 1 0 0 2 0 1 0 0 - 3 0 0 1 0 1 0 1 2 0 0 1 1 0 2 2 0 1 1 ? 0 0 0 1 1 ? ? 2 1 0 0 ? 1 ? 1 1 ? - - 0 0 1 1 0 0 0 1 0 0 0 1 1 0 0 0 0 0 1 2 2 - 1 0 0 0 0 2 1 0 2 0 ? ? ? 2 2 0 1 1 0 0 0 0 0 1 ? ? ? 0 0 0 0 1 1 0 0 ? 2 1 0 0 2 1 - 0 - 1 1 1 1 1 1 ? 1 0 1 0 0 0 0 1

***Trachemys scripta***0 1 0 2 0 0 1 0 0 0 1 0 1 1 2 1 0 0 1 0 - 0 - - ? ? 1 - - 1 1 0 1 1 ? - 0 1 2 1 1 1 0 ? 2 - 2 - 0 0 0 1 2 - - - - - - - - - - - - - - - 1 0 0 - - - - 1 0 1 0 - 1 0 2 0 1 1 1 0 0 0 0 1 - 1 0 0 1 - - - - - - - - 0 0 0 1 0 1 0 0 2 1 0 0 1 0 0 1 1 0 0 1 1 1 1 0 1 - 0 0 0 1 0 0 0 1 1 0 - 1 - 0 0 - - 0 1 0 0 0 2 1 3 0 0 1 - - 0 1 1 0 1 1 1 0 2 2 0 0 1 1 0 0 1 1 2 0 0 2 1 0 0 1 1 0 - - 0 - - 0 0 0 0 0 0 0 1 0 0 0 1 1 0 0 0 0 1 1 2 2 - 1 0 0 0 1 2 0 0 2 0 0 1 0 2 2 0 1 1 0 0 0 0 0 1 ? 2 0 1 0 0 0 0 1 0 0 0 2 1 0 0 1 1 - 0 - 1 1 1 1 1 1 0 1 0 1 0 0 0 1 0

***Trinitichelys hiatti***0 1 ? 2 0 0 1 0 0 0 ? ? 1 1 2 2 0 0 1 1 1 ? ? ? ? ? 1 0 - - - - - ? 0 0 ? - - - ? ? ? ? ? ? ? ? 0 ? 0 ? ? - - - - - - - - - - - - - - - 1 0 ? ? ? ? ? ? ? 1 ? ? 0 ? ? 0 1 1 1 0 0 0 0 ? ? 2 0 0 0 0 1 0 1 1 ? 2 ? ? 1 1 - 0 1 0 0 1 2 0 0 ? ? ? ? ? 0 0 1 ? ? 1 ? ? ? ? ? 0 0 - 0 0 ? 1 0 0 1 ? ? ? ? ? ? ? ? 1 0 0 - 1 0 ? 0 0 1 0 1 1 0 ? 0 1 0 2 2 0 1 1 1 0 0 ? ? 1 0 0 ? 1 0 0 ? ? ? ? ? ? - - 0 0 0 0 0 0 0 0 0 0 2 1 1 0 0 0 0 ? 1 2 2 - 1 0 0 0 0 2 0 0 2 0 ? ? 0 2 2 0 1 1 0 ? 0 0 0 1 ? ? ? 0 0 0 0 0 1 0 0 ? 2 0 0 0 ? 1 ? ? ? 1 1 1 1 1 1 ? 1 0 1 ? 0 0 ? 0

***Waluchelys cavitesta***? ? ? ? ? 0 0 ? 0 0 0 ? ? ? ? ? 0 0 1 0 - 0 ? 0 - ? ? ? ? ? ? ? ? ? ? ? ? ? ? ? ? ? ? ? ? 0 ? 1 ? ? ? ? ? ? ? ? ? ? ? ? ? ? ? ? ? ? ? ? ? ? ? 1 1 0 1 ? ? ? ? 0 0 0 ? ? ? 0 ? ? 0 0 0 ? 0 ? ? ? ? ? ? 0 0 0 0 0 0 ? ? - - ? ? ? ? ? 2 ? ? ? ? ? ? ? ? ? ? ? ? ? ? 0 - ? ? ? ? ? 0 1 ? ? ? ? ? ? ? ? ? ? ? 0 ? ? ? ? ? ? 0 ? ? ? ? ? ? ? ? ? 0 ? ? 1 0 ? ? ? ? ? ? ? ? ? ? ? 1 1 1 0 {0 1} ? 1 ? ? ? - - 0 0 0 0 0 ? ? ? ? ? ? ? ? ? ? ? ? ? ? 1 ? ? 0 ? ? ? ? ? 0 0 ? - 0 0 1 ? ? ? ? ? ? ? ? ? ? 0 ? 0 ? ? ? ? ? ? ? ? ? 1 1 0 ? - ? ? ? ? ? ? ? ? ? ? ? ? ? ? ? ? ? ? 0 0

***Warkalania carinaminor***? ? ? 0 0 ? ? ? ? ? ? ? ? ? ? ? ? ? ? ? ? ? ? ? ? ? ? ? ? ? ? ? ? ? ? ? ? ? ? ? ? ? ? ? ? ? ? ? ? ? ? ? ? ? 1 1 1 0 1 - - 0 1 1 ? ? ? ? 0 ? ? ? ? ? ? ? ? ? ? ? ? ? ? ? ? ? ? ? ? ? ? ? ? ? 1 ? ? ? ? ? ? ? ? ? ? ? ? ? ? ? ? ? ? ? ? ? ? ? ? ? ? ? ? ? ? ? ? ? ? ? ? ? ? ? ? ? ? ? ? ? ? ? ? ? ? ? ? ? ? ? ? ? ? ? ? ? ? ? ? ? ? ? ? ? ? ? ? ? ? ? ? ? ? ? ? ? ? ? ? 0 ? ? ? ? ? ? ? ? ? ? ? ? ? ? ? ? ? ? ? ? ? ? ? ? ? ? ? ? ? ? ? ? ? ? ? ? ? ? ? ? ? ? ? ? ? ? ? ? ? ? 2 0 1 1 ? ? ? ? ? ? ? ? ? ? ? ? ? ? ? ? ? ? ? ? ? ? ? ? ? ? ? ? ? ? ? ? ? ? ? ? ? ? ? ? ? ?

***Xenochelys formosa***? 2 0 ? ? ? ? ? 0 0 1 ? 1 1 2 1 0 1 1 0 - 0 ? ? ? ? 1 ? ? ? ? ? ? ? ? ? ? ? ? ? ? ? ? ? ? ? ? ? 0 1 0 0 2 - - - - - - - - - - - - - - - 1 ? ? ? ? ? ? ? ? ? ? ? ? ? ? 0 1 1 1 0 0 0 0 1 ? 1 ? ? 1 - - - - - ? - ? ? ? ? ? ? 0 0 0 1 1 0 - ? ? ? ? ? 0 ? 0 2 ? 0 1 ? ? ? ? 0 1 1 0 0 ? ? ? ? 1 ? ? ? ? ? 0 2 0 0 0 0 - 3 0 1 1 - - 0 1 1 ? 1 0 ? ? ? ? ? ? 1 1 0 0 1 1 2 1 - ? ? 0 ? ? 2 ? ? ? ? - - 0 0 0 0 0 ? ? 1 0 ? 0 1 1 0 ? 0 1 0 1 2 2 - ? 0 2 0 - 0 ? ? 2 0 ? ? 0 2 ? ? ? 1 ? 0 ? ? ? ? ? ? ? ? 0 0 ? ? 1 1 0 0 2 ? ? ? 1 ? ? ? ? 1 1 1 1 1 2 0 1 ? 1 0 ? 0 1 0

***Xinjiangchelys wusu***0 1 1 2 0 0 0 ? 0 0 ? ? 0 1 1 1 0 0 1 0 - 0 1 ? ? ? 1 0 ? - - - - 1 0 ? ? - - - {0 1} ? 0 ? ? ? ? ? 0 (0 1) 0 0 2 ? ? ? 1 ? ? ? ? ? ? ? ? 0 ? ? 0 0 ? ? ? ? ? ? ? ? ? ? ? ? ? 0 1 1 1 0 0 0 0 ? ? ? 0 0 0 0 0 0 1 1 ? 1 ? ? ? 1 - 0 1 0 0 1 1 0 0 0 ? 0 0 0 0 ? 0 0 1 0 0 ? ? ? ? 0 0 - 0 0 ? ? 0 ? 1 1 0 0 0 0 0 2 ? ? 0 ? ? 3 0 1 0 1 1 1 1 ? ? 0 1 1 0 2 2 ? ? ? ? 0 ? ? 1 {1 2} 0 0 ? 1 0 ? ? 1 ? ? 1 0 - - 0 0 1 0 0 0 ? 0 0 0 0 1 1 0 ? 0 0 ? 1 1 (1 2) - 0 0 0 0 ? {1 2} ? 0 2 0 ? ? 0 2 2 0 1 1 0 ? 0 0 0 1 ? ? ? ? 0 0 0 0 ? 0 0 0 2 1 0 0 2 1 - 0 - 1 1 1 1 1 1 0 1 ? 1 ? ? ? 0 0

***Yaminuechelys maior***0 1 0 ? ? 0 1 ? 0 0 0 ? 1 1 2 3 0 0 1 1 0 0 1 ? ? ? 1 1 1 0 0 0 0 1 0 ? 1 1 3 1 1 0 1 0 2 ? ? - 0 1 0 0 2 - - - - - - - - - - - - - - - 1 1 ? ? ? ? ? 1 0 1 0 ? 0 0 ? 0 1 1 1 0 0 0 0 1 ? ? ? ? 0 0 0 ? ? ? ? ? ? 1 ? 1 - ? 1 0 1 ? ? 0 0 ? ? 0 ? ? 0 ? 1 1 1 1 0 ? 0 ? ? 1 - - 0 0 ? ? ? ? 1 ? ? ? ? ? 0 2 ? ? 0 ? ? 2 0 ? 0 ? 1 0 1 1 0 0 1 1 0 0 ? ? 0 ? ? 0 ? - ? 2 0 0 2 1 0 1 ? 1 0 ? ? ? - - 0 0 1 1 0 ? ? ? ? ? ? ? ? ? ? ? 0 ? ? 2 2 - ? ? ? ? ? 0 0 0 - 0 ? ? 0 ? ? ? ? 0 - ? ? ? ? 1 1 2 ? ? 1 0 0 0 ? ? ? 0 2 0 0 0 1 1 - 0 - 1 1 1 1 1 1 ? ? ? ? ? ? ? 1 0

***Yehguia tatsuensis***0 1 0 ? ? ? ? ? ? ? ? ? ? ? ? ? 0 0 1 1 2 0 ? ? ? ? ? ? ? ? ? ? ? ? ? ? ? ? ? ? ? ? ? ? ? ? ? - ? ? 0 0 2 ? ? ? ? ? ? ? ? ? ? ? ? ? ? ? ? ? ? ? ? ? ? ? ? ? ? ? ? ? ? 0 1 1 1 0 0 0 0 ? ? ? ? ? 0 0 0 0 1 1 ? 2 ? ? ? ? ? ? ? ? 0 1 2 0 0 ? ? ? ? ? 0 ? ? ? 1 ? ? ? ? ? ? 0 0 - 0 0 1 ? ? ? ? ? ? ? ? ? 1 ? ? ? ? ? ? 3 0 ? ? ? ? ? 1 ? ? ? ? ? ? ? ? ? ? ? ? ? ? ? ? ? 0 0 2 1 0 0 ? 1 ? ? ? ? - - 0 1 0 0 0 ? ? ? ? ? ? ? ? ? ? ? ? ? ? ? ? ? ? ? ? ? ? ? ? ? ? ? ? ? 0 ? ? ? ? ? ? ? ? ? ? ? ? ? ? ? ? ? ? ? ? ? ? 0 2 ? ? ? 1 ? ? ? ? ? ? ? ? ? ? ? ? ? ? ? ? ? 0 0

Trees and synapomorphies

Strict consensus

***
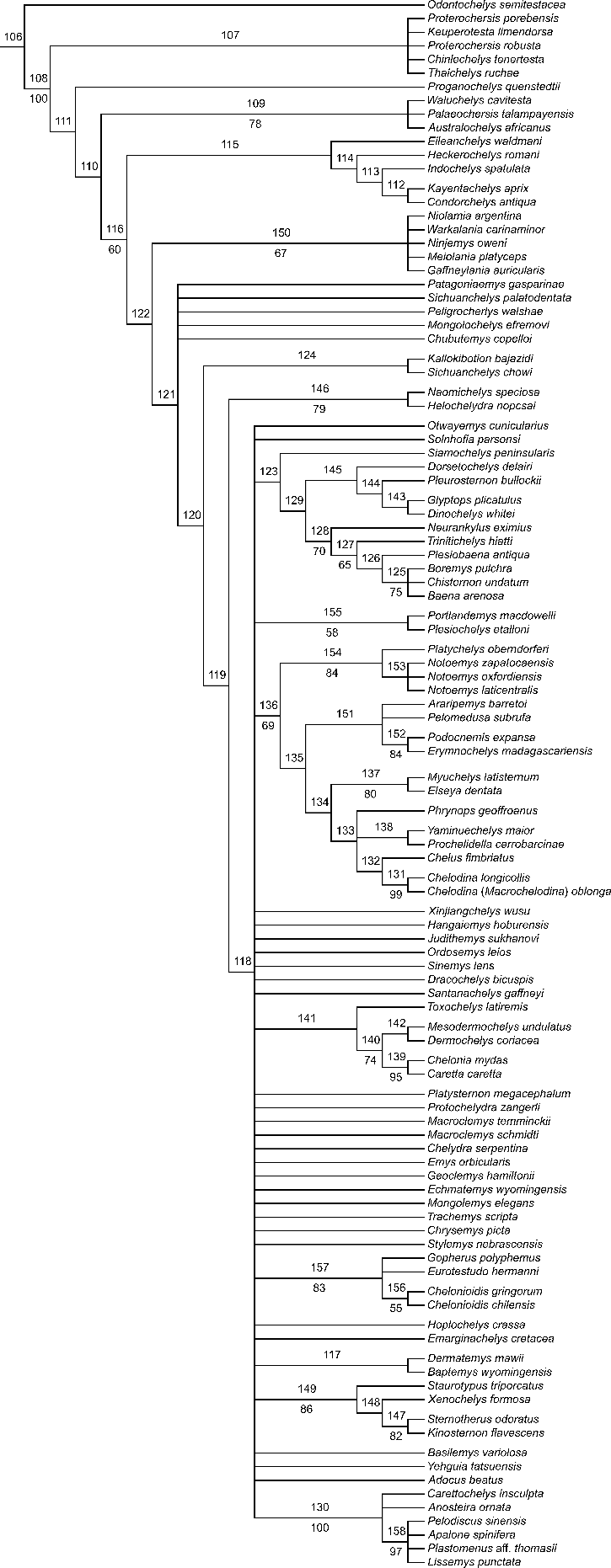
***

**Fig S1. Strict consensus tree.** Above the branches node numbers, below bootstrap values above 50.

***Odontochelys semitestacea*:**

No autapomorphies

***Adocus beatus*:**

Canalis caroticum D (12): enclosed in bone → junction of palatine artery and internal carotid artery not enclosed in bone

Canalis caroticum F (14): all ots path inside the skull → enters the skull through the foramen caroticum laterale between bs and pt

Carapace D (19): sculpturing of the shell absent → present

Cervical articulation H (28): 8)dorsal → 8(dorsal

Cervical vertebra E (37): present → biconvex cervical vertebra in the middle of the neck absent

Cranial scutes A (68): absent → present

Extragular A (96): absent → present

Foramen nervi hypoglossi (XII) (109): not covered ventrally by an extension of the pterygoid and the basioccipital → covered ventrally by an extension of the pterygoid and the basioccipital

Humeral B (116): humero-pectoral sulcus only in the hyoplastra → humero-pectoral sulcus crossing the entoplastron

Humerus A (117): only a groove → ectepicondylarforamen in a channel

Humerus D (120): lateral process seen in dorsal view → lateral process not seen in dorsal view

Hyoplastron B (125): terminates on peripheral 3 → axillary buttress terminates on peripheral 2 or 1

Ilium E (132): thelial process absent → present

Marginal A (148): marginal scales overlap onto costals absent → present

Maxilla D (153): triturating surface with only labial ridge present → labial, lingual and accessory ridges present

Maxilla E (154): accessory ridge on maxilla present all along the triturating surface → accessory ridge only in some sectors of the triturating surface

Neural A (161): neural formula 6>4<6<6<6<6 absent → present

Nuchal C (165): wider than long → longer than wide or as long as wide

Palatine A (171): palatine contribution to anterior extension of lat braincase absent → present, well-developed

Parietal F (177): not contribute to the processus trochlearis oticum → contributes to the processus trochlearis oticum

Pectoral B (181): antero-posteriorly developed → very short antero-posteriorly

Plastral scutes B (195): pronounced midline plastral sulcus sinuous absent → present

Plastron A (196): ligamentous → connection between carapace and plastron osseous

Quadrate F: incisura columella auris (232): quadrate completely rolled-up, quadrate-quadrate and/or quadrate-squamosal close to each other but not sutured → completely closed

Quadrate H (234): processustrochlearis oticum formed by a grate contribution of quadrate → small contribution of the quadrate

Squamosal A (243): squamosal-postorbital contact present → absent

Squamosal D (246): long posterior process protruding beyond condylus occipitalis absent → present

Supraoccipital C (255): horizontal ventral crest in the supraoccipital absent or poorly developed anteriorly → horizontal ventral crest present along all the crista supraoccipitalis

***Anosteira ornata*:**

No autapomorphies

***Apalone spinifera*:**

Femur A (105): rectangular to oval → articular surface of femoral head triangular in dorsal view

Maxilla D (153): triturating surface with only labial ridge present → labial and lingual ridge present

***Araripemys barretoi*:**

Abdominal A (0): present, with medial contact → present, medial contact absent

Carapace B (17): tricarinate carapace absent → present and pronounced

Carapace D (19): sculpturing of the shell absent → present

Cervical vertebra J (42): postzygapophyses not united in midline → postzygapophyses united in midline

Costal C (50): absent, costals fully or almost fully ossified, fontanelles abs or red → present

Dentary A (69): medial contact of dentaries fused → sutured only

Dorsal vertebra B (81): cylindrical, longer than wide, keeled ventrally → smooth and flat ventrally, hexagonal in shape

Entoplastron D (87): entoplastron V-shaped absent → present

Entoplastron F (89): entoplastron tightly sutured with hyoplastron yes → no

Epiplastron A (90): epiplastra and entoplastron narrow and elongate absent → present

Extragular A (96): present → absent

Gular A (112): only one scute → one pair

Hypoplastron A (127): peripheral and costal V → inguinal buttresses contact peripherals only

Hypoplastron B (128): Inguinal buttress terminates on peripheral 8 → 7

Jugal B (140): jugal participation to upper temporal rim absent → present

Maxilla B (151): involving palatine → upper triturating surface not involving palatine or its contribution is minor

Musk ducts A (157): present → absent

Nuchal emargination (166): absent or indistinct → present, excludes peripheral 1

Plastron A (196): connection between carapace and plastron osseous → ligamentous

Plastron B (197): central plastral fontanella absent → present

Pterygoid G (219): medial contact of pterygoids present → absent

***Australochelys africanus*:**

No autapomorphies

***Baena arenosa*:**

Caudal D (25): posterior caudal vertebrae procoelous or platycoelous → posterior caudal vertebrae opisthocoelous

Cranial scutes A (68): absent → present

Maxilla B (151): involving palatine → upper triturating surface not involving palatine or its contribution is minor

Mesoplastron A (155): 1 pair of meso with medial contact → 1 reduced pair

Nuchal C (165): wider than long → longer than wide or as long as wide

Parietal H (179): moderate, f.s.t. but not entire processes trochlearis exposed in dorsal view → absent or weak, foramen stapedio-temporale concealed in dorsal view

Suprapygal A (256): two elements → none

***Baptemys wyomingensis*:**

Carapace B (17): tricarinate carapace absent → present, but only slightly

Humeral B (116): humero-pectoral sulcus only in the hyoplastra → humero-pectoral sulcus crossing the entoplastron

Hypoplastron A (127): inguinal buttresses contact peripherals only → peripheral and costal V

Ilium D (131): posterior notch in acetabulum absent → present

Nuchal emargination (166): absent or indistinct → present, excludes peripheral 1

***Basilemys variolosa*:**

Carapace D (19): sculpturing of the shell absent → present

Cervical vertebra E (37): present → biconvex cervical vertebra in the middle of the neck absent

Extragular A (96): absent → present

Extragular D (98): not reaching the entoplastron → reach the entoplastron

Extragular process B (102): none, the anterolateral edge of the anterior plastral lobe even → minor, the anterolateral edge of the anterior plastral lobe gently scalloped

Humeral B (116): humero-pectoral sulcus only in the hyoplastra → humero-pectoral sulcus crossing the entoplastron

Inframarginal B (134): 3 or more → 2

Manus A (143): most digits with three elongate phalanges → most digits with two shortened phalanges

Marginal A (148): marginal scales overlap onto costals absent → present

Neural A (161): neural formula 6>4<6<6<6<6 absent → present

Nuchal C (165): wider than long → longer than wide or as long as wide

Nuchal emargination (166): absent or indistinct → present, excludes peripheral 1

Plastral scutes B (195): pronounced midline plastral sulcus sinuous absent → present

Plastron A (196): ligamentous → connection between carapace and plastron osseous

Squamosal A (243): squamosal-postorbital contact present → absent

Suprapygal A (256): two elements → one element

***Boremys pulchra*:**

Marginal B (149): serration of posterior marginal scutes in adults pronounced, rounded tips of underlying peripherals → serration of posterior marginal scutes in adults pronounced, spiky tips of underlying peripherals

Postobital-maxilla contact (199): absent, jugal forms part of the orbit → present, jugal excluded from the orbit

***Thaichelys ruchae*:**

Dorsal epiplastral process A (71): lateroventroposterior excavation present, resulting in a distinct depression lateral to the anterior part of the medial ridge on the visceral surface of the entoplastron, partially roofed dorsally by the base of the dorsal epiplastral process → lateroventroposterior excavation absent, no dorsally roofed depression in that area

***Caretta caretta*:**

Marginal B (149): weak or no serration of posterior marginal scutes in adults → serration of posterior marginal scutes in adults pronounced, spiky tips of underlying peripherals

Nuchal emargination (166): absent or indistinct → present, excludes peripheral 1

Peripheral A (187): 11 pairs → more than 11 pairs

Quadrate H (234): processustrochlearis oticum formed by a grate contribution of quadrate → small contribution of the quadrate

***Carettochelys insculpta*:**

Humerus A (117): only a groove → ectepicondylarforamen in a channel

Humerus C (119): lateral process in the proximal end of the humerus → displaced from the proximal end, located in the shaft of the humerus

Quadratojugal B (237): quadratojugal-maxilla contact absent → present

***Chelodina* (*Macrochelodina*) *oblonga*:**

Extragular process B (102): minor, the anterolateral edge of the anterior plastral lobe gently scalloped → none, the anterolateral edge of the anterior plastral lobe even

***Chelodina longicollis*:**

No autapomorphies

***Chelonia mydas*:**

Maxilla D (153): triturating surface with only labial ridge present, or labial and lingual ridge present → labial, lingual and accessory ridges present

Premaxilla E (210): distinct, medial premaxillary hook along the labial margin absent → present

Vertebral E (266): first vertebral scute subrectangular, hexagonal, or trapezoid with posterior edge roughly transverse and not significantly narrower than the anterior edge of the second vertebral scute → first vertebral scute bell-shaped but does not invade the area of the second vertebral scute

***Chelonoidis chilensis*:**

Gular edge (113): rounded at the end to straight → spiky, minor part of the scute width contributing to the spike

Vertebral C (265): on neural V → sulcus between V 3 and 4 on neural VI

***Chelonoidis gringorum*:**

No autapomorphies

***Chelus fimbriatus*:**

Carapace B (17): tricarinate carapace absent → present and pronounced

Cervical vertebra B (34): ventral keels absent or slightly developed in all vertebrae → ventral keels more developed on posterior vertebrae

Cervical vertebra C (35): cervical centrum 8<7 absent → present

Costal B (49): medial contact of up to three posterior costals present → medial contact of posterior costals absent

Costal C (50): absent, costals fully or almost fully ossified, fontanelles abs or red → present

Dorsal vertebra B (81): cylindrical, longer than wide, keeled ventrally → smooth and flat ventrally, hexagonal in shape

Dorsal vertebra C (82): small the entire length → wide all along the entire length of the thoracic vertebrae

Extragular D (98): not reaching the entoplastron → reach the entoplastron

Femur A (105): rectangular to oval → articular surface of femoral head triangular in dorsal view

Humeral B (116): humero-pectoral sulcus only in the hyoplastra → humero-pectoral sulcus crossing the entoplastron

Marginal B (149): weak or no serration of posterior marginal scutes in adults → serration of posterior marginal scutes in adults pronounced, spiky tips of underlying peripherals

Maxilla A (150): contacts each other in ventral view → do not contact each other in ventral view

Nasal A (158): present → absent

Prefrontal C (203): prefrontal-palatine contact absent → prefrontal-palatine contact present

Premaxilla B (207): fusion of premaxilla absent → present

Vomer D (271): absent → vomer-premaxilla contact present

Xiphiplastron B (275): xiphiplastra narrow absent → present

***Chelydra serpentina*:**

Abdominal A (0): present, with medial contact → present, medial contact absent

Anal A (2): only cover parts of the xiphiplastra → anteromedially overlap onto hypoplastra

Caudal D (25): posterior caudal vertebrae procoelous or platycoelous → posterior caudal vertebrae opisthocoelous

Chevron A (45): absent or poorly developed along posterior caudals → present on nearly all caudals

Cranial scutes A (68): absent → present

Diploid number A (70): 50 → 52

Dorsal vertebra B (81): cylindrical, longer than wide, keeled ventrally → smooth and flat ventrally, hexagonal in shape

Dorsal vertebra C (82): small the entire length → wide all along the entire length of the thoracic vertebrae

Entoplastron F (89): entoplastron tightly sutured with hyoplastron yes → no

Epiplastron A (90): epiplastra and entoplastron narrow and elongate absent → present

Frontal A (110): present → frontal contribution to orbit absent

Humeral B (116): humero-pectoral sulcus only in the hyoplastra → humero-pectoral sulcus crossing the entoplastron

Hyoplastron B (125): terminates on peripheral 3 → terminates on peripheral 4

Hypoplastron B (128): Inguinal buttress terminates on peripheral 8 → 7

Marginal B (149): weak or no serration of posterior marginal scutes in adults → serration of posterior marginal scutes in adults pronounced, spiky tips of underlying peripherals

Neural B (162): regular, often hexagonal, longer than wide → irregular in shape, wider than long

Nuchal B (164): elongate costiform process of nuchal absent → present, costiform process contacts peripheral 3

Plastron B (197): central plastral fontanella absent → present

Premaxilla E (210): distinct, medial premaxillary hook along the labial margin absent → present

Quadrate F: incisura columella auris (232): quadrate completely rolled-up, quadrate-quadrate and/or quadrate-squamosal close to each other but not sutured → completely closed

Suprapygal A (256): two elements → more than 2 elements

***Chinlechelys tenertesta*:**

No autapomorphies

***Chisternon undatum*:**

Cervical articulation A (27): not formed → formed

***Chrysemys picta*:**

Cervical articulation I (29): double articulation between 5th and 6th absent → present

Cervical vertebra G (39): biconcave cervical vertebra absent → present

Femur A (105): rectangular to oval → articular surface of femoral head triangular in dorsal view

Gular edge (113): rounded at the end to straight → spiky, minor part of the scute width contributing to the spike

Humerus D (120): lateral process seen in dorsal view → lateral process not seen in dorsal view

Hyoplastron A (124): axillary buttresses contact peripherals only → peripherals and first costal

Hypoplastron A (127): inguinal buttresses contact peripherals only → peripheral and costal V

Inframarginal B (134): 3 or more → 2

Maxilla B (151): involving palatine → upper triturating surface not involving palatine or its contribution is minor

Maxilla D (153): triturating surface with only labial ridge present → labial, lingual and accessory ridges present

Nuchal C (165): wider than long → longer than wide or as long as wide

Parietal F (177): not contribute to the processus trochlearis oticum → contributes to the processus trochlearis oticum

Pectoral B (181): antero-posteriorly developed → very short antero-posteriorly

Plastron A (196): ligamentous → connection between carapace and plastron osseous

Prootic A (211): dorsal exposure large → dorsal exposure reduced or absent

Pterygoid D (216): present → pterygoid-basioccipital contact absent

Squamosal A (243): squamosal-postorbital contact present → absent

Suprapygal A (256): two elements → one element

Xiphiplastron A (274): distinct anal notch absent → present

***Chubutemys copelloi*:**

Basiocccipital B (5): Deep C-shaped concavity present → Deep C-shaped concavity between basioccipital tubera absent

Basisphenoid D (10): Basisphenoid shape not triangular (pentagonal/quadrangular) → triangular

Hypoplastron B (128): Inguinal buttress terminates on peripheral 8 → 7

Musk ducts A (157): absent → present

Nuchal emargination (166): present, includes peripheral 1 → absent or indistinct

Vomer B (269): vomer-pterygoid contact in palatal view present → absent, medial contact of palatines present

***Condorchelys antiqua*:**

No autapomorphies

***Dermatemys mawii*:**

Cervical vertebra E (37): present → biconvex cervical vertebra in the middle of the neck absent

Costal B (49): medial contact of posterior costals absent → medial contact of up to three posterior costals present

Intergular A (137): absent → present

Xiphiplastron A (274): distinct anal notch absent → present

***Dermochelys coriacea*:**

Carapace A (16): partially present → absent

Costal E (52): 8 pairs or less → 9 pairs

Peripheral A (187): 11 pairs → less than 10 pairs

***Dinochelys whitei*:**

Extragular process B (102): none, the anterolateral edge of the anterior plastral lobe even → large, projected about third or more of the extragular scute area

Gular process (114): minor, the anterior edge of the anterior plastral lobe gently scalloped, or none, the anterolateral edge of the anterior plastral lobe even → large, projected about third or more of the gular scute area

Hyoplastron B (125): terminates on peripheral 3 → axillary buttress terminates on peripheral 2 or 1

Inframarginal A (133): present → absent

Vertebral D (267): Position of sulcus between vertebral 4 and 5 on the neural series → on the suprapygals

***Dorsetochelys delairi*:**

No autapomorphies

***Dracochelys bicuspis*:**

Basisphenoid B (8): paired pits on ventral surface absent → present, restricted to the basisphenoid

Canalis caroticum D (12): enclosed in bone → junction of palatine artery and internal carotid artery not enclosed in bone

Canalis caroticum F (14): all ots path inside the skull → enters the skull through the foramen caroticum laterale between bs and pt

Cervical A (26): one cervical present → cervicals absent, carapacial scutes otherwise present

Cervical articulation J (30): present → double articulation between 6th and 7th absent

Cervical articulation L (32): present → double articulation between 7th and 8th absent

Cervical rib A (33): absent → present

Cervical vertebra E (37): present → biconvex cervical vertebra in the middle of the neck absent

Cervical vertebra I (41): neural arch on 8th cervical modified with the postzygapophyses pointing anteroventrally → neural arch on 8th cervical not modified

Cervical vertebra K (43): present well developed (as tall or taller than the high of the centrum) → ventral process on cervical 8 absent

Dorsal rib A (75): extends less than a half of costal 1 → length first thoracic rib long, extends a half or more than a half of the length of costal 1

Dorsal vertebra A (80): faces strongly anteroventrally → anterior articulation of first dorsal centrum faces at most slightly anteroventrally

Entoplastron F (89): entoplastron tightly sutured with hyoplastron yes → no

Epiplastron A (90): epiplastra and entoplastron narrow and elongate absent → present

Humerus A (117): only a groove → ectepicondylarforamen in a channel

Hyoplastron B (125): terminates on peripheral 3 → axillary buttress terminates on peripheral 2 or 1

Maxilla B (151): involving palatine → upper triturating surface not involving palatine or its contribution is minor

Maxilla D (153): triturating surface with only labial ridge present → labial and lingual ridge present

Nuchal emargination (166): absent or indistinct → present, excludes peripheral 1

Pes A (189): absent → claw on 5th digit present

Plastron B (197): central plastral fontanella absent → present

Pterygoid B (213): basipt process absent and sutured articulation → basipt process present and sutured articulation

Pterygoid H (220): absent → pterygoid contribution to foramen palatinum posterius present

***Echmatemys wyomingensis*:**

Humeral B (116): humero-pectoral sulcus only in the hyoplastra → humero-pectoral sulcus crossing the entoplastron

Hyoplastron A (124): axillary buttresses contact peripherals only → peripherals and first costal

Hyoplastron B (125): terminates on peripheral 3 → axillary buttress terminates on peripheral 2 or 1

Hypoplastron A (127): inguinal buttresses contact peripherals only → peripheral and costal V

Hypoplastron B (128): inguinal buttress terminates on peripheral 8 → 7

Inframarginal B (134): 3 or more → 2

Nuchal C (165): wider than long → longer than wide or as long as wide

Plastron A (196): ligamentous → connection between carapace and plastron osseous

Xiphiplastron A (274): distinct anal notch absent → present

***Eileanchelys waldmani*:**

Antrum postoticum A (3): incipient → fully developed

Gular process (114): minor, the anterior edge of the anterior plastral lobe gently scalloped → large, projected about third or more of the gular scute area

***Elseya dentata*:**

Maxilla D (153): triturating surface with only labial ridge present → labial, lingual and accessory ridges present

***Emarginachelys cretacea*:**

Carapace B (17): tricarinate carapace absent → present, but only slightly

Ilium E (132): thelial process absent → present

Marginal B (149): weak or no serration of posterior marginal scutes in adults → serration of posterior marginal scutes in adults pronounced, spiky tips of underlying peripherals

Nuchal B (164): elongate costiform process of nuchal absent → present, costiform process contacts peripheral 3

Palatine A (171): palatine contribution to anterior extension of lat braincase absent → present, well-developed

Squamosal A (243): squamosal-postorbital contact present → absent

***Emys orbicularis*:**

Cervical vertebra G (39): biconcave cervical vertebra absent → present

Entoplastron B (85): short → size of posterior entoplastral process long

Femur A (105): rectangular to oval → articular surface of femoral head triangular in dorsal view

Frontal A (110): present → frontal contribution to orbit absent

Humeral B (116): humero-pectoral sulcus only in the hyoplastra → humero-pectoral sulcus crossing the entoplastron

Humerus D (120): lateral process seen in dorsal view → lateral process not seen in dorsal view

Hypoplastron B (128): Inguinal buttress terminates on peripheral 8 → 7

Nuchal C (165): wider than long → longer than wide or as long as wide

Parietal F (177): not contribute to the processus trochlearis oticum → contributes to the processus trochlearis oticum

Plastron C (198): plastral kinesis absent → present

Prootic A (211): dorsal exposure large → dorsal exposure reduced or absent

Pterygoid D (216): present → pterygoid-basioccipital contact absent

Suprapygal A (256): two elements → one element

***Erymnochelys madagascariensis*:**

Extragular D (98): not reaching the entoplastron → reach the entoplastron

Hyoplastron B (125): terminates on peripheral 3 → axillary buttress terminates on peripheral 2 or 1

***Eurotestudo hermanni*:**

Marginal B (149): weak or no serration of posterior marginal scutes in adults → serration of posterior marginal scutes in adults pronounced, rounded tips of underlying peripherals

Prootic A (211): dorsal exposure large → dorsal exposure reduced or absent

***Gaffneylania auricularis*:**

No autapomorphies

***Geoclemys hamiltonii*:**

Carapace B (17): tricarinate carapace absent → present, but only slightly

Cervical vertebra G (39): biconcave cervical vertebra absent → present

Epiplastron B (91): thick anterior border absent → thick anterior border

Frontal A (110): present → frontal contribution to orbit absent

Gular edge (113): rounded at the end to straight → spiky, minor part of the scute width contributing to the spike

Humeral B (116): humero-pectoral sulcus only in the hyoplastra → humero-pectoral sulcus crossing the entoplastron

Humerus D (120): lateral process seen in dorsal view → lateral process not seen in dorsal view

Hyoplastron A (124): axillary buttresses contact peripherals only → peripherals and first costal

Hypoplastron A (127): inguinal buttresses contact peripherals only → peripheral and costal V

Hypoplastron B (128): Inguinal buttress terminates on peripheral 8 → 7

Inframarginal B (134): 3 or more → 2

Jugal B (140): jugal participation to upper temporal rim absent → present

Maxilla B (151): involving palatine → upper triturating surface not involving palatine or its contribution is minor

Musk ducts A (157): absent → present

Nuchal C (165): wider than long → longer than wide or as long as wide

Parietal F (177): not contribute to the processus trochlearis oticum → contributes to the processus trochlearis oticum

Pectoral B (181): antero-posteriorly developed → very short antero-posteriorly

Plastron A (196): ligamentous → connection between carapace and plastron osseous

Prootic A (211): dorsal exposure large → dorsal exposure reduced or absent

Squamosal A (243): squamosal-postorbital contact present → absent

Vomer E (272): narrow and tall ventral crest on vomer absent → present all along the vomer

Vomer F (273): domed palate absent → present

Xiphiplastron A (274): distinct anal notch absent → present

***Glyptops plicatulus*:**

No autapomorphies

***Gopherus polyphemus*:**

Gular process (114): minor, the anterior edge of the anterior plastral lobe gently scalloped → large, projected about third or more of the gular scute area

Humerus A (117): only a groove → ectepicondylarforamen in a channel

***Hangaiemys hoburensis*:**

Basisphenoid B (8): paired pits on ventral surface absent → present, restricted to the basisphenoid

Basisphenoid D (10): triangular → Basisphenoid shape not triangular (pentagonal/quadrangular)

Canalis caroticum D (12): enclosed in bone → junction of palatine artery and internal carotid artery not enclosed in bone

Canalis caroticum F (14): all ots path inside the skull → enters the skull through the foramen caroticum laterale between bs and pt

Cervical articulation J (30): present → double articulation between 6th and 7th absent

Cervical articulation L (32): present → double articulation between 7th and 8th absent

Cervical vertebra B (34): ventral keels more developed on posterior vertebrae → ventral keels absent or slightly developed in all vertebrae

Cranial scutes A (68): absent → present

Dorsal vertebra A (80): faces strongly anteroventrally → anterior articulation of first dorsal centrum faces at most slightly anteroventrally

Epiplastron A (90): epiplastra and entoplastron narrow and elongate absent → present

Nasal A (158): absent → present

Parietal A (172): absent → parietal-squamosal contact present

Parietal H (179): strong, entire processus trochlearis exposed in dorsal view → moderate, f.s.t. but not entire processes trochlearis exposed in dorsal view

Pterygoid B (213): basipt process absent and sutured articulation → basipt process present and sutured articulation

Pterygoid G (219): medial contact of pterygoids present → absent

Pterygoid H (220): absent → pterygoid contribution to foramen palatinum posterius present

***Heckerochelys romani*:**

Entoplastron B (85): size of posterior entoplastral process long → short

Entoplastron C (86): absent → distinct posterolateral entoplastral process present

Epiplastron A (90): epiplastra and entoplastron narrow and elongate absent → present

Extragular process B (102): minor, the anterolateral edge of the anterior plastral lobe gently scalloped → none, the anterolateral edge of the anterior plastral lobe even

Gular process (114): minor, the anterior edge of the anterior plastral lobe gently scalloped → none, the anterolateral edge of the anterior plastral lobe even

Pterygoid I (221): vertical flange on lateral process absent, or present, almost all along the lateral process → reduced

***Helochelydra nopcsai*:**

No autapomorphies

***Hoplochelys crassa*:**

Abdominal A (0): present, with medial contact → present, medial contact absent

Carapace B (17): tricarinate carapace absent → present and pronounced

Epiplastron B (91): thick anterior border absent → thick anterior border

Hyoplastron B (125): terminates on peripheral 3 → axillary buttress terminates on peripheral 2 or 1

Nuchal B (164): elongate costiform process of nuchal absent → present, process crosses peripheral I to contact pe II and even III

Pectoral A (180): present → absent

Plastron A (196): ligamentous → connection between carapace and plastron osseous

***Indochelys spatulata*:**

No autapomorphies

***Judithemys sukhanovi*:**

Basioccipital A (6): tubercle absent → with two or one ventral basioccipital tubercle

Basisphenoid B (8): paired pits on ventral surface absent → present, restricted to the basisphenoid

Canalis caroticum D (12): enclosed in bone → junction of palatine artery and internal carotid artery not enclosed in bone

Canalis caroticum F (14): all ots path inside the skull → enters the skull through the foramen caroticum laterale between bs and pt

Caudal D (25): posterior caudal vertebrae procoelous or platycoelous → posterior caudal vertebrae opisthocoelous

Cervical articulation J (30): present → double articulation between 6th and 7th absent

Cervical articulation L (32): present → double articulation between 7th and 8th absent

Dorsal vertebra A (80): faces strongly anteroventrally → anterior articulation of first dorsal centrum faces at most slightly anteroventrally

Entoplastron F (89): entoplastron tightly sutured with hyoplastron yes → no

Epiplastron A (90): epiplastra and entoplastron narrow and elongate absent → present

Humerus A (117): only a groove → ectepicondylarforamen in a channel

Hyoplastron B (125): terminates on peripheral 3 → axillary buttress terminates on peripheral 2 or 1

Parietal H (179): strong, entire processus trochlearis exposed in dorsal view → moderate, f.s.t. but not entire processes trochlearis exposed in dorsal view

Pterygoid B (213): basipt process absent and sutured articulation → basipt process present and sutured articulation

Pterygoid H (220): absent → pterygoid contribution to foramen palatinum posterius present

***Jurassichelon oleronensis*:**

Cervical articulation A (27): formed → not formed

Cervical vertebra H (40): total height of centra and neural arch much shorter than the anteroposterior length of the cervical centra → total height of centra and neural arch longer than the anteroposterior length of the cervical centra

Cranial scutes A (68): absent → present

Dorsal rib A (75): extends less than a half of costal 1 → length first thoracic rib long, extends a half or more than a half of the length of costal 1

Hyoplastron A (124): axillary buttresses contact peripherals only → peripherals and first costal

Hyoplastron B (125): terminates on peripheral 3 → axillary buttress terminates on peripheral 2 or 1

Maxilla D (153): triturating surface with only labial ridge present → labial and lingual ridge present

Nasal A (158): absent → present

Parietal A (172): absent → parietal-squamosal contact present

Parietal E (176): processus inferior parietalis forming posterior margin for nerv trigemini absent → ... present

Parietal H (179): strong, entire processus trochlearis exposed in dorsal view → moderate, f.s.t. but not entire processes trochlearis exposed in dorsal view

Plastron B (197): central plastral fontanella absent → present

Prefrontal A (201): medial contact on dorsal skull roof present → medial contact on dorsal skull roof absent

Prefrontal D (204): prefrontal exposure large → reduced

Prootic A (211): dorsal exposure large → dorsal exposure reduced or absent

Pterygoid F (218): foramen palatinum posterius present → present, but open laterally

Supraoccipital A (253): protruding significantly posterior to the foramen magnum → crista occipitalis poorly developed

Vertebral B (264): vertebrals II-IV narrower or as narrow as pleurals → vertebral II-IV broader than pleurals

Vomer B (269): vomer-pterygoid contact in palatal view present → absent, medial contact of palatines present

***Kallokibotion bajazidi*:**

Cervical A (26): one cervical present → cervicals absent, carapacial scutes otherwise present

Neural A (161): neural formula 6>4<6<6<6<6 absent → present

Xiphiplastron A (274): distinct anal notch absent → present

***Kayentachelys aprix*:**

Plastron B (197): present → central plastral fontanella absent

Pterygoid A (212): absent → pterygoid teeth present

Suprapygal A (256): two elements → one element

***Keuperotesta limendorsa*:**

No autapomorphies

***Kinosternon flavescens*:**

Dorsal vertebra B (81): cylindrical, longer than wide, keeled ventrally → smooth and flat ventrally, hexagonal in shape

Musk ducts A (157): present → absent

Plastral kinesis A (192): anterior → anterior and posterior

***Lissemys punctata*:**

Foramen jugulare posterius B (108): separated by opisthotic and or exoccipital → separated from fenestra postotica by pterygoid

Hyo-hypoplastron A (122): not fused → fused

Pterygoid I (221): reduced → vertical flange on lateral process absent

***Macroclemys schmidti*:**

Basisphenoid B (8): paired pits on ventral surface absent → present, restricted to the basisphenoid

Cranial scutes A (68): absent → present

Frontal A (110): present → frontal contribution to orbit absent

Premaxilla E (210): distinct, medial premaxillary hook along the labial margin absent → present

Quadrate F: incisura columella auris (232): quadrate completely rolled-up, quadrate-quadrate and/or quadrate-squamosal close to each other but not sutured → completely closed

***Macroclemys temminckii*:**

Abdominal A (0): present, with medial contact → present, medial contact absent

Anal A (2): only cover parts of the xiphiplastra → anteromedially overlap onto hypoplastra

Caudal C (24): anterior caudal vertebrae procoelous or platycoelous → anterior caudal vertebrae opisthocoelous

Caudal D (25): posterior caudal vertebrae procoelous or platycoelous → posterior caudal vertebrae opisthocoelous

Chevron A (45): absent or poorly developed along posterior caudals → present on nearly all caudals

Cranial scutes A (68): absent → present

Diploid number A (70): 50 → 52

Dorsal vertebra C (82): small the entire length → Costo-vertebral tunnel wide anteriorly and posteriorly, or wide all along the entire length of the thoracic vertebrae

Entoplastron F (89): entoplastron tightly sutured with hyoplastron yes → no

Epiplastron A (90): epiplastra and entoplastron narrow and elongate absent → present

Frontal A (110): present → frontal contribution to orbit absent

Hyoplastron B (125): terminates on peripheral 3 → terminates on peripheral 4

Hypoplastron B (128): Inguinal buttress terminates on peripheral 8 → 7

Marginal B (149): weak or no serration of posterior marginal scutes in adults → serration of posterior marginal scutes in adults pronounced, spiky tips of underlying peripherals

Neural B (162): regular, often hexagonal, longer than wide → irregular in shape, wider than long

Nuchal B (164): elongate costiform process of nuchal absent → present, costiform process contacts peripheral 3

Nuchal emargination (166): absent or indistinct → present, excludes peripheral 1

Pes C (191): 5 digits → 4 digits

Plastron B (197): central plastral fontanella absent → present

Premaxilla E (210): distinct, medial premaxillary hook along the labial margin absent → present

Pubis B (227): cartilaginous or absent → epipubis process osseous or calcified

Quadrate F: incisura columella auris (232): quadrate completely rolled-up, quadrate-quadrate and/or quadrate-squamosal close to each other but not sutured → completely closed

Supramarginal A (252): absent → partial row present

Suprapygal A (256): two elements → one element

***Meiolania platyceps*:**

Cranial scute E (57): scutes A, B, and C forming a continuous posterolateral shelf yes → no

Cranial scute G (59): B scute a recurved horn no → yes

Cranial scute H (60): B scute in cross section triangular → round

Cranial scute M (65): Y scute pentagonal pointing posteriorly and separating the medial contact of G scutes → rectangular not separating the medial contact of G scutes

Pterygoid C2 (215): Intrapterygoid slit extensive, completely covering fcb no → yes

Tail ring B (260): closed ventrally → open ventrally

***Mesodermochelys undulatus*:**

Cervical articulation L (32): present → double articulation between 7th and 8th absent

Cervical vertebra C (35): present → cervical centrum 8<7 absent

***Mongolemys elegans*:**

Basioccipital A (6): tubercle absent → with two or one ventral basioccipital tubercle

Basisphenoid B (8): paired pits on ventral surface absent → present, restricted to the basisphenoid

Canalis caroticum D (12): enclosed in bone → junction of palatine artery and internal carotid artery not enclosed in bone

Canalis caroticum F (14): all ots path inside the skull → enters the skull through the foramen caroticum laterale between bs and pt

Cervical vertebra G (39): biconcave cervical vertebra absent → present

Chevron A (45): absent or poorly developed along posterior caudals → present on nearly all caudals

Femur A (105): rectangular to oval → articular surface of femoral head triangular in dorsal view

Hyoplastron A (124): axillary buttresses contact peripherals only → peripherals and first costal

Hyoplastron B (125): terminates on peripheral 3 → axillary buttress terminates on peripheral 2 or 1

Hypoplastron A (127): inguinal buttresses contact peripherals only → peripheral and costal V

Maxilla B (151): involving palatine → upper triturating surface not involving palatine or its contribution is minor

Maxilla D (153): triturating surface with only labial ridge present → labial, lingual and accessory ridges present

Nuchal C (165): wider than long → longer than wide or as long as wide

Plastron A (196): ligamentous → connection between carapace and plastron osseous

Pterygoid H (220): absent → pterygoid contribution to foramen palatinum posterius present

Pterygoid I (221): reduced → vertical flange on lateral process absent

***Mongolochelys efremovi*:**

Caudal C (24): anterior caudal vertebrae opisthocoelous → anterior caudal vertebrae procoelous or platycoelous

Cervical vertebra F (38): 4th → 3rd

Cranial scute D (56): yes → X scute partially separates G scales no

Epiplastron A (90): epiplastra and entoplastron narrow and elongate absent → present

Jugal-quadrate contact (141): jugal clearly not in contact with quadrate, quadratojugal broad → jugal nearly or clearly in contact with quadrate, quadratojugal reduced

Maxilla D (153): labial and lingual ridge present → labial, lingual and accessory ridges present

Nasal C (160): dorsal exposure of nasal large → greately reduced relative to that of all other elements

Neural B (162): regular, often hexagonal, longer than wide → irregular in shape, wider than long

Prootic A (211): dorsal exposure large → dorsal exposure reduced or absent

Squamosal B (244): squamosal-supraoccipital contact absent → present

***Myuchelys latisternum*:**

Basisphenoid B (8): paired pits on ventral surface absent → present, restricted to the basisphenoid

Hypoplastron B (128): Inguinal buttress terminates on peripheral 8 → 7

***Naomichelys speciosa*:**

No autapomorphies

***Neurankylus eximius*:**

No autapomorphies

***Ninjemys oweni*:**

Cranial scute D (56): yes → X scute partially separates G scales no

Cranial scute F (58): low → D scute high

Cranial scute J (62): yes → A scute small and not forming a large shelf no

***Niolamia argentina*:**

Cranial scute B (54): yes → scute D meeting in midline no

Cranial scute C (55): yes → scute X much smaller than D scute no

Cranial scute D (56): yes → X scute partially separates G scales no

Cranial scute J (62): yes → A scute small and not forming a large shelf no

Cranial scute K (63): A scute comparable in size to B scute → A scute small A scute very large

Cranial scute L (64): large → Y and Z scutes relatively larges mall

Cranial scute N (66): H scute present → absent

***Notoemys laticentralis*:**

No autapomorphies

***Notoemys oxfordiensis*:**

Hypoplastron B (128): Inguinal buttress terminates on peripheral 8 → 7

***Notoemys zapatocaensis*:**

Extragular process B (102): minor, the anterolateral edge of the anterior plastral lobe gently scalloped → large, projected about third or more of the extragular scute area

***Ordosemys leios*:**

Anal A (2): only cover parts of the xiphiplastra → anteromedially overlap onto hypoplastra

Basioccipital A (6): tubercle absent → with two or one ventral basioccipital tubercle

Basisphenoid B (8): paired pits on ventral surface absent → present, restricted to the basisphenoid

Canalis caroticum D (12): enclosed in bone → junction of palatine artery and internal carotid artery not enclosed in bone

Canalis caroticum F (14): all ots path inside the skull → enters the skull through the foramen caroticum laterale between bs and pt

Caudal D (25): posterior caudal vertebrae procoelous or platycoelous → posterior caudal vertebrae opisthocoelous

Cervical articulation J (30): present → double articulation between 6th and 7th absent

Cervical articulation L (32): present → double articulation between 7th and 8th absent

Cervical rib A (33): absent → present

Cervical vertebra C (35): present → cervical centrum 8<7 absent

Cervical vertebra G (39): biconcave cervical vertebra absent → present

Cervical vertebra I (41): neural arch on 8th cervical modified with the postzygapophyses pointing anteroventrally → neural arch on 8th cervical not modified

Dorsal rib A (75): extends less than a half of costal 1 → length first thoracic rib long, extends a half or more than a half of the length of costal 1

Dorsal vertebra A (80): faces strongly anteroventrally → anterior articulation of first dorsal centrum faces at most slightly anteroventrally

Humerus A (117): only a groove → ectepicondylarforamen in a channel

Hyoplastron B (125): terminates on peripheral 3 → axillary buttress terminates on peripheral 2 or 1

Musk ducts A (157): absent → present

Nasal A (158): absent → present

Plastron B (197): central plastral fontanella absent → present

Prefrontal A (201): medial contact on dorsal skull roof present → medial contact on dorsal skull roof absent

Pterygoid B (213): basipt process absent and sutured articulation → basipt process present and sutured articulation

Pterygoid H (220): absent → pterygoid contribution to foramen palatinum posterius present

***Otwayemys cunicularius*:**

Abdominal A (0): present, with medial contact → present, medial contact absent

Caudal C (24): anterior caudal vertebrae procoelous or platycoelous → anterior caudal vertebrae opisthocoelous

Cervical rib A (33): absent → present

Cervical vertebra G (39): biconcave cervical vertebra absent → present

Cervical vertebra H (40): total height of centra and neural arch much shorter than the anteroposterior length of the cervical centra → total height of centra and neural arch longer than the anteroposterior length of the cervical centra

Cervical vertebra I (41): neural arch on 8th cervical modified with the postzygapophyses pointing anteroventrally → neural arch on 8th cervical not modified

Chevron A (45): absent or poorly developed along posterior caudals → present on nearly all caudals

Dorsal rib A (75): extends less than a half of costal 1 → length first thoracic rib long, extends a half or more than a half of the length of costal 1

Dorsal vertebra A (80): faces strongly anteroventrally → anterior articulation of first dorsal centrum faces at most slightly anteroventrally

Extragular A (96): absent → present

Hypoplastron B (128): Inguinal buttress terminates on peripheral 8 → 6

Parietal H (179): strong, entire processus trochlearis exposed in dorsal view → absent or weak, foramen stapedio-temporale concealed in dorsal view, or moderate, f.s.t. but not entire processes trochlearis exposed in dorsal view

Plastron B (197): central plastral fontanella absent → present

Vertebral B (264): vertebrals II-IV narrower or as narrow as pleurals → vertebral II-IV broader than pleurals

***Palaeochersis talampayensis*:**

Maxilla D (153): labial and lingual ridge present → triturating surface with only labial ridge present

***Patagoniaemys gasparinae*:**

Caudal D (25): posterior caudal vertebrae opisthocoelous → posterior caudal vertebrae procoelous or platycoelous

***Peligrochelys walshae*:**

Femur A (105): rectangular to oval → articular surface of femoral head triangular in dorsal view

Neural B (162): regular, often hexagonal, longer than wide → irregular in shape, wider than long

***Pelodiscus sinensis*:**

Pterygoid H (220): absent → pterygoid contribution to foramen palatinum posterius present

***Pelomedusa subrufa*:**

Extragular process B (102): none, the anterolateral edge of the anterior plastral lobe even → minor, the anterolateral edge of the anterior plastral lobe gently scalloped

Marginal B (149): weak or no serration of posterior marginal scutes in adults → serration of posterior marginal scutes in adults pronounced, rounded tips of underlying peripherals

***Phrynops geoffroanus*:**

Basisphenoid B (8): paired pits on ventral surface absent → present, restricted to the basisphenoid

Diploid number A (70): 50 → 58

***Plastomenus* aff. *thomasii*:**

Plastron B (197): present → central plastral fontanella absent

***Platychelys oberndorferi*:**

Hyoplastron B (125): terminates on peripheral 3 → axillary buttress terminates on peripheral 2 or 1

Ilium B (130): in costals only or reaching pygal, but it does not extend onto peripherals → iliac scar extends from costals onto the peripherals and pygal

Marginal B (149): weak or no serration of posterior marginal scutes in adults → serration of posterior marginal scutes in adults pronounced, spiky tips of underlying peripherals

Supramarginal A (252): absent → partial row present

***Platysternon megacephalum*:**

Basisphenoid A (7): rostrum basisphenoidale flat → rod-like, thick, and rounded

Caudal D (25): posterior caudal vertebrae procoelous or platycoelous → posterior caudal vertebrae opisthocoelous

Cervical vertebra B (34): ventral keels more developed on posterior vertebrae → ventral keels absent or slightly developed in all vertebrae

Cervical vertebra G (39): biconcave cervical vertebra absent → present

Cervical vertebra K (43): present well developed (as tall or taller than the high of the centrum) → ventral process on cervical 8 absent

Chevron A (45): absent or poorly developed along posterior caudals → present on nearly all caudals

Diploid number A (70): 50 → 54

Dorsal vertebra B (81): cylindrical, longer than wide, keeled ventrally → smooth and flat ventrally, hexagonal in shape

Dorsal vertebra C (82): small the entire length → Costo-vertebral tunnel wide anteriorly and posteriorly

Frontal A (110): present → frontal contribution to orbit absent

Humerus A (117): only a groove → ectepicondylarforamen in a channel

Hyoplastron B (125): terminates on peripheral 3 → terminates on peripheral 4

Hypoplastron B (128): Inguinal buttress terminates on peripheral 8 → 6

Musk ducts A (157): absent → present

Neural B (162): regular, often hexagonal, longer than wide → irregular in shape, wider than long

Nuchal emargination (166): absent or indistinct → present, excludes peripheral 1

Parietal H (179): strong, entire processus trochlearis exposed in dorsal view → absent or weak, foramen stapedio-temporale concealed in dorsal view

Postobital-maxilla contact (199): absent, jugal forms part of the orbit → present, jugal excluded from the orbit

Premaxilla E (210): distinct, medial premaxillary hook along the labial margin absent → present

Quadrate F: incisura columella auris (232): quadrate completely rolled-up, quadrate-quadrate and/or quadrate-squamosal close to each other but not sutured → completely closed

Quadratojugal B (237): quadratojugal-maxilla contact absent → present

Vertebral D (267): Position of sulcus between vertebral 4 and 5 on the neural series → on the suprapygals

Xiphiplastron A (274): distinct anal notch absent → present

***Plesiobaena antiqua*:**

Cervical articulation A (27): not formed → formed

***Plesiochelys etalloni*:**

Pterygoid F (218): foramen palatinum posterius present → present, but open laterally

***Pleurosternon bullockii*:**

Cervical A (26): one cervical present → cervicals absent, carapacial scutes otherwise present

Gular A (112): one pair → only one scute

Suprapygal A (256): two elements → one element

***Podocnemis expansa*:**

Basisphenoid B (8): paired pits on ventral surface absent → present, restricted to the basisphenoid

Femur A (105): rectangular to oval → articular surface of femoral head triangular in dorsal view

Maxilla A (150): do not contact each other in ventral view → contacts each other in ventral view

Maxilla E (154): accessory ridge on maxilla present all along the triturating surface → accessory ridge only in some sectors of the triturating surface

Pterygoid H (220): pterygoid contribution to foramen palatinum posterius present → absent

***Portlandemys macdowelli*:**

No autapomorphies

***Prochelidella cerrobarcinae*:**

No autapomorphies

***Proganochelys quenstedtii*:**

Caudal A (22): absent → tail club present

Costal E (52): 8 pairs or less → 9 pairs

Extragular process C (104): dorsal surface of the projection convex → dorsal surface distinctly concave in the posterolateral part of the projection

Gular edge (113): rounded at the end to straight → spiky, distinctly conical

Humerus E (121): length of the humerus more than two times the width of the proximal end → length of the humerus two times or less than the width of the proximal end

Nuchal emargination (166): absent or indistinct → present, excludes peripheral 1, or present, includes peripheral 1

Prefrontal E (205): absent → prefrontal heavily sculptured present

Supramarginal A (252): partial row present → complete row present

Vertebral A (263): 5 → 4

***Proterochersis porebensis*:**

No autapomorphies

***Proterochersis robusta*:**

Extragular edge B (100): lateral or anterolateral tip sharp → lateral or anterolateral tip rounded

***Protochelydra zangerli*:**

Abdominal A (0): present, with medial contact → present, medial contact absent

Anal A (2): only cover parts of the xiphiplastra → anteromedially overlap onto hypoplastra

Epiplastron A (90): epiplastra and entoplastron narrow and elongate absent → present

Frontal A (110): present → frontal contribution to orbit absent

***Santanachelys gaffneyi*:**

Cervical articulation L (32): present → double articulation between 7th and 8th absent

Costal C (50): absent, costals fully or almost fully ossified, fontanelles abs or red → present

Cranial scutes A (68): absent → present

Dorsal rib A (75): extends less than a half of costal 1 → length first thoracic rib long, extends a half or more than a half of the length of costal 1

Entoplastron F (89): entoplastron tightly sutured with hyoplastron yes → no

Jugal-quadrate contact (141): jugal clearly not in contact with quadrate, quadratojugal broad → jugal nearly or clearly in contact with quadrate, quadratojugal reduced

Manus and Pes A (144): carpal and tarsal elements not flattened → flattened

Manus B (146): paddles absent → short paddles present

Maxilla D (153): triturating surface with only labial ridge present → labial and lingual ridge present

Nasal A (158): absent → present

Parietal C (174): elongated → length of anterior extension of the lateral braincase wall inter

Parietal H (179): strong, entire processus trochlearis exposed in dorsal view → absent or weak, foramen stapedio-temporale concealed in dorsal view

Plastron B (197): central plastral fontanella absent → present

Prefrontal A (201): medial contact on dorsal skull roof present → medial contact on dorsal skull roof absent

Pterygoid F (218): foramen palatinum posterius present → present, but open laterally

Suprapygal A (256): two elements → one element

Vertebral B (264): vertebrals II-IV narrower or as narrow as pleurals → vertebral II-IV broader than pleurals

Vertebral C (265): on neural V → sulcus between V 3 and 4 on neural VI

Vomer B (269): vomer-pterygoid contact in palatal view present → absent, medial contact of palatines present

***Siamochelys peninsularis*:**

Hypoplastron B (128): Inguinal buttress terminates on peripheral 8 → 7

Plastral scutes B (195): pronounced midline plastral sulcus sinuous absent → present

***Sichuanchelys chowi*:**

Marginal B (149): weak or no serration of posterior marginal scutes in adults → serration of posterior marginal scutes in adults pronounced, rounded tips of underlying peripherals

Vertebral C (265): on neural V → sulcus between V 3 and 4 on neural VI

Vertebral E (266): first vertebral scute subrectangular, hexagonal, or trapezoid with posterior edge roughly transverse and not significantly narrower than the anterior edge of the second vertebral scute → first vertebral scute bell-shaped, wide anteriorly and tapering posteriorly into a narrower, rounded median process invading the area of the wide second vertebral scute

***Sichuanchelys palatodentata*:**

Fenestra perilymphatica A (106): large → relatively small

Humerus A (117): ectepicondylarforamen in a channel → only a groove

Jugal-quadrate contact (141): jugal clearly not in contact with quadrate, quadratojugal broad → jugal nearly or clearly in contact with quadrate, quadratojugal reduced

Maxilla D (153): labial and lingual ridge present → triturating surface with only labial ridge present

Pterygoid A (212): absent → pterygoid teeth present

Pterygoid I (221): vertical flange on lateral process absent → present, almost all along the lateral process, or reduced

Pterygoid M (225): Basisphenoid and pterygoid in different levels, step between both bones → Basisphenoid and pterygoid in the same level

Vertebral E (266): first vertebral scute subrectangular, hexagonal, or trapezoid with posterior edge roughly transverse and not significantly narrower than the anterior edge of the second vertebral scute → first vertebral scute bell-shaped, wide anteriorly and tapering posteriorly into a narrower, rounded median process invading the area of the wide second vertebral scute

***Sinemys lens*:**

Basioccipital A (6): tubercle absent → with two or one ventral basioccipital tubercle

Basisphenoid B (8): paired pits on ventral surface absent → present, restricted to the basisphenoid

Canalis caroticum D (12): enclosed in bone → junction of palatine artery and internal carotid artery not enclosed in bone

Canalis caroticum F (14): all ots path inside the skull → enters the skull through the foramen caroticum laterale between bs and pt

Cervical A (26): one cervical present → cervicals absent, carapacial scutes otherwise present

Dorsal rib A (75): extends less than a half of costal 1 → length first thoracic rib long, extends a half or more than a half of the length of costal 1

Entoplastron F (89): entoplastron tightly sutured with hyoplastron yes → no

Hyoplastron B (125): terminates on peripheral 3 → axillary buttress terminates on peripheral 2 or 1

Musk ducts A (157): absent → present

Nasal A (158): absent → present

Prefrontal A (201): medial contact on dorsal skull roof present → medial contact on dorsal skull roof absent

Prefrontal D (204): prefrontal exposure large → reduced

Pterygoid D (216): present → pterygoid-basioccipital contact absent

Pterygoid H (220): absent → pterygoid contribution to foramen palatinum posterius present

***Solnhofia parsonsi*:**

Basiocccipital B (5): Deep C-shaped concavity between basioccipital tubera absent → Deep C-shaped concavity present

Carapace F (21): The width of the posterior half of carapace is the same or is slightly wider than the anterior half → pentagonal in shape, with the anterior border more or less straight and the posterior half tapering posteriorly

Caudal B (23): formed centra → all centra amphicoelous

Cervical articulation A (27): formed → not formed

Cervical vertebra B (34): ventral keels more developed on posterior vertebrae → ventral keels absent or slightly developed in all vertebrae

Cranial scutes A (68): absent → present

Entoplastron F (89): entoplastron tightly sutured with hyoplastron yes → no

Epiplastron A (90): epiplastra and entoplastron narrow and elongate absent → present

Hyoplastron B (125): terminates on peripheral 3 → axillary buttress terminates on peripheral 2 or 1

Maxilla C (152): secondary palate formed by premaxilla, maxilla, and vomer, palatines not contacting in midline absent → formed by premaxilla, maxilla, and vomer, palatines not contacting in midline present

Nasal A (158): absent → present

Parietal C (174): elongated → length of anterior extension of the lateral braincase wall inter

Parietal H (179): strong, entire processus trochlearis exposed in dorsal view → moderate, f.s.t. but not entire processes trochlearis exposed in dorsal view

Plastron B (197): central plastral fontanella absent → present

Pterygoid H (220): absent → pterygoid contribution to foramen palatinum posterius present

Pterygoid I (221): reduced → vertical flange on lateral process absent

Pterygoid L (224): like in *Kayentachelys* → like in testudinoids

Suprapygal A (256): two elements → more than 2 elements

Vertebral B (264): vertebrals II-IV narrower or as narrow as pleurals → vertebral II-IV broader than pleurals

Vomer B (269): vomer-pterygoid contact in palatal view present → absent, medial contact of palatines present

***Staurotypus triporcatus*:**

Carapace B (17): present, but only slightly → present and pronounced

Entoplastron F (89): entoplastron tightly sutured with hyoplastron yes → no

Jugal B (140): jugal participation to upper temporal rim absent → present

Pelvis B (186): two, big separated fenestra or partially separated → coalescent

***Sternotherus odoratus*:**

No autapomorphies

***Stylemys nebrascensis*:**

Humerus D (120): lateral process seen in dorsal view → lateral process not seen in dorsal view

Hyoplastron A (124): axillary buttresses contact peripherals only → peripherals and first costal

Hypoplastron A (127): inguinal buttresses contact peripherals only → peripherals, costal V, and costal VI

Inframarginal B (134): 3 or more → 2

Manus A (143): most digits with three elongate phalanges → most digits with two shortened phalanges

Pectoral B (181): antero-posteriorly developed → very short antero-posteriorly

Plastron A (196): ligamentous → connection between carapace and plastron osseous

Pubis B (227): cartilaginous or absent → epipubis process osseous or calcified

Squamosal A (243): squamosal-postorbital contact present → absent

Xiphiplastron A (274): distinct anal notch absent → present

***Toxochelys latiremis*:**

Anal A (2): only cover parts of the xiphiplastra → anteromedially overlap onto hypoplastra

Cervical articulation L (32): present → double articulation between 7th and 8th absent

Chevron A (45): absent or poorly developed along posterior caudals → present on nearly all caudals

Nuchal emargination (166): absent or indistinct → present, excludes peripheral 1

***Trachemys scripta*:**

Cervical articulation I (29): double articulation between 5th and 6th absent → present

Cervical vertebra G (39): biconcave cervical vertebra absent → present

Costal D (51): absence of alternative short and long ends in the lateral part of the costals → presence

Femur A (105): rectangular to oval → articular surface of femoral head triangular in dorsal view

Gular edge (113): rounded at the end to straight → spiky, minor part of the scute width contributing to the spike

Humerus D (120): lateral process seen in dorsal view → lateral process not seen in dorsal view

Hyoplastron A (124): axillary buttresses contact peripherals only → peripherals and first costal

Hypoplastron A (127): inguinal buttresses contact peripherals only → peripheral and costal V

Inframarginal B (134): 3 or more → 2

Marginal B (149): weak or no serration of posterior marginal scutes in adults → serration of posterior marginal scutes in adults pronounced, spiky tips of underlying peripherals

Maxilla B (151): involving palatine → upper triturating surface not involving palatine or its contribution is minor

Maxilla D (153): triturating surface with only labial ridge present → labial, lingual and accessory ridges present

Maxilla E (154): accessory ridge on maxilla present all along the triturating surface → accessory ridge only in some sectors of the triturating surface

Nuchal C (165): wider than long → longer than wide or as long as wide

Nuchal emargination (166): absent or indistinct → present, excludes peripheral 1

Parietal A (172): absent → parietal-squamosal contact present

Parietal F (177): not contribute to the processus trochlearis oticum → contributes to the processus trochlearis oticum

Plastron A (196): ligamentous → connection between carapace and plastron osseous

Prootic A (211): dorsal exposure large → dorsal exposure reduced or absent

Squamosal A (243): squamosal-postorbital contact present → absent

Suprapygal A (256): two elements → one element

Xiphiplastron A (274): distinct anal notch absent → present

***Trinitichelys hiatti*:**

No autapomorphies

***Waluchelys cavitesta*:**

Coracoid foramen A (47): length of the coracoid foramen less than half of the length of the glenoid fossa → Length of the coracoid foramen more than half of the length of the glenoid fossa

***Warkalania carinaminor*:**

No autapomorphies

***Xenochelys formosa*:**

Nuchal C (165): wider than long → longer than wide or as long as wide

Pterygoid F (218): foramen palatinum posterius present → absent

Xiphiplastron A (274): distinct anal notch absent → present

***Xinjiangchelys wusu*:**

Anal A (2): only cover parts of the xiphiplastra → anteromedially overlap onto hypoplastra

Basioccipital A (6): tubercle absent → with two or one ventral basioccipital tubercle

Canalis caroticum D (12): enclosed in bone → junction of palatine artery and internal carotid artery not enclosed in bone

Canalis caroticum F (14): all ots path inside the skull → enters the skull through the foramen caroticum laterale between bs and pt

Cervical articulation A (27): formed → not formed

Cervical vertebra B (34): ventral keels more developed on posterior vertebrae → ventral keels absent or slightly developed in all vertebrae

Cranial scutes A (68): absent → present

Extragular A (96): absent → present

Extragular process B (102): none, the anterolateral edge of the anterior plastral lobe even → minor, the anterolateral edge of the anterior plastral lobe gently scalloped

Humerus A (117): only a groove → ectepicondylarforamen in a channel

Humerus E (121): length of the humerus more than two times the width of the proximal end → length of the humerus two times or less than the width of the proximal end

Hyoplastron B (125): terminates on peripheral 3 → axillary buttress terminates on peripheral 2 or 1

Musk ducts A (157): absent → present

Nasal A (158): absent → present

Nasal B (159): nasals contact another medially along their entire length → medial contact of nasals partially or fully hindered by long anterior fl

Neural A (161): neural formula 6>4<6<6<6<6 absent → present

Nuchal emargination (166): absent or indistinct → present, excludes peripheral 1

Prefrontal A (201): medial contact on dorsal skull roof present → medial contact on dorsal skull roof absent

Pterygoid B (213): basipt process absent and sutured articulation → basipt process present and sutured articulation

Pterygoid D (216): present → pterygoid-basioccipital contact absent

***Yaminuechelys maior*:**

Carapace D (19): sculpturing of the shell absent → present

Nuchal C (165): longer than wide or as long as wide → wider than long

Nuchal emargination (166): absent or indistinct → present, excludes peripheral 1

Plastron B (197): central plastral fontanella absent → present

***Yehguia tatsuensis*:**

Carapace D (19): sculpturing of the shell absent → present

Extragular A (96): absent → present

Marginal A (148): marginal scales overlap onto costals absent → present

Plastral scutes B (195): pronounced midline plastral sulcus sinuous absent → present

Plastron A (196): ligamentous → connection between carapace and plastron osseous

Suprapygal A (256): two elements → one element

**Node 107:**

Costal E (52): 8 pairs or less → 10 pairs

Dorsal epiplastral process A (71): lateroventroposterior excavation absent, no dorsally roofed depression in that area → lateroventroposterior excavation present, resulting in a distinct depression lateral to the anterior part of the medial ridge on the visceral surface of the entoplastron, partially roofed dorsally by the base of the dorsal epiplastral process

Dorsal epiplastral process D (74): located more laterally, about halfway or more between the midline and the lateral edge of the anterior plastral lobe → located closer to the midline than to the lateral edge of the anterior plastral lobe, not extending significantly beyond the lateral extent of the gular scutes

Extragular process B (103): dorsal, anterolaterally or laterally directed ridge extending from the tip of the extragular process to the base of the dorsal epiplastral process present and distinct → absent or weak

Ilium A (129): elongated iliac neck absent → present

Intercaudal and caudal scutes (136): absent → present

Pelvis A (185): pelvis-shell attachment by ligaments → sutured

Xiphiplastron A (274): distinct anal notch absent → present

**Node 108:**

No synapomorphies

**Node 109:**

Extragular edge C (101): ventral deflection of the anterior edge absent, the process flat or convex ventrally and V- or U-shaped in cross-section → ventral deflection of the anterior edge present, the process concave ventrally and comma shaped in cross-section

Gular process (114): minor, the anterior edge of the anterior plastral lobe gently scalloped → none, the anterolateral edge of the anterior plastral lobe even

Opisthotic B (168): depressions for musculature absent → present

Parietal D (175): overhanging process of the skull roof absent → present

Peripheral bones (188): Posterior peripheral bones without internal cavity → posterior peripheral bones with internal cavity

**Node 110:**

Cervical vertebra L (44): eighth presacral vertebra has an intermediate (transitional) cervico-dorsal morphology, can be sutured to the carapace but not to the succeeding vertebrae → eighth presacral vertebra free from the carapace and succeeding vertebrae, movable

Pectoral girdle A (182): horizontal plate with a dorsal process, not triradiate, bridge closing coracoid foramen as wide or wider than the width of the coracoid foramen → horizontal plate with a dorsal process, not triradiate, bridge closing coracoid foramen narrower than the width of the coracoid foramen

Pterygoid A (212): pterygoid teeth present → absent

Pterygoid B (213): basipt process present and movable articulation → basipt process present and sutured articulation

Quadrate A (229): flooring of the cranioquadrate space absent → by pt, but pt does not cover the prootic

Quadrate B + C (230): development of the c.t. shallow, but not developed antpost → shallow, but anteroposteriorly developed

Vomer C (270): vomerine and palatine teeth present → absent

**Node 111:**

Abdominal B (1): two pairs → one pair

Cervical vertebra L (44): eighth presacral vertebra co-ossified with the carapace and succeeding vertebrae → eighth presacral vertebra has an intermediate (transitional) cervico-dorsal morphology, can be sutured to the carapace but not to the succeeding vertebrae

Coracoid (46): flat, sub-ovoid or with rounded posteromedial edge (bee-wing shaped) → flat, rectangular or with a distinctly angular posteromedial edge

Femur A (105): articular surface of femoral head triangular in dorsal view → rectangular to oval

Mesoplastron A (155): 2 pairs of meso with medial contact → 1 pair of meso with medial contact

**Node 112:**

Costal E (52): 8 pairs or less → 9 pairs

**Node 113:**

Entoplastron A (84): absent → anterior entoplastral process present

**Node 114:**

Extragular process B (102): large, projected about third or more of the extragular scute area → minor, the anterolateral edge of the anterior plastral lobe gently scalloped

**Node 115:**

Basisphenoid B (8): paired pits on ventral surface absent → present, restricted to the basisphenoid

Frontal A (110): frontal contribution to orbit absent → present

Neural B (162): regular, often hexagonal, longer than wide → irregular in shape, wider than long

Prefrontal D (204): prefrontal exposure large → reduced

Vomer F (273): present → domed palate absent

**Node 116:**

Coracoid (46): flat, rectangular or with a distinctly angular posteromedial edge → columnar, at least at its base

Dorsal epiplastral process D (74): located more laterally, about halfway or more between the midline and the lateral edge of the anterior plastral lobe → located closer to the midline than to the lateral edge of the anterior plastral lobe, not extending significantly beyond the lateral extent of the gular scutes

Entoplastron A (84): anterior entoplastral process present → absent

Extragular edge B (100): lateral or anterolateral tip sharp → lateral or anterolateral tip rounded

Extragular process B (103): dorsal, anterolaterally or laterally directed ridge extending from the tip of the extragular process to the base of the dorsal epiplastral process present and distinct → absent or weak

Hypoischium A (126): present → absent

Ilium A (129): elongated iliac neck absent → present

Lacrimal A (142): present → absent

Opisthotic D2 (170): processusinterfenestralis, present, robust, not reaching the floor of cavum a-j → present, small, reaching the floor of cavum a-j

Pectoral girdle A (182): horizontal plate with a dorsal process, not triradiate, bridge closing coracoid foramen narrower than the width of the coracoid foramen → trirradiate, bridge inexistent

Pelvic girdle (184): ischium not covered ventrally by the plastron, ischium seen in ventral view → ischium covered ventrally by the plastron

Plastron A (196): connection between carapace and plastron osseous → ligamentous

Plastron B (197): central plastral fontanella absent → present

Premaxilla A (206): external nares divided → united

Pygal notch (228): present → absent

Quadrate B + C (230): shallow, but anteroposteriorly developed → deep and anteroposteriorly developed

Recessus scalae tympani A (239): almost inexistent, not surrounded by bone → well developed

Supramarginal A (252): partial row present → absent

Vomer A (268): paired → single

**Node 117:**

Hyoplastron A (124): axillary buttresses contact peripherals only → peripherals and first costal

Maxilla B (151): involving palatine → upper triturating surface not involving palatine or its contribution is minor

Maxilla D (153): triturating surface with only labial ridge present → labial, lingual and accessory ridges present

Palatine A (171): palatine contribution to anterior extension of lat braincase absent → present, well-developed

Pectoral A (180): present → absent

Plastron A (196): ligamentous → connection between carapace and plastron osseous

Squamosal A (243): squamosal-postorbital contact present → absent

Stapedial artery B (249): relatively large → absent

**Node 118:**

Basiocccipital B (5): Deep C-shaped concavity present → Deep C-shaped concavity between basioccipital tubera absent

Basioccipital A (6): with two or one ventral basioccipital tubercle → tubercle absent

Basisphenoid D (10): Basisphenoid shape not triangular (pentagonal/quadrangular) → triangular

Cervical articulation J (30): double articulation between 6th and 7th absent → present

Cervical articulation L (32): double articulation between 7th and 8th absent → present

Cervical rib A (33): present → absent

Cervical vertebra B (34): ventral keels absent or slightly developed in all vertebrae → ventral keels more developed on posterior vertebrae

Cervical vertebra H (40): total height of centra and neural arch longer than the anteroposterior length of the cervical centra → total height of centra and neural arch much shorter than the anteroposterior length of the cervical centra

Chevron A (45): present on nearly all caudals → absent or poorly developed along posterior caudals

Dorsal rib A (75): length first thoracic rib long, extends a half or more than a half of the length of costal 1 → extends less than a half of costal 1

Humerus A (117): ectepicondylarforamen in a channel → only a groove

Maxilla B (151): upper triturating surface not involving palatine or its contribution is minor → involving palatine

Mesoplastron A (155): 1 pair of meso with medial contact → absent

Nasal A (158): present → absent

Opisthotic C (169): present, with an incipient enclosed middle ear region → present, but modified with a enclosed middle ear region

Parietal A (172): parietal-squamosal contact present → absent

Parietal H (179): absent or weak, foramen stapedio-temporale concealed in dorsal view → strong, entire processus trochlearis exposed in dorsal view

Pes A (189): claw on 5th digit present → absent

Prefrontal A (201): medial contact on dorsal skull roof absent → medial contact on dorsal skull roof present

Pterygoid I (221): vertical flange on lateral process absent → reduced

Scapula A (241): lamina between the dorsal process of the scapula and the acromion reduced: *Kallokibotion* → lamina between the dorsal process of the scapula and the acromion absent

Vertebral B (264): vertebral II-IV broader than pleurals → vertebrals II-IV narrower or as narrow as pleurals

**Node 119:**

Canalis caroticum D (12): junction of palatine artery and internal carotid artery not enclosed in bone → enclosed in bone

Canalis caroticum E (13): canalis carotici interni posterior to bifurcation in ac and ap not covered ventrally by bone → covered ventrally by bone

Canalis caroticum F (14): enters the skull through the foramen caroticum laterale between bs and pt → all ots path inside the skull

Canalis caroticum G (15): fpcci (entrance of internal carotid artery into the skull) absent → formed by pterygoid

Cranial scutes A (68): present → absent

Maxilla D (153): labial and lingual ridge present → triturating surface with only labial ridge present

Nasal C (160): dorsal exposure of nasal large → greately reduced relative to that of all other elements

Pterygoid B (213): basipt process present and sutured articulation → basipt process absent and sutured articulation

Pterygoid M (225): Basisphenoid and pterygoid in different levels, step between both bones → Basisphenoid and pterygoid in the same level

**Node 120:**

Cervical vertebra C (35): cervical centrum 8<7 absent → present

Dorsal rib C (77): last dorsal rib long, contacting peripherals → last dorsal rib short

Entoplastron B (85): size of posterior entoplastral process long → short

Epipterygoid A (93): present, rod-like → present, laminar

Extragular process B (102): minor, the anterolateral edge of the anterior plastral lobe gently scalloped → none, the anterolateral edge of the anterior plastral lobe even

Pterygoid D (216): pterygoid-basioccipital contact absent → present

Quadrate A (229): by pt, but pt does not cover the prootic → by pt

**Node 121:**

Antrum postoticum A (3): antrum postoticum absent, or incipient → fully developed

Canalis caroticum F (14): Arteria palatina enters the skull through the interpterygoid vacuity or intrapterygoid slit → enters the skull through the foramen caroticum laterale between bs and pt

Extragular process B (102): large, projected about third or more of the extragular scute area → minor, the anterolateral edge of the anterior plastral lobe gently scalloped

Manus A (143): most digits with two shortened phalanges → most digits with three elongate phalanges

Nuchal emargination (166): absent or indistinct → present, includes peripheral 1

Scapula A (241): lamina between the dorsal process of the scapula and the acromion well developed → lamina between the dorsal process of the scapula and the acromion reduced: *Kallokibotion*

**Node 122:**

Caudal B (23): all centra amphicoelous → formed centra

Cervical articulation A (27): not formed → formed

Parietal B (173): parietal contact with pt, epipt, and/or palatine absent → present

Quadrate F: incisura columella auris (232): widely open, open all along its length, quadrate not completely rolled-up → quadrate completely rolled-up, quadrate-quadrate and/or quadrate-squamosal close to each other but not sutured

Quadrate G (233): processus trochlearis oticum absent → present

Supraoccipital A (253): crista occipitalis poorly developed → protruding significantly posterior to the foramen magnum

Vomer E (272): narrow and tall ventral crest on vomer absent → present all along the vomer

**Node 123:**

Carapace D (19): sculpturing of the shell absent → present

Carapace E (20): like in trionychians → like in *Pleurosternon*

Extragular A (96): absent → present

Mesoplastron A (155): absent → 1 pair of meso with medial contact

**Node 124:**

Plastron A (196): ligamentous → connection between carapace and plastron osseous

**Node 125:**

Cervical A (26): one cervical present → more than one cervical present

Extragular process B (102): none, the anterolateral edge of the anterior plastral lobe even → minor, the anterolateral edge of the anterior plastral lobe gently scalloped

Gular process (114): none, the anterolateral edge of the anterior plastral lobe even → minor, the anterior edge of the anterior plastral lobe gently scalloped

Maxilla D (153): triturating surface with only labial ridge present → labial and lingual ridge present

**Node 126:**

Basiocccipital B (5): Deep C-shaped concavity between basioccipital tubera absent → Deep C-shaped concavity present

Extragular B (97): medial contact of extragulars absent → present, contacting one another posterior to gulars

**Node 127:**

Extragular D (98): not reaching the entoplastron → reach the entoplastron

Prefrontal D (204): reduced → absent or near absent

**Node 128:**

Epipterygoid A (93): present, laminar → absent

Marginal B (149): weak or no serration of posterior marginal scutes in adults → serration of posterior marginal scutes in adults pronounced, rounded tips of underlying peripherals

**Node 129:**

Hypoplastron A (127): inguinal buttresses contact peripherals only → peripheral and costal V

Plastron A (196): ligamentous → connection between carapace and plastron osseous

**Node 130:**

Carapace A (16): carapacial scutes present → partially present

Carapace D (19): sculpturing of the shell absent → present

Cervical vertebra B (34): ventral keels more developed on posterior vertebrae → ventral keels absent or slightly developed in all vertebrae

Cervical vertebra E (37): present → biconvex cervical vertebra in the middle of the neck absent

Cervical vertebra K (43): present well developed (as tall or taller than the high of the centrum) → ventral process on cervical 8 absent

Costal B (49): medial contact of posterior costals absent → medial contact of up to three posterior costals present

Diploid number A (70): 50 → 66 o 68

Entoplastron F (89): entoplastron tightly sutured with hyoplastron yes → no

Foramen nervi hypoglossi (XII) (109): not covered ventrally by an extension of the pterygoid and the basioccipital → covered ventrally by an extension of the pterygoid and the basioccipital

Hyoplastron B (125): terminates on peripheral 3 → terminates on peripheral 4

Hypoplastron B (128): Inguinal buttress terminates on peripheral 8 → 7

Ischium A (138): with lateral processes present → with lateral processes absent

Manus C (147): flippers absent → short flippers present, or elongate flippers present

Maxilla B (151): involving palatine → upper triturating surface not involving palatine or its contribution is minor

Palatine A (171): palatine contribution to anterior extension of lat braincase absent → present, well-developed

Parietal F (177): not contribute to the processus trochlearis oticum → contributes to the processus trochlearis oticum

Pelvis B (186): two, big separated fenestra or partially separated → coalescent

Peripheral A (187): 11 pairs → 10 pairs

Plastral scutes A (194): present → absent

Prefrontal C (203): prefrontal-palatine contact present → prefrontal-palatine contact absent

Premaxilla B (207): fusion of premaxilla absent → present

Premaxilla C (208): foramen praepalatinum present → absent, foramen intermaxillaris present

Pterygoid G (219): medial contact of pterygoids present → absent

Pterygoid L (224): like in *Kayentachelys* → like in testudinoids

Pubis A (226): lateral process small, poorly developed, columnar → lateral process well developed and flat

Quadrate F: incisura columella auris (232): quadrate completely rolled-up, quadrate-quadrate and/or quadrate-squamosal close to each other but not sutured → completely closed

Quadrate H (234): processustrochlearis oticum formed by a grate contribution of quadrate → small contribution of the quadrate

Squamosal A (243): squamosal-postorbital contact present → absent

Squamosal D (246): long posterior process protruding beyond condylus occipitalis absent → present

Supraoccipital C (255): horizontal ventral crest in the supraoccipital absent or poorly developed anteriorly → horizontal ventral crest present along all the crista supraoccipitalis

Suprapygal A (256): two elements → none, or one element

Vomer B (269): vomer-pterygoid contact in palatal view present → absent, medial contact of palatines present

Vomer D (271): vomer-premaxilla contact present → absent

**Node 131:**

Carapace D (19): sculpturing of the shell absent → present

Costal A (48): medial contact of costal I absent → present

Costal B (49): medial contact of up to three posterior costals present → medial contact of all costals present

Entoplastral scute (83): absent → present

Frontal B (111): not fused → fused

Humerus B (118): shoulder present → shoulder absent: pleurodires

Hypoplastron A (127): peripheral and costal V → inguinal buttresses contact peripherals only

Parietal A (172): parietal-squamosal contact present → absent

Quadrate I (235): Quadrate-basisphenoid contact absent → present

**Node 132:**

Hypoplastron B (128): Inguinal buttress terminates on peripheral 8 → 7

Neural B (162): regular, often hexagonal, longer than wide → irregular in shape, wider than long

**Node 133:**

Dentary A (69): medial contact of dentaries fused → sutured only

Exoccipital A (95): medial contact of exoccipitals dorsal to foramen magnum absent → present

Extragular process B (102): none, the anterolateral edge of the anterior plastral lobe even → minor, the anterolateral edge of the anterior plastral lobe gently scalloped

Foramen nervi hypoglossi (XII) (109): not covered ventrally by an extension of the pterygoid and the basioccipital → covered ventrally by an extension of the bo

Supraoccipital A (253): protruding significantly posterior to the foramen magnum → crista occipitalis poorly developed

**Node 134:**

Maxilla A (150): do not contact each other in ventral view → contacts each other in ventral view

Nasal A (158): absent → present

Parietal A (172): absent → parietal-squamosal contact present

Prefrontal A (201): medial contact on dorsal skull roof present → medial contact on dorsal skull roof absent

Prefrontal D (204): prefrontal exposure large → reduced

Quadratojugal A (236): present → absent, due to the presence of a deep lower temporal emargination

**Node 135:**

Cervical articulation J (30): present → double articulation between 6th and 7th absent

Cervical articulation L (32): present → double articulation between 7th and 8th absent

Costal B (49): medial contact of posterior costals absent → medial contact of up to three posterior costals present

Nuchal C (165): wider than long → longer than wide or as long as wide

Pelvis B (186): two, big separated fenestra or partially separated → coalescent

Stapedial artery C (250): foramen stapedio-temporalis located in the dorsal part of the otic region and points dorsally → located in the anterior wall of the otic region and points anteriorly

Suprapygal A (256): two elements → one element

**Node 136:**

Basisphenoid D (10): triangular → Basisphenoid shape not triangular (pentagonal/quadrangular)

Canalis caroticum G (15): formed by pterygoid → formed by pro, pro and bs, or pro and pt

Cervical vertebra B (34): ventral keels more developed on posterior vertebrae → ventral keels absent or slightly developed in all vertebrae

Cervical vertebra D (36): triangular diapophyses absent → triangular diapophyses present

Cervical vertebra I (41): neural arch on 8th cervical modified with the postzygapophyses pointing anteroventrally → neural arch on 8th cervical not modified

Cervical vertebra K (43): present well developed (as tall or taller than the high of the centrum) → ventral process on cervical 8 absent

Dorsal vertebra A (80): faces strongly anteroventrally → anterior articulation of first dorsal centrum faces at most slightly anteroventrally

Extragular A (96): absent → present

Gular A (112): one pair → only one scute

Hyoplastron A (124): axillary buttresses contact peripherals only → peripherals and first costal

Inframarginal A (133): present → absent

Opisthotic C (169): present, but modified with a enclosed middle ear region → ventral ridge on opisthotic absent

Pelvis A (185): pelvis-shell attachment by ligaments → sutured

Plastron A (196): ligamentous → connection between carapace and plastron osseous

Pterygoid D (216): present → pterygoid-basioccipital contact absent

Quadrate A (229): by pt → by qu and pro

Quadrate G (233): present → processus trochlearis oticum absent

Xiphiplastron A (274): distinct anal notch absent → present

**Node 137:**

Costal A (48): medial contact of costal I absent → present

Costal B (49): medial contact of up to three posterior costals present → medial contact of all costals present

Hyoplastron B (125): terminates on peripheral 3 → axillary buttress terminates on peripheral 2 or 1

Marginal B (149): weak or no serration of posterior marginal scutes in adults → serration of posterior marginal scutes in adults pronounced, spiky tips of underlying peripherals

Parietal H (179): strong, entire processus trochlearis exposed in dorsal view → absent or weak, foramen stapedio-temporale concealed in dorsal view

**Node 138:**

Mesoplastron A (155): absent → 1 reduced pair

Plastron A (196): connection between carapace and plastron osseous → ligamentous

**Node 139:**

Basiocccipital B (5): Deep C-shaped concavity between basioccipital tubera absent → Deep C-shaped concavity present

Carapace F (21): The width of the posterior half of carapace is the same or is slightly wider than the anterior half → pentagonal in shape, with the anterior border more or less straight and the posterior half tapering posteriorly

Cervical vertebra L (44): eighth presacral vertebra free from the carapace and succeeding vertebrae, movable → eighth presacral vertebra has an intermediate (transitional) cervico-dorsal morphology, can be sutured to the carapace but not to the succeeding vertebrae

Cranial scutes A (68): absent → present

Maxilla C (152): secondary palate formed by premaxilla, maxilla, and vomer, palatines not contacting in midline absent → formed by premaxilla, maxilla, and vomer, palatines not contacting in midline present

Pelvis B (186): two, big separated fenestra or partially separated → coalescent

Premaxilla C (208): foramen praepalatinum present → absent, premaxillae well-ossified

Vomer D (271): vomer-premaxilla contact present → absent

**Node 140:**

Antrum postoticum A (3): fully developed → incipient

Basisphenoid A (7): rostrum basisphenoidale flat → rod-like, thick, and rounded

Manus B (146): short paddles present → elongate paddles present

Parietal A (172): absent → parietal-squamosal contact present

Parietal H (179): moderate, f.s.t. but not entire processes trochlearis exposed in dorsal view → absent or weak, foramen stapedio-temporale concealed in dorsal view

Pterygoid F (218): foramen palatinum posterius present → absent

Pterygoid I (221): reduced → vertical flange on lateral process absent

Pterygoid L (224): like in *Kayentachelys* → like in testudinoids

**Node 141:**

Costal C (50): absent, costals fully or almost fully ossified, fontanelles abs or red → present

Entoplastron F (89): entoplastron tightly sutured with hyoplastron yes → no

Epiplastron A (90): epiplastra and entoplastron narrow and elongate absent → present

Hyoplastron B (125): terminates on peripheral 3 → terminates on peripheral 4

Manus and Pes A (144): carpal and tarsal elements not flattened → flattened

Manus B (146): paddles absent → short paddles present

Nuchal A (163): articulation absent → cervical articulates with nuchal along a raised pedestal

Parietal H (179): strong, entire processus trochlearis exposed in dorsal view → moderate, f.s.t. but not entire processes trochlearis exposed in dorsal view

Plastron B (197): central plastral fontanella absent → present

Squamosal E (247): Qu-Sq contact tightly sutured → wide open

Xiphiplastron B (275): xiphiplastra narrow absent → present

**Node 142:**

Carapace A (16): carapacial scutes present → partially present

Cervical vertebra H (40): total height of centra and neural arch much shorter than the anteroposterior length of the cervical centra → total height of centra and neural arch longer than the anteroposterior length of the cervical centra

Plastral scutes A (194): present → absent

**Node 143:**

Premaxilla E (210): distinct, medial premaxillary hook along the labial margin absent → present

**Node 144:**

Canalis caroticum D (12): enclosed in bone → junction of palatine artery and internal carotid artery not enclosed in bone

Canalis caroticum E (13): covered ventrally by bone → canalis carotici interni posterior to bifurcation in ac and ap not covered ventrally by bone

Canalis caroticum F (14): all ots path inside the skull → enters the skull through the foramen caroticum laterale between bs and pt

Nasal B (159): nasals contact another medially along their entire length → medial contact of nasals partially or fully hindered by long anterior fl

Postobital-maxilla contact (199): absent, jugal forms part of the orbit → present, jugal excluded from the orbit

Pterygoid B (213): basipt process absent and sutured articulation → basipt process present and sutured articulation

Pterygoid G (219): medial contact of pterygoids present → absent

**Node 145:**

Basioccipital A (6): tubercle absent → with two or one ventral basioccipital tubercle

Basisphenoid B (8): paired pits on ventral surface absent → present, restricted to the basisphenoid

Basisphenoid D (10): triangular → Basisphenoid shape not triangular (pentagonal/quadrangular)

Parietal A (172): absent → parietal-squamosal contact present

Parietal H (179): moderate, f.s.t. but not entire processes trochlearis exposed in dorsal view → absent or weak, foramen stapedio-temporale concealed in dorsal view

**Node 146:**

Basisphenoid B (8): paired pits on ventral surface absent → present in in the basisphenoid and pterygoid or in the pterygoid

Carapace D (19): sculpturing of the shell absent → present

Postobital-maxilla contact (199): absent, jugal forms part of the orbit → present, jugal excluded from the orbit

Prefrontal D (204): prefrontal exposure large → reduced

**Node 147:**

Costal A (48): medial contact of costal I absent → present

Entoplastron E (88): present → absent

Humeral A (115): 1 pair → 2 pair subdivided by a plastral hinge

Intergular A (137): absent → present

**Node 148:**

Vertebral E (266): first vertebral scute subrectangular, hexagonal, or trapezoid with posterior edge roughly transverse and not significantly narrower than the anterior edge of the second vertebral scute → first vertebral scute bell-shaped but does not invade the area of the second vertebral scute

**Node 149:**

Abdominal B (1): one pair → absent

Canalis caroticum C (11): pattern A → pattern B

Carapace B (17): tricarinate carapace absent → present, but only slightly

Cervical vertebra F (38): 4th → 3rd

Diploid number A (70): 50 → 56

Dorsal rib B (76): contact of the two last dorsal rib pairs with costals present → absent

Dorsal rib E (79): 10 pairs → 9 pairs or less

Frontal A (110): present → frontal contribution to orbit absent

Humerus D (120): lateral process seen in dorsal view → lateral process not seen in dorsal view

Hyoplastron B (125): terminates on peripheral 3 → terminates on peripheral 4

Hypoplastron B (128): Inguinal buttress terminates on peripheral 8 → 7

Ilium D (131): posterior notch in acetabulum absent → present

Ilium E (132): thelial process absent → present

Inframarginal B (134): 3 or more → 2

Inframarginal C (135): axillar and inguinal not in contact → axillar and inguinal in contact

Ischium A (138): with lateral processes present → with lateral processes absent

Maxilla B (151): involving palatine → upper triturating surface not involving palatine or its contribution is minor

Musk ducts A (157): absent → present

Nuchal B (164): elongate costiform process of nuchal absent → present, process crosses peripheral I to contact pe II and even III

Palatine A (171): palatine contribution to anterior extension of lat braincase absent → present, well-developed

Parietal F (177): not contribute to the processus trochlearis oticum → contributes to the processus trochlearis oticum

Pectoral A (180): present → absent

Peripheral A (187): 11 pairs → 10 pairs

Plastron A (196): ligamentous → connection between carapace and plastron osseous

Pterygoid I (221): reduced → vertical flange on lateral process absent

Pubis A (226): lateral process small, poorly developed, columnar → lateral process well developed and flat

Quadrate F: incisura columella auris (232): quadrate completely rolled-up, quadrate-quadrate and/or quadrate-squamosal close to each other but not sutured → completely closed

Quadrate H (234): processustrochlearis oticum formed by a grate contribution of quadrate → small contribution of the quadrate

Quadratojugal B (237): quadratojugal-maxilla contact absent → present

Squamosal A (243): squamosal-postorbital contact present → absent

Stapedial artery B (249): relatively large → significantly reduced in size

Suprapygal A (256): two elements → one element

**Node 150:**

Canalis caroticum E (13): canalis carotici interni posterior to bifurcation in ac and ap not covered ventrally by bone → covered ventrally by bone

Canalis caroticum G (15): fpcci (entrance of internal carotid artery into the skull) absent → formed by pterygoid

Caudal A (22): absent → tail club present

Cranial scute O (67): scale F formed by several scales → scale F formed by only one scale

Eustachian tube (94): not enclosed in bone → enclosed in bone

Humerus E (121): length of the humerus more than two times the width of the proximal end → length of the humerus two times or less than the width of the proximal end

Pterygoid B (213): basipt process present and sutured articulation → basipt process absent and sutured articulation

Pterygoid D (216): pterygoid-basioccipital contact absent → present

Pterygoid L (224): processuspterygoideus externus like in *Proganochelys* → like in testudinoids

Quadrate A (229): by pt, but pt does not cover the prootic → by pt

Quadratojugal C (238): quadratojugal-squamosal contact below cavum tympani absent → present

Squamosal B (244): squamosal-supraoccipital contact absent → present

Squamosal C (245): posterolateral protuberances developing horns absent, or small protuberances → big protuberances developed as horns

Supraoccipital B (254): large supraoccipital exposure to dorsal skull roof absent → present

Tail ring A (259): absent → present

**Node 151:**

Cranial scutes A (68): absent → present

Diploid number A (70): 50 → 28-34

Quadrate D (231): precolumellar fossa absent → large and deep

Vomer A (268): single → single, greatly reduced

Vomer B (269): vomer-pterygoid contact in palatal view present → absent, medial contact of palatines present

**Node 152:**

Humeral B (116): humero-pectoral sulcus only in the hyoplastra → humero-pectoral sulcus crossing the entoplastron

Parietal H (179): strong, entire processus trochlearis exposed in dorsal view → absent or weak, foramen stapedio-temporale concealed in dorsal view

Pterygoid K (223): Fossa podocnemidoidea absent → present

Quadrate I (235): Quadrate-basisphenoid contact absent → present

**Node 153:**

Extragular process B (102): none, the anterolateral edge of the anterior plastral lobe even → minor, the anterolateral edge of the anterior plastral lobe gently scalloped

**Node 154:**

Carapace F (21): The width of the posterior half of carapace is the same or is slightly wider than the anterior half → pentagonal in shape, with the anterior border more or less straight and the posterior half tapering posteriorly

Dorsal rib A (75): extends less than a half of costal 1 → length first thoracic rib long, extends a half or more than a half of the length of costal 1

Dorsal rib D (78): articulation tubercule on the anterior face of the first thoracic rib absent, smooth anterior face → present

Dorsal vertebra B (81): cylindrical, longer than wide, keeled ventrally → smooth and flat ventrally, hexagonal in shape

Dorsal vertebra C (82): small the entire length → wide all along the entire length of the thoracic vertebrae

Extragular D (98): not reaching the entoplastron → reach the entoplastron

Neural B (162): regular, often hexagonal, longer than wide → irregular in shape, wider than long

Plastron B (197): central plastral fontanella absent → present

Vertebral B (264): vertebrals II-IV narrower or as narrow as pleurals → vertebral II-IV broader than pleurals

Vertebral C (265): on neural V → sulcus between V 3 and 4 on neural VI

**Node 155:**

Basisphenoid B (8): paired pits on ventral surface absent → present, restricted to the basisphenoid

Cranial scutes A (68): absent → present

Maxilla D (153): triturating surface with only labial ridge present → labial and lingual ridge present

Nasal A (158): absent → present

Opisthotic C (169): present, but modified with a enclosed middle ear region → present, with an incipient enclosed middle ear region

Parietal C (174): elongated → length of anterior extension of the lateral braincase wall inter

Parietal E (176): processus inferior parietalis forming posterior margin for nerv trigemini absent → ... present

Parietal H (179): strong, entire processus trochlearis exposed in dorsal view → moderate, f.s.t. but not entire processes trochlearis exposed in dorsal view

Pterygoid H (220): absent → pterygoid contribution to foramen palatinum posterius present

**Node 156:**

Cervical A (26): one cervical present → cervicals absent, carapacial scutes otherwise present

**Node 157:**

Cervical vertebra G (39): biconcave cervical vertebra absent → present

Costal D (51): absence of alternative short and long ends in the lateral part of the costals → presence

Diploid number A (70): 50 → 52

Epiplastron B (91): thick anterior border absent → thick anterior border

Humerus D (120): lateral process seen in dorsal view → lateral process not seen in dorsal view

Hyoplastron B (125): terminates on peripheral 3 → axillary buttress terminates on peripheral 2 or 1

Inframarginal B (134): 3 or more → 2

Manus A (143): most digits with three elongate phalanges → most digits with two shortened phalanges

Maxilla B (151): involving palatine → upper triturating surface not involving palatine or its contribution is minor

Maxilla D (153): triturating surface with only labial ridge present → labial, lingual and accessory ridges present

Neural B (162): regular, often hexagonal, longer than wide → irregular in shape, wider than long

Nuchal C (165): wider than long → longer than wide or as long as wide

Parietal F (177): not contribute to the processus trochlearis oticum → contributes to the processus trochlearis oticum

Pectoral B (181): antero-posteriorly developed → very short antero-posteriorly

Pes C (191): 5 digits → 4 digits

Plastron A (196): ligamentous → connection between carapace and plastron osseous

Pterygoid I (221): reduced → vertical flange on lateral process absent

Quadrate F: incisura columella auris (232): quadrate completely rolled-up, quadrate-quadrate and/or quadrate-squamosal close to each other but not sutured → completely closed

Squamosal A (243): squamosal-postorbital contact present → absent

Vomer E (272): narrow and tall ventral crest on vomer absent → present all along the vomer

Vomer F (273): domed palate absent → present

Xiphiplastron A (274): distinct anal notch absent → present

**Node 158:**

Carapace A (16): partially present → absent

Cervical articulation H (28): 8)dorsal → none, vertebrae only meet at zygapophyses

Dorsal vertebra B (81): cylindrical, longer than wide, keeled ventrally → smooth and flat ventrally, hexagonal in shape

Entoplastron D (87): entoplastron V-shaped absent → present

Humerus B (118): shoulder present → shoulder absent: pleurodires

Jugal B (140): jugal participation to upper temporal rim absent → present

Manus and Pes B (145): hyperphalangy manus digits 4 and 5, pes digit 4 no → yes

Maxilla A (150): do not contact each other in ventral view → contacts each other in ventral view

Peripheral A (187): 10 pairs → less than 10 pairs

Plastron B (197): central plastral fontanella absent → present

Premaxilla D (209): exclusion of premaxilla from the apertura narium externa absent → present

Majority rule (50%)

***
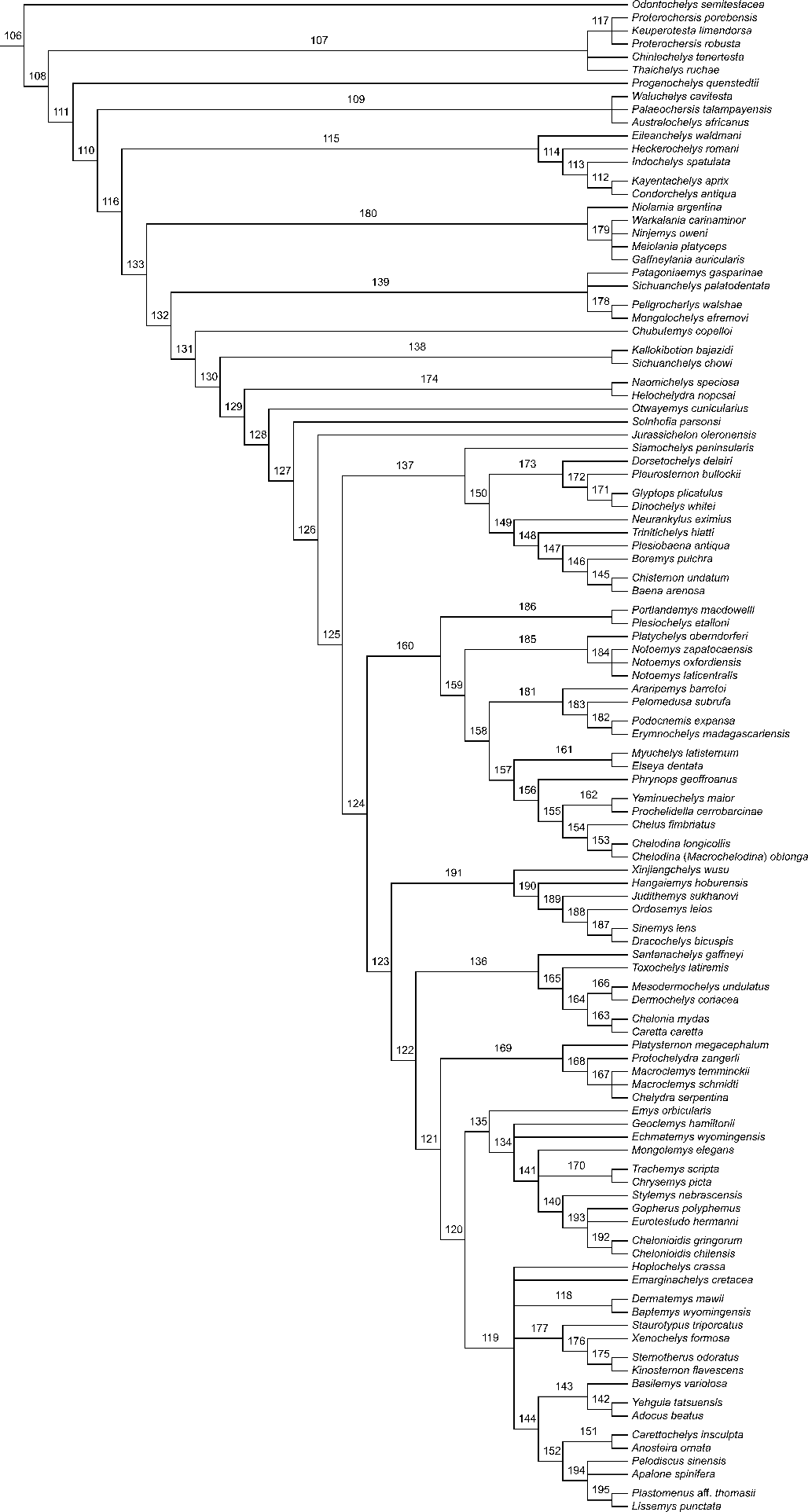
***

**Fig S2. Majority rule (50%) consensus tree.** Numbers above the branches indicate node numbers.

***Odontochelys semitestacea*:**

No autapomorphies

***Adocus beatus*:**

Pectoral B (181): antero-posteriorly developed → very short antero-posteriorly

***Anosteira ornata*:**

No autapomorphies

***Apalone spinifera*:**

Femur A (105): rectangular to oval → articular surface of femoral head triangular in dorsal view

Ilium E (132): present → thelial process absent

Maxilla D (153): triturating surface with only labial ridge present → labial and lingual ridge present

***Araripemys barretoi*:**

Abdominal A (0): present, with medial contact → present, medial contact absent

Carapace B (17): tricarinate carapace absent → present and pronounced

Carapace D (19): sculpturing of the shell absent → present

Cervical vertebra J (42): postzygapophyses not united in midline → postzygapophyses united in midline

Costal C (50): absent, costals fully or almost fully ossified, fontanelles abs or red → present

Dentary A (69): medial contact of dentaries fused → sutured only

Dorsal vertebra B (81): cylindrical, longer than wide, keeled ventrally → smooth and flat ventrally, hexagonal in shape

Entoplastron D (87): entoplastron V-shaped absent → present

Entoplastron F (89): entoplastron tightly sutured with hyoplastron yes → no

Epiplastron A (90): epiplastra and entoplastron narrow and elongate absent → present

Extragular A (96): present → absent

Gular A (112): only one scute → one pair

Hypoplastron A (127): peripheral and costal V → inguinal buttresses contact peripherals only

Hypoplastron B (128): Inguinal buttress terminates on peripheral 8 → 7

Jugal B (140): jugal participation to upper temporal rim absent → present

Maxilla B (151): involving palatine → upper triturating surface not involving palatine or its contribution is minor

Musk ducts A (157): present → absent

Nuchal emargination (166): absent or indistinct → present, excludes peripheral 1

Plastron A (196): connection between carapace and plastron osseous → ligamentous

Plastron B (197): central plastral fontanella absent → present

Pterygoid G (219): medial contact of pterygoids present → absent

***Australochelys africanus*:**

No autapomorphies

***Baena arenosa*:**

Cranial scutes A (68): absent → present

Maxilla B (151): involving palatine → upper triturating surface not involving palatine or its contribution is minor

Mesoplastron A (155): 1 pair of meso with medial contact → 1 reduced pair

Nuchal C (165): wider than long → longer than wide or as long as wide

Parietal H (179): moderate, f.s.t. but not entire processes trochlearis exposed in dorsal view → absent or weak, foramen stapedio-temporale concealed in dorsal view

***Baptemys wyomingensis*:**

Gular process (114): none, the anterolateral edge of the anterior plastral lobe even → minor, the anterior edge of the anterior plastral lobe gently scalloped

Hypoplastron A (127): inguinal buttresses contact peripherals only → peripheral and costal V

Ilium D (131): posterior notch in acetabulum absent → present

Nuchal emargination (166): absent or indistinct → present, excludes peripheral 1

***Basilemys variolosa*:**

Extragular D (98): not reaching the entoplastron → reach the entoplastron

Ilium E (132): present → thelial process absent

Inframarginal B (134): 3 or more → 2

Manus A (143): most digits with three elongate phalanges → most digits with two shortened phalanges

Nuchal emargination (166): absent or indistinct → present, excludes peripheral 1

***Boremys pulchra*:**

Marginal B (149): serration of posterior marginal scutes in adults pronounced, rounded tips of underlying peripherals → serration of posterior marginal scutes in adults pronounced, spiky tips of underlying peripherals

Postobital-maxilla contact (199): absent, jugal forms part of the orbit → present, jugal excluded from the orbit

***Thaichelys ruchae*:**

No autapomorphies

***Caretta caretta*:**

Marginal B (149): weak or no serration of posterior marginal scutes in adults → serration of posterior marginal scutes in adults pronounced, spiky tips of underlying peripherals

Nuchal emargination (166): absent or indistinct → present, excludes peripheral 1

Peripheral A (187): 11 pairs → more than 11 pairs

Quadrate H (234): processustrochlearis oticum formed by a grate contribution of quadrate → small contribution of the quadrate

***Carettochelys insculpta*:**

Quadratojugal B (237): quadratojugal-maxilla contact absent → present

***Chelodina* (*Macrochelodina*) *oblonga*:**

Extragular process B (102): minor, the anterolateral edge of the anterior plastral lobe gently scalloped → none, the anterolateral edge of the anterior plastral lobe even

***Chelodina longicollis*:**

No autapomorphies

***Chelonia mydas*:**

Cervical vertebra K (43): ventral process on cervical 8 absent → present well developed (as tall or taller than the high of the centrum)

Maxilla D (153): triturating surface with only labial ridge present, or labial and lingual ridge present → labial, lingual and accessory ridges present

Premaxilla E (210): distinct, medial premaxillary hook along the labial margin absent → present

Vertebral E (266): first vertebral scute subrectangular, hexagonal, or trapezoid with posterior edge roughly transverse and not significantly narrower than the anterior edge of the second vertebral scute → first vertebral scute bell-shaped but does not invade the area of the second vertebral scute

***Chelonoidis chilensis*:**

Gular edge (113): rounded at the end to straight → spiky, minor part of the scute width contributing to the spike

Vertebral C (265): on neural V → sulcus between V 3 and 4 on neural VI

***Chelonoidis gringorum*:**

No autapomorphies

***Chelus fimbriatus*:**

Carapace B (17): tricarinate carapace absent → present and pronounced

Cervical vertebra B (34): ventral keels absent or slightly developed in all vertebrae → ventral keels more developed on posterior vertebrae

Cervical vertebra C (35): cervical centrum 8<7 absent → present

Costal B (49): medial contact of up to three posterior costals present → medial contact of posterior costals absent

Costal C (50): absent, costals fully or almost fully ossified, fontanelles abs or red → present

Dorsal vertebra B (81): cylindrical, longer than wide, keeled ventrally → smooth and flat ventrally, hexagonal in shape

Dorsal vertebra C (82): small the entire length → wide all along the entire length of the thoracic vertebrae

Extragular D (98): not reaching the entoplastron → reach the entoplastron

Femur A (105): rectangular to oval → articular surface of femoral head triangular in dorsal view

Humeral B (116): humero-pectoral sulcus only in the hyoplastra → humero-pectoral sulcus crossing the entoplastron

Marginal B (149): weak or no serration of posterior marginal scutes in adults → serration of posterior marginal scutes in adults pronounced, spiky tips of underlying peripherals

Maxilla A (150): contacts each other in ventral view → do not contact each other in ventral view

Nasal A (158): present → absent

Prefrontal C (203): prefrontal-palatine contact absent → prefrontal-palatine contact present

Premaxilla B (207): fusion of premaxilla absent → present

Vomer D (271): absent → vomer-premaxilla contact present

Xiphiplastron B (275): xiphiplastra narrow absent → present

***Chelydra serpentina*:**

Suprapygal A (256): two elements → more than 2 elements

***Chinlechelys tenertesta*:**

No autapomorphies

***Chisternon undatum*:**

Cervical articulation A (27): not formed → formed

***Chrysemys picta*:**

Pectoral B (181): antero-posteriorly developed → very short antero-posteriorly

Pterygoid D (216): present → pterygoid-basioccipital contact absent

***Chubutemys copelloi*:**

Basiocccipital B (5): Deep C-shaped concavity present → Deep C-shaped concavity between basioccipital tubera absent

Basisphenoid D (10): Basisphenoid shape not triangular (pentagonal/quadrangular) → triangular

Hyoplastron B (125): axillary buttress terminates on peripheral 2 or 1 → terminates on peripheral 3

Hypoplastron B (128): Inguinal buttress terminates on peripheral 8 → 7

Musk ducts A (157): absent → present

***Condorchelys antiqua*:**

No autapomorphies

***Dermatemys mawii*:**

Carapace B (17): present, but only slightly → tricarinate carapace absent

Cervical vertebra E (37): present → biconvex cervical vertebra in the middle of the neck absent

Costal B (49): medial contact of posterior costals absent → medial contact of up to three posterior costals present

Humeral B (116): humero-pectoral sulcus crossing the entoplastron → humero-pectoral sulcus only in the hyoplastra

Intergular A (137): absent → present

Xiphiplastron A (274): distinct anal notch absent → present

***Dermochelys coriacea*:**

Carapace A (16): partially present → absent

Costal E (52): 8 pairs or less → 9 pairs

Peripheral A (187): 11 pairs → less than 10 pairs

***Dinochelys whitei*:**

Extragular process B (102): none, the anterolateral edge of the anterior plastral lobe even → large, projected about third or more of the extragular scute area

Gular process (114): minor, the anterior edge of the anterior plastral lobe gently scalloped, or none, the anterolateral edge of the anterior plastral lobe even → large, projected about third or more of the gular scute area

Hyoplastron B (125): terminates on peripheral 3 → axillary buttress terminates on peripheral 2 or 1

Inframarginal A (133): present → absent

Vertebral D (267): Position of sulcus between vertebral 4 and 5 on the neural series → on the suprapygals

***Dorsetochelys delairi*:**

No autapomorphies

***Dracochelys bicuspis*:**

Basioccipital A (6): with two or one ventral basioccipital tubercle → tubercle absent

Maxilla D (153): triturating surface with only labial ridge present → labial and lingual ridge present

Nuchal emargination (166): absent or indistinct → present, excludes peripheral 1

Pes A (189): absent → claw on 5th digit present

***Echmatemys wyomingensis*:**

Hyoplastron B (125): terminates on peripheral 3 → axillary buttress terminates on peripheral 2 or 1

***Eileanchelys waldmani*:**

Antrum postoticum A (3): incipient → fully developed

Gular process (114): minor, the anterior edge of the anterior plastral lobe gently scalloped → large, projected about third or more of the gular scute area

***Elseya dentata*:**

Maxilla D (153): triturating surface with only labial ridge present → labial, lingual and accessory ridges present

***Emarginachelys cretacea*:**

Marginal B (149): weak or no serration of posterior marginal scutes in adults → serration of posterior marginal scutes in adults pronounced, spiky tips of underlying peripherals

Nuchal B (164): elongate costiform process of nuchal absent, or present, process crosses peripheral I to contact pe II and even III → present, costiform process contacts peripheral 3

Plastron A (196): connection between carapace and plastron osseous → ligamentous

***Emys orbicularis*:**

Entoplastron B (85): short → size of posterior entoplastral process long

Plastron C (198): plastral kinesis absent → present

Pterygoid D (216): present → pterygoid-basioccipital contact absent

***Erymnochelys madagascariensis*:**

Extragular D (98): not reaching the entoplastron → reach the entoplastron

Hyoplastron B (125): terminates on peripheral 3 → axillary buttress terminates on peripheral 2 or 1

***Eurotestudo hermanni*:**

Hypoplastron A (127): peripheral and costal V → inguinal buttresses contact peripherals only

Marginal B (149): weak or no serration of posterior marginal scutes in adults → serration of posterior marginal scutes in adults pronounced, rounded tips of underlying peripherals

***Gaffneylania auricularis*:**

No autapomorphies

***Geoclemys hamiltonii*:**

Carapace B (17): tricarinate carapace absent → present, but only slightly

Epiplastron B (91): thick anterior border absent → thick anterior border

Gular edge (113): rounded at the end to straight → spiky, minor part of the scute width contributing to the spike

Jugal B (140): jugal participation to upper temporal rim absent → present

Pectoral B (181): antero-posteriorly developed → very short antero-posteriorly

Pterygoid J (222): not reaching the exoccipitals → reaching the exoccipitals

Vomer E (272): narrow and tall ventral crest on vomer absent → present all along the vomer

Vomer F (273): domed palate absent → present

***Glyptops plicatulus*:**

No autapomorphies

***Gopherus polyphemus*:**

Gular process (114): minor, the anterior edge of the anterior plastral lobe gently scalloped → large, projected about third or more of the gular scute area

Humerus A (117): only a groove → ectepicondylarforamen in a channel

***Hangaiemys hoburensis*:**

Basisphenoid D (10): triangular → Basisphenoid shape not triangular (pentagonal/quadrangular)

Pterygoid G (219): medial contact of pterygoids present → absent

***Heckerochelys romani*:**

Entoplastron B (85): size of posterior entoplastral process long → short

Entoplastron C (86): absent → distinct posterolateral entoplastral process present

Epiplastron A (90): epiplastra and entoplastron narrow and elongate absent → present

Extragular process B (102): minor, the anterolateral edge of the anterior plastral lobe gently scalloped → none, the anterolateral edge of the anterior plastral lobe even

Gular process (114): minor, the anterior edge of the anterior plastral lobe gently scalloped → none, the anterolateral edge of the anterior plastral lobe even

Pterygoid I (221): vertical flange on lateral process absent, or present, almost all along the lateral process → reduced

***Helochelydra nopcsai*:**

No autapomorphies

***Hoplochelys crassa*:**

Abdominal A (0): present, with medial contact → present, medial contact absent

Carapace B (17): present, but only slightly → present and pronounced

Epiplastron B (91): thick anterior border absent → thick anterior border

Hyoplastron B (125): terminates on peripheral 3 → axillary buttress terminates on peripheral 2 or 1

***Indochelys spatulata*:**

No autapomorphies

***Judithemys sukhanovi*:**

Cervical vertebra K (43): ventral process on cervical 8 absent → present well developed (as tall or taller than the high of the centrum)

***Jurassichelon oleronensis*:**

Maxilla D (153): triturating surface with only labial ridge present → labial and lingual ridge present

Parietal E (176): processus inferior parietalis forming posterior margin for nerv trigemini absent → ... present

Prootic A (211): dorsal exposure large → dorsal exposure reduced or absent

Pterygoid F (218): foramen palatinum posterius present → present, but open laterally

***Kallokibotion bajazidi*:**

Cervical A (26): one cervical present → cervicals absent, carapacial scutes otherwise present

Neural A (161): neural formula 6>4<6<6<6<6 absent → present

Xiphiplastron A (274): distinct anal notch absent → present

***Kayentachelys aprix*:**

Plastron B (197): present → central plastral fontanella absent

Pterygoid A (212): absent → pterygoid teeth present

Suprapygal A (256): two elements → one element

***Keuperotesta limendorsa*:**

No autapomorphies

***Kinosternon flavescens*:**

Dorsal vertebra B (81): cylindrical, longer than wide, keeled ventrally → smooth and flat ventrally, hexagonal in shape

Musk ducts A (157): present → absent

Plastral kinesis A (192): anterior → anterior and posterior

***Lissemys punctata*:**

Foramen jugulare posterius B (108): separated by opisthotic and or exoccipital → separated from fenestra postotica by pterygoid

Hyo-hypoplastron A (122): not fused → fused

Pterygoid I (221): reduced → vertical flange on lateral process absent

***Macroclemys schmidti*:**

Basisphenoid B (8): paired pits on ventral surface absent → present, restricted to the basisphenoid

***Macroclemys temminckii*:**

Caudal C (24): anterior caudal vertebrae procoelous or platycoelous → anterior caudal vertebrae opisthocoelous

Pubis B (227): cartilaginous or absent → epipubis process osseous or calcified

Supramarginal A (252): absent → partial row present

Suprapygal A (256): two elements → one element

***Meiolania platyceps*:**

Cranial scute E (57): scutes A, B, and C forming a continuous posterolateral shelf yes → no

Cranial scute G (59): B scute a recurved horn no → yes

Cranial scute H (60): B scute in cross section triangular → round

Cranial scute M (65): Y scute pentagonal pointing posteriorly and separating the medial contact of G scutes → rectangular not separating the medial contact of G scutes

Pterygoid C2 (215): Intrapterygoid slit extensive, completely covering fcb no → yes

Tail ring B (260): closed ventrally → open ventrally

***Mesodermochelys undulatus*:**

Cervical vertebra C (35): present → cervical centrum 8<7 absent

***Mongolemys elegans*:**

Basioccipital A (6): tubercle absent → with two or one ventral basioccipital tubercle

Basisphenoid B (8): paired pits on ventral surface absent → present, restricted to the basisphenoid

Canalis caroticum D (12): enclosed in bone → junction of palatine artery and internal carotid artery not enclosed in bone

Canalis caroticum F (14): all ots path inside the skull → enters the skull through the foramen caroticum laterale between bs and pt

Chevron A (45): absent or poorly developed along posterior caudals → present on nearly all caudals

Hyoplastron B (125): terminates on peripheral 3 → axillary buttress terminates on peripheral 2 or 1

Inframarginal B (134): 2 → 3 or more

Pterygoid H (220): absent → pterygoid contribution to foramen palatinum posterius present

Squamosal A (243): absent → squamosal-postorbital contact present

Xiphiplastron A (274): present → distinct anal notch absent

***Mongolochelys efremovi*:**

Caudal C (24): anterior caudal vertebrae opisthocoelous → anterior caudal vertebrae procoelous or platycoelous

Cervical vertebra F (38): 4th → 3rd

Maxilla D (153): labial and lingual ridge present → labial, lingual and accessory ridges present

Prootic A (211): dorsal exposure large → dorsal exposure reduced or absent

***Myuchelys latisternum*:**

Basisphenoid B (8): paired pits on ventral surface absent → present, restricted to the basisphenoid

Hypoplastron B (128): Inguinal buttress terminates on peripheral 8 → 7

***Naomichelys speciosa*:**

No autapomorphies

***Neurankylus eximius*:**

No autapomorphies

***Ninjemys oweni*:**

Cranial scute D (56): yes → X scute partially separates G scales no

Cranial scute F (58): low → D scute high

Cranial scute J (62): yes → A scute small and not forming a large shelf no

***Niolamia argentina*:**

Cranial scute D (56): yes → X scute partially separates G scales no

Cranial scute J (62): yes → A scute small and not forming a large shelf no

Cranial scute K (63): A scute comparable in size to B scute → A scute small A scute very large

Cranial scute L (64): large → Y and Z scutes relatively larges mall

Cranial scute N (66): H scute present → absent

***Notoemys laticentralis*:**

No autapomorphies

***Notoemys oxfordiensis*:**

Hypoplastron B (128): Inguinal buttress terminates on peripheral 8 → 7

***Notoemys zapatocaensis*:**

Extragular process B (102): minor, the anterolateral edge of the anterior plastral lobe gently scalloped → large, projected about third or more of the extragular scute area

***Ordosemys leios*:**

Cervical vertebra C (35): present → cervical centrum 8<7 absent

Cervical vertebra G (39): biconcave cervical vertebra absent → present

***Otwayemys cunicularius*:**

Abdominal A (0): present, with medial contact → present, medial contact absent

Caudal B (23): all centra amphicoelous → formed centra

Hypoplastron B (128): Inguinal buttress terminates on peripheral 8 → 6

***Palaeochersis talampayensis*:**

Maxilla D (153): labial and lingual ridge present → triturating surface with only labial ridge present

***Patagoniaemys gasparinae*:**

No autapomorphies

***Peligrochelys walshae*:**

Femur A (105): rectangular to oval → articular surface of femoral head triangular in dorsal view

***Pelodiscus sinensis*:**

Pterygoid H (220): absent → pterygoid contribution to foramen palatinum posterius present

***Pelomedusa subrufa*:**

Marginal B (149): weak or no serration of posterior marginal scutes in adults → serration of posterior marginal scutes in adults pronounced, rounded tips of underlying peripherals

***Phrynops geoffroanus*:**

Basisphenoid B (8): paired pits on ventral surface absent → present, restricted to the basisphenoid

***Plastomenus* aff. *thomasii*:**

Plastron B (197): present → central plastral fontanella absent

***Platychelys oberndorferi*:**

Extragular process B (102): minor, the anterolateral edge of the anterior plastral lobe gently scalloped → none, the anterolateral edge of the anterior plastral lobe even

Hyoplastron B (125): terminates on peripheral 3 → axillary buttress terminates on peripheral 2 or 1

Ilium B (130): in costals only or reaching pygal, but it does not extend onto peripherals → iliac scar extends from costals onto the peripherals and pygal

Marginal B (149): weak or no serration of posterior marginal scutes in adults → serration of posterior marginal scutes in adults pronounced, spiky tips of underlying peripherals

Supramarginal A (252): absent → partial row present

***Platysternon megacephalum*:**

Basisphenoid A (7): rostrum basisphenoidale flat → rod-like, thick, and rounded

Cervical vertebra B (34): ventral keels more developed on posterior vertebrae → ventral keels absent or slightly developed in all vertebrae

Cervical vertebra G (39): biconcave cervical vertebra absent → present

Gular process (114): none, the anterolateral edge of the anterior plastral lobe even → minor, the anterior edge of the anterior plastral lobe gently scalloped

Humerus A (117): only a groove → ectepicondylarforamen in a channel

Hypoplastron B (128): 7 → 6

Parietal H (179): moderate, f.s.t. but not entire processes trochlearis exposed in dorsal view, or strong, entire processus trochlearis exposed in dorsal view → absent or weak, foramen stapedio-temporale concealed in dorsal view

Postobital-maxilla contact (199): absent, jugal forms part of the orbit → present, jugal excluded from the orbit

Quadratojugal B (237): quadratojugal-maxilla contact absent → present

Vertebral D (267): Position of sulcus between vertebral 4 and 5 on the neural series → on the suprapygals

Xiphiplastron A (274): distinct anal notch absent → present

***Plesiobaena antiqua*:**

Cervical articulation A (27): not formed → formed

***Plesiochelys etalloni*:**

Pterygoid F (218): foramen palatinum posterius present → present, but open laterally

***Pleurosternon bullockii*:**

Cervical A (26): one cervical present → cervicals absent, carapacial scutes otherwise present

Gular A (112): one pair → only one scute

Suprapygal A (256): two elements → one element

***Podocnemis expansa*:**

Basisphenoid B (8): paired pits on ventral surface absent → present, restricted to the basisphenoid

Femur A (105): rectangular to oval → articular surface of femoral head triangular in dorsal view

Maxilla A (150): do not contact each other in ventral view → contacts each other in ventral view

Maxilla E (154): accessory ridge on maxilla present all along the triturating surface → accessory ridge only in some sectors of the triturating surface

Pterygoid H (220): pterygoid contribution to foramen palatinum posterius present → absent

***Portlandemys macdowelli*:**

Pterygoid J (222): not reaching the exoccipitals → reaching the exoccipitals

***Prochelidella cerrobarcinae*:**

No autapomorphies

***Proganochelys quenstedtii*:**

Caudal A (22): absent → tail club present

Costal E (52): 8 pairs or less → 9 pairs

Extragular process C (104): dorsal surface of the projection convex → dorsal surface distinctly concave in the posterolateral part of the projection

Gular edge (113): rounded at the end to straight → spiky, distinctly conical

Humerus E (121): length of the humerus more than two times the width of the proximal end → length of the humerus two times or less than the width of the proximal end

Nuchal emargination (166): absent or indistinct → present, excludes peripheral 1, or present, includes peripheral 1

Prefrontal E (205): absent → prefrontal heavily sculptured present

Supramarginal A (252): partial row present → complete row present

Vertebral A (263): 5 → 4

***Proterochersis porebensis*:**

No autapomorphies

***Proterochersis robusta*:**

No autapomorphies

***Protochelydra zangerli*:**

No autapomorphies

***Santanachelys gaffneyi*:**

Dorsal rib A (75): extends less than a half of costal 1 → length first thoracic rib long, extends a half or more than a half of the length of costal 1

Prefrontal A (201): medial contact on dorsal skull roof present → medial contact on dorsal skull roof absent

Pterygoid F (218): foramen palatinum posterius present → present, but open laterally

Suprapygal A (256): two elements → one element

Vertebral B (264): vertebrals II-IV narrower or as narrow as pleurals → vertebral II-IV broader than pleurals

Vertebral C (265): on neural V → sulcus between V 3 and 4 on neural VI

Vomer B (269): vomer-pterygoid contact in palatal view present → absent, medial contact of palatines present

***Siamochelys peninsularis*:**

Hypoplastron B (128): Inguinal buttress terminates on peripheral 8 → 7

Plastral scutes B (195): pronounced midline plastral sulcus sinuous absent → present

***Sichuanchelys chowi*:**

Marginal B (149): weak or no serration of posterior marginal scutes in adults → serration of posterior marginal scutes in adults pronounced, rounded tips of underlying peripherals

Nuchal emargination (166): absent or indistinct → present, includes peripheral 1

Vertebral C (265): on neural V → sulcus between V 3 and 4 on neural VI

Vertebral E (266): first vertebral scute subrectangular, hexagonal, or trapezoid with posterior edge roughly transverse and not significantly narrower than the anterior edge of the second vertebral scute → first vertebral scute bell-shaped, wide anteriorly and tapering posteriorly into a narrower, rounded median process invading the area of the wide second vertebral scute

***Sichuanchelys palatodentata*:**

Cranial scute B (54): scute D meeting in midline no → yes

Fenestra perilymphatica A (106): large → relatively small

Humerus A (117): ectepicondylarforamen in a channel → only a groove

Maxilla D (153): labial and lingual ridge present → triturating surface with only labial ridge present

Pterygoid A (212): absent → pterygoid teeth present

Vertebral E (266): first vertebral scute subrectangular, hexagonal, or trapezoid with posterior edge roughly transverse and not significantly narrower than the anterior edge of the second vertebral scute → first vertebral scute bell-shaped, wide anteriorly and tapering posteriorly into a narrower, rounded median process invading the area of the wide second vertebral scute

***Sinemys lens*:**

Epiplastron A (90): present → epiplastra and entoplastron narrow and elongate absent

Pterygoid B (213): basipt process present and sutured articulation → basipt process absent and sutured articulation

Pterygoid D (216): present → pterygoid-basioccipital contact absent

***Solnhofia parsonsi*:**

Carapace F (21): The width of the posterior half of carapace is the same or is slightly wider than the anterior half → pentagonal in shape, with the anterior border more or less straight and the posterior half tapering posteriorly

Entoplastron F (89): entoplastron tightly sutured with hyoplastron yes → no

Epiplastron A (90): epiplastra and entoplastron narrow and elongate absent → present

Maxilla C (152): secondary palate formed by premaxilla, maxilla, and vomer, palatines not contacting in midline absent → formed by premaxilla, maxilla, and vomer, palatines not contacting in midline present

Prefrontal A (201): medial contact on dorsal skull roof absent → medial contact on dorsal skull roof present

***Staurotypus triporcatus*:**

Carapace B (17): present, but only slightly → present and pronounced

Entoplastron F (89): entoplastron tightly sutured with hyoplastron yes → no

Jugal B (140): jugal participation to upper temporal rim absent → present

Pelvis B (186): two, big separated fenestra or partially separated → coalescent

***Sternotherus odoratus*:**

No autapomorphies

***Stylemys nebrascensis*:**

Hypoplastron A (127): peripheral and costal V → peripherals, costal V, and costal VI

Pubis B (227): cartilaginous or absent → epipubis process osseous or calcified

***Toxochelys latiremis*:**

Anal A (2): only cover parts of the xiphiplastra → anteromedially overlap onto hypoplastra

Chevron A (45): absent or poorly developed along posterior caudals → present on nearly all caudals

Nuchal emargination (166): absent or indistinct → present, excludes peripheral 1

***Trachemys scripta*:**

Costal D (51): absence of alternative short and long ends in the lateral part of the costals → presence

Marginal B (149): weak or no serration of posterior marginal scutes in adults → serration of posterior marginal scutes in adults pronounced, spiky tips of underlying peripherals

Maxilla E (154): accessory ridge on maxilla present all along the triturating surface → accessory ridge only in some sectors of the triturating surface

Nuchal emargination (166): absent or indistinct → present, excludes peripheral 1

Parietal A (172): absent → parietal-squamosal contact present

***Trinitichelys hiatti*:**

No autapomorphies

***Waluchelys cavitesta*:**

Coracoid foramen A (47): Length of the coracoid foramen less than half of the length of the glenoid fossa → Length of the coracoid foramen more than half of the length of the glenoid fossa

Marginal B (149): weak or no serration of posterior marginal scutes in adults → serration of posterior marginal scutes in adults pronounced, rounded tips of underlying peripherals

***Warkalania carinaminor*:**

No autapomorphies

***Xenochelys formosa*:**

Nuchal C (165): wider than long → longer than wide or as long as wide

Pterygoid F (218): foramen palatinum posterius present → absent

Xiphiplastron A (274): distinct anal notch absent → present

***Xinjiangchelys wusu*:**

Cervical articulation A (27): formed → not formed

Humerus E (121): length of the humerus more than two times the width of the proximal end → length of the humerus two times or less than the width of the proximal end

Nasal B (159): nasals contact another medially along their entire length → medial contact of nasals partially or fully hindered by long anterior fl

Neural A (161): neural formula 6>4<6<6<6<6 absent → present

Nuchal emargination (166): absent or indistinct → present, excludes peripheral 1

Prefrontal A (201): medial contact on dorsal skull roof present → medial contact on dorsal skull roof absent

Pterygoid D (216): present → pterygoid-basioccipital contact absent

***Yaminuechelys maior*:**

Carapace D (19): sculpturing of the shell absent → present

Nuchal C (165): longer than wide or as long as wide → wider than long

Nuchal emargination (166): absent or indistinct → present, excludes peripheral 1

Plastron B (197): central plastral fontanella absent → present

***Yehguia tatsuensis*:**

Humeral B (116): humero-pectoral sulcus crossing the entoplastron → humero-pectoral sulcus only in the hyoplastra

**Node 107:**

Dorsal epiplastral process D (74): located more laterally, about halfway or more between the midline and the lateral edge of the anterior plastral lobe → located closer to the midline than to the lateral edge of the anterior plastral lobe, not extending significantly beyond the lateral extent of the gular scutes

Extragular process B (103): dorsal, anterolaterally or laterally directed ridge extending from the tip of the extragular process to the base of the dorsal epiplastral process present and distinct → absent or weak

Ilium A (129): elongated iliac neck absent → present

**Node 108:**

No synapomorphies

**Node 109:**

Extragular edge C (101): ventral deflection of the anterior edge absent, the process flat or convex ventrally and V- or U-shaped in cross-section → ventral deflection of the anterior edge present, the process concave ventrally and comma shaped in cross-section

Gular process (114): minor, the anterior edge of the anterior plastral lobe gently scalloped → none, the anterolateral edge of the anterior plastral lobe even

Opisthotic B (168): depressions for musculature absent → present

Parietal D (175): overhanging process of the skull roof absent → present

Peripheral bones (188): Posterior peripheral bones without internal cavity → posterior peripheral bones with internal cavity

**Node 110:**

Cervical vertebra L (44): eighth presacral vertebra has an intermediate (transitional) cervico-dorsal morphology, can be sutured to the carapace but not to the succeeding vertebrae → eighth presacral vertebra free from the carapace and succeeding vertebrae, movable

Marginal B (149): serration of posterior marginal scutes in adults pronounced, spiky tips of underlying peripherals → weak or no serration of posterior marginal scutes in adults

Pectoral girdle A (182): horizontal plate with a dorsal process, not triradiate, bridge closing coracoid foramen as wide or wider than the width of the coracoid foramen → horizontal plate with a dorsal process, not triradiate, bridge closing coracoid foramen narrower than the width of the coracoid foramen

Pterygoid A (212): pterygoid teeth present → absent

Pterygoid B (213): basipt process present and movable articulation → basipt process present and sutured articulation

Quadrate A (229): flooring of the cranioquadrate space absent → by pt, but pt does not cover the prootic

Quadrate B + C (230): development of the c.t. shallow, but not developed antpost → shallow, but anteroposteriorly developed

Vomer C (270): vomerine and palatine teeth present → absent

**Node 111:**

Abdominal B (1): two pairs → one pair

Cervical vertebra L (44): eighth presacral vertebra co-ossified with the carapace and succeeding vertebrae → eighth presacral vertebra has an intermediate (transitional) cervico-dorsal morphology, can be sutured to the carapace but not to the succeeding vertebrae

Coracoid (46): flat, sub-ovoid or with rounded posteromedial edge (bee-wing shaped) → flat, rectangular or with a distinctly angular posteromedial edge

Femur A (105): articular surface of femoral head triangular in dorsal view → rectangular to oval

Mesoplastron A (155): 2 pairs of meso with medial contact → 1 pair of meso with medial contact

**Node 112:**

Costal E (52): 8 pairs or less → 9 pairs

**Node 113:**

Entoplastron A (84): absent → anterior entoplastral process present

**Node 114:**

Extragular process B (102): large, projected about third or more of the extragular scute area → minor, the anterolateral edge of the anterior plastral lobe gently scalloped

**Node 115:**

Basisphenoid B (8): paired pits on ventral surface absent → present, restricted to the basisphenoid

Frontal A (110): frontal contribution to orbit absent → present

Neural B (162): regular, often hexagonal, longer than wide → irregular in shape, wider than long

Prefrontal D (204): prefrontal exposure large → reduced

Vomer F (273): present → domed palate absent

**Node 116:**

Coracoid (46): flat, rectangular or with a distinctly angular posteromedial edge → columnar, at least at its base

Dorsal epiplastral process D (74): located more laterally, about halfway or more between the midline and the lateral edge of the anterior plastral lobe → located closer to the midline than to the lateral edge of the anterior plastral lobe, not extending significantly beyond the lateral extent of the gular scutes

Entoplastron A (84): anterior entoplastral process present → absent

Extragular edge B (100): lateral or anterolateral tip sharp → lateral or anterolateral tip rounded

Extragular process B (103): dorsal, anterolaterally or laterally directed ridge extending from the tip of the extragular process to the base of the dorsal epiplastral process present and distinct → absent or weak

Hypoischium A (126): present → absent

Ilium A (129): elongated iliac neck absent → present

Lacrimal A (142): present → absent

Opisthotic D2 (170): processusinterfenestralis, present, robust, not reaching the floor of cavum a-j → present, small, reaching the floor of cavum a-j

Pectoral girdle A (182): horizontal plate with a dorsal process, not triradiate, bridge closing coracoid foramen narrower than the width of the coracoid foramen → trirradiate, bridge inexistent

Pelvic girdle (184): ischium not covered ventrally by the plastron, ischium seen in ventral view → ischium covered ventrally by the plastron

Plastron A (196): connection between carapace and plastron osseous → ligamentous

Plastron B (197): central plastral fontanella absent → present

Premaxilla A (206): external nares divided → united

Pygal notch (228): present → absent

Quadrate B + C (230): shallow, but anteroposteriorly developed → deep and anteroposteriorly developed

Recessus scalae tympani A (239): almost inexistent, not surrounded by bone → well developed

Supramarginal A (252): partial row present → absent

Vomer A (268): paired → single

**Node 117:**

Dorsal epiplastral process A (71): lateroventroposterior excavation absent, no dorsally roofed depression in that area → lateroventroposterior excavation present, resulting in a distinct depression lateral to the anterior part of the medial ridge on the visceral surface of the entoplastron, partially roofed dorsally by the base of the dorsal epiplastral process

Marginal B (149): serration of posterior marginal scutes in adults pronounced, spiky tips of underlying peripherals → serration of posterior marginal scutes in adults pronounced, rounded tips of underlying peripherals

**Node 118:**

Hyoplastron A (124): axillary buttresses contact peripherals only → peripherals and first costal

Maxilla D (153): triturating surface with only labial ridge present → labial, lingual and accessory ridges present

Stapedial artery B (249): relatively large → absent

**Node 119:**

Carapace B (17): tricarinate carapace absent → present, but only slightly

Ilium E (132): thelial process absent → present

Palatine A (171): palatine contribution to anterior extension of lat braincase absent → present, well-developed

Pectoral A (180): present → absent

Pubis A (226): lateral process small, poorly developed, columnar → lateral process well developed and flat

**Node 120:**

Humerus D (120): lateral process seen in dorsal view → lateral process not seen in dorsal view

Maxilla B (151): involving palatine → upper triturating surface not involving palatine or its contribution is minor

Squamosal A (243): squamosal-postorbital contact present → absent

**Node 121:**

Cervical articulation J (30): double articulation between 6th and 7th absent → present

Cervical articulation L (32): double articulation between 7th and 8th absent → present

Cervical vertebra I (41): neural arch on 8th cervical not modified → neural arch on 8th cervical modified with the postzygapophyses pointing anteroventrally

Dorsal vertebra A (80): anterior articulation of first dorsal centrum faces at most slightly anteroventrally → faces strongly anteroventrally

Foramen jugulare posterius A (107): coalescent with fenestra postotica → separated from fenestra postotica

Pterygoid H (220): pterygoid contribution to foramen palatinum posterius present → absent

**Node 122:**

Cervical vertebra B (34): ventral keels absent or slightly developed in all vertebrae → ventral keels more developed on posterior vertebrae

Humerus A (117): ectepicondylarforamen in a channel → only a groove

Hypoplastron B (128): Inguinal buttress terminates on peripheral 8 → 7

**Node 123:**

Dorsal rib A (75): length first thoracic rib long, extends a half or more than a half of the length of costal 1 → extends less than a half of costal 1

Hyoplastron A (124): peripherals and first costal → axillary buttresses contact peripherals only

**Node 124:**

Caudal B (23): all centra amphicoelous → formed centra

Caudal C (24): anterior caudal vertebrae opisthocoelous → anterior caudal vertebrae procoelous or platycoelous

Cervical articulation A (27): not formed → formed

Cervical vertebra H (40): total height of centra and neural arch longer than the anteroposterior length of the cervical centra → total height of centra and neural arch much shorter than the anteroposterior length of the cervical centra

Chevron A (45): present on nearly all caudals → absent or poorly developed along posterior caudals

Extragular process B (102): none, the anterolateral edge of the anterior plastral lobe even → minor, the anterolateral edge of the anterior plastral lobe gently scalloped

Prefrontal A (201): medial contact on dorsal skull roof absent → medial contact on dorsal skull roof present

Pubis B (227): epipubis process osseous or calcified → cartilaginous or absent

**Node 125:**

Hyoplastron B (125): axillary buttress terminates on peripheral 2 or 1 → terminates on peripheral 3

Plastron B (197): present → central plastral fontanella absent

Pterygoid J (222): reaching the exoccipitals → not reaching the exoccipitals

Vomer B (269): absent, medial contact of palatines present → vomer-pterygoid contact in palatal view present

**Node 126:**

Hyoplastron A (124): axillary buttresses contact peripherals only → peripherals and first costal

Parietal C (174): length of anterior extension of the lateral braincase wall inter → elongated

Pterygoid L (224): like in testudinoids → like in *Kayentachelys*

**Node 127:**

Cervical articulation A (27): formed → not formed

Cervical rib A (33): present → absent

**Node 128:**

Mesoplastron A (155): 1 pair of meso with medial contact → absent

**Node 129:**

Canalis caroticum D (12): junction of palatine artery and internal carotid artery not enclosed in bone → enclosed in bone

Canalis caroticum E (13): canalis carotici interni posterior to bifurcation in ac and ap not covered ventrally by bone → covered ventrally by bone

Canalis caroticum F (14): enters the skull through the foramen caroticum laterale between bs and pt → all ots path inside the skull

Canalis caroticum G (15): fpcci (entrance of internal carotid artery into the skull) absent → formed by pterygoid

Maxilla D (153): labial and lingual ridge present → triturating surface with only labial ridge present

Nasal C (160): dorsal exposure of nasal large → greately reduced relative to that of all other elements

Pterygoid B (213): basipt process present and sutured articulation → basipt process absent and sutured articulation

Pterygoid J (222): not reaching the exoccipitals → reaching the exoccipitals

**Node 130:**

Entoplastron B (85): size of posterior entoplastral process long → short

Pterygoid D (216): pterygoid-basioccipital contact absent → present

Pterygoid L (224): processuspterygoideus externus like in *Proganochelys* → like in testudinoids

Quadrate A (229): by pt, but pt does not cover the prootic → by pt

**Node 131:**

Vomer B (269): vomer-pterygoid contact in palatal view present → absent, medial contact of palatines present

**Node 132:**

Antrum postoticum A (3): antrum postoticum absent, or incipient → fully developed

Canalis caroticum F (14): Arteria palatina enters the skull through the interpterygoid vacuity or intrapterygoid slit → enters the skull through the foramen caroticum laterale between bs and pt

Extragular process B (102): large, projected about third or more of the extragular scute area → minor, the anterolateral edge of the anterior plastral lobe gently scalloped

Manus A (143): most digits with two shortened phalanges → most digits with three elongate phalanges

Scapula A (241): lamina between the dorsal process of the scapula and the acromion well developed → lamina between the dorsal process of the scapula and the acromion reduced: *Kallokibotion*

**Node 133:**

Cervical articulation A (27): not formed → formed

Parietal B (173): parietal contact with pt, epipt, and/or palatine absent → present

Quadrate F: incisura columella auris (232): widely open, open all along its length, quadrate not completely rolled-up → quadrate completely rolled-up, quadrate-quadrate and/or quadrate-squamosal close to each other but not sutured

Quadrate G (233): processus trochlearis oticum absent → present

Supraoccipital A (253): crista occipitalis poorly developed → protruding significantly posterior to the foramen magnum

Vomer E (272): narrow and tall ventral crest on vomer absent → present all along the vomer

**Node 134:**

Gular process (114): none, the anterolateral edge of the anterior plastral lobe even → minor, the anterior edge of the anterior plastral lobe gently scalloped

Hyoplastron A (124): axillary buttresses contact peripherals only → peripherals and first costal

Hypoplastron A (127): inguinal buttresses contact peripherals only → peripheral and costal V

Xiphiplastron A (274): distinct anal notch absent → present

**Node 135:**

Cervical vertebra G (39): biconcave cervical vertebra absent → present

Nuchal C (165): wider than long → longer than wide or as long as wide

**Node 136:**

Costal C (50): absent, costals fully or almost fully ossified, fontanelles abs or red → present

Entoplastron F (89): entoplastron tightly sutured with hyoplastron yes → no

Jugal-quadrate contact (141): jugal clearly not in contact with quadrate, quadratojugal broad → jugal nearly or clearly in contact with quadrate, quadratojugal reduced

Manus and Pes A (144): carpal and tarsal elements not flattened → flattened

Manus B (146): paddles absent → short paddles present

Plastron B (197): central plastral fontanella absent → present

**Node 137:**

Carapace D (19): sculpturing of the shell absent → present

Mesoplastron A (155): absent → 1 pair of meso with medial contact

**Node 138:**

Plastron A (196): ligamentous → connection between carapace and plastron osseous

Plastron B (197): present → central plastral fontanella absent

**Node 139:**

Antrum postoticum B (4): enlarged and laterally enclosed → enlarged but not closed laterally

Basisphenoid B (8): paired pits on ventral surface absent → present in the basisphenoid (basisphenoid highly rugose) and developing posteriorly reaching the basioccipital

Nuchal emargination (166): absent or indistinct → present, includes peripheral 1

**Node 140:**

Manus A (143): most digits with three elongate phalanges → most digits with two shortened phalanges

Pectoral B (181): antero-posteriorly developed → very short antero-posteriorly

**Node 141:**

Humeral B (116): humero-pectoral sulcus crossing the entoplastron → humero-pectoral sulcus only in the hyoplastra

Hypoplastron B (128): 7 → Inguinal buttress terminates on peripheral 8

Maxilla D (153): triturating surface with only labial ridge present → labial, lingual and accessory ridges present

**Node 142:**

Extragular process B (102): minor, the anterolateral edge of the anterior plastral lobe gently scalloped → none, the anterolateral edge of the anterior plastral lobe even

**Node 143:**

Neural A (161): neural formula 6>4<6<6<6<6 absent → present

Nuchal C (165): wider than long → longer than wide or as long as wide

Pubis A (226): lateral process well developed and flat → lateral process small, poorly developed, columnar

**Node 144:**

Carapace B (17): present, but only slightly → tricarinate carapace absent

Carapace D (19): sculpturing of the shell absent → present

Cervical vertebra E (37): present → biconvex cervical vertebra in the middle of the neck absent

Foramen jugulare posterius A (107): separated from fenestra postotica → coalescent with fenestra postotica

Foramen nervi hypoglossi (XII) (109): not covered ventrally by an extension of the pterygoid and the basioccipital → covered ventrally by an extension of the pterygoid and the basioccipital

Pterygoid J (222): not reaching the exoccipitals → reaching the exoccipitals

Quadrate F: incisura columella auris (232): quadrate completely rolled-up, quadrate-quadrate and/or quadrate-squamosal close to each other but not sutured → completely closed

Squamosal D (246): long posterior process protruding beyond condylus occipitalis absent → present

Supraoccipital C (255): horizontal ventral crest in the supraoccipital absent or poorly developed anteriorly → horizontal ventral crest present along all the crista supraoccipitalis

**Node 145:**

Parietal A (172): absent → parietal-squamosal contact present

**Node 146:**

Caudal B (23): all centra amphicoelous → formed centra

Cervical A (26): one cervical present → more than one cervical present

Extragular process B (102): none, the anterolateral edge of the anterior plastral lobe even → minor, the anterolateral edge of the anterior plastral lobe gently scalloped

Gular process (114): none, the anterolateral edge of the anterior plastral lobe even → minor, the anterior edge of the anterior plastral lobe gently scalloped

**Node 147:**

Basiocccipital B (5): Deep C-shaped concavity between basioccipital tubera absent → Deep C-shaped concavity present

Extragular B (97): medial contact of extragulars absent → present, contacting one another posterior to gulars

**Node 148:**

Extragular D (98): not reaching the entoplastron → reach the entoplastron

Prefrontal D (204): reduced → absent or near absent

**Node 149:**

Epipterygoid A (93): present, laminar → absent

Marginal B (149): weak or no serration of posterior marginal scutes in adults → serration of posterior marginal scutes in adults pronounced, rounded tips of underlying peripherals

**Node 150:**

Hypoplastron A (127): inguinal buttresses contact peripherals only → peripheral and costal V

Plastron A (196): ligamentous → connection between carapace and plastron osseous

**Node 151:**

Manus C (147): short flippers present → elongate flippers present

Pterygoid I (221): reduced → vertical flange on lateral process absent

**Node 152:**

Carapace A (16): carapacial scutes present → partially present

Cervical vertebra B (34): ventral keels more developed on posterior vertebrae → ventral keels absent or slightly developed in all vertebrae

Cervical vertebra K (43): present well developed (as tall or taller than the high of the centrum) → ventral process on cervical 8 absent

Entoplastron F (89): entoplastron tightly sutured with hyoplastron yes → no

Manus C (147): flippers absent → short flippers present

Pelvis B (186): two, big separated fenestra or partially separated → coalescent

Peripheral A (187): 11 pairs → 10 pairs

Plastral scutes A (194): present → absent

Plastron A (196): connection between carapace and plastron osseous → ligamentous

Prefrontal C (203): prefrontal-palatine contact present → prefrontal-palatine contact absent

Premaxilla B (207): fusion of premaxilla absent → present

Premaxilla C (208): foramen praepalatinum present → absent, foramen intermaxillaris present

Pterygoid G (219): medial contact of pterygoids present → absent

Pterygoid L (224): like in *Kayentachelys* → like in testudinoids

Vomer B (269): vomer-pterygoid contact in palatal view present → absent, medial contact of palatines present

Vomer D (271): vomer-premaxilla contact present → absent

**Node 153:**

Carapace D (19): sculpturing of the shell absent → present

Costal A (48): medial contact of costal I absent → present

Costal B (49): medial contact of up to three posterior costals present → medial contact of all costals present

Entoplastral scute (83): absent → present

Frontal B (111): not fused → fused

Humerus B (118): shoulder present → shoulder absent: pleurodires

Hypoplastron A (127): peripheral and costal V → inguinal buttresses contact peripherals only

Parietal A (172): parietal-squamosal contact present → absent

Quadrate I (235): Quadrate-basisphenoid contact absent → present

**Node 154:**

Hypoplastron B (128): Inguinal buttress terminates on peripheral 8 → 7

Neural B (162): regular, often hexagonal, longer than wide → irregular in shape, wider than long

**Node 155:**

Cervical vertebra J (42): postzygapophyses not united in midline → postzygapophyses united in midline

**Node 156:**

Dentary A (69): medial contact of dentaries fused → sutured only

Exoccipital A (95): medial contact of exoccipitals dorsal to foramen magnum absent → present

Foramen nervi hypoglossi (XII) (109): not covered ventrally by an extension of the pterygoid and the basioccipital → covered ventrally by an extension of the bo

Supraoccipital A (253): protruding significantly posterior to the foramen magnum → crista occipitalis poorly developed

**Node 157:**

Cranial scutes A (68): present → absent

Maxilla A (150): do not contact each other in ventral view → contacts each other in ventral view

Prefrontal A (201): medial contact on dorsal skull roof present → medial contact on dorsal skull roof absent

Prefrontal D (204): prefrontal exposure large → reduced

Quadratojugal A (236): present → absent, due to the presence of a deep lower temporal emargination

**Node 158:**

Costal B (49): medial contact of posterior costals absent → medial contact of up to three posterior costals present

Dorsal rib A (75): length first thoracic rib long, extends a half or more than a half of the length of costal 1 → extends less than a half of costal 1

Humerus A (117): ectepicondylarforamen in a channel → only a groove

Nuchal C (165): wider than long → longer than wide or as long as wide

Pelvis B (186): two, big separated fenestra or partially separated → coalescent

Stapedial artery C (250): foramen stapedio-temporalis located in the dorsal part of the otic region and points dorsally → located in the anterior wall of the otic region and points anteriorly

Suprapygal A (256): two elements → one element

**Node 159:**

Basisphenoid D (10): triangular → Basisphenoid shape not triangular (pentagonal/quadrangular)

Canalis caroticum G (15): formed by pterygoid → formed by pro, pro and bs, or pro and pt

Gular A (112): one pair → only one scute

Inframarginal A (133): present → absent

Opisthotic C (169): present, with an incipient enclosed middle ear region → ventral ridge on opisthotic absent

Pelvis A (185): pelvis-shell attachment by ligaments → sutured

Pterygoid D (216): present → pterygoid-basioccipital contact absent

Quadrate A (229): by pt → by qu and pro

Quadrate G (233): present → processus trochlearis oticum absent

Xiphiplastron A (274): distinct anal notch absent → present

**Node 160:**

Hypoplastron A (127): inguinal buttresses contact peripherals only → peripheral and costal V

Opisthotic C (169): present, but modified with a enclosed middle ear region → present, with an incipient enclosed middle ear region

Plastron A (196): ligamentous → connection between carapace and plastron osseous

**Node 161:**

Costal A (48): medial contact of costal I absent → present

Costal B (49): medial contact of up to three posterior costals present → medial contact of all costals present

Extragular process B (102): minor, the anterolateral edge of the anterior plastral lobe gently scalloped → none, the anterolateral edge of the anterior plastral lobe even

Hyoplastron B (125): terminates on peripheral 3 → axillary buttress terminates on peripheral 2 or 1

Marginal B (149): weak or no serration of posterior marginal scutes in adults → serration of posterior marginal scutes in adults pronounced, spiky tips of underlying peripherals

Parietal H (179): moderate, f.s.t. but not entire processes trochlearis exposed in dorsal view, or strong, entire processus trochlearis exposed in dorsal view → absent or weak, foramen stapedio-temporale concealed in dorsal view

**Node 162:**

Mesoplastron A (155): absent → 1 reduced pair

Plastron A (196): connection between carapace and plastron osseous → ligamentous

**Node 163:**

Basiocccipital B (5): Deep C-shaped concavity between basioccipital tubera absent → Deep C-shaped concavity present

Carapace F (21): The width of the posterior half of carapace is the same or is slightly wider than the anterior half → pentagonal in shape, with the anterior border more or less straight and the posterior half tapering posteriorly

Cervical vertebra L (44): eighth presacral vertebra free from the carapace and succeeding vertebrae, movable → eighth presacral vertebra has an intermediate (transitional) cervico-dorsal morphology, can be sutured to the carapace but not to the succeeding vertebrae

Dorsal vertebra A (80): anterior articulation of first dorsal centrum faces at most slightly anteroventrally → faces strongly anteroventrally

Jugal-quadrate contact (141): jugal nearly or clearly in contact with quadrate, quadratojugal reduced → jugal clearly not in contact with quadrate, quadratojugal broad

Maxilla C (152): secondary palate formed by premaxilla, maxilla, and vomer, palatines not contacting in midline absent → formed by premaxilla, maxilla, and vomer, palatines not contacting in midline present

Pelvis B (186): two, big separated fenestra or partially separated → coalescent

Premaxilla C (208): foramen praepalatinum present → absent, premaxillae well-ossified

Vomer D (271): vomer-premaxilla contact present → absent

**Node 164:**

Antrum postoticum A (3): fully developed → incipient

Basisphenoid A (7): rostrum basisphenoidale flat → rod-like, thick, and rounded

Manus B (146): short paddles present → elongate paddles present

Parietal A (172): absent → parietal-squamosal contact present

Pterygoid F (218): foramen palatinum posterius present → absent

Pterygoid I (221): reduced → vertical flange on lateral process absent

Pterygoid L (224): like in *Kayentachelys* → like in testudinoids

**Node 165:**

Epiplastron A (90): epiplastra and entoplastron narrow and elongate absent → present

Hyoplastron B (125): terminates on peripheral 3 → terminates on peripheral 4

Xiphiplastron B (275): xiphiplastra narrow absent → present

**Node 166:**

Carapace A (16): carapacial scutes present → partially present

Cervical vertebra H (40): total height of centra and neural arch much shorter than the anteroposterior length of the cervical centra → total height of centra and neural arch longer than the anteroposterior length of the cervical centra

Plastral scutes A (194): present → absent

**Node 167:**

Plastron B (197): central plastral fontanella absent → present

**Node 168:**

Abdominal A (0): present, with medial contact → present, medial contact absent

Anal A (2): only cover parts of the xiphiplastra → anteromedially overlap onto hypoplastra

Epiplastron A (90): epiplastra and entoplastron narrow and elongate absent → present

**Node 169:**

Chevron A (45): absent or poorly developed along posterior caudals → present on nearly all caudals

Hyoplastron B (125): terminates on peripheral 3 → terminates on peripheral 4

Neural B (162): regular, often hexagonal, longer than wide → irregular in shape, wider than long

Pterygoid J (222): not reaching the exoccipitals → reaching the exoccipitals

Quadrate F: incisura columella auris (232): quadrate completely rolled-up, quadrate-quadrate and/or quadrate-squamosal close to each other but not sutured → completely closed

**Node 170:**

Cervical articulation I (29): double articulation between 5th and 6th absent → present

Gular edge (113): rounded at the end to straight → spiky, minor part of the scute width contributing to the spike

Suprapygal A (256): two elements → one element

**Node 171:**

Premaxilla E (210): distinct, medial premaxillary hook along the labial margin absent → present

**Node 172:**

Canalis caroticum D (12): enclosed in bone → junction of palatine artery and internal carotid artery not enclosed in bone

Canalis caroticum E (13): covered ventrally by bone → canalis carotici interni posterior to bifurcation in ac and ap not covered ventrally by bone

Canalis caroticum F (14): all ots path inside the skull → enters the skull through the foramen caroticum laterale between bs and pt

Nasal B (159): nasals contact another medially along their entire length → medial contact of nasals partially or fully hindered by long anterior fl

Postobital-maxilla contact (199): absent, jugal forms part of the orbit → present, jugal excluded from the orbit

Pterygoid B (213): basipt process absent and sutured articulation → basipt process present and sutured articulation

Pterygoid G (219): medial contact of pterygoids present → absent

**Node 173:**

Basioccipital A (6): tubercle absent → with two or one ventral basioccipital tubercle

Basisphenoid B (8): paired pits on ventral surface absent → present, restricted to the basisphenoid

Basisphenoid D (10): triangular → Basisphenoid shape not triangular (pentagonal/quadrangular)

Parietal H (179): moderate, f.s.t. but not entire processes trochlearis exposed in dorsal view → absent or weak, foramen stapedio-temporale concealed in dorsal view

**Node 174:**

Basisphenoid B (8): paired pits on ventral surface absent → present in in the basisphenoid and pterygoid or in the pterygoid

Carapace D (19): sculpturing of the shell absent → present

Cranial scutes A (68): present → absent

Postobital-maxilla contact (199): absent, jugal forms part of the orbit → present, jugal excluded from the orbit

**Node 175:**

Costal A (48): medial contact of costal I absent → present

Entoplastron E (88): present → absent

Humeral A (115): 1 pair → 2 pair subdivided by a plastral hinge

Intergular A (137): absent → present

**Node 176:**

Gular process (114): none, the anterolateral edge of the anterior plastral lobe even → minor, the anterior edge of the anterior plastral lobe gently scalloped

Vertebral E (266): first vertebral scute subrectangular, hexagonal, or trapezoid with posterior edge roughly transverse and not significantly narrower than the anterior edge of the second vertebral scute → first vertebral scute bell-shaped but does not invade the area of the second vertebral scute

**Node 177:**

Abdominal B (1): one pair → absent

Dorsal rib B (76): contact of the two last dorsal rib pairs with costals present → absent

Dorsal rib E (79): 10 pairs → 9 pairs or less

Frontal A (110): present → frontal contribution to orbit absent

Hyoplastron B (125): terminates on peripheral 3 → terminates on peripheral 4

Ilium D (131): posterior notch in acetabulum absent → present

Inframarginal B (134): 3 or more → 2

Inframarginal C (135): axillar and inguinal not in contact → axillar and inguinal in contact

Peripheral A (187): 11 pairs → 10 pairs

Pterygoid I (221): reduced → vertical flange on lateral process absent

Quadrate F: incisura columella auris (232): quadrate completely rolled-up, quadrate-quadrate and/or quadrate-squamosal close to each other but not sutured → completely closed

Quadratojugal B (237): quadratojugal-maxilla contact absent → present

Stapedial artery B (249): relatively large → significantly reduced in size

**Node 178:**

Neural B (162): regular, often hexagonal, longer than wide → irregular in shape, wider than long

Squamosal C (245): posterolateral protuberances developing horns absent → small protuberances

**Node 179:**

Cranial scale P (53): scale J formed by several scales → scale J formed by only one scale

Cranial scute B (54): scute D meeting in midline no → yes

Maxilla D (153): labial and lingual ridge present → labial, lingual and accessory ridges present

**Node 180:**

Canalis caroticum E (13): canalis carotici interni posterior to bifurcation in ac and ap not covered ventrally by bone → covered ventrally by bone

Canalis caroticum G (15): fpcci (entrance of internal carotid artery into the skull) absent → formed by pterygoid

Caudal A (22): absent → tail club present

Cranial scute O (67): scale F formed by several scales → scale F formed by only one scale

Marginal B (149): weak or no serration of posterior marginal scutes in adults → serration of posterior marginal scutes in adults pronounced, rounded tips of underlying peripherals, or serration of posterior marginal scutes in adults pronounced, spiky tips of underlying peripherals

Pterygoid B (213): basipt process present and sutured articulation → basipt process absent and sutured articulation

Pterygoid D (216): pterygoid-basioccipital contact absent → present

Pterygoid L (224): processuspterygoideus externus like in *Proganochelys* → like in testudinoids

Quadratojugal C (238): quadratojugal-squamosal contact below cavum tympani absent → present

Squamosal B (244): squamosal-supraoccipital contact absent → present

Squamosal C (245): posterolateral protuberances developing horns absent → big protuberances developed as horns

Supraoccipital B (254): large supraoccipital exposure to dorsal skull roof absent → present

Tail ring A (259): absent → present

**Node 181:**

Nasal A (158): present → absent

Quadrate D (231): precolumellar fossa absent → large and deep

Vomer B (269): vomer-pterygoid contact in palatal view present → absent, medial contact of palatines present

**Node 182:**

Extragular process B (102): minor, the anterolateral edge of the anterior plastral lobe gently scalloped → none, the anterolateral edge of the anterior plastral lobe even

Humeral B (116): humero-pectoral sulcus only in the hyoplastra → humero-pectoral sulcus crossing the entoplastron

Parietal H (179): moderate, f.s.t. but not entire processes trochlearis exposed in dorsal view, or strong, entire processus trochlearis exposed in dorsal view → absent or weak, foramen stapedio-temporale concealed in dorsal view

Pterygoid K (223): Fossa podocnemidoidea absent → present

**Node 183:**

Maxilla D (153): triturating surface with only labial ridge present → labial, lingual and accessory ridges present

Mesoplastron A (155): absent → 1 reduced pair

**Node 184:**

Hypoplastron A (127): peripheral and costal V → inguinal buttresses contact peripherals only

Musk ducts A (157): present → absent

**Node 185:**

Dorsal rib D (78): articulation tubercule on the anterior face of the first thoracic rib absent, smooth anterior face → present

Dorsal vertebra B (81): cylindrical, longer than wide, keeled ventrally → smooth and flat ventrally, hexagonal in shape

Dorsal vertebra C (82): small the entire length → wide all along the entire length of the thoracic vertebrae

Extragular D (98): not reaching the entoplastron → reach the entoplastron

Mesoplastron A (155): absent → 1 reduced pair

Neural B (162): regular, often hexagonal, longer than wide → irregular in shape, wider than long

Plastron B (197): central plastral fontanella absent → present

Vertebral C (265): on neural V → sulcus between V 3 and 4 on neural VI

**Node 186:**

Basisphenoid B (8): paired pits on ventral surface absent → present, restricted to the basisphenoid

Maxilla D (153): triturating surface with only labial ridge present → labial and lingual ridge present

Parietal C (174): elongated → length of anterior extension of the lateral braincase wall inter

Parietal E (176): processus inferior parietalis forming posterior margin for nerv trigemini absent → ... present

**Node 187:**

Cervical A (26): one cervical present → cervicals absent, carapacial scutes otherwise present

**Node 188:**

Dorsal rib A (75): extends less than a half of costal 1 → length first thoracic rib long, extends a half or more than a half of the length of costal 1

Parietal H (179): moderate, f.s.t. but not entire processes trochlearis exposed in dorsal view → strong, entire processus trochlearis exposed in dorsal view

Plastron B (197): central plastral fontanella absent → present

Prefrontal A (201): medial contact on dorsal skull roof present → medial contact on dorsal skull roof absent

**Node 189:**

Cervical vertebra B (34): ventral keels absent or slightly developed in all vertebrae → ventral keels more developed on posterior vertebrae

Entoplastron F (89): entoplastron tightly sutured with hyoplastron yes → no

**Node 190:**

Basisphenoid B (8): paired pits on ventral surface absent → present, restricted to the basisphenoid

Epiplastron A (90): epiplastra and entoplastron narrow and elongate absent → present

**Node 191:**

Anal A (2): only cover parts of the xiphiplastra → anteromedially overlap onto hypoplastra

Canalis caroticum D (12): enclosed in bone → junction of palatine artery and internal carotid artery not enclosed in bone

Canalis caroticum F (14): all ots path inside the skull → enters the skull through the foramen caroticum laterale between bs and pt

Hyoplastron B (125): terminates on peripheral 3 → axillary buttress terminates on peripheral 2 or 1

Pterygoid B (213): basipt process absent and sutured articulation → basipt process present and sutured articulation

**Node 192:**

Cervical A (26): one cervical present → cervicals absent, carapacial scutes otherwise present

**Node 193:**

Costal D (51): absence of alternative short and long ends in the lateral part of the costals → presence

Epiplastron B (91): thick anterior border absent → thick anterior border

Hyoplastron B (125): terminates on peripheral 3 → axillary buttress terminates on peripheral 2 or 1

Pes C (191): 5 digits → 4 digits

**Node 194:**

Carapace A (16): partially present → absent

Cervical articulation H (28): 8)dorsal → none, vertebrae only meet at zygapophyses

Dorsal vertebra B (81): cylindrical, longer than wide, keeled ventrally → smooth and flat ventrally, hexagonal in shape

Entoplastron D (87): entoplastron V-shaped absent → present

Humerus B (118): shoulder present → shoulder absent: pleurodires

Jugal B (140): jugal participation to upper temporal rim absent → present

Manus and Pes B (145): hyperphalangy manus digits 4 and 5, pes digit 4 no → yes

Maxilla A (150): do not contact each other in ventral view → contacts each other in ventral view

Peripheral A (187): 10 pairs → less than 10 pairs

Plastron B (197): central plastral fontanella absent → present

Premaxilla D (209): exclusion of premaxilla from the apertura narium externa absent → present

**Node 195:**

Foramen jugulare posterius A (107): coalescent with fenestra postotica → separated from fenestra postotica

References

1. Hay OP. The fossil turtles of North America. Washington D.C.: Carnegie Institution; 1908.

2. Meylan PA, Gaffney ES. The skeletal morphology of the Cretaceous cryptodiran turtle, <i>Adocus<\i>, and the relationships of the Trionychoidea. Am Museum Novit. 1989;2941: 1–60.

3. Price LI. Quelônio amphichelydia no Cretáceo Inferior do nordeste do Brasilá. Rev Bras Geociências. 1973;3: 84–96. doi:10.25249/0375-7536.19738496

4. Meylan PA. Skeletal morphology and relationships of the Early Cretaceous side-necked turtle, *Araripemys barretoi* (testudines: Pelomedusoides: Araripemydidae), from the Santana Formation of Brazil. J Vertebr Paleontol. 1996;16: 20–33. doi:10.1080/02724634.1996.10011280

5. Gaffney ES. The systematics of the North American family Baenidae (Reptilia, Cryptodira). Bull Am Museum Nat Hist. 1972;147: 241–320.

6. Adrian B. Stratigraphic range extension of the turtle *Boremys pulchra* (Testudinata, Baenidae) through at least the uppermost Cretaceous. Foss Rec. 2022;25: 275–285. doi:10.3897/fr.25.85563

7. Brinkman DB, Nicholls EL. Anatomy and relationships of the turtle *Boremys pulchra* (Testudines: Baenidae). J Vertebr Paleontol. 1991;11: 302–315. doi:10.1080/02724634.1991.10011400

8. Broin F de, Ingavat R, Janvier P, Sattayarak N. Triassic turtle remains from northeastern Thailand. J Vertebr Paleontol. 1982;2: 41–46.

9. Broin F de. *Proganochelys ruchae* n.sp., chélonien du Trias supérieur de Thaïlande. Stud Palaeocheloniologica. 1984;1: 87–97.

10. Wyneken J. The anatomy of sea turtles. U.S. Department of Commerce NOAA Technical Memorandum NMFS-SEFSC-470; 2001.

11. Waite ER. The osteology of the New Guinea turtle (*Carettochelys insculpta*, Ramsay). Rec Aust Museum. 1905;6: 110–118. doi:10.3853/j.0067-1975.6.1905.994

12. Boulenger GA. Catalogue of the chelonians, rhynchocephalians, and crocodiles in the British Museum (Natural History). London: Order of the Trustees; 1889.

13. Shea G, Thomson S, Georges A. The identity of *Chelodina oblonga* Gray 1841 (Testudines: Chelidae) reassessed. Zootaxa. 2020;4779: 419–437. doi:10.11646/zootaxa.4779.3.9

14. Rhodin AGJ, Iverson JB, Bour R, Fritz U, Georges A, Shaffer HB, et al. Turtles of the world. Annotated checklist and atlas of taxonomy, synonymy, distribution, and conservation status (9th ed.). Chelonian Res Monogr. 2021;8: 1–472.

15. Simpson GG. A Miocene tortoise from Patagonia. Am Museum Novit. 1942;1209: 1–6.

16. Oriozabala C, Sterli J, Ruiz LG. Morphology of the mid-sized tortoises (Testudines: Testudinidae) from the middle Miocene of northwestern Chubut (Argentina). Ameghiniana. 2018;55: 30–54. doi:10.5710/AMGH.18.05.2017.3078

17. Maniel IJ, de la Fuente MS. A review of the fossil record of turtles of the clade *Pan-Chelidae*. Bull Peabody Museum Nat Hist. 2016;57: 191–227.

18. Joyce WG. A review of the fossil record of turtles of the clade *Pan-Chelydridae*. Bull Peabody Museum Nat Hist. 2016;57: 21–56.

19. Sterli J, de la Fuente MS, Umazano AM. New remains and new insights on the Gondwanan meiolaniform turtle *Chubutemys copelloi* from the Lower Cretaceous of Patagonia, Argentina. Gondwana Res. 2015;27: 978–994. doi:10.1016/j.gr.2013.08.016

20. Gaffney ES, Rich TH, Vickers-Rich P, Constantine A, Vacca R, Kool L. *Chubutemys*, a new eucryptodiran Ttrtle from the Early Cretaceous of Argentina, and the relationships of the Meiolaniidae. Am Museum Novit. 2007;3599: 1–35. doi:10.1206/0003-0082(2007)3599[1:CANETF]2.0.CO;2

21. Sterli J. A new, nearly complete stem turtle from the Jurassic of South America with implications for turtle evolution. Biol Lett. 2008;4: 286–289. doi:10.1098/rsbl.2008.0022

22. Sterli J, de la Fuente MS, Rougier GW. New remains of *Condorchelys antiqua* (Testudinata) from the Early-Middle Jurassic of Patagonia: anatomy, phylogeny, and paedomorphosis in the early evolution of turtles. J Vertebr Paleontol. 2018;38: e1480112. doi:10.1080/02724634.2018.1480112

23. Völker H. Über das Stamm-, Gliedmaßen- und Hautskelet von *Dermochelys coriacea* L. Zool Jahrbücher. 1913;33: 431–552.

24. Gaffney ES. The Jurassic turtles of North America. Bull Am Museum Nat Hist. 1979;162: 91–135.

25. Brinkman DB. New material of *Dracochelys* (Eucryptodira: Sinemydidae) from the Junggar Basin, Xinjiang, People’s Republic of China. Can J Earth Sci. 2001;38: 1645–1651.

26. Anquetin J, Barrett PM, Jones MEH, Moore-Fay S, Evans SE. A new stem turtle from the Middle Jurassic of Scotland: new insights into the evolution and palaeoecology of basal turtles. Proc R Soc B. 2009;276: 879–886.

27. Anquetin J. The anatomy of the basal turtle *Eileanchelys waldmani* from the Middle Jurassic of the Isle of Skye, Scotland. Earth Environ Sci Trans R Soc Edinburgh. 2010;101: 67–96. doi:10.1017/S1755691010009217

28. Whetstone KN. A new genus of cryptodiran turtles (Testudinoidea, Chelydridae) from the Upper Cretaceous Hell Creek Formation of Montana. Univ Kansas Sci Bull. 1978;51: 539–563.

29. Pérez-García A, De Lapparent De Broin F, Murelaga X. The *Erymnochelys* group of turtles (Pleurodira, Podocnemididae) in the Eocene of Europe: New taxa and paleobiogeographical implications. Palaeontol Electron. 2017;20: 14A. doi:10.26879/687

30. Garbin R de C. Intraspecific variation in the shell of Geoemydidae Turtles: Applications to systematics and paleontology. University of Fribourg. 2019.

31. Auffenberg W. The genus *Gopherus* (Testudinidae): Pt. I. Osteology and relationships of extant species. Bull Florida State Museum, Biol Sci. 1976;20: 47–110.

32. Sukhanov VB, Narmandakh P. Novaya rannemelovaya cherepakha iz kontinentalnykh otlozheniy severnoy Gobi. Mesozoic Cenozoic Faunas Biostratigraphy Mong Jt Sov Palaeontol Exped Trans. 1974;1: 192–200.

33. Sukhanov VB. An archaic turtle, *Heckerochelys romani* gen. et sp. nov., from the Middle Jurassic of Moscow Region, Russia. Foss Turt Res. 2006;1: 112–118.

34. Datta PM, Manna P, Ghosh SC, Das DP. The first Jurassic turtle from India. Palaeontology. 2000;43: 99–109. doi:10.1111/1475-4983.00120

35. Joyce WG, Bandyopadhyay S. A reevaluation of the basal turtle *Indochelys spatulata* from the Early-Middle Jurassic (Toarcian-Aalenian) of India, with descriptions of new material. PeerJ. 2020;8: e8542. doi:10.7717/peerj.8542

36. Parham JF, Hutchison JH. A new eucryptodiran turtle from the Late Cretaceous of North America (Dinosaur Provincial Park, Alberta, Canada). J Vertebr Paleontol. 2003;23: 783–798. doi:10.1671/5

37. Rieppel OC. The skull of the Upper Jurassic cryptodire turtle *Thalassemys*, with a reconsideration of the chelonian braincase. Palaeontogr A. 1980;171: 105–140.

38. Gaffney ES, Meylan PA. The Transylvanian turtle, *Kallokibotion*, a primitive cryptodire of Cretaceous age. Am Museum Novit. 1992; 1–37.

39. Pérez-García A, Codrea V. New insights on the anatomy and systematics of *Kallokibotion* Nopcsa, 1923, the enigmatic uppermost Cretaceous basal turtle (stem Testudines) from Transylvania. Zool J Linn Soc. 2017; 1–25. doi:10.1093/zoolinnean/zlx037/4101225/New-insights-on-the-anatomy-and-systematics-of

40. Nopcsa F. *Kallokibotium*. A primitive amphichelydean tortoise from the uppermost Cretaceous of Hungary. Palaeontol Hungarica. 1923;1: 1–34.

41. Gaffney ES, Hutchison JH, Jenkins FA, Meeker LJ. Modern turtle origins: The oldest known cryptodire. Science (80- ). 1987;237: 289–291. doi:10.1126/science.237.4812.289

42. Joyce WG, Jenkins FA, Rowe T. The presence of cleithra in the basal turtle *Kayentachelys aprix*. Foss Turt Res. 2006;1: 93–103.

43. Joyce WG, Schoch RR, Lyson TR. The girdles of the oldest fossil turtle, *Proterochersis robusta*, and the age of the turtle crown. BMC Evol Biol. 2013;13: 266. doi:10.1186/1471-2148-13-266

44. Szczygielski T, Sulej T. Revision of the Triassic European turtles *Proterochersis* and *Murrhardtia* (Reptilia, Testudinata, Proterochersidae), with the description of new taxa from Poland and Germany. Zool J Linn Soc. 2016;177: 395–427. doi:10.1111/zoj.12374

45. Szczygielski T. Homeotic shift at the dawn of the turtle evolution. R Soc Open Sci. 2017;4: 160933. doi:http://dx.doi.org/10.1098/rsos.160933

46. Szczygielski T, Piechowski R. Limb anatomy of the Triassic turtles: appendicular osteology of *Proterochersis* (Testudinata, Proterochersidae). Zool J Linn Soc. 2023.

47. Meylan PA. The phylogenetic relationships of soft-shelled turtles (family Trionychidae). Bull Am Museum Nat Hist. 1987;186: 1–101.

48. Vitek NS, Joyce WG. A review of the fossil record of new world turtles of the clade *Pan-Trionychidae*. Bull Peabody Museum Nat Hist. 2015;56: 185–244.

49. Joyce WG. Phylogenetic relationships of Mesozoic turtles. Bull Peabody Museum Nat Hist. 2007;48: 3–102.

50. Gaffney ES. The comparative osteology of the Triassic turtle *Proganochelys*. Bull Am Museum Nat Hist. 1990;194: 1–263.

51. Gaffney ES. The postcranial morphology of *Meiolania platyceps* and a review of the Meiolaniidae. Bull Am Museum Nat Hist. 1996;229: 1–166.

52. Hirayama R, Chitoku T. Family Dermochelyidae (superfamily Chelonioidea) from the Upper Cretaceous of North Japan. Trans Palaeontol Soc Japan. 1996;284: 597–622.

53. Khosatzky LI, Młynarski M. Chelonians from the Upper Cretaceous of the Gobi Desert, Mongolia. Palaeontol Pol. 1971;25: 131–144.

54. Cadena EA, Ksepka DT, Norell MA. New material of *Mongolemys elegans* Khosatzky and Mlynarski, 1971 (Testudines: Lindholmemydidae), from the Late Cretaceous of Mongolia with comments on bone histology and phylogeny. Am Museum Novit. 2013;1971: 1–28. doi:10.1206/3766.2

55. Khosatzky LI. Big turtle of the Late Cretaceous of Mongolia. Russ J Herpetol. 1997;4: 148–154.

56. Suzuki S, Chinzorig T, Tsubamoto T. A catalog of *Mongolochelys* collected by the HMNS-MPC Joint Paleontological Expedition. Hayashibara Museum Nat Sci Res Bull. 2010;3: 119–131.

57. Joyce WG, Sterli J, Chapman SD. The skeletal morphology of the solemydid turtle *Naomichelys speciosa* from the Early Cretaceous of Texas. J Paleontol. 2014;88: 1257–1287. doi:10.1666/14-002

58. Larson DW, Longrich NR, Evans DC, Ryan MJ. A new species of *Neurankylus* from the Milk River Formation (Cretaceous: Santonian) of Alberta, Canada, and a eevision of the type species *N. eximius*. In: Brinkman DB, Holroyd PA, Gardner JD, editors. Morphology and Evolution of Turtles. Dordrecht: Springer; 2012. pp. 385–405.

59. Sterli J, de la Fuente MS. Re-description and evolutionary remarks on the Patagonian horned turtle *Niolamia argentina* Ameghino, 1899 (Testudinata, Meiolaniidae). J Vertebr Paleontol. 2011;31: 1210–1229. doi:10.1080/02724634.2011.615685

60. Lapparent de Broin F de, De La Fuente MS, Fernandez MS. *Notoemys laticentralis* (Chelonii, Pleurodira), Late Jurassic of Argentina: New examination of the anatomical structures and comparisons. Rev Paleobiol. 2007;26: 99–136.

61. López-Conde OA, Alvarado-Ortega J. Revisión morfológica de las tortugas del género *Notoemys*. Paleontol Mex. 2017;6: 79–89.

62. Fernandez MS, de la Fuente MS. Redescription and phylogenetic position of *Notoemys*: The oldest Gondwanian pleurodiran turtle. Neues Jahrb für Geol und Paläntologie Abhandlungen. 1994;193: 81–105.

63. de la Fuente MS, Iturralde-Vinent M. A new pleurodiran turtle from the Jagua Formation (Oxfordian) of Western Cuba. J Paleontol. 2001;75: 860–869.

64. Cadena E, Gaffney ES. *Notoemys zapatocaeneis*, a new side necked turtle (Pleurodira: Platychelyidae) from the Early Cretaceous of Colombia. Am Museum Novit. 2005;3470: 1–19. doi:10.1206/0003-0082(2005)470<0001:NZANST>2.0.CO;2

65. Cadena EA, Jaramillo CA, Bloch JI. New material of the platychelyid turtle *Notoemys zapatocaensis* from the Early Cretaceous of Colombia; Implications for understanding Pleurodira evolution. In: Brinkman DB, Holroyd PA, Gardner JD, editors. Morphology and Evolution of Turtles. Dordrecht: Springer Science+Business Media; 2013. pp. 105–120.

66. Li C, Wu X, Rieppel O, Wang L, Zhao L. An ancestral turtle from the Late Triassic of southwestern China. Nature. 2008;456: 497–501. doi:10.1038/nature07533

67. Lyson TR, Schachner ER, Botha-Brink J, Scheyer TM, Lambertz M, Bever GS, et al. Origin of the unique ventilatory apparatus of turtles. Nat Commun. 2014;5: 5211. doi:10.1038/ncomms6211

68. Nagashima H, Hirasawa T, Sugahara F, Takechi M, Usuda R, Sato N, et al. Origin of the unique morphology of the shoulder girdle in turtles. J Anat. 2013;223: 547–556. doi:10.1111/joa.12116

69. Nagashima H, Sugahara F, Takechi M, Ericsson R, Kawashima-Ohya Y, Narita Y, et al. Evolution of the turtle body plan by the folding and creation of new muscle connections. Science. 2009;325: 193–6. doi:10.1126/science.1173826

70. Rothschild BM, Naples V. Decompression syndrome and diving behavior in *Odontochelys*, the first turtle. Acta Palaeontol Pol. 2015;60: 163–167. doi:10.4202/app.2012.0113

71. Li C, Fraser NC, Rieppel OC, Xiao-Chun W, Wu XC. A Triassic stem turtle with an edentulous beak. Nature. 2018;560: 476–479. doi:10.1038/s41586-018-0419-1

72. Hirayama R. Oldest known turtle *Odontochelys* [in Japanese]. O Kenshō Suru, Iden. 2009;63: 2–4.

73. Hirasawa T, Pascual-Anaya J, Kamezaki N, Taniguchi M, Mine K, Kuratani S. The evolutionary origin of the turtle shell and its dependence on the axial arrest of the embryonic rib cage. J Exp Zool Part B Mol Dev Evol. 2015;324: 194–207. doi:10.1002/jez.b.22579

74. Hirasawa T, Nagashima H, Kuratani S. The endoskeletal origin of the turtle carapace. Nat Commun. 2013;4: 1–7. doi:10.1038/ncomms3107

75. Brinkman DB, Peng J-H. *Ordosemys leios*, n.gen., n.sp., a new turtle from the Early Cretaceous of the Ordos Basin, Inner Mongolia. Can J Earth Sci. 1993;30: 2128–2138.

76. Gaffney ES, Kool L, Brinkman DB, Rich TH, Vickers-Rich P. *Otwayemys*, a new cryptodiran turtle from the Early Cretaceous of Australia. Am Museum Novit. 1998;3233: 1–28. doi:n/a

77. Rougier GW, De La Fuente MS, Arcucci AB. Late Triassic turtles from South America. Science (80- ). 1995;268: 855–858. doi:10.1126/science.268.5212.855

78. Sterli J, de la Fuente MS, Rougier GW. Anatomy and relationships of *Palaeochersis talampayensis*, a Late Triassic turtle from Argentina. Palaeontogr Abteilung A. 2007;281: 1–61.

79. Sterli J, Martínez RN, Cerda IA, Apaldetti C. Appearances can be deceptive: Bizarre shell microanatomy and histology in a new Triassic turtle (Testudinata) from Argentina at the dawn of turtles. Pap Palaeontol. 2021;7: 1097–1132. doi:10.1002/spp2.1334

80. de la Fuente MS, Sterli J, Krapovickas V. Triassic turtles from Pangea: The legacy from South America. J South Am Earth Sci. 2021;105: 102910. doi:10.1016/j.jsames.2020.102910

81. Sterli J, de la Fuente MS. A new turtle from the La Colonia Formation (Campanian-Maastrichtian), Patagonia, Argentina, with remarks on the evolution of the vertebral column in turtles. Palaeontology. 2011;54: 63–78. doi:10.1111/j.1475-4983.2010.01002.x

82. Baldo D, Martinez P, Boeris JM, Giraudo AR. Reptilia, Chelonii, Chelidae, *Phrynops geoffroanus* Schweigger, 1812 and *Mesoclemmys vanderhaegei* (Bour, 1973): distribution extension, new country record, and new province records in Argentina. Check List. 2007;3: 348–352. doi:10.15560/3.4.348

83. Bona P, Alcalde L. Chondrocranium and skeletal development of *Phrynops hilarii* (Pleurodira: Chelidae). Acta Zool. 2009;90: 301–325. doi:10.1111/j.1463-6395.2008.00356.x

84. Bräm H. Die Schildkröten aus dem oberen Jura (Malm) der Gegend von Solothurn. Schweizerische Paläontologische Abhandlungen. 1965;83: 1–190.

85. Sullivan PM, Joyce WG. The shell and pelvic anatomy of the Late Jurassic turtle *Platychelys oberndorferi* based on material from Solothurn, Switzerland. Swiss J Palaeontol. 2017;136: 323–343.

86. Brinkman DB. Anatomy and systematics of *Plesiobaena antiqua* (Testudines; Baenidae) from the mid-Campanian Judith River Group of Alberta, Canada. J Vertebr Paleontol. 2003;23: 146–155. doi:10.1671/0272-4634(2003)23

87. Anquetin J, Deschamps S, Claude J. The rediscovery and redescription of the holotype of the Late Jurassic turtle *Plesiochelys etalloni*. PeerJ. 2014;2: e258. doi:10.7717/peerj.258

88. Anquetin J, Püntener C, Billon-Bruyat J-P. A taxonomic review of the Late Jurassic eucryptodiran turtles from the Jura Mountains (Switzerland and France). PeerJ. 2014;2: e369. doi:doi: 10.7717/peerj.369

89. Guerrero A, Pérez-García A. Shell anomalies in the European aquatic stem turtle *Pleurosternon bullockii* (Paracryptodira, Pleurosternidae). Diversity. 2021;13: 518. doi:10.3390/d13110518

90. Guerrero A, Pérez-García A. Ontogenetic development of the European basal aquatic turtle *Pleurosternon bullockii* (Paracryptodira, Pleurosternidae). Foss Rec. 2021;24: 357–377. doi:10.5194/fr-24-357-2021

91. de la Fuente MS, Umazano AM, Sterli J, Carballido JL. New chelid turtles of the lower section of the Cerro Barcino formation (Aptian-Albian?), Patagonia, Argentina. Cretac Res. 2011;32: 527–537. doi:10.1016/j.cretres.2011.03.007

92. Gaffney ES. The shell morphology of the Triassic turtle *Proganochelys*. Neues Jahrb für Geol und Paläontologie - Abhandlungen. 1985;170: 1–26.

93. Fraas E. *Proganochelys quenstedtii* Baur (*Psammochelys keuperina* Qu.). Einer neuer Fund der Keuperschildkröte aus dem Stubensandstein. Jahreshefte des Vereins für Vaterländische Naturkd Württemb. 1899;55: 401–423.

94. Quenstedt FA. *Psammochelys keuperina*. (Ψάμμος Sand, χέλυς Schildkröte.). Jahreshefte des Vereins für Vaterländische Naturkd Württemb. 1889;45: 120–130.

95. Scheyer TM, Klein N, Evers SW, Mautner A-K, Pabst B. First evidence of *Proganochelys quenstedtii* (Testudinata) from the Plateosaurus bonebeds (Norian, Late Triassic) of Frick, Canton Aargau, Switzerland. Swiss J Palaeontol. 2022;141: 17.

96. Werneburg I, Kyriakouli C, Szczygielski T. A surface scan of the “Tübingen Steinkern”, Holotype of *Proganochelys quenstedtii* (Testudinata), with some historical remarks. MorphoMuseuM. 2022;8: e168. doi:10.18563/journal.m3.168

97. Sulej T, Niedźwiedzki G, Bronowicz R. A new Late Triassic vertebrate fauna from Poland with turtles, aetosaurs, and coelophysoid dinosaurs. J Vertebr Paleontol. 2012;32: 1033–1041. doi:10.1080/02724634.2012.694384

98. Szczygielski T, Słowiak J, Dróżdż D. Shell variability in the stem turtles *Proterochersis* spp. PeerJ. 2018;6: e6134. doi:10.7717/peerj.6134

99. Szczygielski T, Sulej T. The early composition and evolution of the turtle shell (Reptilia, Testudinata). Palaeontology. 2019;62: 375–415.

100. Szczygielski T. Obscure by name: solving the enigma of *Chelytherium obscurum*, the first described Triassic turtle. Zool J Linn Soc. 2021;192: 1111–1122.

101. Szczygielski T, Słowiak J. Shell histology of the Triassic turtle, *Proterochersis porebensis*, provides novel insights about shell ankylosis. Comptes Rendus - Palevol. 2022;21: 619–679.

102. Czepiński Ł, Dróżdż D, Szczygielski T, Tałanda M, Pawlak W, Lewczuk A, et al. An Upper Triassic terrestrial vertebrate assemblage from the forgotten Kocury locality in southern Poland with a new aetosaur taxon. J Vertebr Paleontol. 2021;41: e1898977. doi:10.1080/02724634.2021.1898977

103. Fraas E. *Proterochersis*, eine pleurodire Schildkröte aus dem Keuper. Jahreshefte des Vereins für Vaterländische Naturkd Württemb. 1913;69: 13–30.

104. Karl H-V, Tichy G. *Murrhardtia staeschei* n. gen. n. sp. – eine neue Schildkröte aus der Oberen Trias von Süddeutschland. Joannea Geol und Paläontologie. 2000;2: 57–72.

105. Hirayama R. Oldest known sea turtle. Nature. 1998;392: 705–708.

106. Tong H, Buffetaut E, Suteethorn V. Middle Jurassic turtles from southern Thailand. Geol Mag. 2002;139: 687–697. doi:10.1017/S0016756802006805

107. Ye Y, Pi X. A new genus of Chengyuchelyidae from Dashanpu, Zigong, Sichuan. Vertebr Palasiat. 1997;7: 182–188.

108. Tong H, Danilov I, Ye Y, Ouyang H, Peng G. Middle Jurassic turtles from the Sichuan Basin, China: A review. Geol Mag. 2012;149: 675–695. doi:10.1017/S0016756811000859

109. Joyce WG, Rabi M, Clark JM, Xu X. A toothed turtle from the Late Jurassic of China and the global biogeographic history of turtles. BMC Evol Biol. 2016;16: 236. doi:10.1186/s12862-016-0762-5

110. Brinkman DB, Peng J-H. New material of *Sinemys* (Testudines, Sinemydidae) from the Early Cretaceous of China. Can J Earth Sci. 1993;30: 2139–2152.

111. Joyce WG. The first complete skeleton of *Solnhofia parsonsi* (Cryptodira, Eurysternidae) from the Upper Jurassic of Germany and its taxonomic implications. J Paleontol. 2000;74: 684–700. doi:10.1666/0022-3360(2000)074<0684

112. Case EC. A specimen of *Stylemys nebrascensis* Leidy, showing the bones of the feet and limbs. Contrib from Museum Paleontol Univ Michigan. 1936;5: 69–73.

113. Zangerl R. The vertebrate fauna of the Selma Formation of Alabama. Part IV. the turtles of the family Toxochelyidae. Fieldiana Geol Mem. 1953;3: 1–306. doi:10.5962/bhl.title.5224

114. Zhu H. Plastron reduction and associated myology in turtles, and its implications for functional morphology and natural history. Marshall University. 2011.

115. Drumheller SK, Maddox H, Stocker MR, Noto CR. Differentiating convergent pathologies in turtle shells using computed tomographic scanning of modern and fossil bone. Palaeontol Electron. 2023;26: a15.

116. Hay OP. Description of two new genera (*Echmatemys* and *Xenochelys*) and two new species (*Xenochelys formosa* and *Terrapene putnami*) of fossil turtles. Bull Am Museum Nat Hist. 1906;22: 27–31.

117. Borque JR. Fossil Kinosternidae from the Oligocene and Miocene of Florida, USA. In: Brinkman DB, Holroyd PA, Gardner JD, editors. Morphology and Evolution of Turtles. Dordrecht: Springer; 2012. pp. 459–475.

118. Rabi M, Zhou CF, Wings O, Ge S, Joyce WG. A new xinjiangchelyid turtle from the Middle Jurassic of Xinjiang, China and the evolution of the basipterygoid process in Mesozoic turtles. BMC Evol Biol. 2013;13: 203. doi:10.1186/1471-2148-13-203

119. Bona P, de la Fuente MS. Phylogenetic and paleobiogeographic implications of *Yaminuechelys maior* (Staesche, 1929) new comb., a large long-necked chelid turtle from the early Paleocene of Patagonia, Argentina. J Vertebr Paleontol. 2005;25: 569–582.

120. Danilov IG, Parham JF. A redescription of “*Plesiochelys*” *tatsuensis* from the Late Jurassic of China, with comments on the antiquity of the crown clade Cryptodira. J Vertebr Paleontol. 2006;26: 573–580. doi:10.1671/0272-4634(2006)26[573:AROPTF]2.0.CO;2

121. Jaekel O. Über die Wirbeltierfunde aus der oberen Trias von Halberstadt. Paläontologische Zeitschrift. 1914;1: 155–215.

122. Jaekel O. Die Wirbeltierfunde aus dem Keuper von Halberstadt. Serie II. Testudinata. Palaeontol Zeitschrift. 1916;2: 88–214. doi:10.1007/BF03160328
