# Supplementary figures and images for: The Triassic turtle of Thailand – revision of ‘Proganochelys’ ruchae
Source: PLoS One. 2025 Mar 19;20(3):e0316338. doi: 10.1371/journal.pone.0316338 (PMC12279380; doi:10.1371/journal.pone.0316338)

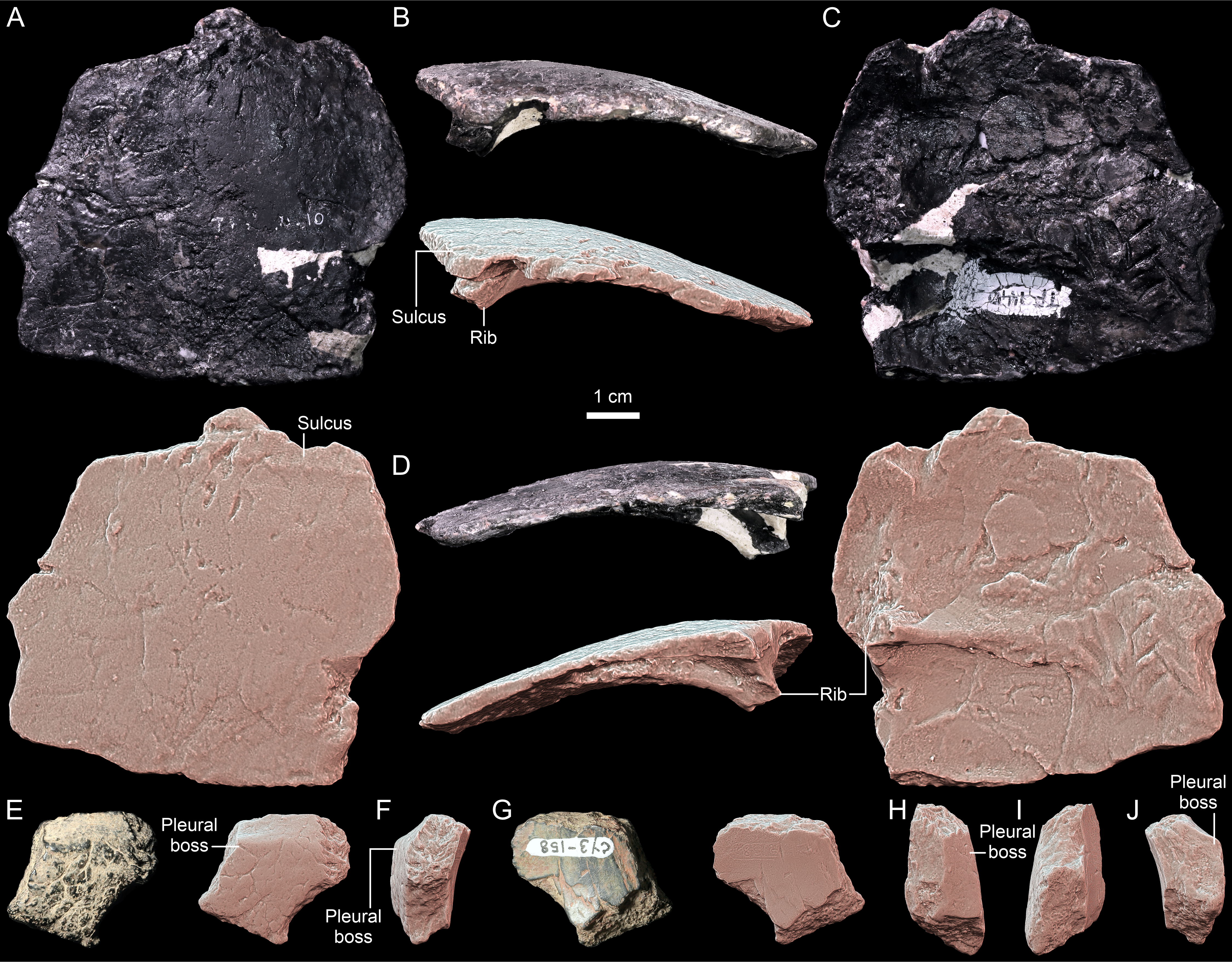

Supplement: S1 Archive — ZIP archive with Figs 1–4 in full resolution. (ZIP) [file pone.0316338.s005.zip › Fig4.tif]

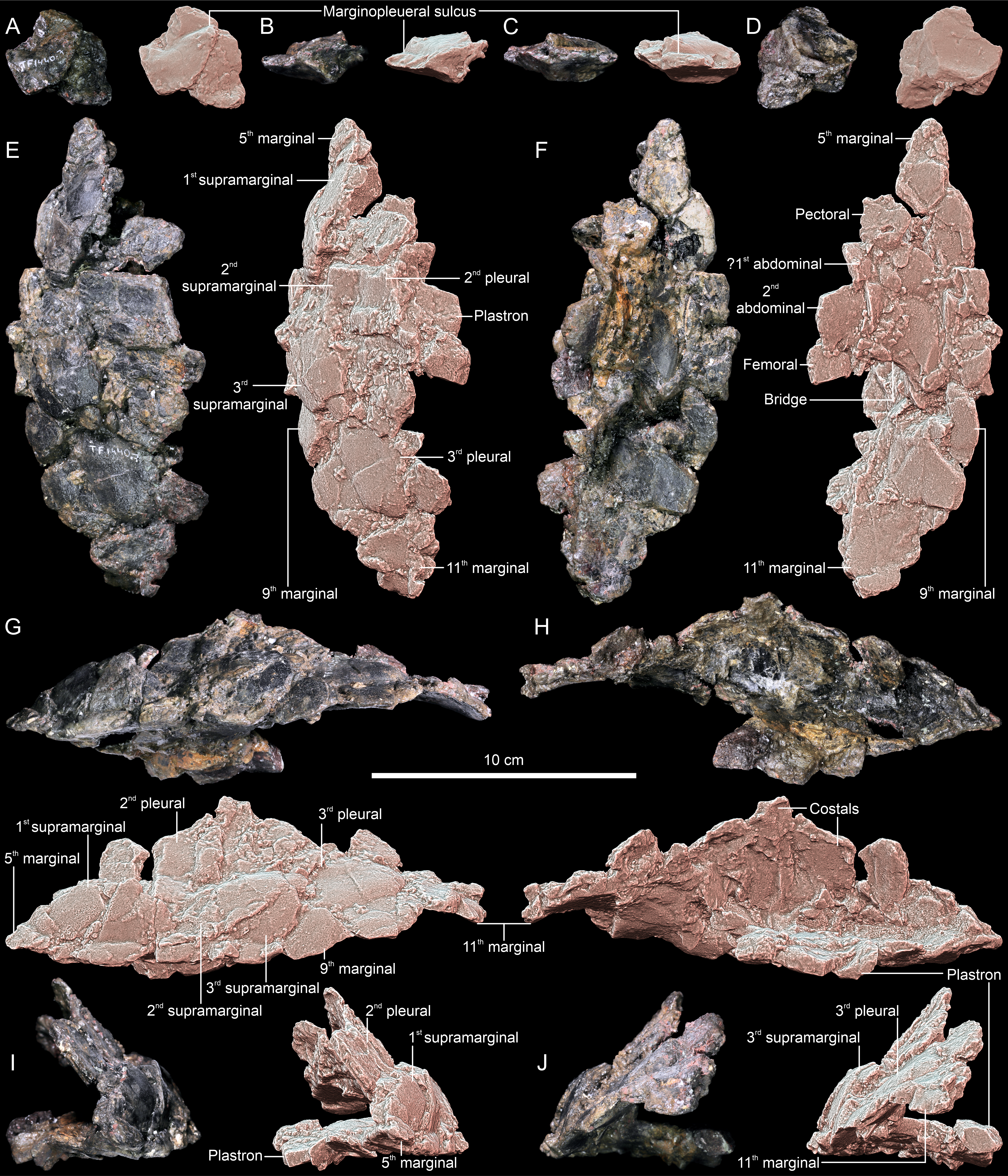

Supplement: S1 Archive — ZIP archive with Figs 1–4 in full resolution. (ZIP) [file pone.0316338.s005.zip › Fig1.tif]

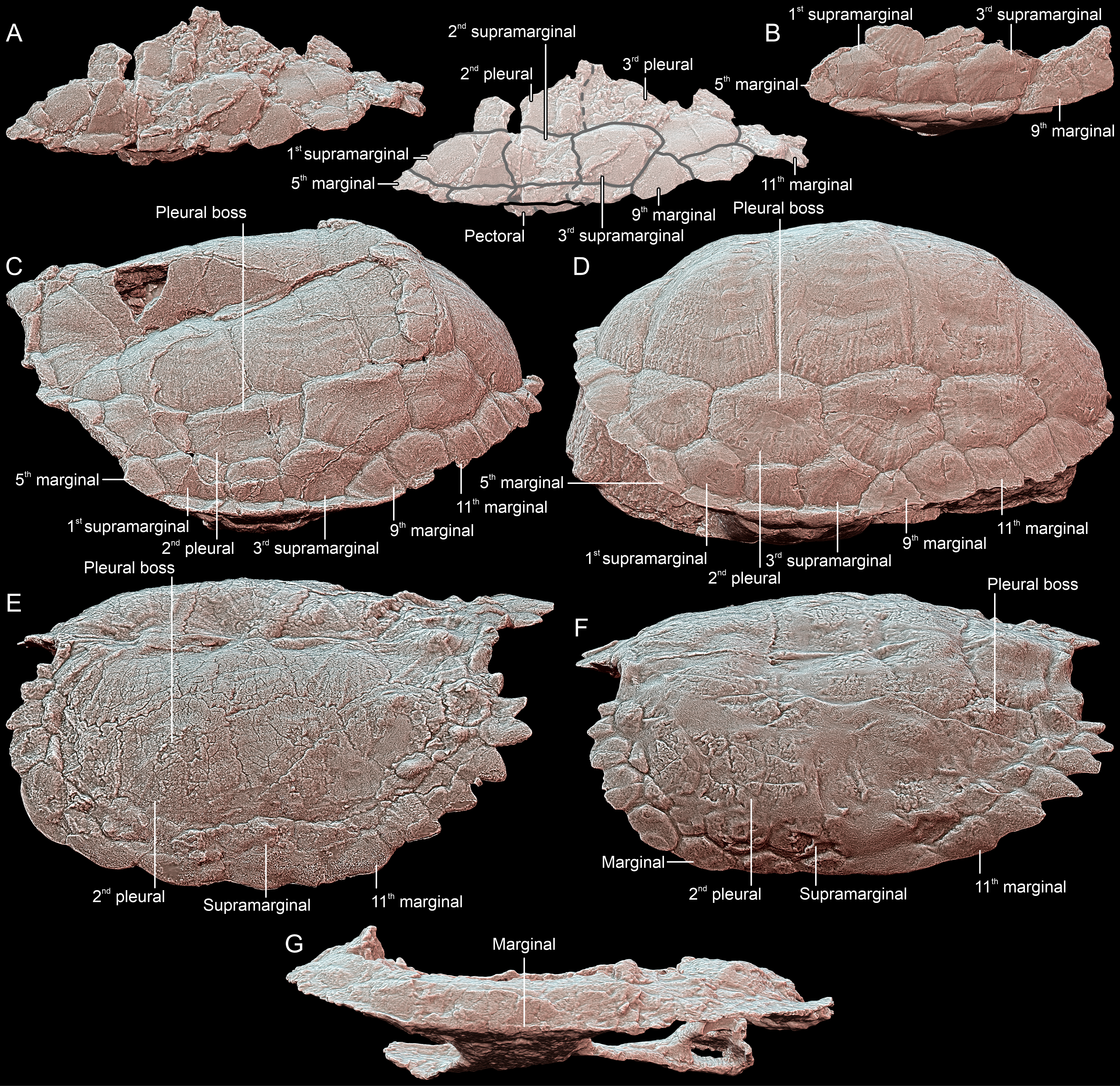

Supplement: S1 Archive — ZIP archive with Figs 1–4 in full resolution. (ZIP) [file pone.0316338.s005.zip › Fig2.tif]

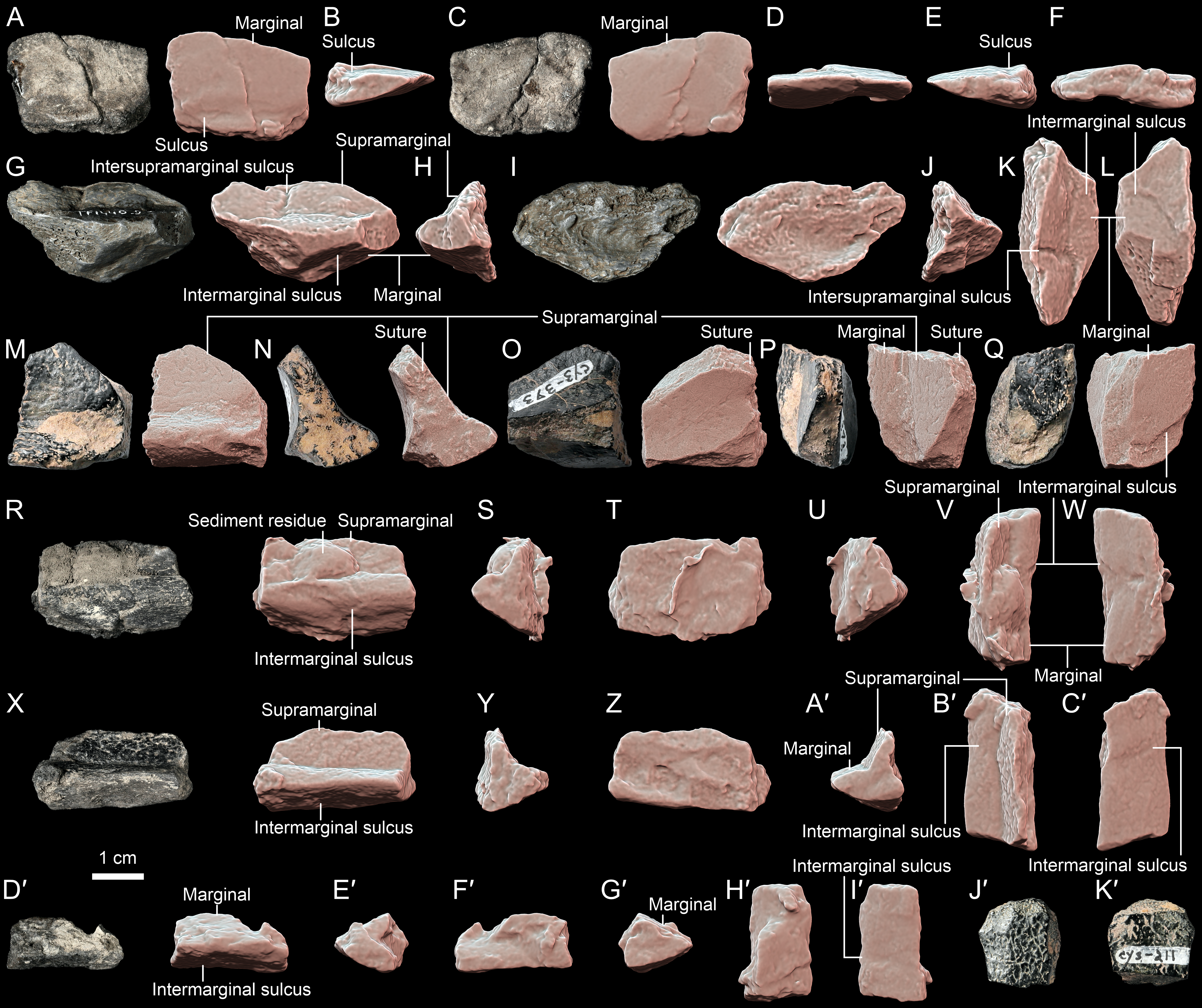

Supplement: S1 Archive — ZIP archive with Figs 1–4 in full resolution. (ZIP) [file pone.0316338.s005.zip › Fig3.tif]

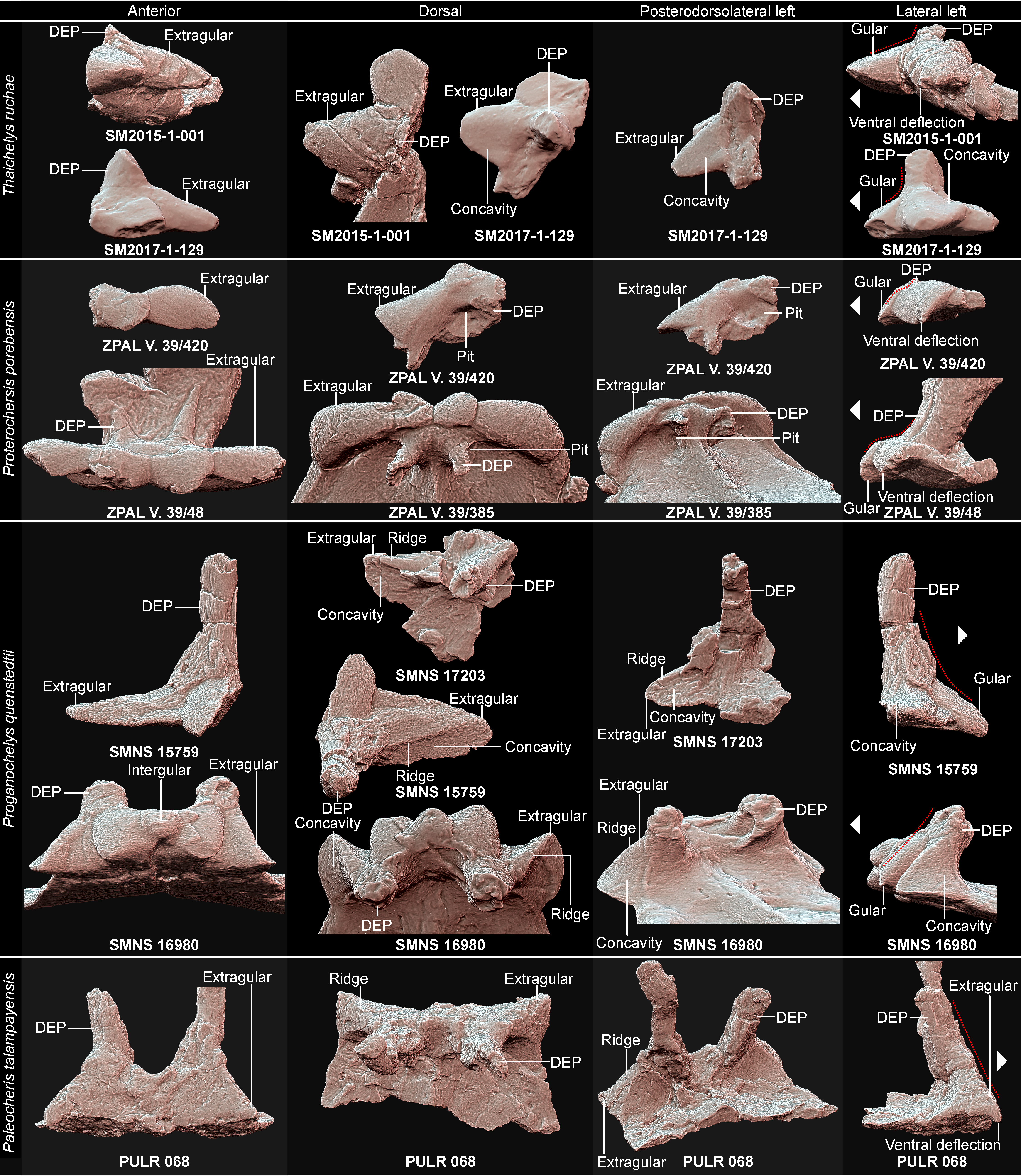

Supplement: S2 Archive — ZIP archive with Figs 5–9 in full resolution. (ZIP) [file pone.0316338.s006.zip › Fig9.tif]

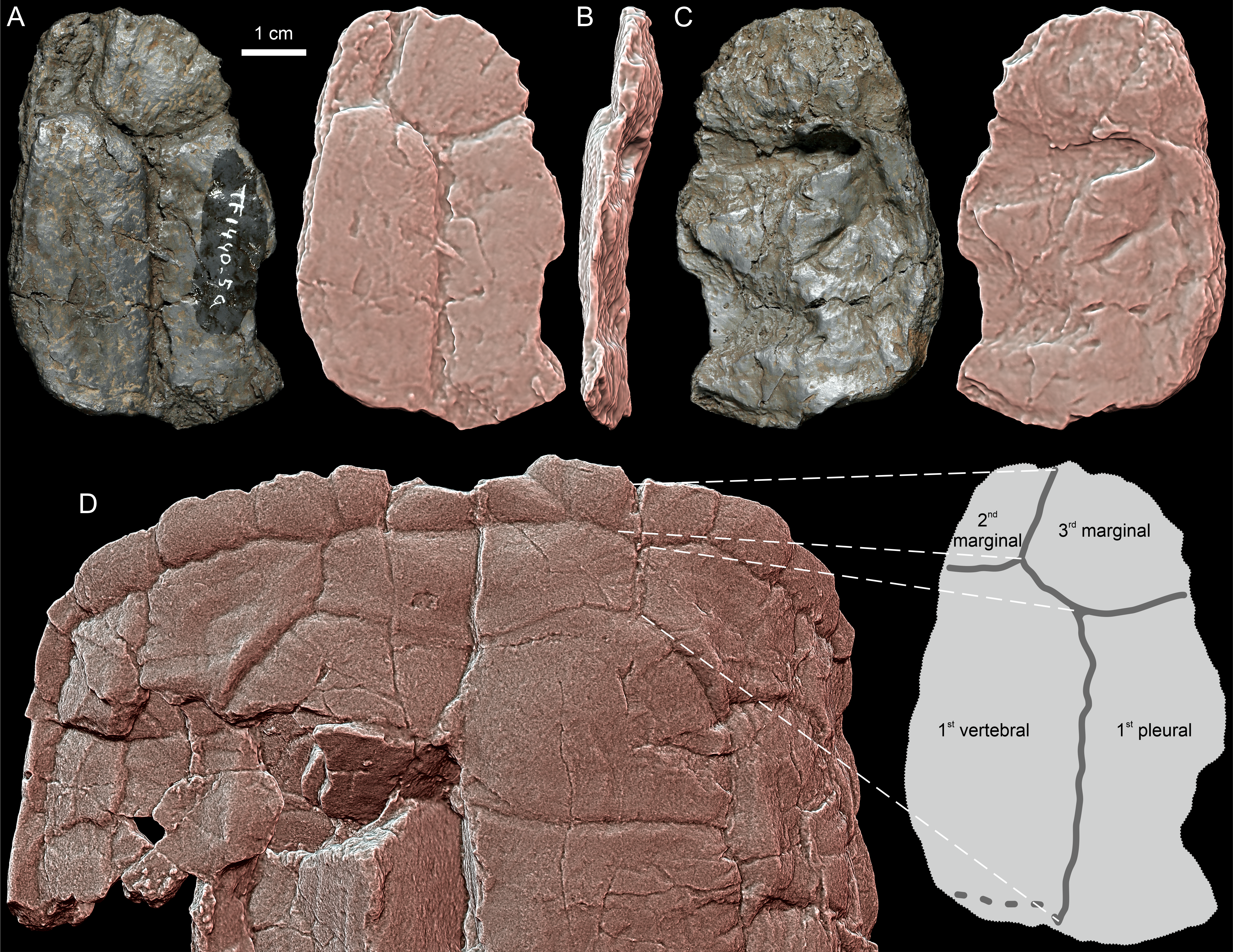

Supplement: S2 Archive — ZIP archive with Figs 5–9 in full resolution. (ZIP) [file pone.0316338.s006.zip › Fig5.tif]

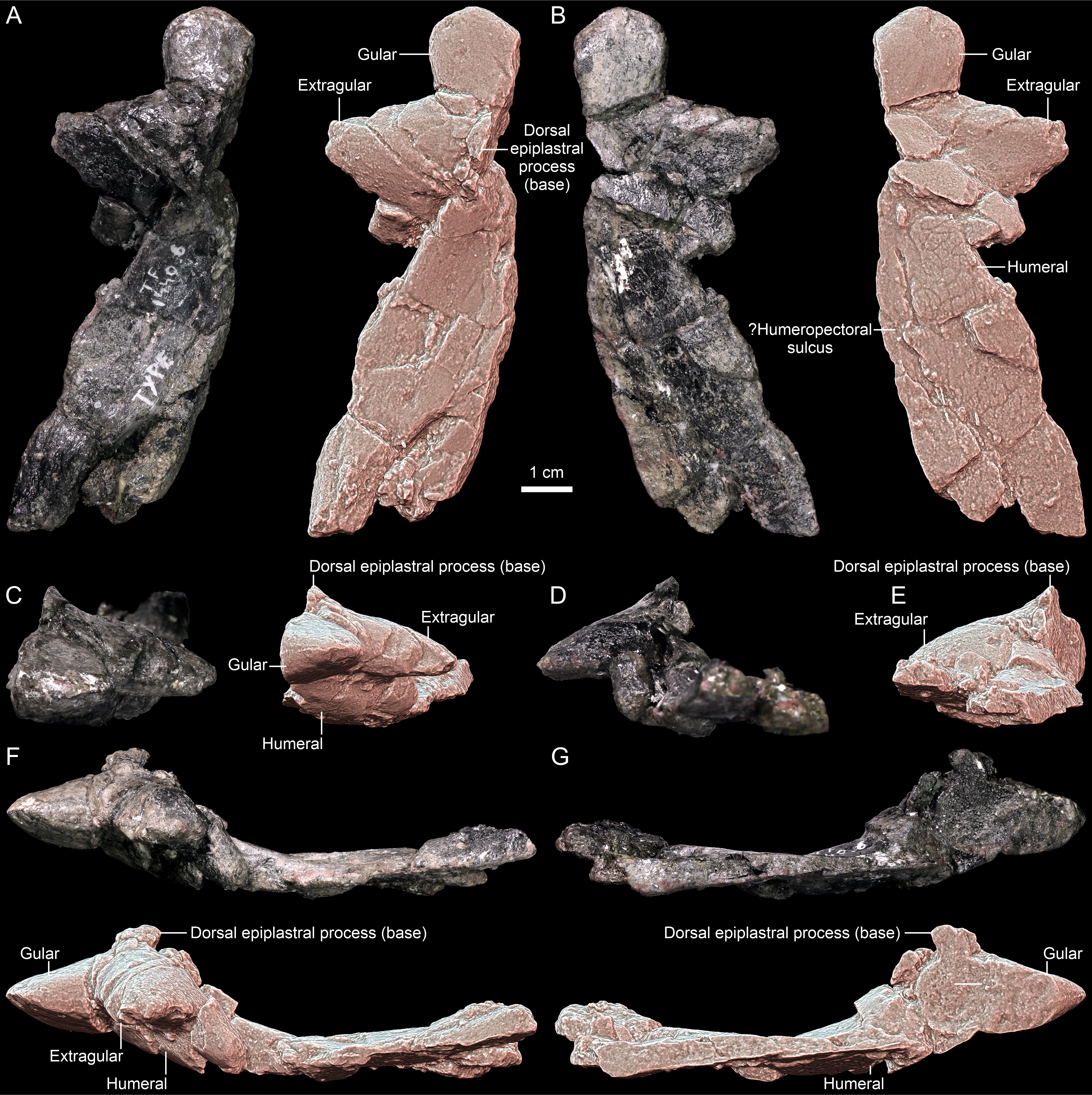

Supplement: S2 Archive — ZIP archive with Figs 5–9 in full resolution. (ZIP) [file pone.0316338.s006.zip › Fig6.tif]

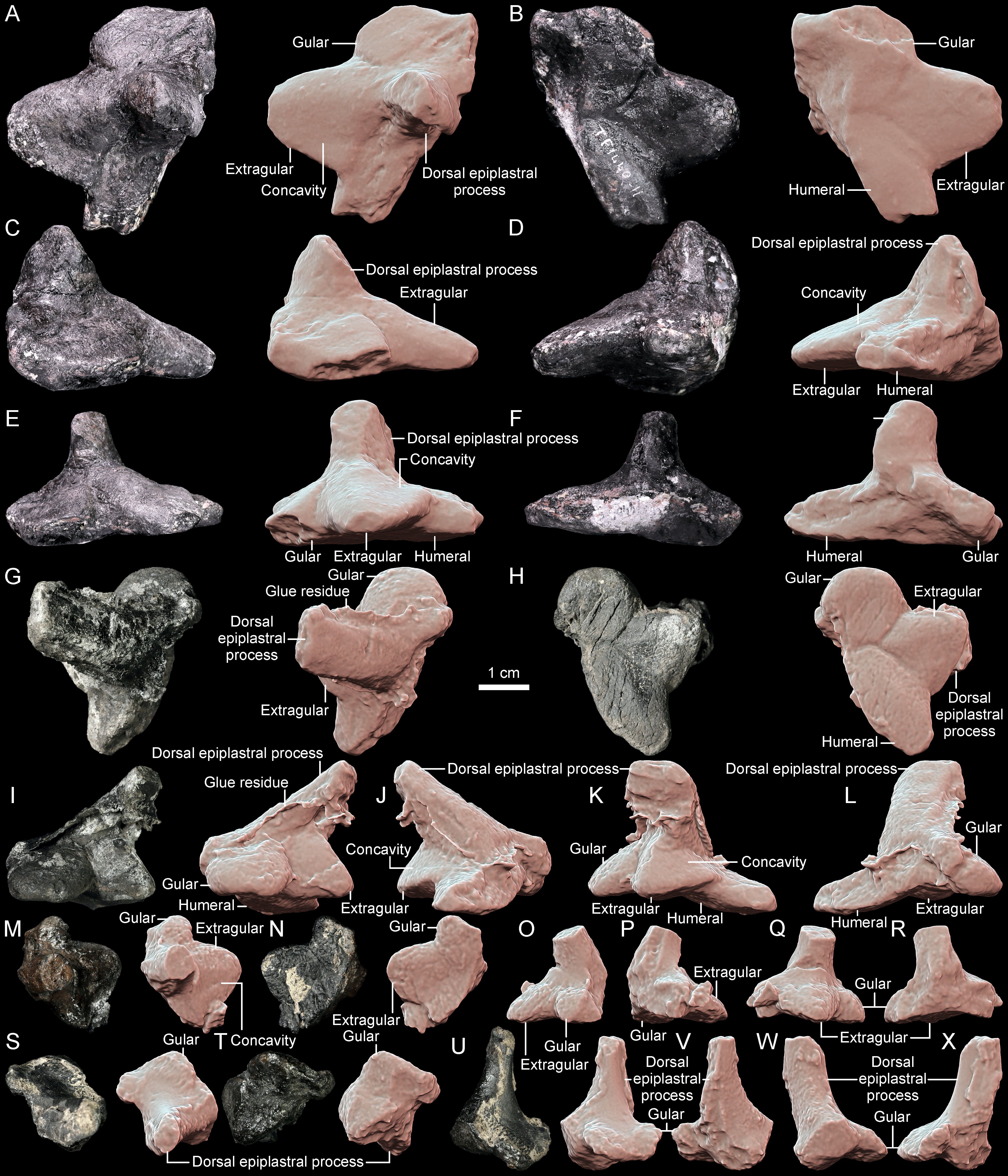

Supplement: S2 Archive — ZIP archive with Figs 5–9 in full resolution. (ZIP) [file pone.0316338.s006.zip › Fig7.tif]

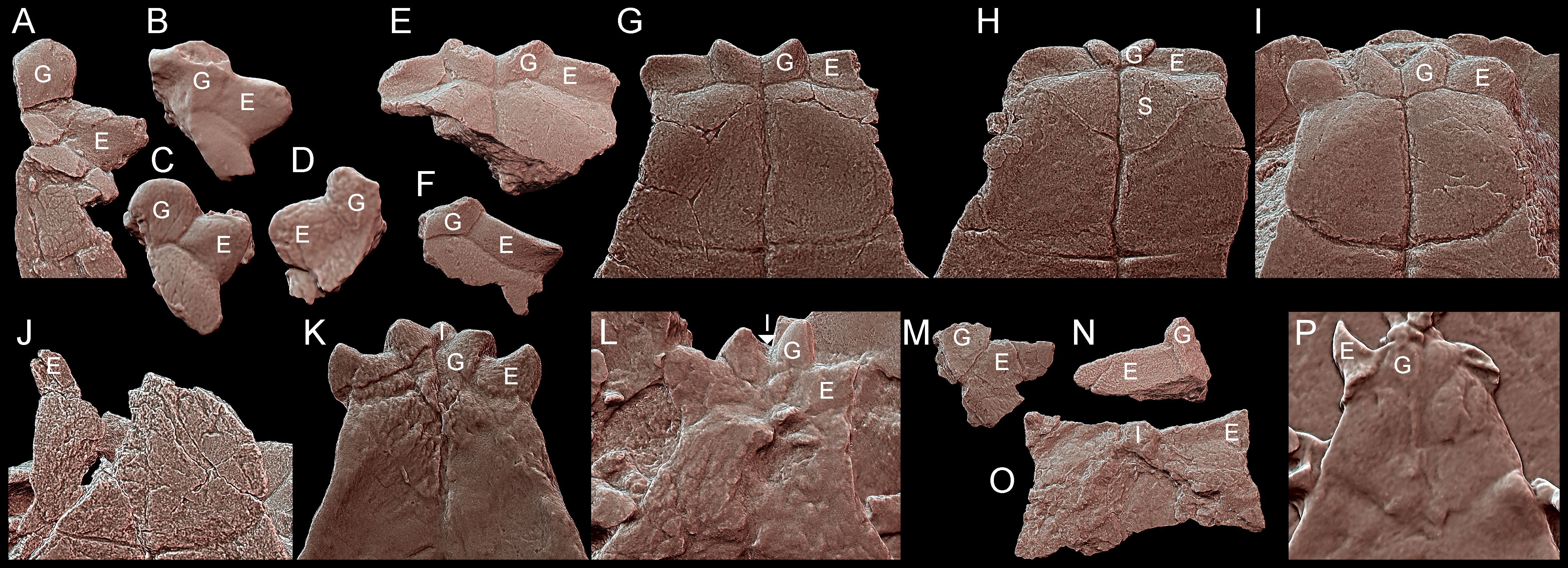

Supplement: S2 Archive — ZIP archive with Figs 5–9 in full resolution. (ZIP) [file pone.0316338.s006.zip › Fig8.tif]

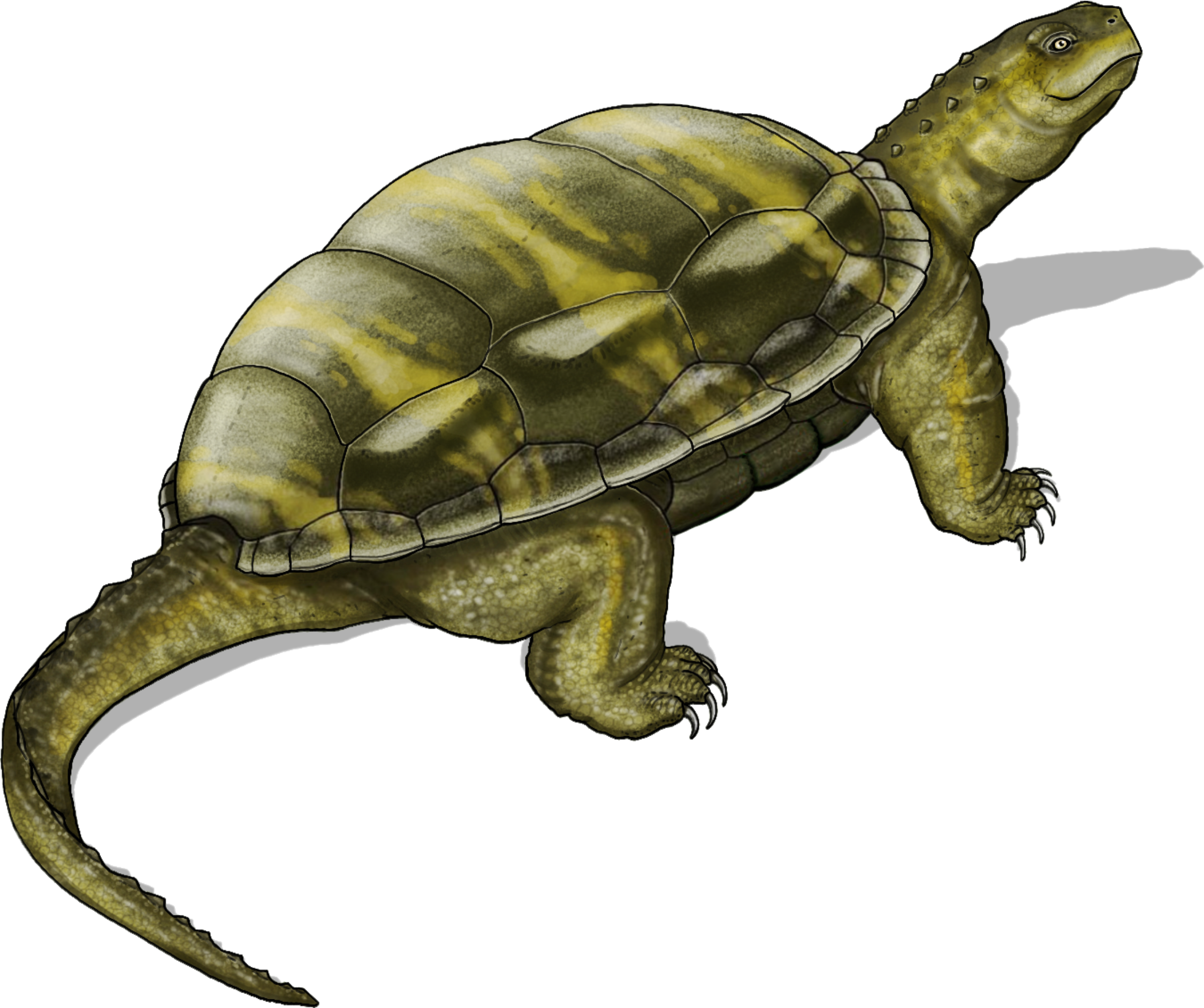

Supplement: S3 Archive — ZIP archive with Figs 10–18 in full resolution. (ZIP) [file pone.0316338.s007.zip › Fig18.tif]

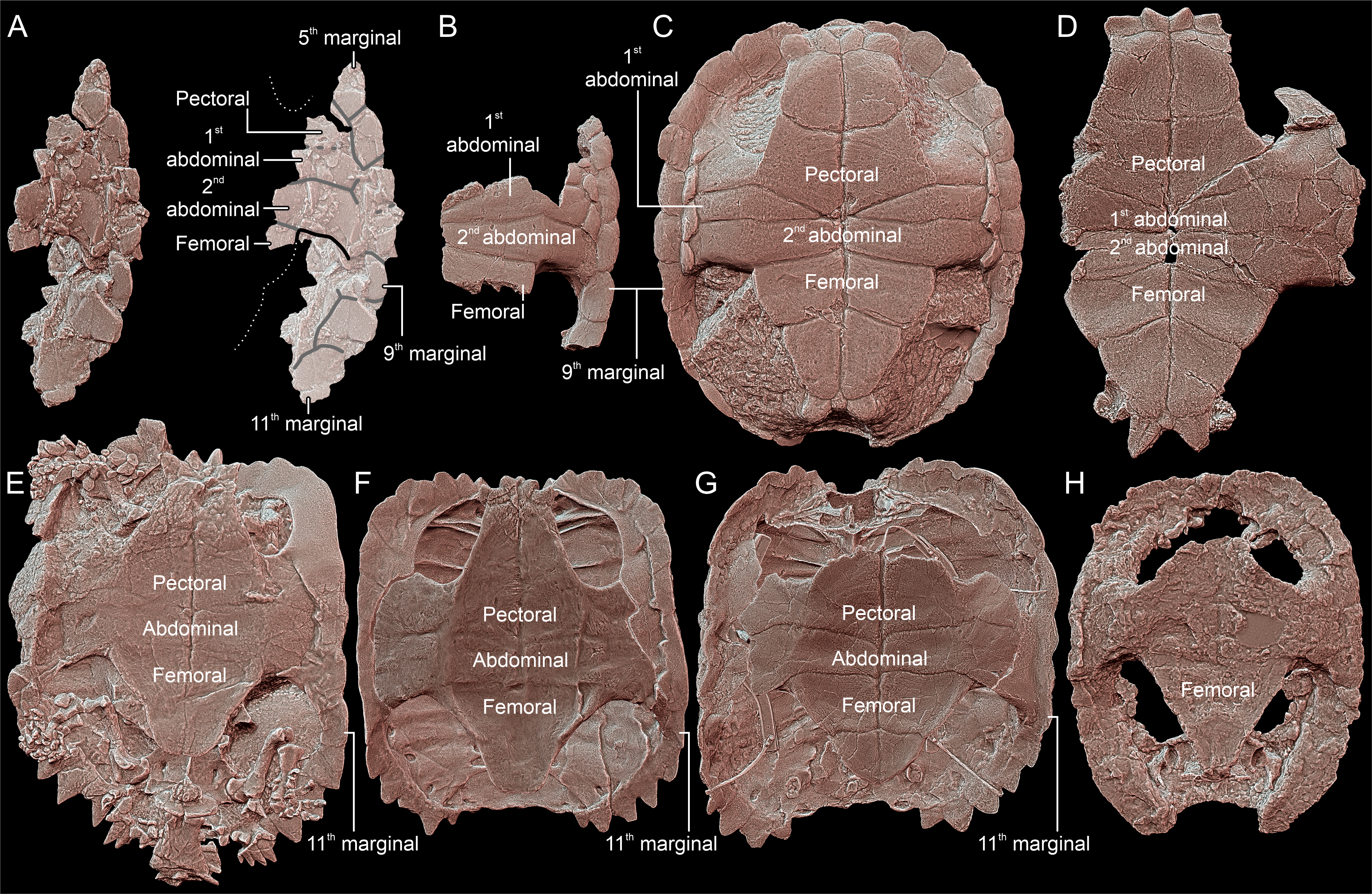

Supplement: S3 Archive — ZIP archive with Figs 10–18 in full resolution. (ZIP) [file pone.0316338.s007.zip › Fig10.tif]

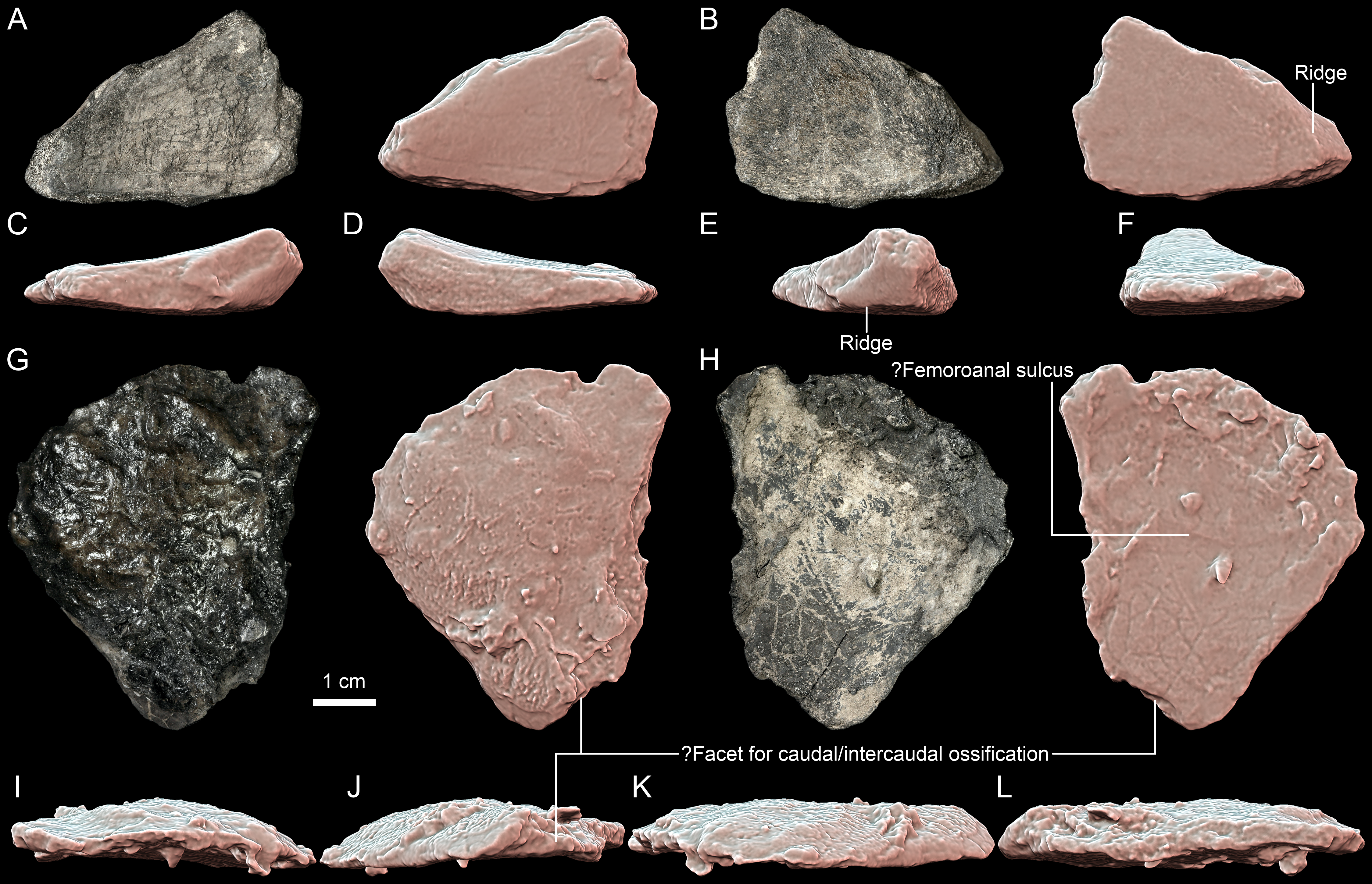

Supplement: S3 Archive — ZIP archive with Figs 10–18 in full resolution. (ZIP) [file pone.0316338.s007.zip › Fig11.tif]

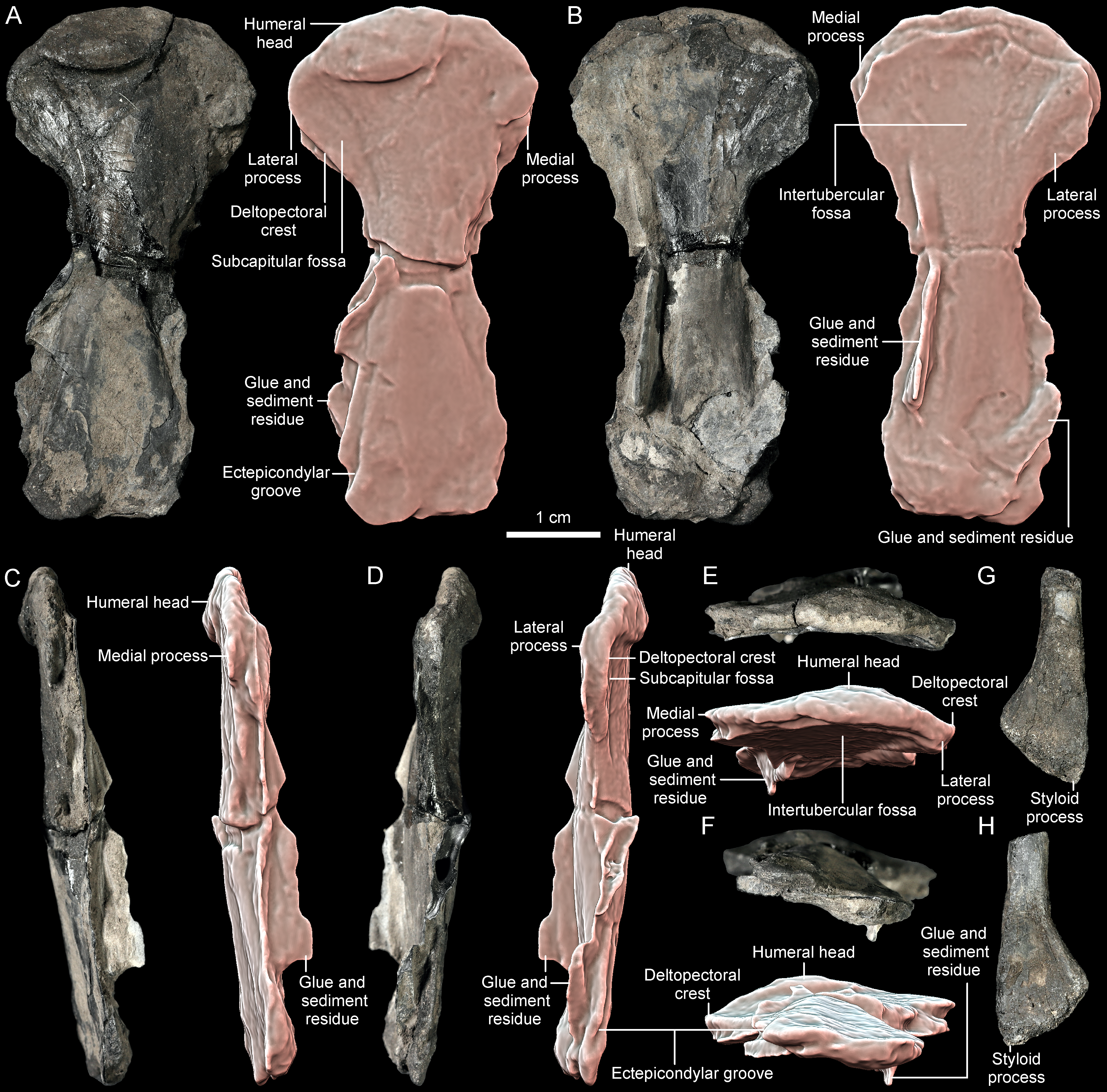

Supplement: S3 Archive — ZIP archive with Figs 10–18 in full resolution. (ZIP) [file pone.0316338.s007.zip › Fig12.tif]

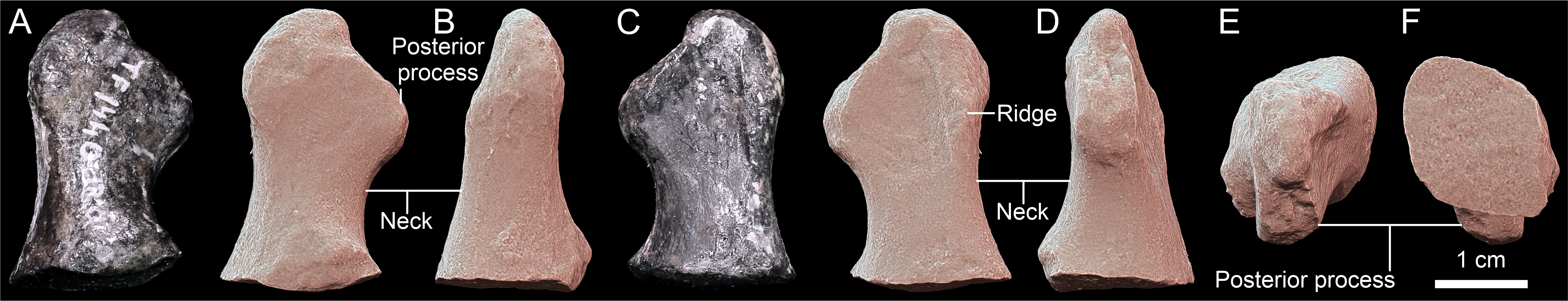

Supplement: S3 Archive — ZIP archive with Figs 10–18 in full resolution. (ZIP) [file pone.0316338.s007.zip › Fig13.tif]

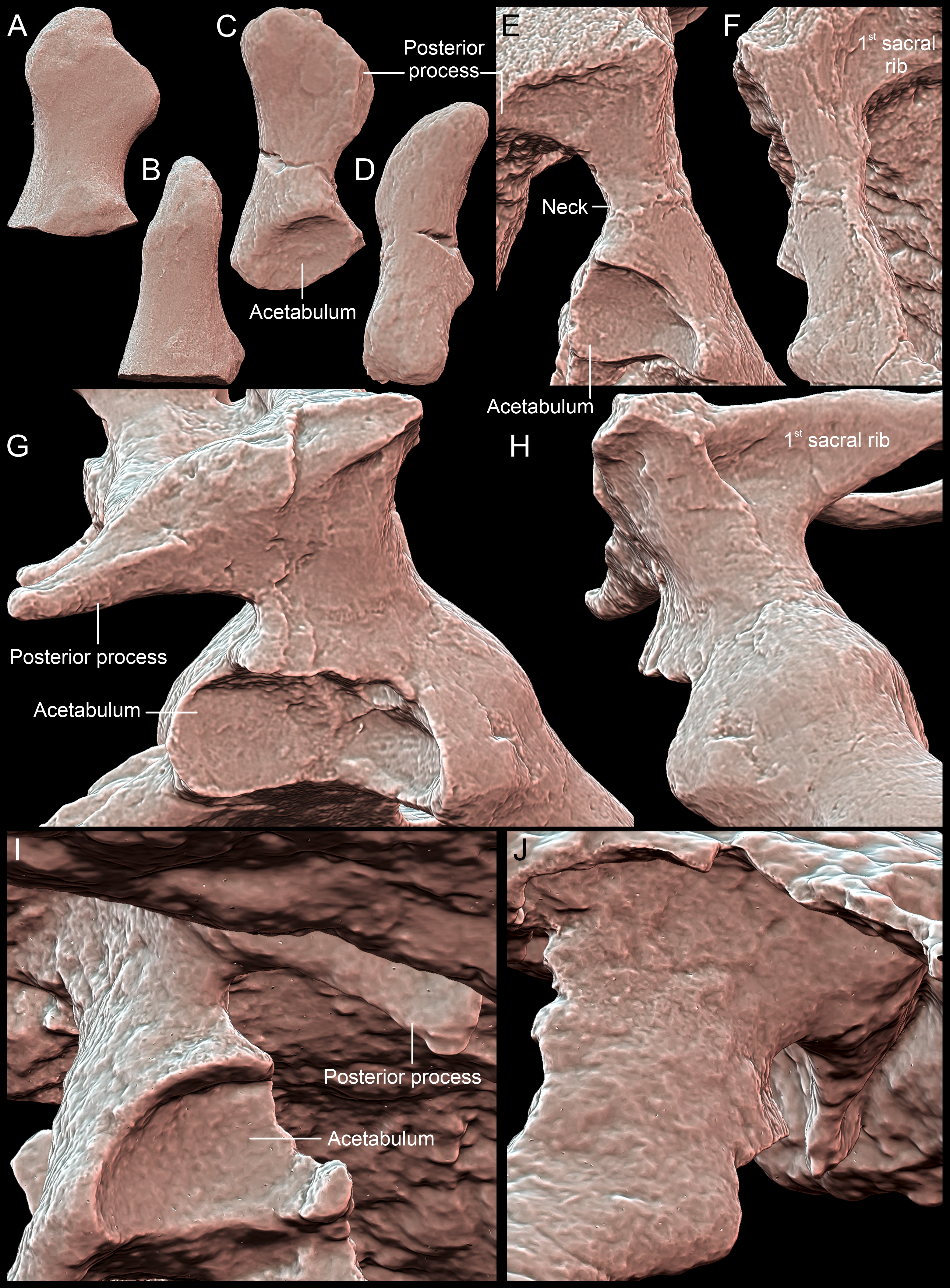

Supplement: S3 Archive — ZIP archive with Figs 10–18 in full resolution. (ZIP) [file pone.0316338.s007.zip › Fig14.tif]

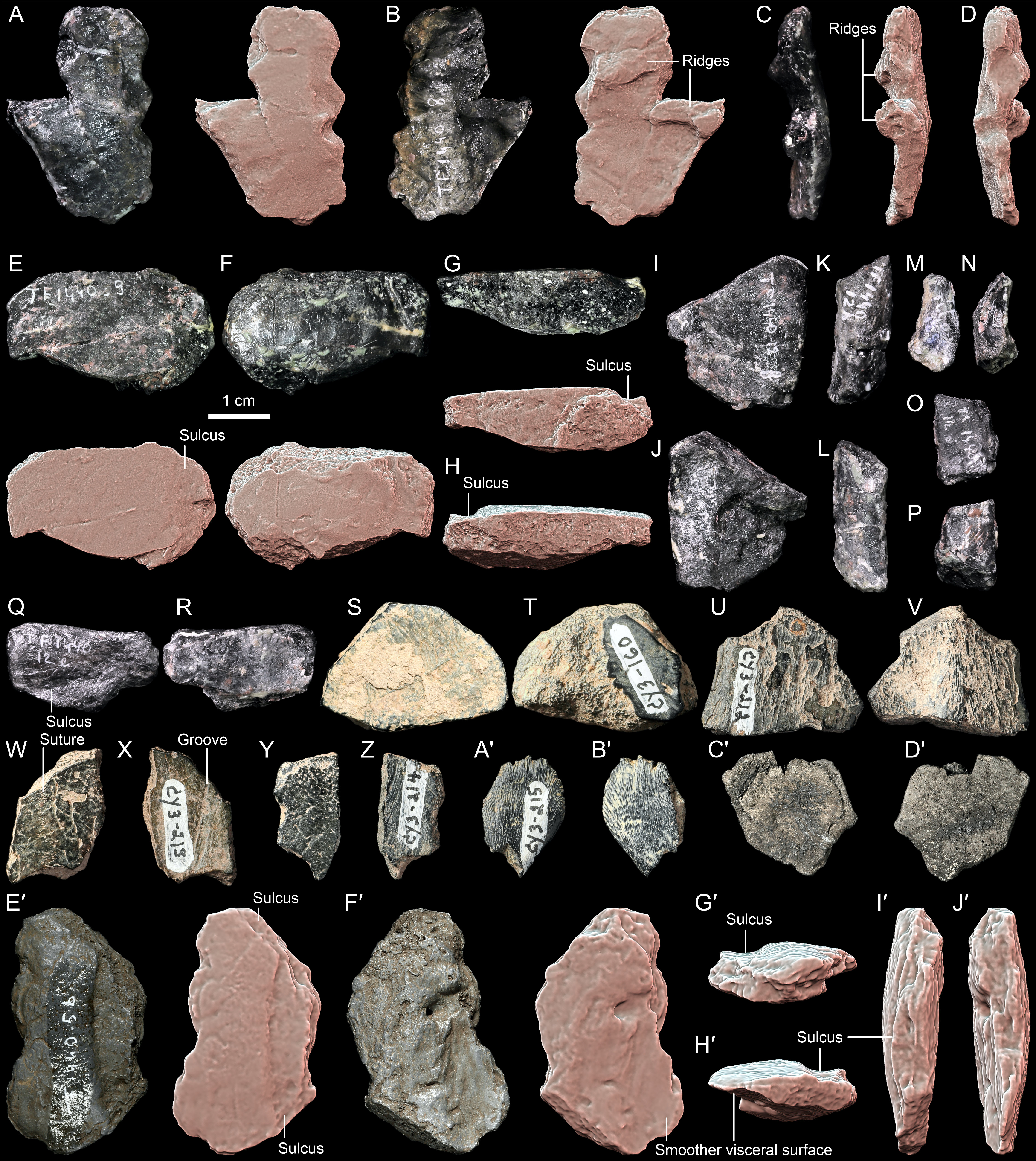

Supplement: S3 Archive — ZIP archive with Figs 10–18 in full resolution. (ZIP) [file pone.0316338.s007.zip › Fig15.tif]

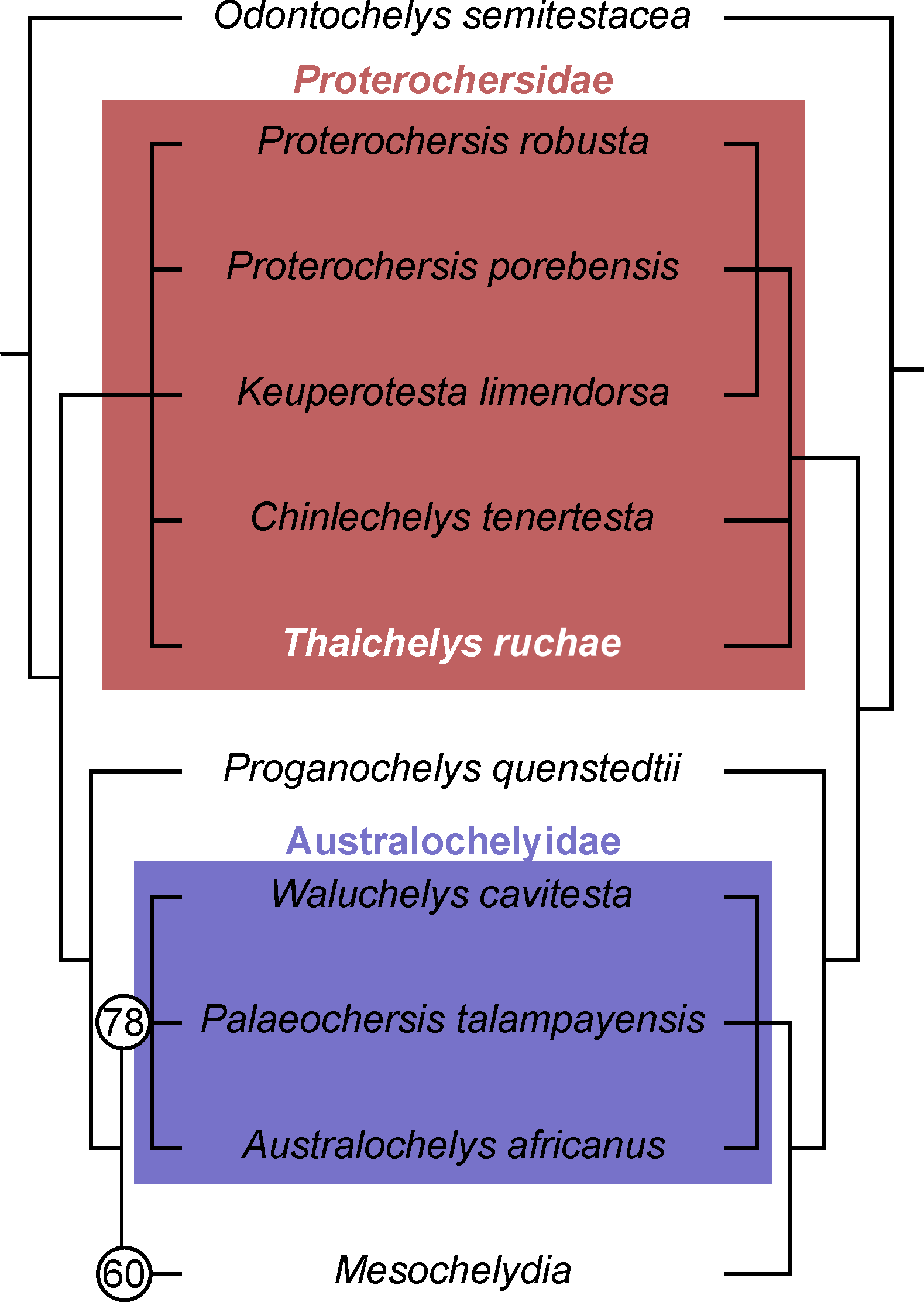

Supplement: S3 Archive — ZIP archive with Figs 10–18 in full resolution. (ZIP) [file pone.0316338.s007.zip › Fig16.tif]

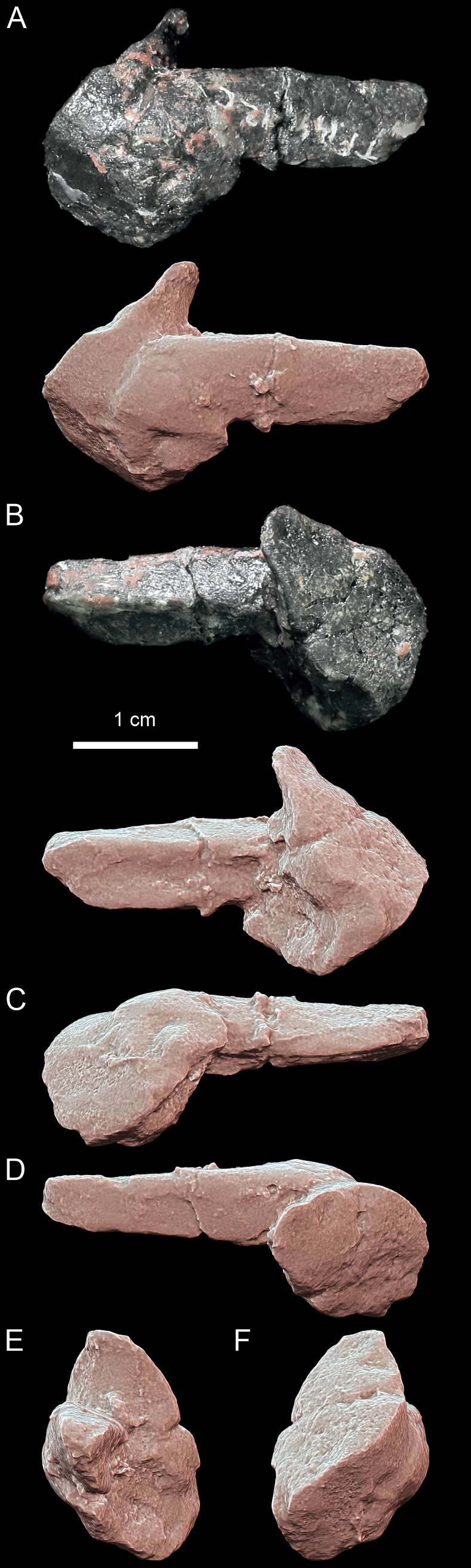

Supplement: S3 Archive — ZIP archive with Figs 10–18 in full resolution. (ZIP) [file pone.0316338.s007.zip › Fig17.tif]
